# Oxazole-Based Ferroptosis Inhibitors with Promising Properties to Treat Central Nervous System Diseases

**DOI:** 10.1021/acs.jmedchem.4c03149

**Published:** 2025-02-06

**Authors:** Camilla Scarpellini, Greta Klejborowska, Caroline Lanthier, Ariane Toye, Karolina Musiałek, Emily Van San, Magali Walravens, Maya Berg, Behrouz Hassannia, Pieter Van der Veken, Hans De Winter, Tom Vanden Berghe, Koen Augustyns

**Affiliations:** 1 Laboratory of Medicinal Chemistry, Department of Pharmaceutical Sciences, Faculty of Pharmaceutical, Biomedical and Veterinary Sciences, University of Antwerp, Antwerp 2610, Belgium; 2 Cell Death Signaling Lab, Department of Biomedical Sciences, University of Antwerp, Antwerp 2610, Belgium; 3 Infla-Med Centre of Excellence, University of Antwerp, Antwerp 2610, Belgium; 4 Molecular Signalling and Cell Death Unit, VIB Center for Inflammation Research, Ghent 9052, Belgium; 5 Department of Biomedical Molecular Biology, Ghent University, Ghent 9000, Belgium; 6 Department of Medical Chemistry, Faculty of Chemistry, Adam Mickiewicz University, Poznań 61-614, Poland

## Abstract

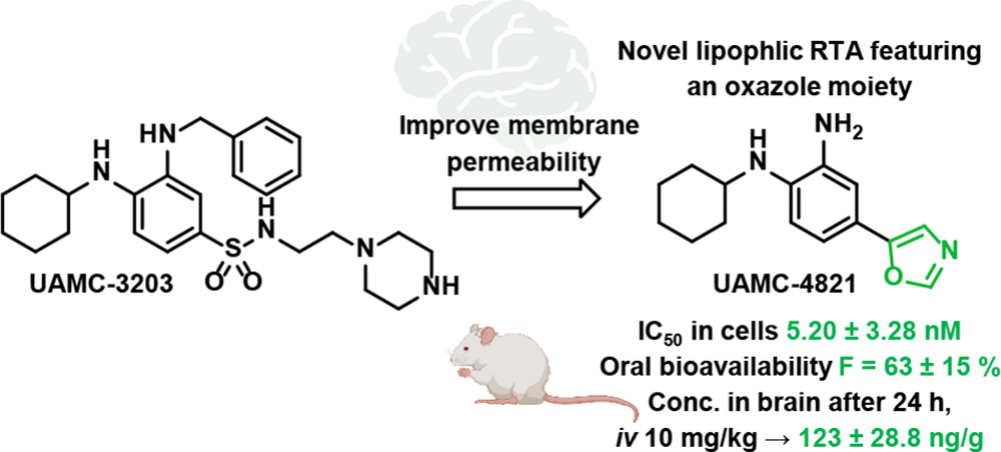

Ferroptosis plays an important role in the occurrence and development of many diseases, including neurodegenerative diseases. Thus, ferroptosis inhibitors able to cross the blood-brain barrier may have therapeutic potential. The best ferroptosis inhibitors so far are lipophilic radical trapping antioxidants (RTAs) that block lipid peroxidation in membranes. Several generations of ferrostatins have been synthesized, among which **UAMC-3203** showed high potency in animal models with improved properties compared to ferrostatin-1. To further improve its pharmacokinetics properties, drug-likeness, and permeability, we modified **UAMC-3203** by decreasing the size of the molecule and reducing its polarity by replacing the sulfonamide first by amide groups and subsequently by isosteric oxazoles. Herein, we present the design, synthesis, and biological evaluation of a novel series of oxazole RTAs with high potency, excellent oral bioavailability, and high concentrations in brain tissue.

## Introduction

Discovered in 2012 by Stockwell’s group, ferroptosis is an iron-dependent form of regulated cell death (RCD), more precisely a form of necrosis, characterized by the accumulation of lipid peroxides leading to cell membrane damage and disruption.^1^ Since then, an exponential growth in the number of publications and groups working in the ferroptosis field was observed.^2^ It became clear that not only the iron-catalyzed lipid peroxidation regulated by the Fenton chemistry is involved in ferroptosis, but also multiple cellular metabolic pathways play a role.^3^ Lipid peroxidation is considered the main hallmark of ferroptosis.^4^ Phopsolipids are the main components of the cellular membrane and therefore the propagation of (phospho)lipid peroxidation can lead to disruption of the cell membrane integrity and many different pathologies such as acute kidney injury, ishemia reperfusion injury, corneal dysfunction and many other.^5−8^ Recent studies have identified ferroptosis hallmarks across several neurodegenerative diseases, including Alzheimer’s disease, Parkinson’s disease, Huntington’s disease, amyotrophic lateral sclerosis, and multiple sclerosis.^9−12^ These findings support the growing hypothesis that ferroptosis may play a key role in the progression of these conditions.^13^ The process starts from polyunsaturated fatty acid (PUFAs) that are incorporated into cell membranes by long-chain-fatty-acid—CoA ligase 4 (ACSL4) and lysophosphatidylcholine acyltransferase 3 (LPCAT3), respectively.^14−17^ Then, lipid peroxides formation can be enzymatically mediated by lipoxygenases (LOXs) or cytochrome P450 oxidoreductase (POR).^18,19^ The nonenzymatic generation of lipid peroxides depends on the intracellular availability of the labile iron pool.^19,20^ In physiological conditions, glutathione peroxidase 4 (GPX4) is responsible for transforming the reactive lipid peroxides into the corresponding inactive lipid alcohol.^21^ In order to exert its mechanism of action, GPX4 requires its catalytic selenocysteine residue and two electrons provided by the cofactor glutathione (GSH) or low molecular thiols (cysteine) and protein thiols.^22^ During reduction, GSH is oxidized to glutathione disulfide (GSSG) and the reduced form can then be reobtained via the enzyme glutathione reductase.^23^ The pivotal role of GPX4 is to protect the phospholipid bilayers of the cell membrane from lipid peroxide accumulation and the consequent cell membrane damage, disruption and final ferroptosis.^24^ Another GSH-independent regulator of ferroptosis, which works in parallel to GPX4, is ferroptosis suppressor protein 1 (FSP1).^25^ A new regulatory pathway involving FSP1 emerged recently as a key element of ferroptosis metabolism, such as the FSP1-CoQ10-NAD(P)H axis.^26^ FSP1 is also involved in the nonclassical vitamin K oxidation–reduction cycle, making it a promising therapeutic target.^27^ In pathological conditions, when GPX4 and FSP1 defense pathways are interrupted, the use of ferroptosis inhibitors can help control the progression of the process and the propagation of lipid peroxidations.^1,28,29^

One of the most common strategies to tackle lipid peroxides formation is the use of lipophilic RTAs.^30,31^ Ferrostatin 1 (Fer-1) is the first potent ferroptosis inhibitor reported in 2012 by Dixon and Skouta, which can control ferroptosis in cells.^1,32^ Initially considered as lead compound, Fer-1 demonstrated even some activity *in vivo*.^33,34^ However, it showed also several constraints linked with the presence of a labile ester moiety which limits its application *in vivo*.^35^ In 2018, we reported a novel Fer-1 analog, UAMC-3203, which showed improved potency, stability and solubility compared to Fer-1.^36,37^ We replaced the ester moiety with a more stable sulfonamide substituted with ethyl piperazine in order to improve the solubility. The addition of a benzyl moiety on the free NH_2_ of Fer-1, successfully improved the stability and the potency of our analog. More interestingly, our compound showed lack of toxicity after daily administration over four weeks in an *in vivo* mouse model.^38^ Currently, UAMC-3203 is one of the most active RTAs used to investigate different ferroptosis-driven disease models *in vitro* and *in vivo*.^39−43^ However, UAMC-3203 presented limited oral bioavailability and blood-brain barrier (BBB) permeability preventing the use of our lead compound in neurodegenerative disease models. To further improve the properties of UAMC-3203 and to explore the potential of ferroptosis inhibitors in neurodegenerative diseases, we designed a novel series of small lipophilic RTAs. We assessed the antiferroptotic activity *in vitro* and determined their solubility. The activity as RTAs of the novel series was experimentally determined via the fluorescence-enabled inhibited autoxidation (FENIX) assay and finally, selected compounds were tested for their stability in human and mouse microsomes. The most promising compounds were subjected to the MDCK-MDR1 permeability assay to experimentally assess their BBB-crossing potential, followed by the determination of the pharmacokinetics (PK) profile of the best compounds.^44^

## Results and Discussion

### Compound Design

To improve the poor oral bioavailability and blood-brain barrier permeability of UAMC-3203 and analogs, we hypothesized that decreasing the size of the molecule, reducing its basicity and reducing the number of hydrogen bond donors (HBD) might improve membrane absorption and hence PK properties of our compounds. These are important parameters contributing to the CNS-MPO score, which is predictive of central nervous system (CNS) activity.^45^ Since we previously reported a series of amide RTAs with promising properties, and also Ji et al. reported antiferroptosis activity of formylpiperazine analogs of Fer-1, a first step in our design was to replace the sulfonamide ethyl linker with a series of amides without an ethyl linker, where the heterocyclic solubility-enhancing group is directly linked to the amide.^36,46^ This reduces molecular weight (MW), HBD and basicity. Additionally, we explored the impact of replacing the (fluoro)benzyl moiety with cyclohexyl and cyclopentyl groups to further improve metabolic stability.^46^ In subsequent steps, we investigated the introduction of a 5-membered heterocycle as a bioisostere of an amide or sulfonamide to further reduce MW, HBD and basicity, while maintaining the optimal lipophilicity and solubility of UAMC-3203.

**Figure 1:**
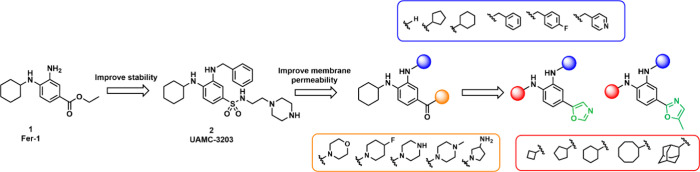
Representation of the benzamide and oxazole series of Fer-1 (**1**) analogues designed and synthesized based on the scaffold of UAMC-3203 (**2**).

Moreover, in support of our hypothesis, Stockwell and co-workers recently claimed in a patent the potential of oxadiazole RTAs for the treatment of different ferroptosis-driven CNS diseases.^47^ Lu et al. reported that some tetrazole-based Fer-1 analogs demonstrated high potency in the low nanomolar range.^48^ The use of heterocycles is a common practice in medicinal chemistry and oxazole-containing compounds have shown broad potential.^49^ Among the oxazole synthetic procedures that have been reported, we selected the ferric chloride-mediated cyclization and the Van Leusen procedure for the synthesis of the 5-methyl-oxazol-2-yl and oxazol-5-yl series, respectively.^50,51^ The above-described design strategy is summarized in Figure 1.

**Scheme 1:**
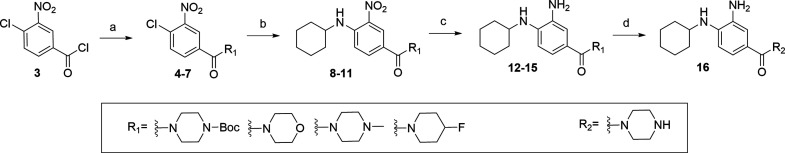
Synthetic Route for the Synthesis of Benzamides with Free −NH_2_ Group (**13**-**16**) Reaction conditions: (a) respective amine, DCM, pyridine, rt, 16 h (53–98%); (b) cyclohexylamine, DIPEA, ACN, 80 °C, 16 h (37–91%); (c) 20% Pd(OH)**_2_**/C, H**_2_**, MeOH, rt, 16 h (36–85%); (d) HCl in dioxane 4 M, DCM, rt, 2 h (74%).

**Scheme 2:**
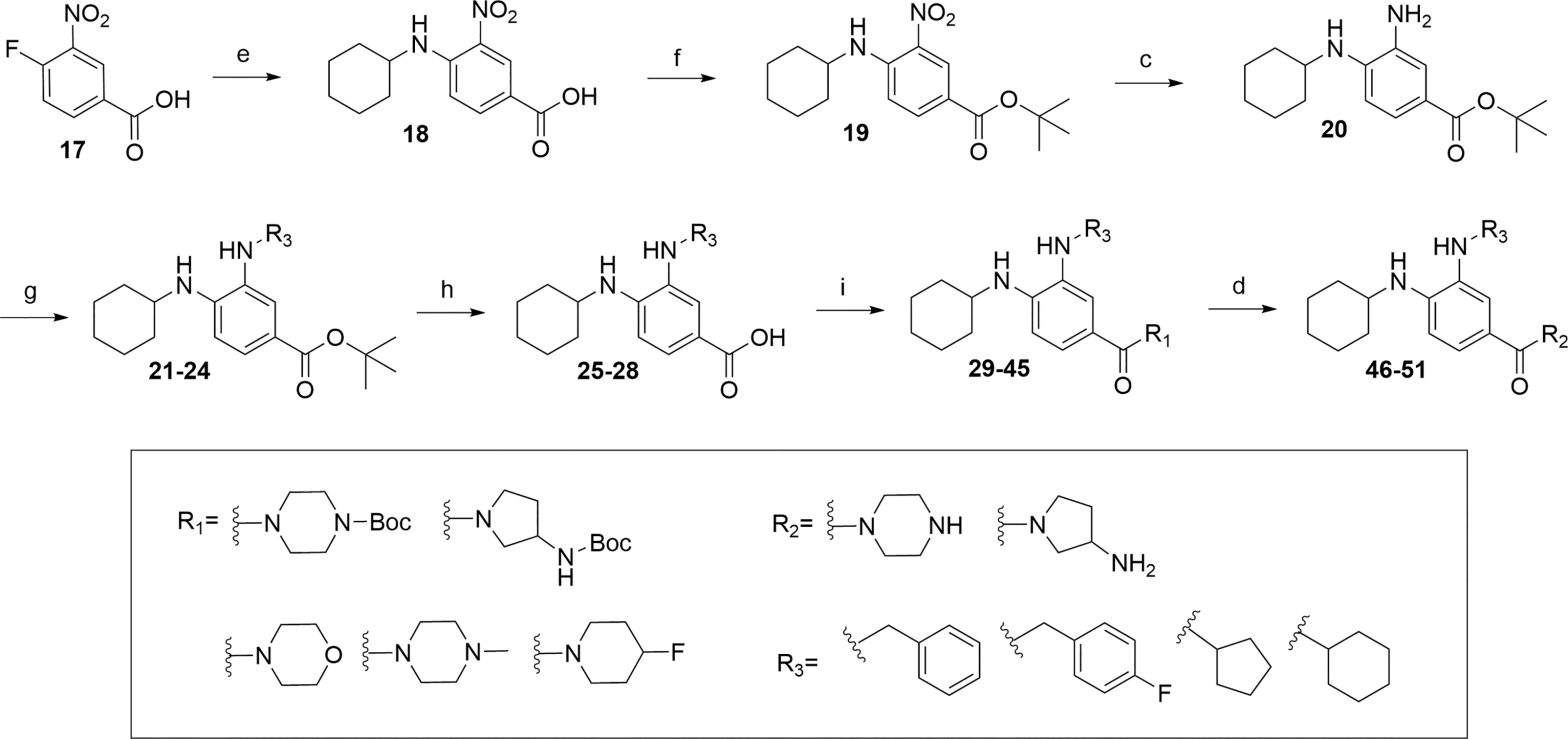
Synthetic Route for the Synthesis of Benzamides with Substituents on Both Aniline Nitrogens (**35**-**51**) Reaction conditions: (e) cyclohexylamine, DIPEA, ACN, 80 °C, 16 h (crude); (f) (Boc)_2_O, DIPEA, DMAP, THF, 60 °C, 72–96 h (96%); (c) 20% Pd(OH)_2_/C, H_2_, MeOH, rt, 16 h (36%); (g) benzaldehyde or proper ketone, AcOH, NaCNBH_4_, dry THF, rt, 16 h (30–91%); (h) TFA, DCM, rt, 16 h (88–97%); (i) proper amine, DIPEA, HATU, THF, 0 °C to rt, 16 h (16–44%); (d) HCl in dioxane 4 M, DCM, rt, 2 h (46–94%).

**Scheme 3:**
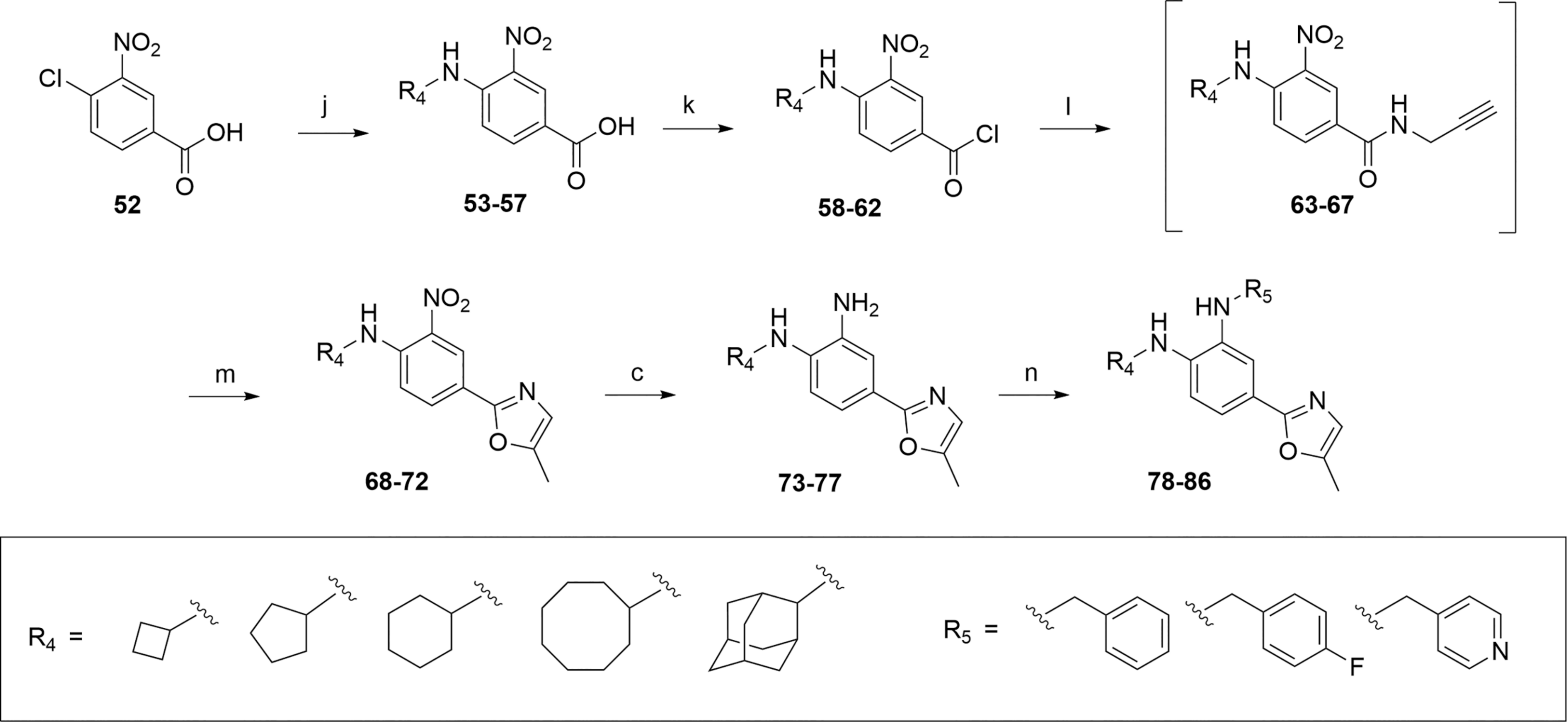
Synthetic Route for the Synthesis of 5-Methyl-oxazol-2-yl Series (**73**-**86**) Reaction conditions: (j) respective cylcloalkylamine, DIPEA, ACN, 90 °C, 5–6 days (40–96%); (k) SOCl_2_, 0 to 90 °C, 2 h (crude); (l) prop-2-yn-1-amine, TEA, DCM, 2.5 - 3.5 h, 0 °C to rt (crude); (m) FeCl_3_, 1,2-DCE, 80 °C, 16 h (16–60%); (c) 20% Pd(OH)_2_/C, H_2_ gas, MeOH, rt, 16 h (56–94%); (n) benzyl or pyridinyl bromide, DIPEA, DCM, 40 °C – 60 °C, 6.5 – 24 h (18–46%).

**Scheme 4:**
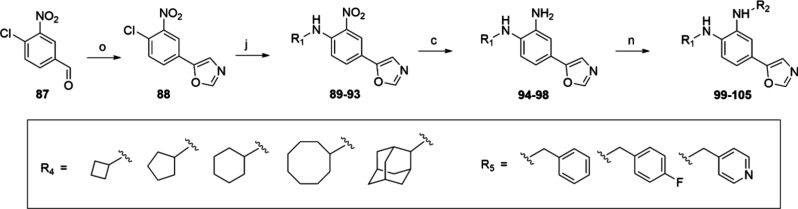
Synthetic Route for the Synthesis of Oxazol-5-yl series (**94**-**105**) Reaction conditions: (o) TosMIC, K_2_CO_3_, MeOH, 40 to 80 °C, 1 h (62%); (j) respective cylcloalkylamine, DIPEA, ACN, 90 °C, 5 days (81–97%); (c) 20% Pd(OH)_2_/C, H_2_ gas, MeOH, rt, 16 h; (9–50%) (n) benzyl/pyridinyl bromide, DIPEA, DCM, 50 °C, 16 h (4–47%).

As described in Schemes 1–4, in total 48 radical-trapping antioxidants were designed and synthesized. The benzamide series, summarized in Table 1, covers analogs with heterocyclic rings such as morpholine (**13, 35**-**38**), *N*-methylpiperazine (**14, 39**-**42**), 4-fluoropiperidine (**15, 43**-**45**), piperazine (**16, 46**-**49**), and pyrrolidin-3-amine (**50-51**) directly involved in amide bond formation. The lipophilic cyclohexyl anchor present in both Fer-1 (**1**) and UAMC-3203 (**2**) scaffolds were retained, and we further diversified the substituents on the other aniline nitrogen by replacing previously used benzyl and 4-fluorobenzyl moieties (still kept as references) with cyclohexyl and cyclopentyl to further modulate metabolic stability. Based on the 5-methyl-oxazol-2-yl scaffold (also referred to as methyl oxazole) compounds **73**-**86** are reported in Table 2 while the series of oxazol-5-yl (also referred to as oxazole) **94**-**105** is reported in Table 3. To modulate lipophilicity we replaced the cyclohexyl of UAMC-3203 by smaller rings (cyclobutyl **73, 78, 94** and **99** cyclopentyl **74, 79, 95** and **100**) and bigger rings like cycloctyl (**76, 81, 97** and **102**) and adamantyl (**77, 82, 98** and **103**) and compared these with the corresponding cyclohexyl analogs (**75, 80, 96** and **101**). Moreover, previous structure–activity relationship (SAR) studies suggested the possibility to modulate the metabolic stability of our RTAs by introducing a substituted aryl ring. Therefore, we synthesized analogs with a 4-fluorobenzyl moiety (**83, 84** and **104**) or a pyridinyl ring (**85, 86** and **105**).

**Table 1:**
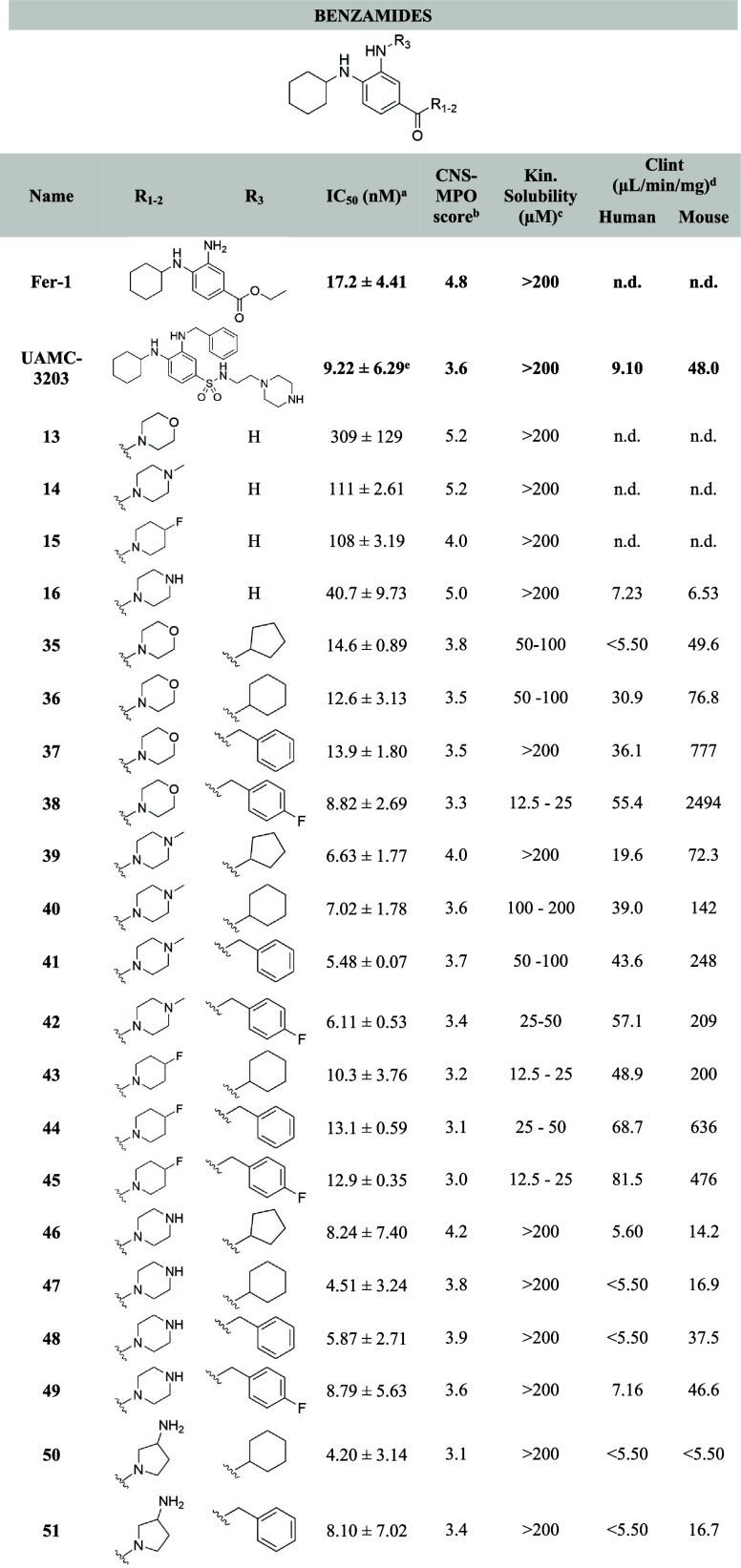
Structures of the Novel Benzamide Analogues Based on Fer-1 and UAMC-3203 Scaffolds with their Corresponding IC_50_, Kinetic Solubility, Calculated CNS-MPO Scores, and Microsomal Stability^f^

a IC_50_ values are calculated using a sigmoidal dose–response curve (see Experimental Section). The mean values are calculated from duplicate measurements (*n* = 2) in HT-1080 cells reported with their corresponding standard deviations.

b CNS-MPO score = central nervous system multiparameter optimization.

c Kinetic solubility in PBS buffer pH 7.4.

d Cl_int_ = intrinsic clearance in human or mouse liver microsomes, verapamil and dextrometorphan were used as controls for human microsomes, diazepam and diphenhydramine were used as controls for mouse liver (see Experimental Section and Supporting Information Tables S2 and S3).

e IC_50_ of UAMC-3203 correspond to the mean value of six independent experiments (*n* = 6).

f n.d. = not determined.

**Table 2:**
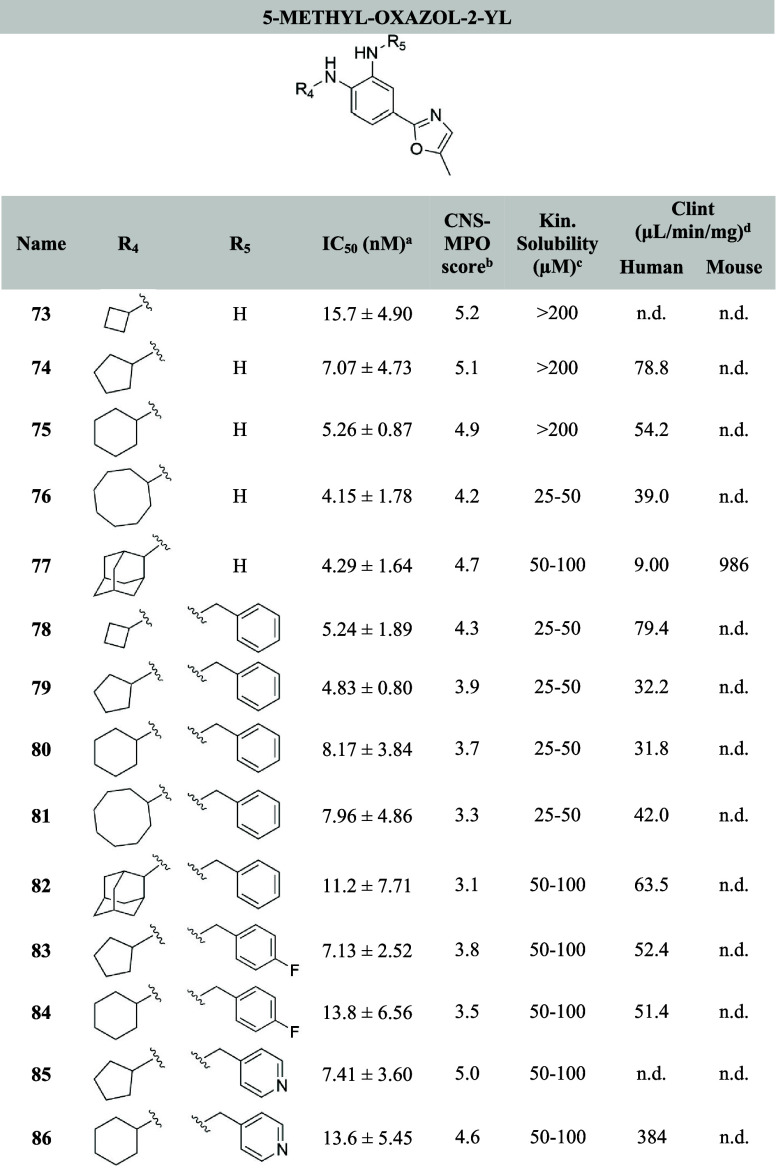
Structures of the Novel 5-Methyl-oxazol-2-yl Analogues with Their Corresponding IC_50_, Kinetic Solubility, Calculated CNS-MPO Scores, and Microsomal Stability^e^

a IC_50_ values are calculated using a sigmoidal dose–response curve (see Experimental Section). The mean values are calculated from duplicate measurements (*n* = 2) in HT-1080 cells reported with their corresponding standard deviations.

b CNS-MPO score = central nervous system multiparameter optimization.

c Kinetic solubility in PBS buffer pH 7.4.

d Cl_int_ = intrinsic clearance in human or mouse liver microsomes, verapamil and dextrometorphan were used as controls for human microsomes, diazepam and diphenhydramine were used as controls for mouse liver (see Experimental Section and Supporting Information).

e n.d. = not determined.

**Table 3:**
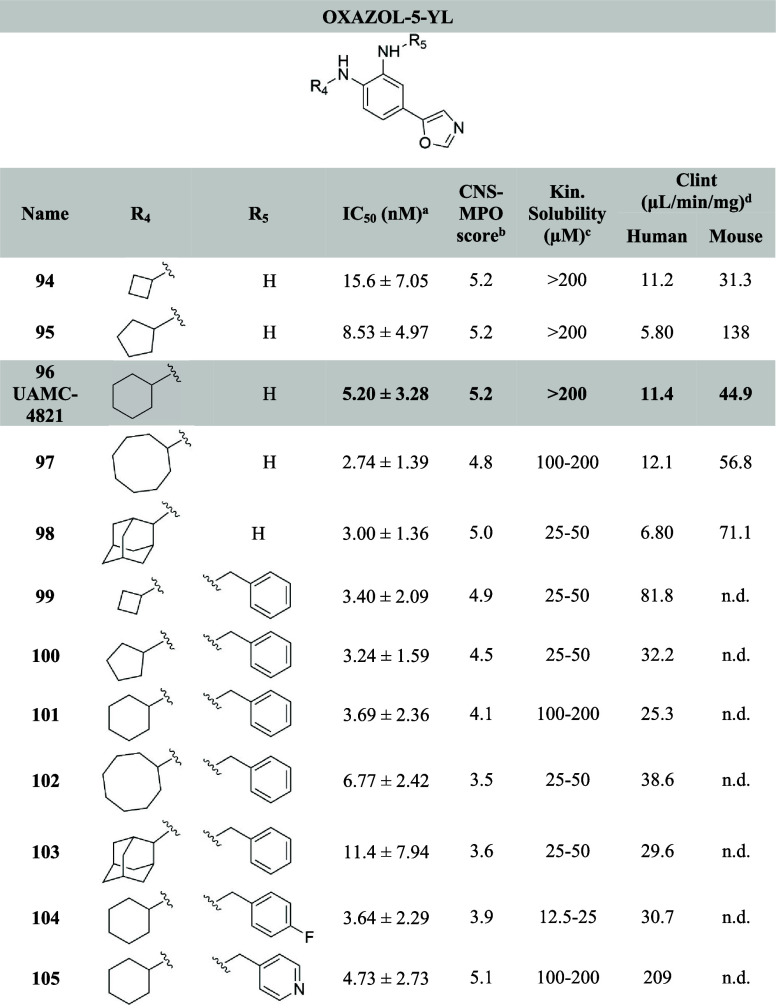
Structures of the Novel Oxazol-5-yl Analogues with Their Corresponding IC_50_, Kinetic Solubility, Calculated CNS-MPO Scores, and Microsomal Stability^e^

a IC_50_ values are calculated using a sigmoidal dose–response curve (see Experimental Section). The mean values are calculated from duplicate measurements (*n* = 2) in HT-1080 cells reported with their corresponding standard deviations.

b CNS-MPO score = central nervous system multiparameter optimization.

c Kinetic solubility in PBS buffer pH 7.4.

d Cl_int_ = intrinsic clearance in human or mouse liver microsomes, verapamil and dextrometorphan were used as controls for human microsomes, diazepam and diphenhydramine were used as controls for mouse liver (see Experimental Section and Supporting Information).

e n.d. = not determined.

### Chemistry

The synthesis of the benzamide series is depicted in Scheme 1 and Scheme 2. To obtain analogs **13**-**16** with the free -NH_2_ group, we applied a previously described synthetic route starting from the amide coupling of commercially available 4-chloro-3-nitrobenzoyl chloride (**3**) with respective amines, followed by nucleophilic aromatic substitution, palladium-catalyzed hydrogenation of the nitro group, and finally Boc deprotection, if required.^37^

For convenience in synthesizing the remaining compounds depicted in Scheme 2, we decided to reverse the order of synthetic steps. The new synthetic route begins with the nucleophilic aromatic substitution of commercially available 4-fluoro-3-nitrobenzoic acid (**17**) with cyclohexylamine. The resulting 4-(cyclohexylamino)-3-nitrobenzoic acid (**18**) was then converted to the corresponding *tert-*butyl ester (**19**). This was followed by palladium-catalyzed hydrogenation of the nitro group and a reductive amination step with the respective aldehyde or ketone to obtain common intermediates **25**-**28**. This approach allowed us to generate common intermediates, such as 4-cyclohexylaminobenzoic acids derivatized at the C_3_ position (**25**-**28**), which could then be further coupled with respective amines to obtain compounds **29**-**45**. If needed, Boc deprotection was subsequently performed to obtain compounds **46**-**51**.

The synthesis of the 5-methyl-oxazol-2-yl series is reported in Scheme 3. In the first step, the commercially available 4-chloro-3-nitrobenzoic acid **52** was substituted with the corresponding cyloalkylamines to obtain the intermediates **53**-**57**. The carboxylic acid analogs **53**-**57** were converted in their corresponding acyl chlorides **58**-**62** before the 5-methyl-oxazol-2-yl formation via the two-step ferric chloride mediated cyclization. Briefly, the crude alkynyl amides intermediates **63**-**67** were first formed by reacting the crude intermediates **58**-**62** with prop-2-yn-1-amine in presence of triethylamine. Then, the crude compounds reacted with ferric chloride to promote cyclization to form the desired 5-methyl-oxazol-2-yl scaffolds **68**-**72.** The intermediates **73**-**77** were synthesized via palladium-catalyzed hydrogenation of the 3-nitro group of **68**-**72**. Finally, the corresponding benzyl, 4-fluorobenzyl and pyridinyl analogs **78**-**86** were formed via nucleophilic substitution.

The synthesis of the oxazol-5-yl series follows the Van Leusen procedure. The commercially available starting material 4-chloro-3-nitrobenzaldehyde **87** was converted into a 5-substituted oxazole derivative **88** upon reaction with *p*-tosylmethyl isocyanide (TosMIC). The rest of the steps including nucleophilic aromatic substitution (**89–93**), palladium-catalyzed hydrogenation (**94**-**98**) and nucleophilic substitution (**99**-**105**) were analogs to the 5-methyl-oxazol-2-yl series.

### Discussion

The antiferroptotic activity of compounds **13**-**16**, **35**-**51**, **73**-**86** and **94**-**105** was tested in fibrosarcoma HT-1080 cells using ML162 to induce ferroptosis. The HT-1080 cell line demonstrated high sensitivity to different ferroptosis triggers and therefore is extensively used to study ferroptosis. The inhibitory activity was compared with the benchmark Fer-1 (IC_50_ = 17.2 nM) and our lead compound UAMC-3203 (IC_50_ = 9.22 nM). Kinetic solubility in PBS buffer (pH 7.4) and microsomal stability for interesting potent compounds were also evaluated and reported in Tables 1–3. CNS-MPO scores were calculated as a predictive parameter of BBB permeability.^45^

#### Ferroptosis Inhibition

The majority of the compounds, except benzamide analogs with unsubstituted NH_2_ (**13**-**16**), demonstrated to be very potent ferroptosis inhibitors, equipotent or more potent than the benchmark Fer-1 and our in house benchmark UAMC-3203.^37^ We found that compounds with a free NH_2_ group (**13**-**16**) exhibited the lowest potency (IC_50_ = 40 – 309 nM). Among substituted benzamides, there were no significant differences in activity; however, derivatives with piperazine (**46**-**49**), methylpiperazine (**39**-**42**) and pyrrolidin-3-amine (**50**-**51**) substituents were notably the most active. All these derivatives demonstrated IC_50_ values below 10 nM. As expected, in terms of activity no remarkable differences were observed between the two series of oxazole analogs. In both series, compounds featuring an unsubstituted NH_2_ and cyclobutyl lipophilic anchor (**73** and **94**) are the least active compounds (IC_50_ = 15 nM for both). Compounds **76** (IC_50_ = 4.15 nM) and **97** (IC_50_ = 2.74 nM), featuring an unsubstituted NH_2_ and a cyclooctyl are the most active analogs for both series in terms of potency. Similarly, compound **98** has an IC_50_ = 3.00 nM. In general, compounds with an unsubstituted NH_2_ and an increasing number of carbons in the cycloalkyl moiety (cyclooctyl **76** and **97** and 2-adamantyl **77** and **98**) lead to improved activity. An opposite trend was observed for benzylated analogs: the smaller the cycloalkyl ring (cyclobutyl **78** and **99**, and cyclopentyl **79** and **100** for both series and analog **101** with the cyclohexyl moiety for the oxazole) the better the potency is (**99** has an IC_50_ = 3.40 nM and **100** has an IC_50_ = 3.24 nM). The introduction of heteroatoms on the benzyl ring (**83**-**86** and **104**-**105**) did not lead to a significant improvement in the activity. Among the analogs with cyclohexyl as R_1_ and benzyl as R_2_ comparable to our in-house benchmark UAMC-3203, the oxazole **101** (IC_50_ = 3.69 nM) is slightly more potent than the methyl oxazole **80** (IC_50_ = 8.17 nM). The same trend is visible among the corresponding 4-fluorobenzyl analogs **104** and **84**, and the corresponding pyridine-4-ylmethyl analogs **105** and **86**.

#### Lipophilic Radical Trapping Potency

**Figure 2:**
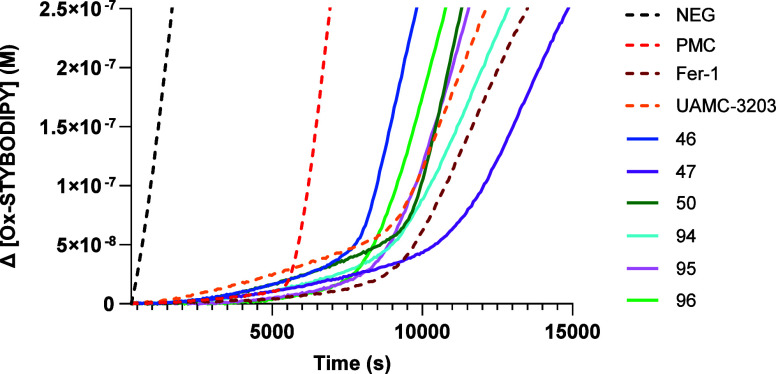
RTA activity of selected compounds (**46, 47, 50, 94**-**96**) at 4 μM was assessed through the FENIX assay in comparison with 2,2,5,7,8-pentamethyl-6-hydroxychromane (PMC), Fer-1 and UAMC-3203. The curves of these reference compounds are depicted with dotted lines. The negative control (uninhibited autoxidation) is represented as a blackdotted line.

Fluorescence-Enabled Inhibited Autoxidation (FENIX) assay was recently developed by Shah et al.^44^ FENIX is a spectrometric method that enables to determine lipophilic radical trapping potency in liposomes which mimics the phospholipid bilayer of the cell membrane. Di-*tert*-undecylhyponitrite (DTUN) is used as a lipophilic radical initiator to promote the autoxidation of lipids. The progress of the oxidation and its inhibition by RTAs can be easily monitored by fluorescence using styrene-conjugated BODIPY (STY-BODIPY) as a fluorogenic probe. FENIX assay is a valuable tool to evaluate the antiferroptotic potency and demonstrate the mechanism of action since its results correlate well with *in vitro* cellular data. Potent RTAs with a good curve profile can significantly delay the autoxidation of lipids. The time of inhibition (*x*-axis) is reported in correlation with the concentration of the oxidized STY-BODIPY fluorescence probe (Figure 2).^30^ The FENIX curves of the selected compounds reported below (**46**, **47**, **50**, **94**-**96**) confirm that the inhibition of ferroptosis occurs due to a lipophilic radical-trapping antioxidant mechanism. However, a strong correlation between the IC_50_ value of ferroptosis inhibition and the log k_inh_ of the FENIX assay was not observed. Nevertheless, all the potent compounds (IC_50_ between 2 and 20 nM) showed a strong delay in autoxidation (log k_inh_ between 4.1 and 5.8), whereas the less potent compounds **13**-**16** (IC_50_ > 40 nM) were weaker lipophilic antioxidants (log k_inh_ < 3.8).

The curves for all the tested analogs, calculated inhibition rate constants (K_inh_) and the corresponding stoichiometry (*n*) values for all the synthesized compounds, are reported in the Supporting Information (Table S1 and Figures S2–S4).

#### Solubility

The kinetic solubility in PBS at pH 7.4 of the new series of compounds was also assessed similarly to our previously published sulfonamide series.^37^ For the benzamide series, the most soluble analogs, with solubility greater than 200 μM, are those with an unsubstituted NH_2_ group (**13**-**16**) and compounds with a basic nitrogen in the substituent attached to the amide (**46**-**51**). For the remaining benzamides, an increase in lipophilicity decreased solubility, with the exceptions of compounds **37** and **39**. The introduction of a substituent on the NH_2_ in the oxazole series is detrimental to the solubility. The analogs featuring an unsubstituted NH_2_ and small cycloalkyl moieties (cyclobutyl, cyclopentyl and cyclohexyl) retain a high solubility (>200 μM) similar to Fer-1 and UAMC-3203 (Tables 1–3).

#### Microsomal Stability

For most of the compounds we determined stability in human microsomes (Tables 1–3). Under the assay conditions in human microsomes compounds with a Cl_int_ < 8.60 mL/min*mg have a low clearance, whereas compounds with a Cl_int_ > 47.0 mL/min*mg show high clearance. Among the benzamides, compounds **16** and **46**-**51**, which contain piperazine and pyrrolidin-3-amine groups, exhibited low clearance. Additionally, among the other benzamides, only compound **35**, which features morpholine and cyclopentyl moieties, demonstrated low clearance. In the oxazole series, compounds **95** and **98** are the only ones with low clearance in human microsomes, followed by those without a benzylic group (**94**, **96**-**97**) and methyl-oxazole **77**, which exhibited medium clearance at the lower end of the range.^52^ Selected compounds, based on their favorable properties, were also tested for stability in mouse microsomes. Under the assay conditions, compounds with a Cl_int_ < 13.1 mL/min/mg are considered to have low clearance, whereas compounds with a Cl_int_ > 71.1 mL/min/mg exhibit high clearance.^52^ While UAMC-3203 showed medium clearance (Cl_int_ = 48.0 mL/min/mg), the aforementioned benzamides demonstrated either low or comparable clearance to UAMC-3203. Additionally, two oxazoles, **94** and **96**, with cyclobutyl and cyclohexyl substituents, respectively, also fell within the medium clearance range. Surprisingly, the corresponding analog with a cyclopentyl ring, **95**, was metabolized much faster in mouse microsomes.

#### MDCK-MDR1 Permeability Assay

The MDCK-MDR1 assay employs the Madin-Darby Canine Kidney (MDCK) cell line, which is transfected to overexpress human P-glycoprotein (multidrug resistance protein 1, MDR1), to assess whether a compound is a substrate of the P-glycoprotein (P-gp) efflux transporter. The assay is considered one of the well validated *in vitro* experiments to determine whether a compound will be able to penetrate the CNS. Based on the properties determined above, six structurally diverse molecules, including three benzamides (**46, 47, 50**) and three oxazoles (**94**-**96**), were selected for the MDCK-MDR1. The results shown in Table 4 indicate that all oxazoles are superior in terms of permeability, achieving high mean P_app_ values for apical-to-basolateral transport without being substrates for P-gp efflux.

**Table 4:** In Vitro Permeability and Efflux Ratios of Selected Compounds Determined in MDCK-MDR1 Assay^a^

	**MDCK permeability dynamic**		
**compound**	**A2B mean*P***_**app**_**± SD(10**^**–6**^**cm/s)**	**B2A mean*P***_**app**_**± SD(10**^**–6**^**cm/s)**	**efflux ratio**
**46**	0.709 ± 0.021	60.60 ± 2.430	85.4
**47**	0.683 ± 0.029	64.00 ± 1.480	93.8
**50**	0.569 ± 0.009	62.90 ± 2.130	111
**94**	62.50 ± 1.800	70.10 ± 22.60	1.12
**95**	51.10 ± 0.111	53.90 ± 0.334	1.05
**96 (UAMC-4821)**	44.10 ± 1.260	41.30 ± 0.186	0.937

a *P*_app_ – the apparent permeability coefficient; A2B – transport of the compound from the apical compartment to the basolateral compartment; B2A - transport of the compound from the basolateral compartment to the apical compartment. A high efflux ratio indicates P-gp mediated drug efflux.

#### Pharmacokinetic (PK) Study

**Figure 3:**
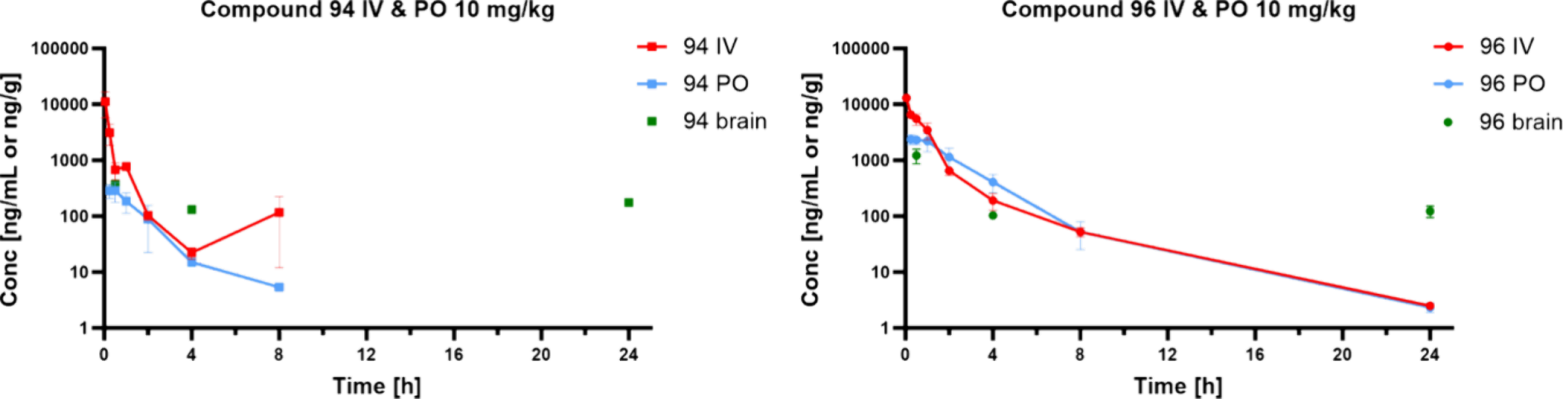
In vivo pharmacokinetic profiles of **94** and **96** – concentrations in plasma after IV and PO administration 10 mg/kg and in brain tissue after IV administration at selected time points. Data is expressed as the mean (*n* = 3 for all time points, except *n* = 6 for IV at 0.05 h, *n* = 1 for **94**, PO at 8 h). Compound **94** was below the detection limit at the last time points after both IV and PO administration. Mean values ± SD in plasma and different organs are reported in the Supporting Information in Table S6 and Figure S6.

Compounds **94** and **96**, were selected based on *in vitro* permeability in the MDCK-MDR1 assay, as well as other evaluated parameters, including microsomal stability, for further determination of their pharmacokinetic profiles *in vivo* (Table 5). PK profiles following intravenous (IV) and oral administration (PO) at 10 mg/kg to male CD-1 mice were determined in comparison with UAMC-3203. Following a single IV dose, plasma samples were collected up to 24 h at eight time points whereas following a single PO dose, plasma samples were collected via serial sampling at seven time points up to 24 h (Figure 3). Following IV dosing (10 mg/kg), **94** is quantifiable in plasma up to 8 h, characterized by a moderate clearance in plasma (54% liver blood flow (LBF), assuming a mouse LBF of 90 mL/min/kg) and a moderate volume of distribution (3.01 L/kg). Concentrations in five organs (liver, lung, heart, kidney and brain) were determined at three time point (0.5, 4 and 24 h). The highest concentrations at 0.5 and 4 h were measured in kidney (data reported in the Supporting Information in Table S4), while the highest concentration at 24 h was measured in brain. Following oral dosing of **94** (10 mg/kg), maximum plasma levels (C_max_ = 351 ng/mL) were reached at 0.5 h postdose, with a half-life of 1.05 h and a mean oral bioavailability of 13%.

**Table 5:** In Vivo Pharmacokinetics Profile of **UAMC-3203**, **94**, and **96**^a^

	**IV** (10 mg/kg)			**PO** (10 mg/kg)		
**compound**	**UAMC-3203**	**94**	**96 (UAMC-4821)**	**UAMC-3203**	**94**	**96 (UAMC-4821)**
**t**_**1/2**_ (h)	*0.51*^b^ 4.77^c^	0.11^b^^,^^d^ NC^c^	0.49^b^ 2.95^c^^,^^d^	2.4	1.05 ± 0.5	2.1 ± 0.7
**T**_**max**_ (h)				1.0	0.5	0.5
**C**_**max**_ (ng/mL)				80.0 ± 8.30	351 ± 75.0	2773 ± 343
**C**_**0**_ (ng/mL)	5905	15387	15535			
**AUC**_**0-last**_ (hr*ng/mL)	3670	3401	9417	243 ± 127	445 ± 116	5894 ± 1414
**AUC**_**0-inf**_ (hr*ng/mL)	3677	3420	9419	238	460 ± 117	5923 ± 1381
**F** %				7 ± 3	13 ± 3.4	63 ± 15
**CL** (mL/min/kg)	45.3	48.7	17.7			
**V**_**ss**_ (L/kg)	8.59	3.01	1.41			

a Data are expressed as the mean ± SD (*n* = 3);

b αt_1/2_ - initial phase.

c βt_1/2_ - terminal phase.

d Predominant; NC = not calculated, r^2^ < 0.9; late T_max_ (insufficient data points post C_max_). Noncompartmental analysis of PK data.

Overall, **96** has a better pharmacokinetic profile. Following IV dosing (10 mg/kg), it is quantifiable in plasma up to 24 h, characterized by a low clearance in plasma (20% LBF) and a moderate volume of distribution (1.41 L/kg). The predominant initial phase half-life is 0.49 h, and the terminal phase half-life is 2.95 h. The highest concentrations at 0.5 and 4 h were measured in the liver and kidney (data reported in the Supporting Information in Table S6 and Figure S6), while the highest concentration at 24 h was measured in the brain. Following oral dosing of **96** (10 mg/kg), maximum plasma levels (C_max_ = 2773 ng/mL) were reached at 0.5 h postdose, with a half-life of 2.1 h and a mean oral bioavailability of 63%.

Concludingly, **96** has lower clearance, and a longer half-life compared to **94**. After oral dosing **96** has an 8-fold higher C_max_ and a 13-fold higher AUC compared to **94**, resulting in a high oral bioavailability of **96**. Moreover, **96** demonstrated lower clearance (CL = 17.7 mL/min/kg), and significantly improved oral bioavailability (F = 63%) compared to UAMC-3203 (CL = 45.3 mL/min/kg and F = 7%).

Taking into account the high oral bioavailability and the high concentration in brain after 24 h we consider compound **96** as the best candidate to evaluate ferroptosis inhibition in disease animal models of the central nervous system.

## Conclusions

The interest in developing novel compounds with potent antiferroptotic activity, oral bioavailability and good blood-brain barrier permeability is an emerging area of research to further evaluate the role of ferroptosis in different neurodegenerative diseases, potentially resulting in novel lead compounds for drug discovery.^53^ Since the discovery of ferroptosis and Fer-1, several compounds have been reported as potent ferroptosis inhibitors. After 12 years, despite the discovery of diverse biochemical pathways involved in ferroptosis, RTAs are one of the most explored and the most potent classes of ferroptosis inhibitors. In 2018, we reported a Fer-1 analog, UAMC-3203 which demonstrated to be an excellent candidate to protect against ferroptosis-driven diseases, *in vitro* and *in vivo*. UAMC-3203 however has limited oral bioavailability and limited exposure in the CNS. Therefore, we designed and synthesized a novel library of lipophilic RTAs. We hypothesized that decreasing the size of the molecule, reducing its basicity and reducing the number of hydrogen bond donors (HBD) would induce favorable pharmacokinetic properties. We modulated the lipophilicity with various cycloalkyl substituents, and we introduced two cyclic amide bioisosters, 5-methyl-oxazol-2-yl and oxazol-5-yl. This resulted in a series of highly potent ferroptosis inhibitors with a lipophilic radical trapping mechanism of action. Furthermore, oxazoles with an unsubstituted NH_2_ on the aromatic ring showed high solubility and low clearance in human microsomes.

Compound **96** (**UAMC-4821**) emerged as the best compound of the series in terms of activity, CNS-MPO score, FENIX assay results, solubility, human microsomal stability, BBB permeability and its PK profile after IV and oral administration. Compound **96** shows ferroptosis inhibition in the same range as UAMC-3203 (IC_50_ = 5.20 nM vs IC_50_ = 9.22 nM for UAMC-3203). It has low clearance in human microsomes, and despite showing moderate clearance in mouse microsomes, **96** has low clearance *in vivo* in mice. After 24 h **96** still showed high concentrations in brain, and combined with a favorable oral bioavailability of 63%, these are highly interesting properties with potential for chronic administration in the treatement of neurodegenerative diseases. In the near future, we will further explore **96***in vivo* in ferroptosis based CNS animal models.

## Experimental Section

### General Information

Unless otherwise stated, laboratory reagent grade solvents were used. Reagents were obtained from various commercial sources and were used without any prior purification. Characterization of all compounds was done with ^1^H and ^13^C NMR and mass spectrometry. NMR spectra were recorded with a 400 MHz Bruker Avance III Nanobay spectrometer with Ultrashield. All obtained spectra were analyzed using MestReNova analytical chemistry software. Chemical shifts are displayed in ppm and coupling constants are shown in hertz (Hz). ES mass spectra were obtained from an Esquire 3000plus Ion Trap Mass Spectrometer from Bruker Daltonics. The UPLC (ultra performance liquid chromatography), used to quantify the purity of the products, was an ACQUITY UPLC H-Class system with a TUV detector Waters coupled to an MS detector Waters Qda. Waters Acquity UPLC BEH C18 1.7 μm, 2.1 mm × 50 mm column was used. The eluent was composed of two different solvents. Solvent C consisted of water with 0.1% formic acid, solvent D was acetonitrile. For most of the experiments, unless stated otherwise, the following general method was used. The column was first equilibrated for 0.15 min with a mixture of 95% solvent C and 5% solvent D. After that, solvent D was increased linearly to 100% over 1.75 min before being held constant for 0.25 min, followed by a mixture of 95% solvent C and 5% solvent D for 0.75 min (flow rate 0.7 mL/min). All mass spectra were recorded over a *m*/*z* range of 100–1000. The wavelength for UV detection was 254 nm. Method II starts with equilibration of column for 0.15 min with a mixture of 95% solvent C and 5% solvent D. After that, solvent D was increased linearly to 100% over 2.50 min before being held constant for 0.75 min, followed by a mixture of 95% solvent C and 5% solvent D for 0.75 min (flow rate 0.7 mL/min). Selected compounds of the invention were analyzed by high resolution mass spectrometry: 10 μL of each sample (concentration = 10^–5^ M) was injected using the CapLC system (Waters, Manchester, UK) and electrosprayed using a standard electrospray source. Samples were injected with an interval of 5 min. Positive ion mode accurate mass spectra were acquired using a Q-TOF II instrument (Waters, Man-chester, UK). The MS was calibrated prior to use with a 0.2% H_3_PO_4_ solution. The spectra were lock mass corrected using the known mass of the nearest H_3_PO_4_ cluster. During the chemical synthesis, flash purification was performed when necessary, on a Biotage ISOLERA One flash system equipped with an internal variable dual wavelength diode array detector (200–400 nm). Reverse phase purifications were done using Buchi EcoFlex C18 cartridges, dry sample loading was done by self-packing sample cartridges using Celite 545. Gradients used varied for each purification. Several synthesis procedures that were used in the preparation of intermediates and final products are summarized here as “General Procedures”. All reactions were performed under argon unless otherwise stated. All target compound are more than 95% pure by UPLC analysis.

#### General Procedure A: Nucleophilic Substitution (**2**-**5**):

A stirred solution of 4-chloro-3-nitrobenzoyl chloride **3** (1.0 eq) in DCM (20 mL), pyridine (2.0 eq) and the respective amine (1.0 eq) were added and the reaction was stirred at room temperature for 16 h. After completion, the reaction mixture was diluted with EtOAc (10 mL) and the organic layers were washed with water (3 × 10 mL) and 1N HCl, dried using anhydrous sodium sulfate before being concentrated in vacuo. The crude compounds **2–5** were used without any further purification for the next step.

#### General Procedure B: Nucleophilic AromaticSsubstitution (**8**-**11**)

To a solution of compound **4–7** (1.0 eq) in ACN (40 mL), cyclohexylamine (3.0 eq) and DIPEA (3.0 eq) were added. The reaction was stirred for 72–96 h at 90 °C. After completion, the mixture was cooled down to room temperature and extracted with EtOAc (3 × 10 mL). The collected organic layers were washed with water (20 mL) then dried using anhydrous sodium sulfate and concentrated in vacuo. The residue was purified by reversed-phase flash column chromatography (FCC) (water + 0.1% formic acid/ACN) to obtain compounds **8–11.**

#### General Procedure C: Catalytic Hydrogenation (**12-15**, **20**, **73-77**, and **94-98**)

Compounds **8-11** (1.0 eq Scheme 1) or **19** (1.0 eq Scheme 2) or **68-72** (1.0 eq Scheme 3) or **89–93** (1.0 eq Scheme 4) were dissolved in MeOH (60 mL) and the solution was purged with argon. Palladium hydroxide on carbon (20% Pd(OH)_2_/C, 50% of water, 0.1 eq) was added under inert atmosphere while continuously stirring. Next, the reaction mixture was put under hydrogen atmosphere and stirred for 12–72 h at room temperature. After completion of the reaction, the solution was filtered through a patch of Celite and the filtrate was concentrated under reduced pressure. The crude compound was purified by reversed-phase FCC (water/MeOH) to obtain compound **12–15, 20, 73–77** and **94–98**.

#### General Procedure D: Boc Deprotection (**16** and **46-51**):

Compounds **12** (1.0 eq Scheme 1) or **29, 32, 36, 37, 41, 42** (1.0 eq Scheme 2) were dissolved in DCM (10 mL) followed by the addition of a 4 M solution of HCl in 1,4-dioxane (15.0 eq) The reaction mixture was stirred at room temperature for 2 h. After completion of the reaction, diethyl ether was added to the reaction mixture in order to precipitate compounds **16** and **46– 51**. The obtained HCl salts were subsequently washed with a minimal amount of diethyl ether.

#### Procedure E: Nucleophilic Aromatic Substitution (4-(Cyclohexylamino)-3-nitrobenzoic acid (**18**))

To a stirred solution of compounds 4-fluoro-3-nitrobenzoic acid **17** (2.250 g, 12.15 mmol) in can (60 mL), DIPEA (6.35 mL, 36.5 mmol) was added, followed by the addition of cyclohexylamine (4,17 mL, 36.5 mmol). The reaction mixture was stirred for 72–96 h at room temperature. After completion of the reaction, the yellow precipitate was filtered, washed with water and dried under vacuum, providing compounds 4-(cyclohexylamino)-3-nitrobenzoic acid **18** (3.210 g, 12.15 mmol, 100% yield) which was used for the next step without further purification. ^1^H NMR (400 MHz, DMSO-*d*_*6*_) δ 0.99 (t, *J* = 7.22 Hz, 2H), 1.22 - 1.31 (m, 1H), 1.38 - 1.48 (m, 2H), 1.57 - 1.64 (m, 1H), 1.68 - 1.76 (m, 1H), 1.92 - 1.99 (m, 2H), 2.77 (q, *J* = 7.23 Hz, 1H), 3.68 - 3.77 (m, 1H), 7.32 (d, *J* = 9.26 Hz, 1H), 7.50 - 7.59 (m, 1H), 7.78 (ddd, *J* = 0.70, 2.26, 9.17 Hz, 1H), 8.28 (d, *J* = 7.79 Hz, 1H), 8.43 (d, *J* = 2.25 Hz, 1H); ^13^C NMR (101 MHz, DMSO-*d*_*6*_) δ 15.13, 24.47, 25.42, 32.23, 37.96, 51.16, 116.50, 126.53, 130.02, 133.79, 146.32; MS (ESI) *m*/*z* = 265 [M+H]^+^.

#### Procedure F: Esterification (*tert*-Butyl 4-(cyclohexylamino)-3-nitrobenzoate (**19**))

To a stirred solution of 4-(cyclohexylamino)-3-nitrobenzoic acid **18** (3.210 g, 12.15 mmol) in THF (60 mL), DIPEA (8.46 mL, 48.6 mmol) and DMAP (1.558, 12.75 mmol) were added followed by the addition of di-*t*-butyldicarbonate (10.60 g, 48.60 mmol). The reaction mixture was refluxed for 72–96 h. Then, the reaction mixture was directly loaded on Celite and the crude compound was purified by FCC (hep/EtOAc) to yield *tert*-butyl 4-(cyclohexylamino)-3-nitrobenzoate **19** (3.750 g, 11.70 mmol, 96% yield).^1^H NMR (400 MHz, CDCl_3_) δ 1.03 – 1.19 (m, 3H), 1.26 – 1.35 (m, 2H), 1.58 (s, 9H), 1.81 (dd, *J* = 4.4, 9.1 Hz, 1H), 1.91 (dd, *J* = 4.0, 12.2 Hz, 2H), 2.06 (d, *J* = 9.9 Hz, 2H), 3.51 – 3.61 (m, 1H), 6.86 (d, *J* = 9.1 Hz, 1H), 7.97 (ddd, *J* = 0.7, 2.1, 9.2 Hz, 1H), 8.38 (d, *J* = 7.5 Hz, 1H), 8.79 (d, *J* = 2.1 Hz, 1H); ^13^C NMR (101 MHz, CDCl_3_) δ 25.05, 25.62, 28.40, 32.71, 51.47, 81.31, 113.80, 118.78, 129.65, 131.09, 136.32, 146.82, 164.59; MS (ESI) *m*/*z* = 321 [M+H]^+^.

#### General Procedure G: Reductive Amination (**21-24**)

Compound **20** (1.0 eq) was dissolved in THF(20 mL), followed by the addition of acetic acid (23.0 eq) and the corresponding aldehyde or ketone (3.0 eq). After 15 min, sodium cyanoborohydride (2.5 eq) was added and the reaction mixture was stirred at room temperature for 12–16 h. After completion, the reaction was quenched with water and the product was extracted with DCM (2 × 10 mL). The combined organic layers were dried over sodium sulfate, filtered and loaded on Celite. The crude compounds were purified by FCC (hep/EtOAc) to yield products **21–24**.

#### General Procedure H: *t*-Bu Ester Hydrolysis (**25-28**)

To a stirred solution of compounds **21–24** (1.0 eq.) in DCM (15 mL), TFA (10 eq.) was added, and the reaction mixture was stirred 16 h at room temperature. After completion, acetonitrile was added to the reaction mixture in order to precipitate compounds **25–28** obtained as TFA salts.

#### General Procedure I: Amide Coupling (**29-45**)

A stirred solution of compound **25–28** (1.0 eq) in THF (10 mL) was cooled to 0 °C in an ice bath, followed by the addition of HATU (1.4 eq), DIPEA (3.0 eq) and the corresponding amine (1.5 eq). The reaction mixture was stirred for 16 h at room temperature. After completion, the reaction mixture was directly loaded on Celite without any additional workup. The crude compound was purified by reversed-phase FCC (water/MeOH) to obtain products **29–45.**

#### General Procedure J: Nucleophilic Aromatic Substitution (**53-57** and **89-93**)

To a solution of 4-chloro-3-nitrobenzoic acid **52** (1.0 eq) or intermediate **88** (1.0 eq) in ACN (30 mL) the appropriate cycloalkyl amine (3.5 eq) and *N,N-*Di-iso-propyl ethylamine (3.5 eq) were added. The reaction was stirred for 5 days at 90 °C. After completion, the mixture was cooled down to room temperature and extracted with EtOAc (3 × 10 mL). The collected organic layers were washed with water (20 mL) then dried using anhydrous sodium sulfate and concentrated *in vacuo.* The residue was purified by reversed-phase FCC (water + 0.1% formic acid/ACN) to obtain the intermediates **53–57** and **89–93.**

#### General Procedure K Acyl Chloride Formation (**58-62**)

Compounds **53–57** (1.0 eq) were dissolved in a cooled solution of thionyl chloride (20.0 eq). The reaction mixture was stirred for 15 min. at 0 °C. Then, the temperature was increased to 90 °C and stirred for another 2 h at reflux. After completion, the reaction mixture was concentrated under reduced pressure providing compounds **58–62** The compound crudes were used without any further purification in the next step.

#### General Procedure L-M: Two-Step Regiocontrolled 2-Oxazole Formation (**68-72**)

To a cooled solution of prop-2-yn-1-amine (1.3 eq) in DCM (30 mL), compounds **58–62** (1.0 eq) and TEA 3.0 eq) were added. Then, the reaction mixture was stirred for 1 - 3 h at room temperature. After completion, the reaction mixture was quenched with water (10 mL) and extracted with DCM (3 × 20 mL). The combined organic layers were dried using anhydrous sodium sulfate, and concentrated *in vacuo*, providing the appropriate nitril intermediates **63–67.** Without further purification, the nitril intermediates **63–67** (1.0 eq) were dissolved in 1,2-dichloroethane (30 mL). Ferric chloride (1.0 eq) was added, and the reaction mixture was stirred overnight at 80 °C. After completion, the reaction mixture was quenched with water (10 mL) and extracted with DCM (3 × 20 mL). The organic layers were dried using anhydrous sodium sulfate before being concentrated *in vacuo*. The residue was purified by FCC (hep/EtOAc) to obtain the intermediates **68–72.**

#### General Procedure N: Nucleophilic Aromatic Substitution (**78-86** and **99-105**)

Compounds **73–77** (1.0 eq) or **94–98** (1.0 eq) were dissolved in DCM (20 mL) followed by the addition of *N,N*-Di-*iso*-propylethylamine (1.0 eq) and the corresponding benzyl- or pyridinylderivate (1.0 eq). The mixture was allowed to stir at 35–45 °C for 5 h. The benzyl bromide residue was removed by silica filtration while the crude product was washed with acetone (10 mL) followed by a mixture EtOAc/MeOH (9:1). Finally, the residue was purified by FCC (water/MeOH) to yield final compounds **78–86** and **99–105**.

#### Procedure O: Van Leusen Oxazole Formation (5-(4-Chloro-3-nitrophenyl)oxazole (**88**)

TosMIC (2.640 g, 13.52 mmol) was dissolved in MeOH (60 mL). Potassium carbonate (3.720 g, 26.93 mmol) was added, and the reaction mixture was stirred for 10 min. at 40 °C under reflux conditions. Then, 4-chloro-3-nitrobenzaldehyde **87** (2.510 g, 13.51 mmol) was added and the temperature was increased to 80 °C for 1 h. After completion, the reaction mixture was poured in water and extracted three times with DCM (3 × 10 mL). The combined organic fractions were dried over anhydrous sodium sulfate, filtered and further concentrated under reduced pressure. The residue was purified by FCC (DCM/EtOAc) to obtain compound 5-(4-chloro-3-nitrophenyl)oxazole **88** (2.250 g, 10.02 mmol, 62% yield). ^1^H NMR (400 MHz, DMSO-*d*_6_) δ 7.88 (d, *J* = 8.5 Hz, 1H), 7.94 (s, 1H), 8.01 (dd, *J* = 2.1, 8.5 Hz, 1H), 8.41 (d, *J* = 2.1 Hz, 1H), 8.57 (s, 1H).); MS (ESI) *m*/*z* 225 [M+H]^+^.

#### *tert*-Butyl 4-(4-Chloro-3-nitrobenzoyl)piperazine-1-carboxylate (**4**)

General procedure A was followed using compound **3** (1.000 g, 4.960 mmol) and *tert-*butyl piperazine-1-carboxylate (0.920 g, 4.960 mmol) as the corresponding amine to provide *tert-*butyl 4-(4-chloro-3-nitrobenzoyl)piperazine-1-carboxylate **4** (1.631 g, 4.410 mmol, 97% yield). ^1^H NMR (400 MHz, CDCl_3_) δ 1.49 (s, 9H), 3.24 (t, *J* = 5.2 Hz, 2H), 3.38 – 3.62 (m, 4H), 3.76 (d, *J* = 5.3 Hz, 2H), 7.64 (d, *J* = 3.3 Hz, 1H), 8.23 (dd, *J* = 8.3, 1.9 Hz, 1H), 8.59 (d, *J* = 2.0 Hz, 1H); ^13^C NMR (101 MHz, CDCl_3_) δ 28.35, 42.43, 43.66, 47.75, 80.69, 124.62, 128.83, 131.72, 132.40, 135.16, 147.84, 154.42, 166.93; MS (ESI) *m*/*z* = 314.0 [M-t-bu]^+^.

#### (4-Chloro-3-nitrophenyl)(morpholino)methanone (**5**)

General procedure A was followed using compound **3** (4.000 g, 18.20 mmol) and morpholine (0.518 mL, 6.000 mmol) as the corresponding amine to provide (4-chloro-3-nitrophenyl)(morpholino)methanone **5** (1.600 g, 5.910 mmol, 98% yield). ^1^H NMR (400 MHz, MeOD) δ 3.45 (br. s, 2H), 3.63 – 3.67 (m, 2H), 3.73 – 3.77 (m, 4H), 7.78 (d, *J* = 0.9 Hz, 1H), 8.21 (dd, *J* = 2.0, 8.4 Hz, 1H), 8.48 (d, *J* = 1.9 Hz, 1H); ^13^C NMR (101 MHz, MeOD) δ 43.94, 67.59, 125.58, 127.45, 132.96, 133.33, 134.92, 149.58, 168.87; MS (ESI) *m*/*z* = 271.1 [M+H]^+^.

#### (4-Chloro-3-nitrophenyl)(4-methylpiperazin-1-yl)methanone (**6**)

General procedure A was followed using compound **3** (1.500 g, 7.440 mmol) and 1-methylpiperazine (0.745 g, 7.440 mmol) as the corresponding amine to provide (4-chloro-3-nitrophenyl)(4-methylpiperazin-1-yl)methanone **6** (1.863 g, 6.570 mmol, 96% yield). ^1^H NMR (400 MHz, CDCl_3_) δ 2.48 (s, 3H), 2.60 – 2.80 (m, 4H), 3.76 (d, *J* = 119.0 Hz, 4H), 7.95 (d, *J* = 1.9 Hz, 1H), 8.18 (dd, *J* = 2.0, 8.6 Hz, 1H), 8.52 – 8.57 (m, 1H); ^13^C NMR (101 MHz, MeOD) δ 46.34, 51.19, 53.39; 123.25, 128.74, 129.36, 133.14, 135.64, 147.82, 168.78; MS (ESI) *m*/*z* = 284.1 [M+H]^+^.

#### (4-Chloro-3-nitrophenyl)(4-fluoropiperidin-1-yl)methanone (**7**)

General procedure A was followed using compound **3** (0.200 g, 0.909 mmol) and 4-fluoropiperidine (0.094 g, 0.909 mmol) as the corresponding amine to provide (4-chloro-3-nitrophenyl)(4-fluoropiperidin-1-yl)methanone **7** (0.137 g, 0.478 mmol, 53% yield); ^1^H NMR (400 MHz, CDCl_3_) δ 1.93 (d, *J* = 40.8 Hz, 4H), 3.42 (s, 1H), 3.57 (td, *J* = 3.7, 11.2 Hz, 2H), 4.07 (s, 1H), 4.93 (dtt, *J* = 3.0, 5.5, 47.6 Hz, 1H), 7.56 (dd, *J* = 1.9, 8.2 Hz, 1H), 7.62 (d, *J* = 8.2 Hz, 1H), 7.93 (d, *J* = 1.9 Hz, 1H); ^13^C NMR (101 MHz, CDCl_3_) δ 31.12 (d, *J* = 86.2 Hz), 40.97 (d, *J* = 531.6 Hz), 86.96 (d, *J* = 171.9 Hz), 124.43, 128.59, 131.59, 132.34, 135.47, 147.79, 166.87; MS (ESI) *m*/*z* = 288 [M+H]^+^.

#### *tert*-Butyl 4-(4-(cyclohexylamino)-3-nitrobenzoyl)piperazine-1-carboxylate (**8**)

General procedure B was followed using compound **4** (1.631 g, 4.410 mmol), to provide *tert*-butyl 4-(4-(cyclohexylamino)-3-nitrobenzoyl)piperazine-1-carboxylate **8** (1.730 g, 4.000 mmol, 91% yield). ^1^H NMR (400 MHz, CDCl_3_) δ 1.31 – 1.40 (m, 1H), 1.42 – 1.46 (m, 2H), 1.49 (s, 9H), 1.62 – 1.66 (m, 2H), 1.66 – 1.73 (m, 1H), 1.79 – 1.89 (m, 2H), 2.03 – 2.11 (m, 2H), 3.40 – 3.54 (m, 4H), 3.53 – 3.59 (m, 1H), 3.59 – 3.69 (m, 4H), 6.93 (d, *J* = 9.0 Hz, 1H), 7.56 (dd, *J* = 2.1, 8.9 Hz, 1H), 8.28 – 8.35 (m, 2H); ^13^C NMR (101 MHz, CDCl_3_) δ 24.47, 25.48, 28.38, 32.61, 51.26, 77.23, 80.43, 114.49, 121.11, 127.06, 130.46, 135.52, 145.52, 154.57, 168.95; MS (ESI) *m*/*z* = 377.1 [M+H]^+^.

#### (4-(Cyclohexylamino)-3-nitrophenyl)(morpholino)methanone (**9**)

General procedure B was followed using compound **5** (1.600 g, 5.910 mmol), to provide (4-(cyclohexylamino)-3-nitrophenyl)(morpholino)methanone **9** (1.780 g, 5.340 mmol, 90% yield). ^1^H NMR (400 MHz, MeOD) δ 1.25 – 1.53 (m, 5H), 1.63 – 1.73 (m, 1H), 1.92 – 2.01 (m, 2H), 2.01 – 2.12 (m, 2H), 2.96 – 3.06 (m, 1H), 3.65 (s, 4H), 3.66 – 3.73 (m, 4H), 7.12 (d, *J* = 9.1 Hz, 1H), 7.56 (dd, *J* = 2.1, 9.0 Hz, 1H), 8.27 (d, *J* = 2.2 Hz, 1H); ^13^C NMR (101 MHz, MeOD) δ 25.40, 25.95, 33.59, 51.51, 52.24, 67.78, 115.88, 122.11, 128.06, 132.26, 134.69, 146.74, 170.87; MS (ESI) *m*/*z* 334.2 [M+H]^+^.

#### (4-(Cyclohexylamino)-3-nitrophenyl)(4-methylpiperazin-1-yl)methanone (**10**)

General procedure B was followed using compound **6** (1.863 g, 3.940 mmol), to provide (4-(cyclohexylamino)-3-nitrophenyl)(4-methylpiperazin-1-yl)methanone **10** (1.200 g, 3.460 mmol, 88% yield). ^1^H NMR (400 MHz, CDCl_3_) δ 1.28 – 1.37 (m, 1H), 1.36 – 1.50 (m, 4H), 1.62 – 1.69 (m, 1H), 1.78 – 1.84 (m, 2H), 1.98 – 2.11 (m, 2H), 2.35 (s, 3H), 2.39 – 2.58 (m, 4H), 3.48 – 3.59 (m, 1H), 3.59 – 3.81 (m, 4H), 6.90 (d, *J* = 9.0 Hz, 1H), 7.54 (dd, *J* = 2.1, 8.9 Hz, 1H), 8.28 (d, *J* = 2.2 Hz, 2H); ^13^C NMR (101 MHz, CDCl_3_) δ 24.48, 25.49, 32.62, 45.93, 51.24, 54.90, 77.24, 114.39, 121.44, 126.97, 130.44, 135.58, 145.44, 168.66; MS (ESI) *m*/*z* = 347.1 [M+H]^+^.

#### (4-(Cyclohexylamino)-3-nitrophenyl)(4-fluoropiperidin-1-yl)methanone (**11**)

General procedure B was followed using compound **7** (0.137 g, 0.478 mmol), to provide ((4-(cyclohexylamino)-3-nitrophenyl)(4-fluoropiperidin-1-yl)methanone **11** (0.062 g, 0.177 mmol, 37% yield). ^1^H NMR (400 MHz, CDCl_3_) δ 1.26 – 1.38 (m, 1H), 1.40 – 1.48 (m, 3H), 1.62 (s, 3H), 1.66 – 1.73 (m, 1H), 1.80 – 1.88 (m, 2H), 1.88 – 1.93 (m, 1H), 1.93 – 2.00 (m, 2H), 2.07 (dd, *J* = 10.1, 4.5 Hz, 2H), 3.51 – 3.60 (m, 1H), 3.60 – 3.71 (m, 2H), 3.79 (s, 1H), 4.85 – 5.04 (m, 1H), 6.93 (d, *J* = 9.0 Hz, 1H), 7.57 (dd, *J* = 9.0, 2.1 Hz, 1H), 8.31 (d, *J* = 2.2 Hz, 2H); ^13^C NMR (101 MHz, CDCl_3_) δ 24.48, 25.49, 30.98 – 31.50 (m), 51.25, 77.23, 77.34, 87.58 (d, *J* = 171.5 Hz), 114.43, 121.51, 126.76, 130.44, 135.46, 145.46, 168.85; MS (ESI) *m*/*z* = 350.1 [M+H]^+^.

#### *tert*-Butyl 4-(3-amino-4-(cyclohexylamino)benzoyl)piperazine-1-carboxylate (**12**)

General procedure C was followed using compound **8** (1.730 g, 4.00 mmol), to provide *tert*-butyl 4-(3-amino-4-(cyclohexylamino)benzoyl)piperazine-1-carboxylate **12** (0.934 g, 2.320 mmol, 58% yield). ^1^H NMR (400 MHz, MeOD) δ 1.17 – 1.35 (m, 3H), 1.49 (s, 11H), 1.71 (dt, *J* = 12.8, 3.8 Hz, 1H), 1.82 (dt, *J* = 13.3, 3.8 Hz, 2H), 2.09 (dd, *J* = 12.6, 4.0 Hz, 2H), 3.36 (d, *J* = 3.6 Hz, 1H), 3.48 (s, 4H), 3.56 – 3.70 (m, 4H), 6.62 (d, *J* = 8.7 Hz, 1H), 6.77 – 6.96 (m, 2H); ^13^C NMR (101 MHz, MeOD) δ 24.86, 25.72, 27.20, 32.83, 51.37, 80.22, 109.67, 115.18, 119.80, 122.07, 133.49, 138.54, 154.90, 172.77; MS (ESI) *m*/*z* 403.4 [M+H]^+^.

#### (3-Amino-4-(cyclohexylamino)phenyl)(morpholino)methanone (**13**)

General procedure C was followed using compound **9** (1.760 g, 5.280 mmol), to provide (3-amino-4-(cyclohexylamino)phenyl)(morpholino)methanone **13** (0.911 g, 3.000 mmol, 56% yield). ^1^H NMR (400 MHz, DMSO-*d*_*6*_) δ 1.10 – 1.26 (m, 3H), 1.26 – 1.42 (m, 2H), 1.61 (dt, *J* = 3.6, 12.6 Hz, 1H), 1.73 (dt, *J* = 3.7, 13.1 Hz, 2H), 1.96 (dd, *J* = 3.9, 12.8 Hz, 2H), 3.15 – 3.29 (m, 1H), 3.48 (q, *J* = 4.8 Hz, 4H), 3.56 (q, *J* = 4.8 Hz, 4H), 4.53 (d, *J* = 7.5 Hz, 1H), 4.69 (br. s, 1H), 6.41 (d, *J* = 8.2 Hz, 1H), 6.59 (dd, *J* = 2.0, 8.1 Hz, 1H), 6.65 (d, *J* = 2.0 Hz, 1H); ^13^C NMR (101 MHz, DMSO-*d*_*6*_) δ 24.53, 25.46, 32.54, 50.58, 66.09, 108.23, 113.65, 117.66, 121.87, 134.09, 136.32, 170.27; HRMS: calcd for C_17_H_25_N_3_O_2_ 304.2020, found 304.2028 [M + H]^+^.

#### (3-Amino-4-(cyclohexylamino)phenyl)(4-methylpiperazin-1-yl)methanone (**14**)

General procedure C was followed using compound **10** (1.200 g, 3.460 mmol), to provide (3-amino-4-(cyclohexylamino)phenyl)(4-methylpiperazin-1-yl)methanone **14** (0.394 g, 1.250 mmol, 36% yield); ^1^H NMR (400 MHz, MeOD) δ 1.17 – 1.34 (m, 3H), 1.42 (qt, *J* = 3.3, 12.7 Hz, 2H), 1.69 (dt, *J* = 3.8, 12.6 Hz, 1H), 1.80 (dt, *J* = 3.8, 13.3 Hz, 2H), 2.07 (dd, *J* = 3.9, 12.9 Hz, 2H), 2.31 (s, 3H), 2.44 (t, 4H), 3.35 (s, 1H), 3.66 (t, *J* = 4.9 Hz, 4H), 6.59 (d, *J* = 8.6 Hz, 1H), 6.77 – 6.84 (m, 2H); ^13^C NMR (101 MHz, MeOD) δ 26.23, 27.10, 34.22, 46.02, 49.85, 52.74, 55.88, 111.07, 116.52, 121.03, 123.63, 134.92, 139.78, 173.78; HRMS: calcd for C_18_H_28_N_4_O_1_ 317.2336, found 317.2343 [M + H]^+^.

#### (3-Amino-4-(cyclohexylamino)phenyl)(4-fluoropiperidin-1-yl)methanone (**15**)

General procedure C was followed using compound **11** (0.062 g, 0.177 mmol), to provide (3-amino-4-(cyclohexylamino)phenyl)(4-fluoropiperidin-1-yl)methanone **14** (0.048 g, 0.150 mmol, 85% yield). ^1^H NMR (400 MHz, MeOD) δ 1.20 – 1.33 (m, 3H), 1.36 – 1.48 (m, 2H), 1.69 (dt, *J* = 3.7, 12.9 Hz, 1H), 1.81 (dt, *J* = 3.8, 13.1 Hz, 6H), 2.08 (dd, *J* = 3.7, 12.8 Hz, 2H), 3.25 – 3.29 (m, 0.5 H), 3.33 – 3.35 (m, 0.5 H), 3.67 (d, *J* = 8.1 Hz, 4H), 4.81 (tt, *J* = 3.3, 6.4 Hz, 1H), 6.60 (d, *J* = 8.7 Hz, 1H), 6.78 – 6.85 (m, 2H); ^13^C NMR (101 MHz, MeOD) δ 24.86, 25.73, 31.07 (d, *J* = 19.7 Hz), 51.40, 87.69 (d, *J* = 170.9 Hz), 109.79, 114.96, 119.42, 122.57, 133.52, 138.35, 172.66; HRMS: calcd for C_18_H_26_N_3_O_1_F_1_ 320.2133, found 320.2136 [M + H]^+^.

#### (3-Amino-4-(cyclohexylamino)phenyl)(piperazin-1-yl)methanone (**16**)

General procedure D was followed using compound **12** (0.100 g, 0.248 mmol), to provide (3-amino-4-(cyclohexylamino)phenyl)(piperazin-1-yl)methanone hydrochloride **16** (0.086 g, 0.284 mmol, 74% yield). ^1^H NMR (400 MHz, MeOD) δ 1.17 – 1.30 (m, 1H), 1.30 – 1.46 (m, 4H), 1.67 (d, *J* = 11.4 Hz, 1H), 1.78 – 1.86 (m, 2H), 2.01 – 2.09 (m, 2H), 3.38 – 3.52 (m, 1H), 3.85 (t, *J* = 5.2 Hz, 4H), 7.03 (d, *J* = 8.4 Hz, 1H), 7.20 (d, *J* = 8.5 Hz, 1H), 7.25 – 7.30 (m, 1H), 8.10 (t, *J* = 7.0 Hz, 1H), 8.65 (tt, *J* = 1.6, 8.0 Hz, 1H), 8.86 (d, *J* = 5.8 Hz, 1H), ^13^C NMR (101 MHz, MeOD) δ 24.46, 25.13, 31.14, 43.04, 54.45, 56.08, 121.22, 124.15, 127.38, 141.66, 146.85, 170.36; HRMS: calcd for C_17_H_26_N_4_O_1_ 303.2179, found 303.2191 [M + H]^+^.

#### *tert*-Butyl 3-Amino-4-(cyclohexylamino)benzoate (**20**)

General procedure C was followed using compound **19** (3.750 g, 11.70 mmol), to provide *tert*-butyl 3-amino-4-(cyclohexylamino)benzoate **20** (1.230 g, 4.240 mmol, 36% yield). ^1^H NMR (400 MHz, MeOD) δ 1.19 – 1.35 (m, 3H), 1.37 – 1.51 (m, 2H), 1.55 (s, 9H), 1.65 – 1.74 (m, 1H), 1.81 (dt, *J* = 3.7, 13.1 Hz, 2H), 2.03 – 2.13 (m, 2H), 3.32 – 3.39 (m, 1H), 6.55 (dd, *J* = 0.6, 8.4 Hz, 1H), 7.30 (d, *J* = 2.0 Hz, 1H), 7.35 (dd, *J* = 2.1, 8.4 Hz, 1H); ^13^C NMR (101 MHz, DMSO-*d*_*6*_) δ 26.23, 27.07, 28.61, 34.21, 52.63, 80.89, 110.07, 118.29, 120.19, 124.16, 133.71, 142.39, 168.80; MS (ESI) *m*/*z* = 291.0 [M+H]^+^.

#### *tert*-Butyl 4-(cyclohexylamino)-3-(cyclopentylamino)benzoate (**21**)

General procedure G was followed using compound **20** (1.000 g, 3.440 mmol) and cyclopentanone (0.915 mL, 10.33 mmol) as the corresponding ketone to provide *tert*-butyl 4-(cyclohexylamino)-3-(cyclopentylamino)benzoate **21** (1.150 g, 3.210 mmol, 91% yield). ^1^H NMR (400 MHz, MeOD) δ 1.19 – 1.36 (m, 6H), 1.38 – 1.47 (m, 2H), 1.56 (s, 9H), 1.60 – 1.72 (m, 2H), 1.72 – 1.85 (m, 4H), 1.98 – 2.11 (m, 4H), 3.32 – 3.39 (m, 1H), 3.78 (ddd, *J* = 5.4, 6.9, 12.3 Hz, 1H), 6.54 (d, *J* = 8.5 Hz, 1H), 7.23 (d, *J* = 2.0 Hz, 1H), 7.35 (dd, *J* = 2.0, 8.4 Hz, 1H); ^13^C NMR (101 MHz, MeOD) δ 25.35, 26.31. 27.09, 28.63, 34.14, 34.17, 52.76, 56.37, 80.85, 109.62, 115.10, 120.12, 123.28, 135.64, 142.75, 169.13; MS (ESI) *m*/*z* = 359.0 [M+H]^+^.

#### *tert*-Butyl 3,4-Bis(cyclohexylamino)benzoate (**22**)

General procedure G was followed using compound **20** (4.740 g, 16.32 mmol) and cyclohexanone (5.070 mL, 49.00 mmol) as the corresponding ketone to provide *tert*-butyl 3,4-bis(cyclohexylamino)benzoate **22** (5.520 g, 14.82 mmol, 91% yield). ^1^H NMR (400 MHz, MeOD) δ 1.18 – 1.50 (m, 10H), 1.56 (s, 9H), 1.69 (dt, *J* = 3.5, 12.3 Hz, 2H), 1.75 – 1.85 (m, 4H), 2.05 (dt, *J* = 3.9, 12.2 Hz, 4H), 3.15 (tt, *J* = 3.7, 10.5 Hz, 1H), 3.32 – 3.37 (m, 1H), 6.55 (d, *J* = 8.5 Hz, 1H), 7.24 (d, *J* = 2.0 Hz, 1H), 7.35 (dd, *J* = 2.0, 8.4 Hz, 1H); ^13^C NMR (101 MHz, MeOD) δ 26.24, 26.44, 27.08, 27.24, 28.61, 34.18, 34.40, 52.70, 54.14, 80.90, 110.04, 116.26, 120.11, 123.68, 134.65, 143.33, 169.05; MS (ESI) *m*/*z* = 373.0 [M+H]^+^.

#### *tert*-Butyl 3-(Benzylamino)-4-(cyclohexylamino)benzoate (**23**)

General procedure G was followed using compound **20** (2.000 g, 6.890 mmol) and benzaldehyde (2.108 mL, 20.66 mmol) as the corresponding aldehyde to provide *tert*-butyl 3-(benzylamino)-4-(cyclohexylamino)benzoate **23** (0.780 g, 2.050 mmol, 30% yield). ^1^H NMR (400 MHz, MeOD) δ 1.14 – 1.35 (m, 3H), 1.35 – 1.46 (m, 2H), 1.50 (s, 9H), 1.68 (dd, *J* = 6.6, 10.5 Hz, 1H), 1.80 (dt, *J* = 3.7, 12.7 Hz, 2H), 2.07 (dd, *J* = 3.8, 13.1 Hz, 2H), 3.31 – 3.41 (m, 1H), 4.32 (s, 2H), 6.55 (d, *J* = 8.4 Hz, 1H), 7.12 (d, *J* = 2.0 Hz, 1H), 7.19 – 7.26 (m, 1H), 7.28 – 7.34 (m, 3H), 7.38 (d, *J* = 7.1 Hz, 2H); ^13^C NMR (101 MHz, MeOD) δ 24.85, 25.27, 25.68, 27.19, 27.39, 32.80, 48.10, 51.33, 79.40, 108.30, 112.82, 118.84, 121.80, 126.53, 127.26, 128.03, 134.40, 139.82, 140.89, 167.58; MS (ESI) *m*/*z* = 381.4 [M+H]^+^.

#### *tert*-Butyl 4-(Cyclohexylamino)-3-((4-fluorobenzyl)amino)benzoate (**24**)

General procedure G was followed using compound **20** (1.230 g, 4.240 mmol) and 4-fluorobenzaldehyde (0.454 mL, 4.240 mmol) as the corresponding aldehyde to provide *tert*-butyl 4-(cyclohexylamino)-3-((4-fluorobenzyl)amino)benzoate **24** (0.893 g, 2.241 mmol, 53% yield). ^1^H NMR (400 MHz, DMSO-*d*_*6*_) δ 1.11 – 1.23 (m, 2H), 1.23 – 1.41 (m, 3H), 1.44 (s, 9H), 1.62 (dt, *J* = 4.2, 15.3 Hz, 1H), 1.74 (dt, *J* = 3.7, 13.4 Hz, 2H), 1.99 (d, *J* = 3.8 Hz, 2H), 3.26 – 3.33 (m, 1H), 4.28 (d, *J* = 5.3 Hz, 2H), 5.08 (d, *J* = 7.3 Hz, 1H), 5.50 (t, *J* = 5.5 Hz, 1H), 6.47 (d, *J* = 8.4 Hz, 1H), 6.87 (d, *J* = 2.0 Hz, 1H), 7.10 – 7.20 (m, 3H), 7.35 – 7.44 (m, 2H); ^13^C NMR (101 MHz, DMSO-*d*_*6*_) δ 24.68, 25.60, 27.97, 32.52, 46.33, 50.85, 78.54, 107.72, 110.61, 110.67, 114.99 (d, *J* = 21.3 Hz), 118.14, 118.17, 120.58, 120.62, 129.10 (d, *J* = 8.0 Hz), 133.86, 133.92, 136.10 (d, *J* = 2.9 Hz), 139.36, 139.42, 161.15 (d, *J* = 241.9 Hz), 165.74; MS (ESI) *m*/*z* = 399.4 [M+H]+.

#### 4-(Cyclohexylamino)-3-(cyclopentylamino)benzoic acid (**25**)

General procedure H was followed using compound **21** (1.348 g, 3.760 mmol), to provide 4-(cyclohexylamino)-3-((4-fluorobenzyl)amino)benzoic acid **25** (1.093 g, 3.610 mmol, 96% yield). ^1^H NMR (400 MHz, DMSO-*d*_*6*_) δ 1.11 – 1.28 (m, 4H), 1.35 (qt, *J* = 3.2, 12.5 Hz, 2H), 1.47 – 1.78 (m, 8H), 1.85 – 2.01 (m, 4H),3.31 (ddt, *J* = 3.8, 7.4, 10.6 Hz, 1H), 3.73 (p, *J* = 6.3 Hz, 1H), 6.55 (d, *J* = 8.5 Hz, 1H), 7.18 (s, 1H), 7.32 (d, *J* = 8.4 Hz, 1H); ^13^C NMR (101 MHz, DMSO-*d*_*6*_) δ, 23.91, 24.74, 25.57, 31.89, 32.46, 51.03, 55.55, 108.78, 112.90, 117.50, 122.88, 131.14, 140.26, 167.85; MS (ESI) *m*/*z* = 303.2 [M+H]^+^.

#### 3,4-Bis(cyclohexylamino)benzoic Acid (**26**)

General procedure H was followed using compound **22** (2.940 g, 7.890 mmol), to provide 4-(cyclohexylamino)-3-((4-fluorobenzyl)amino)benzoic acid **26** (2.219 g, 7.010 mmol, 89% yield). ^1^H NMR (400 MHz, MeOD) δ 1.22 – 1.54 (m, 10H), 1.72 (dt, *J* = 3.6, 13.2 Hz, 2H), 1.78 – 1.91 (m, 3H), 1.98 – 2.12 (m, 5H), 3.37 – 3.54 (m, 2H), 6.93 (d, *J* = 8.7 Hz, 1H), 7.76 (s, 1H), 7.86 (d, *J* = 8.7 Hz, 1H); ^13^C NMR (101 MHz, MeOD) δ 25.68, 26.00, 26.14, 26.77, 31.03, 33.55, 53.23, 60.62, 113.86, 116.53, 119.43, 126.67, 132.03, 145.07, 169.08; MS (ESI) *m*/*z* = 317.3 [M+H]^+^.

#### 3-(Benzylamino)-4-(cyclohexylamino)benzoic Acid (**27**)

General procedure H was followed using compound **23** (0.790 g, 2.080 mmol), to provide 4-(cyclohexylamino)-3-((4-fluorobenzyl)amino)benzoic acid **27** (0.800 g, 2.460 mmol, 88% yield). ^1^H NMR (400 MHz, DMSO-*d*_*6*_) δ 1.23 – 1.26 (m, 2H), 1.29 – 1.41 (m, 2H), 1.56 – 1.65 (m, 1H), 1.69 – 1.74 (m, 2H), 1.81 – 1.93 (m, 1H), 1.98 (dd, *J* = 3.6, 12.5 Hz, 2H), 2.90 – 3.04 (m, 1H), 3.33 (td, *J* = 3.3, 6.8 Hz, 1H), 4.31 (s, 2H), 6.53 (d, *J* = 8.4 Hz, 1H), 6.97 (d, *J* = 2.0 Hz, 1H), 7.21 – 7.27 (m, 2H), 7.34 (td, *J* = 6.1, 8.3 Hz, 4H), 7.72 (s, 1H); ^13^C NMR (101 MHz, DMSO-*d*_*6*_) δ 24.68, 25.57, 32.46, 40.15, 47.27, 51.05, 111.40, 114.43, 117.34, 121.26, 126.83, 127.40, 128.32, 133.69, 136.54, 139.50, 167.91; MS (ESI) *m*/*z* = 325.0 [M+H]^+^.

#### 4-(Cyclohexylamino)-3-((4-fluorobenzyl)amino)benzoic Acid (**28**)

General procedure H was followed using compound **24** (0.890 g, 2.230 mmol), to provide 4-(cyclohexylamino)-3-((4-fluorobenzyl)amino)benzoic acid **28** (1.000 g, 2.190 mmol, 97% yield). ^1^H NMR (400 MHz, MeOD) δ 1.20 – 1.34 (m, 3H), 1.34 – 1.50 (m, 3H), 1.80 – 1.88 (m, 2H), 2.01 – 2.10 (m, 2H), 3.44 (ddt, *J* = 3.8, 7.4, 10.5 Hz, 1H), 4.42 (s, 2H), 6.84 (d, *J* = 8.5 Hz, 1H), 7.03 – 7.13 (m, 2H), 7.34 – 7.43 (m, 3H), 7.57 (dd, *J* = 1.9, 8.4 Hz, 1H); ^13^C NMR (101 MHz, DMSO-*d*_*6*_) δ 24.66, 25.58, 32.55, 46.38, 51.89, 107.18, 110.98, 115.38 (d, *J* = 21.3 Hz), 118.18, 120.89, 129.18 (d, *J* = 8.0 Hz), 133.56, 136.18 (d, *J* = 2.9 Hz), 139.47, 161.23 (d, *J* = 241.9 Hz), 166.78; MS (ESI) *m*/*z* = 343.2 [M+H]^+^.

#### *tert*-Butyl 4-(4-(Cyclohexylamino)-3-(cyclopentylamino)benzoyl)piperazine-1-carboxylate (**29**)

General procedure I was followed using compound **25** (0.236 g, 0.780 mmol) and *tert*-butyl piperazine-1-carboxylate (0.203 g, 1.093 mmol) as the corresponding amine to provide *tert*-butyl 4-(4-(cyclohexylamino)-3-(cyclopentylamino)benzoyl)piperazine-1-carboxylate **29** (0.148 g, 0.314 mmol, 40% yield). ^1^H NMR (400 MHz, MeOD) δ 1.18 – 1.33 (m, 3H), 1.35 – 1.44 (m, 2H), 1.47 (s, 9H), 1.52 – 1.72 (m, 3H), 1.73 – 1.85 (m, 3H), 1.96 – 2.11 (m, 4H), 3.24 – 3.30 (m, 1H), 3.48 (d, *J* = 6.0 Hz, 3H), 3.63 (dd, *J* = 3.8, 6.7 Hz, 4H), 3.78 (p, *J* = 6.3 Hz, 1H), 6.61 (d, *J* = 8.2 Hz, 1H), 6.71 (d, *J* = 1.9 Hz, 1H), 6.80 (dd, *J* = 1.9, 8.1 Hz, 1H);^13^C NMR (101 MHz, MeOD) δ 25.30, 26.34, 27.14, 28.61, 30.90, 34.16, 34.22, 53.00, 56.10, 81.64, 110.91, 113.11, 119.97, 123.74, 126.13, 136.77, 140.07, 156.31, 174.58; MS (ESI) *m*/*z* = 471.5 [M+H]^+^.

#### *tert*-Butyl 4-(3,4-Bis(cyclohexylamino)benzoyl)piperazine-1-carboxylate (**30**)

General procedure I was followed using compound **26** (0.300 g, 0.948 mmol) and *tert*-butyl piperazine-1-carboxylate (0.247 g, 1.327 mmol) as the corresponding amine to provide *tert*-butyl 4-(3,4-bis(cyclohexylamino)benzoyl)piperazine-1-carboxylate **30** (0.202 g, 0.417 mmol, 44% yield). ^1^H NMR (400 MHz, MeOD) δ 1.19 – 1.47 (m, 7H), 1.49 (s, 9H), 1.71 (dt, *J* = 3.7, 12.7 Hz, 3H), 1.82 (dt, *J* = 3.8, 13.6 Hz, 5H), 2.07 (dt, *J* = 4.1, 11.1 Hz, 5H), 3.20 (tt, *J* = 3.7, 10.5 Hz, 1H), 3.29 (dt, *J* = 3.6, 10.4 Hz, 1H), 3.49 (d, *J* = 6.0 Hz, 4H), 3.64 (dd, *J* = 3.8, 6.7 Hz, 4H), 6.63 (d, *J* = 8.2 Hz, 1H), 6.74 (d, *J* = 1.9 Hz, 1H), 6.82 (dd, *J* = 1.9, 8.1 Hz, 1H); ^13^C NMR (101 MHz, MeOD) δ 26.28, 26.38, 27.14, 27.20, 28.61, 30.90, 34.23, 34.37, 52.98, 53.64, 81.63, 111.39, 113.86, 120.17, 123.81, 126.12, 135.94, 140.44, 156.32, 174.49; MS (ESI) *m*/*z* = 485.5 [M+H]^+^.

#### *tert*-Butyl 4-(3-(Benzylamino)-4-(cyclohexylamino)benzoyl)piperazine-1-carboxylate (**31**)

General procedure I was followed using compound **27** (0.200 g, 0.438 mmol) and *tert*-butyl piperazine-1-carboxylate (0.114 g, 0.613 mmol) as the corresponding amine to provide *tert*-butyl 4-(3-(benzylamino)-4-(cyclohexylamino)benzoyl)piperazine-1-carboxylate **31** (0.125 g, 0.254 mmol, 58% yield). ^1^H NMR (400 MHz, CDCl_3_) δ 1.30 (dt, *J* = 13.3, 46.6 Hz, 6H), 1.47 (s, 9H), 1.64 (q, *J* = 14.8 Hz, 4H), 1.78 (d, *J* = 12.7 Hz, 3H), 2.07 (d, *J* = 11.9 Hz, 2H), 3.41 (d, *J* = 63.0 Hz, 10H), 4.32 (s, 2H), 6.72 (s, 2H), 6.90 (d, *J* = 8.0 Hz, 1H), 7.30 (dt, *J* = 3.0, 7.3 Hz, 1H), 7.32 – 7.44 (m, 4H). MS (ESI) *m*/*z* = 493.4 [M+H]^+^.

#### *tert*-Butyl 4-(4-(Cyclohexylamino)-3-((4-fluorobenzyl)amino)benzoyl)piperazine-1-carboxylate (**32**)

General procedure I was followed using compound **28** (0.200 g, 0.584 mmol) and *tert*-butyl piperazine-1-carboxylate (0.163 g, 0.876 mmol) as the corresponding amine to provide *tert*-butyl 4-(4-(cyclohexylamino)-3-((4-fluorobenzyl)amino)benzoyl)piperazine-1-carboxylate **32** (0.171 g, 0.335 mmol, 57% yield). ^1^H NMR (400 MHz,MeOD) δ 1.19 – 1.35 (m, 3H), 1.40 (dt, *J* = 3.2, 12.6 Hz, 2H), 1.46 (s, 9H), 1.69 (dt, *J* = 3.1, 12.7 Hz, 1H), 1.81 (dt, *J* = 3.7, 13.1 Hz, 2H), 2.09 (dd, *J* = 3.8, 12.6 Hz, 2H), 3.15 – 3.28 (m, 4H), 3.32 – 3.56 (m, 5H), 4.36 (s, 2H), 4.60 (s, 2H), 6.43 (d, *J* = 2.0 Hz, 1H), 6.63 (d, *J* = 8.2 Hz, 1H), 6.80 (dd, *J* = 1.9, 8.2 Hz, 1H), 6.98 – 7.08 (m, 2H), 7.30 – 7.39 (m, 2H); ^13^C NMR (101 MHz, MeOD) δ 26.27, 27.13, 28.60, 34.27, 47.94, 49.28, 49.50, 52.91, 81.63, 111.06, 112.35, 116.12 (d, *J* = 21.5 Hz), 120.41, 123.52, 129.96 (d, *J* = 8.0 Hz), 135.98, 137.02 (d, *J* = 3.0 Hz), 139.78, 156.22, 162.95 (d, *J* = 243.4 Hz), 164.49, 174.33; MS (ESI) *m*/*z* = 511.4 [M+H]^+^.

#### *tert*-Butyl (1-(3,4-Bis(cyclohexylamino)benzoyl)pyrrolidin-3-yl)carbamate (**33**)

General procedure I was followed using compound **26** (0.150 g, 0.474 mmol) and *tert*-butyl pyrrolidin-3-ylcarbamate (0.124 g, 0.664 mmol) as the corresponding amine to provide *tert*-butyl (1-(3,4-bis(cyclohexylamino)benzoyl)pyrrolidin-3-yl)carbamate **33** (0.089 g, 0.184 mmol, 39% yield). ^1^H NMR (400 MHz, MeOD) δ 1.24 (ttt, *J* = 12.7, 6.9, 3.6 Hz, 6H), 1.54 – 1.33 (m, 13H), 1.73 – 1.63 (m, 2H), 1.79 (dt, *J* = 13.4, 3.7 Hz, 4H), 1.95 – 1.84 (m, 1H), 2.05 (dt, *J* = 12.7, 3.5 Hz, 4H), 2.23 – 2.10 (m, 1H), 3.18 (tt, *J* = 10.5, 3.7 Hz, 1H), 3.27 (dt, *J* = 10.3, 3.6 Hz, 1H), 3.49 – 3.37 (m, 1H), 3.84 – 3.55 (m, 3H), 4.21 – 3.96 (m, 1H), 6.59 (d, *J* = 8.3 Hz, 1H), 6.86 (d, *J* = 6.1 Hz, 1H), 6.97 – 6.88 (m, 1H); ^13^C NMR (101 MHz, MeOD) δ 26.28, 26.39, 27.13, 27.21, 28.73, 30.56, 33.17, 34.22, 34.39, 45.74, 50.55, 51.99, 52.91, 53.01, 53.70, 56.52, 80.29, 110.95, 114.18, 120.46, 124.95, 135.53, 140.62, 157.97, 173.29; MS (ESI) *m*/*z* = 485.4 [M+H]^+^.

#### *tert*-Butyl (1-(3-(Benzylamino)-4-(cyclohexylamino)benzoyl)pyrrolidin-3-yl)carbamate (**34**)

General procedure I was followed using compound **27** (0.200 g, 0.438 mmol) and *tert*-butyl pyrrolidin-3-ylcarbamate (0.114 g, 0.613 mmol) as the corresponding amine to provide *tert*-butyl (1-(3-(benzylamino)-4-(cyclohexylamino)benzoyl)pyrrolidin-3-yl)carbamate **34** (0.125 g, 0.254 mmol, 58% yield). ^1^H NMR (400 MHz, MeOD) δ 1.26 – 1.33 (m, 2H), 1.44 (d, *J* = 16.3 Hz, 12H), 1.69 (tt, *J* = 6.8, 15.1 Hz, 2H), 1.74 – 1.95 (m, 3H), 2.09 (dd, *J* = 3.5, 12.6 Hz, 2H), 2.96 – 3.28 (m, 2H), 3.35 (dd, *J* = 3.7, 10.4 Hz, 1H), 3.61 (ddd, *J* = 7.2, 15.5, 63.5 Hz, 2H), 3.83 – 4.08 (m, 1H), 4.37 (s, 2H), 6.61 (dd, *J* = 4.9, 8.3 Hz, 2H), 6.90 (d, *J* = 8.3 Hz, 1H), 7.21 (t, *J* = 7.4 Hz, 1H), 7.30 (dd, *J* = 6.7, 8.4 Hz, 2H), 7.36 (d, *J* = 7.1 Hz, 2H); ^13^C NMR (101 MHz, MeOD) δ 19.46, 21.65, 24.88, 25.74, 27.35, 32.84, 32.88, 51.51, 53.40, 60.14, 78.93, 109.36, 111.29, 118.88, 126.52, 126.92, 128.14, 134.56, 138.45, 139.82, 171.59, 171.88; MS (ESI) *m*/*z* = 493.4 [M+H]^+^.

#### (4-(Cyclohexylamino)-3-(cyclopentylamino)phenyl)(morpholino)methanone (**35**)

General procedure I was followed using compound **25** (0.150 g, 0.496 mmol) and morpholine (0.060 g, 0.694 mmol) as the corresponding amine to provide (4-(cyclohexylamino)-3-(cyclopentylamino)phenyl)(morpholino)methanone **35** (0.150 g, 0.404 mmol, 81% yield). ^1^H NMR (400 MHz, MeOD) δ 1.18 – 1.34 (m, 3H), 1.42 (qt, *J* = 3.3, 12.7 Hz, 2H), 1.52 – 1.74 (m, 5H), 1.74 – 1.84 (m, 4H), 1.96 – 2.11 (m, 4H), 3.24 – 3.29 (m, 1H), 3.66 (dd, *J* = 4.0, 7.2 Hz, 8H), 3.72 – 3.84 (m, 1H), 6.60 (d, *J* = 8.2 Hz, 1H), 6.70 (d, *J* = 2.0 Hz, 1H), 6.79 (dd, *J* = 2.0, 8.1 Hz, 1H); ^13^C NMR (101 MHz, MeOD) δ 25.30, 26.34, 27.14, 34.16, 34.22, 53.00, 56.09, 67.96, 110.94, 113.10, 119.91, 123.67, 136.80, 139.99, 174.38; HRMS: calcd for C_22_H_33_N_3_O_2_ 372.2646, found 372.2653 [M + H]^+^.

#### (3,4-Bis(cyclohexylamino)phenyl)(morpholino)methanone (**36**)

General procedure I was followed using compound **26** (0.200 g, 0.632 mmol) and morpholine (0.077 g, 0.885 mmol) as the corresponding amine to provide bis(cyclohexylamino)phenyl)(morpholino)methanone **36** (0.048 g, 0.126 mmol, 20% yield). ^1^H NMR (400 MHz, MeOD) δ 1.14 – 1.33 (m, 7H), 1.34 – 1.48 (m, 3H), 1.69 (dt, *J* = 3.6, 12.9 Hz, 2H), 1.78 – 1.86 (m, 3H), 2.05 (dt, *J* = 3.7, 12.7 Hz, 5H), 3.18 (tt, *J* = 3.7, 10.5 Hz, 1H), 3.24 – 3.30 (m, 1H), 3.67 (h, *J* = 4.2 Hz, 8H), 6.61 (d, *J* = 8.2 Hz, 1H), 6.71 (d, *J* = 2.0 Hz, 1H), 6.79 (dd, *J* = 2.0, 8.1 Hz, 1H); ^13^C NMR (101 MHz, MeOD) δ 26.29, 26.38, 27.14, 27.21, 34.23, 34.37, 52.99, 53.66, 67.95, 111.41, 113.86, 120.11, 123.75, 135.98, 140.35, 174.28; HRMS: calcd for C_23_H_35_N_3_O_2_ 386.2802, found 386.2802 [M + H]^+^.

#### (3-(Benzylamino)-4-(cyclohexylamino)phenyl)(morpholino)methanone (**37**)

General procedure I was followed using compound **27** (0.200 g, 0.438 mmol) and morpholine (0.074 mL, 0.863 mmol) as the corresponding amine to provide 3-(benzylamino)-4-(cyclohexylamino)phenyl)(morpholino)methanone **37** (0.081 g, 0.206 mmol, 33% yield). ^1^H NMR (400 MHz, DMSO-*d*_*6*_) δ 1.14 – 1.28 (m, 3H), 1.35 (t, *J* = 12.5 Hz, 2H), 1.63 (d, *J* = 12.5 Hz, 1H), 1.71 – 1.79 (m, 2H), 2.00 (d, *J* = 12.1 Hz, 2H), 3.24 – 3.31 (m, 4H), 3.31 (s, 1H), 3.37 – 3.43 (m, 4H), 4.32 (d, *J* = 5.5 Hz, 2H), 4.79 (d, *J* = 7.2 Hz, 1H), 5.53 (t, *J* = 5.6 Hz, 1H), 6.29 (d, *J* = 1.9 Hz, 1H), 6.47 (d, *J* = 8.2 Hz, 1H), 6.64 (dd, *J* = 1.9, 8.1 Hz, 1H), 7.23 (dq, *J* = 3.8, 8.7 Hz, 1H), 7.29 – 7.36 (m, 4H); ^13^C NMR (101 MHz, DMSO-*d*_*6*_) δ 24.71, 25.66, 32.69, 46.71, 50.93, 66.10, 108.37, 109.72, 118.08, 121.98, 126.60, 126.95, 128.32, 134.03, 136.79, 139.93, 170.47; HRMS: calcd for C_24_H_31_N_3_O_2_ 394.2489, found 394.2498 [M + H]^+^.

#### (4-(Cyclohexylamino)-3-((4-fluorobenzyl)amino)phenyl)(4-methylpiperazin-1-yl)methanone (**38**)

General procedure I was followed using compound **28** (0.200 g, 0.584 mmol) and morpholine (0.071 mL, 0.818 mmol) as the corresponding amine to provide **38** (4-(cyclohexylamino)-3-((4-fluorobenzyl)amino)phenyl)(morpholino)methanone (0.126 g, 0.306 mmol, 52% yield). ^1^H NMR (400 MHz,MeOD) δ 1.19 – 1.35 (m, 3H), 1.36 – 1.51 (m, 2H), 1.68 (s, 1H), 1.81 (dt, *J* = 3.7, 13.1 Hz, 2H), 2.04 – 2.13 (m, 2H), 3.32– 3.37 (m, 1H), 3.39 – 3.57 (m, 8H), 4.36 (s, 2H), 4.60 (s, 2H), 6.44 (d, *J* = 2.0 Hz, 1H), 6.63 (d, *J* = 8.2 Hz, 1H), 6.79 (dd, *J* = 1.9, 8.1 Hz, 1H), 6.98 – 7.09 (m, 2H), 7.32 – 7.40 (m, 2H); ^13^C NMR (101 MHz, MeOD) δ 26.27, 27.13, 34.27, 48.04, 52.92, 67.81, 111.09, 111.21, 112.39, 116.11 (d, *J* = 21.7 Hz), 120.32, 123.50, 129.99 (d, *J* = 8.1 Hz), 135.10, 136.12,137.04 (d, *J* = 2.7 Hz), 139.72, 142.68, 162.08, 174.17; HRMS: calcd for C_24_H_30_N_3_O_2_F_1_ 412.2395, found 412.2401 [M + H]^+^.

#### (4-(Cyclohexylamino)-3-(cyclopentylamino)phenyl)(4-methylpiperazin-1-yl)methanone (**39**)

General procedure I was followed using compound **25** (0.100 g, 3.310 mmol) and 1-methylpiperazine (0.046 g, 0.463 mmol) as the corresponding amine to provide (4-(cyclohexylamino)-3-(cyclopentylamino)phenyl)(4-methylpiperazin-1-yl)methanone **39** (0.120 g, 0.312 mmol, 94% yield). ^1^H NMR (400 MHz, MeOD) δ 1.26 (ddt, *J* = 6.6, 12.3, 15.1 Hz, 3H), 1.36 – 1.48 (m, 2H), 1.51 – 1.73 (m, 5H), 1.74 – 1.85 (m, 4H), 1.96 – 2.12 (m, 4H), 2.35 (s, 3H), 2.50 (t, *J* = 5.1 Hz, 4H), 3.24 – 3.29 (m, 1H), 3.64 – 3.73 (m, 4H), 3.75 – 3.81 (m, 1H), 6.60 (d, *J* = 8.2 Hz, 1H), 6.69 (d, *J* = 1.9 Hz, 1H), 6.78 (dd, *J* = 1.9, 8.1 Hz, 1H); ^13^C NMR (101 MHz, MeOD) δ 25.30, 26.35, 27.14, 34.17, 34.23, 45.98, 53.01, 55.92, 56.09, 110.94, 113.04, 119.86, 123.82, 136.77, 139.98, 174.32; HRMS: calcd for C_23_H_36_N_4_O_1_ 385.2962, found 385.2969 [M + H]^+^.

#### (3,4-Bis(cyclohexylamino)phenyl)(4-methylpiperazin-1-yl)methanone (**40**)

General procedure I was followed using compound **26** (0.200 g, 0.632 mmol) and 1-methylpiperazine (0.089 g, 0.885 mmol) as the corresponding amine to provide (3,4-bis(cyclohexylamino)phenyl)(4-methylpiperazin-1-yl)methanone **40** (0.046 g, 0.115 mmol, 18% yield). ^1^H NMR (400 MHz, MeOD) δ 1.17 – 1.33 (m, 7H), 1.34 – 1.50 (m, 3H), 1.63 – 1.74 (m, 2H), 1.80 (dp, *J* = 3.6, 10.8 Hz, 3H), 2.05 (dt, *J* = 3.6, 12.8 Hz, 5H), 2.33 (s, 3H), 2.48 (t, *J* = 5.1 Hz, 4H), 3.17 (ddd, *J* = 3.7, 6.8, 10.5 Hz, 1H), 3.24 – 3.31 (m, 1H), 3.64 – 3.71 (m, 4H), 6.61 (d, *J* = 8.2 Hz, 1H), 6.70 (d, *J* = 1.9 Hz, 1H), 6.78 (dd, *J* = 1.9, 8.1 Hz, 1H); ^13^C NMR (101 MHz, MeOD) δ 26.29, 26.39, 27.14, 27.21, 34.24, 34.36, 46.00, 52.99, 53.66, 55.93, 111.42, 113.77, 120.05, 123.91, 135.94, 140.32, 174.22; HRMS: calcd for C_24_H_38_N_4_O_1_ 399.3118, found 399.3124 [M + H]^+^.

#### (3-(Benzylamino)-4-(cyclohexylamino)phenyl)(4-methylpiperazin-1-yl)methanone (**41**)

General procedure I was followed using compound **27** (0.200 g, 0.438 mmol) and 1-methylpiperazine (0.061 g, 0.613 mmol) as the corresponding amine to provide (3-(benzylamino)-4-(cyclohexylamino)phenyl)(4-methylpiperazin-1-yl)methanone **41** (0.093 g, 0.229 mmol, 52% yield). ^1^H NMR (400 MHz, MeOD) δ 1.31 (td, *J* = 3.4, 11.6 Hz, 3H), 1.37 – 1.52 (m, 2H), 1.70 (s, 1H), 1.83 (dt, *J* = 3.7, 13.0 Hz, 2H), 2.12 (dd, *J* = 3.8, 12.5 Hz, 2H), 2.27 (s, 5H), 3.34 – 3.57 (m, 4H), 4.41 (br. s, 2H), 6.43 (d, *J* = 1.9 Hz, 1H), 6.65 (d, *J* = 8.2 Hz, 1H), 6.80 (dd, *J* = 1.9, 8.1 Hz, 1H), 7.19 – 7.27 (m, 1H), 7.28 – 7.39 (m, 4H); ^13^C NMR (101 MHz, MeOD) δ 24.88, 25.75, 32.89, 44.55, 47.30, 51.54, 54.37, 109.76, 110.86, 118.70, 122.31, 126.49, 126.74, 128.18, 134.81, 138.18, 139.78, 172.67; HRMS: calcd for C_25_H_34_N_4_O_1_ 407.2805, found 407.2814 [M + H]^+^.

#### (4-(Cyclohexylamino)-3-((4-fluorobenzyl)amino)phenyl)(morpholino)methanone (**42**)

General procedure I was followed using compound **28** (0.200 g, 0.584 mmol) and 1-methylpiperazine (0.088 g, 0.876 mmol) as the corresponding amine to provide ((4-(cyclohexylamino)-3-((4-fluorobenzyl)amino)phenyl)(4-methylpiperazin-1-yl)methanone **42** (0.110 g, 0.259 mmol, 44% yield). ^1^H NMR (400 MHz, DMSO*-d*_*6*_) δ 1.12 – 1.28 (m, 3H), 1.36 (qt, *J* = 3.3, 12.5 Hz, 2H), 1.63 (dt, *J* = 3.6, 12.7 Hz, 1H), 1.75 (dq, *J* = 3.6, 13.1 Hz, 2H), 1.99 (dd, *J* = 3.8, 12.6 Hz, 2H), 2.08 – 2.11 (m, 4H), 2.13 (s, 3H), 3.21 – 3.32 (m, 5H), 4.29 (d, *J* = 5.3 Hz, 2H), 4.75 (d, *J* = 7.2 Hz, 1H), 5.52 (t, *J* = 5.5 Hz, 1H), 6.26 (d, *J* = 1.9 Hz, 1H), 6.47 (d, *J* = 8.2 Hz, 1H), 6.61 (dd, *J* = 1.9, 8.0 Hz, 1H), 7.15 (t, *J* = 8.9 Hz, 2H), 7.32 – 7.40 (m, 2H); ^13^C NMR (101 MHz, DMSO*-d*_*6*_) δ 24.42, 25.38, 32.41, 45.33, 45.77, 50.64, 54.28, 108.11, 109.37, 114.74 (d, *J* = 21.1 Hz), 117.62, 122.28, 128.49 (d, *J* = 8.0 Hz), 133.64, 135.72 (d, *J* = 2.8 Hz), 136.37,160.83 (d, *J* = 241.7 Hz), 170.02; HRMS: calcd for C_25_H_33_N_4_O_1_F_1_ 425.2711, found 425.2720 [M + H]^+^.

#### (3,4-Bis(cyclohexylamino)phenyl)(4-fluoropiperidin-1-yl)methanone (**43**)

General procedure I was followed using compound **26** (0.200 g, 0.632 mmol) and 4-fluoropiperidine (0.091 g, 0.885 mmol) as the corresponding amine to provide (3,4-bis(cyclohexylamino)phenyl)(4-fluoropiperidin-1-yl)methanone **43** (0.088 g, 0.219 mmol, 35% yield). ^1^H NMR (400 MHz, MeOD) δ 1.17 – 1.51 (m, 10H), 1.69 (dt, *J* = 3.5, 15.7 Hz, 2H), 1.74 – 1.86 (m, 4H), 1.85 – 1.99 (m, 4H), 2.05 (dt, *J* = 3.7, 12.5 Hz, 4H), 3.18 (tt, *J* = 3.7, 10.5 Hz, 1H), 3.26 (dd, *J* = 3.7, 10.4 Hz, 1H), 3.69 (t, *J* = 5.9 Hz, 4H), 4.81 (tt, *J* = 3.3, 6.5 Hz, 0.5 H), 4.93 (tt, *J* = 3.3, 6.5 Hz, 0.5 H), 6.62 (d, *J* = 8.2 Hz, 1H), 6.71 (d, *J* = 1.9 Hz, 1H), 6.79 (dd, *J* = 1.9, 8.1 Hz, 1H); ^13^C NMR (101 MHz, MeOD) δ 26.29, 26.38, 27.14, 27.20, 30.89, 32.60 (d, *J* = 19.3 Hz), 34.24, 34.36, 53.02, 53.65, 89.11 (d, *J* = 171.0 Hz), 111.55, 113.59, 119.82, 124.29, 135.96, 140.25, 174.36; HRMS: calcd for C_24_H_36_N_3_O_1_F_1_ 402.2915, found 402.2923 [M + H]^+^.

#### (3-(Benzylamino)-4-(cyclohexylamino)phenyl)(4-fluoropiperidin-1-yl)methanone (**44**)

General procedure I was followed using compound **27** (0.300 g, 0.948 mmol) and 4-fluoropiperidine (0.063 g, 0.613 mmol) as the corresponding amine to provide (3-(benzylamino)-4-(cyclohexylamino)phenyl)(4-fluoropiperidin-1-yl)methanone **44** (0.038 g, 0.093 mmol, 21% yield). ^1^H NMR (400 MHz, DMSO*-d*_*6*_) δ 1.13 – 1.27 (m, 4H), 1.35 (t, *J* = 12.5 Hz, 2H), 1.51 (s, 2H), 1.63 (d, *J* = 10.9 Hz, 2H), 1.70 – 1.78 (m, 3H), 2.00 (d, *J* = 12.3 Hz, 2H), 3.23 – 3.30 (m, 2H), 3.38 – 3.46 (m, 2H), 4.31 (d, *J* = 5.3 Hz, 2H), 4.69 – 4.88 (m, 2H), 5.49 (t, *J* = 5.6 Hz, 1H), 6.33 (d, *J* = 1.9 Hz, 1H), 6.47 (d, *J* = 8.4 Hz, 1H), 6.63 (dd, *J* = 1.9, 8.0 Hz, 1H), 7.19 – 7.27 (m, 1H), 7.33 (q, *J* = 3.2 Hz, 4H); ^13^C NMR (101 MHz, DMSO*-d*_*6*_) δ 25.19, 26.14, 29.96, 31.55 (d, *J* = 19.1 Hz), 33.18, 47.25, 51.41, 88.91 (d, *J* = 169.5 Hz), 108.82, 109.92, 118.19, 123.01, 127.03, 127.08, 127.50, 128.76, 128.92, 134.63, 137.15, 140.41, 170.97; HRMS: calcd for C_25_H_32_N_3_O_1_F_1_ 410.2601, found 410.2614 [M + H]^+^.

#### (4-(Cyclohexylamino)-3-((4-fluorobenzyl)amino)phenyl)(4-fluoropiperidin-1-yl)methanone (**45**)

General procedure I was followed using compound **28** (0.200 g, 0.584 mmol) and 4 4-fluoropiperidine (0.084 g, 0.818 mmol) as the corresponding amine to provide (4-(cyclohexylamino)-3-((4-fluorobenzyl)amino)phenyl)(4-fluoropiperidin-1-yl)methanone **45** (0.039 g, 0.091 mmol, 16% yield). ^1^H NMR (400 MHz, MeOD) δ 1.21 – 1.33 (m, 3H), 1.35 – 1.49 (m, 2H), 1.58 – 1.64 (m, 1H), 1.81 (d, *J* = 13.9 Hz, 2H), 2.09 (d, *J* = 12.3 Hz, 2H), 3.32 – 3.37 (m, 1H), 3.35 – 3.56 (m, 2H), 4.35 (s, 2H), 4.61 (s, 6H), 4.72 (dq, *J* = 3.2, 6.4 Hz, 1H), 6.45 (d, *J* = 2.0 Hz, 1H), 6.63 (d, *J* = 8.2 Hz, 1H), 6.78 (dd, *J* = 1.9, 8.2 Hz, 1H), 6.97 – 7.08 (m, 2H), 7.31 – 7.39 (m, 2H); ^13^C NMR (101 MHz, MeOD) δ 26.27, 27.14, 30.07, 32.41 (d, *J* = 17.3 Hz), 34.28, 48.10, 52.96, 88.98 (d, *J* = 170.7 Hz), 111.67, 112.15, 116.10 (d, *J* = 21.6 Hz), 119.95, 124.12, 130.06 (d, *J* = 8.5 Hz), 136.66 (d, *J* = 80.5 Hz), 139.60, 152.56, 163.32 (d, *J* = 243.7 Hz), 174.25; HRMS: calcd for C_25_H_31_N_3_O_1_F_2_ 428.2508, found 428.2524 [M + H]^+^.

#### (4-(Cyclohexylamino)-3-(cyclopentylamino)phenyl)(piperazin-1-yl)methanone (**46**)

General procedure D was followed using compound **29** (0.140 g, 0.297 mmol), to provide (3,4-bis(cyclohexylamino)phenyl)(piperazin-1-yl)methanone **46** (0.126 g, 0.273 mmol, 88% yield). ^1^H NMR (400 MHz, MeOD) δ 1.19 – 1.51 (m, 5H), 1.63 – 1.72 (m, 3H), 1.72 – 1.91 (m, 7H), 1.95 – 2.09 (m, 4H), 3.31 (t, *J* = 6.1 Hz, 4H), 3.46 (td, *J* = 3.9, 10.4 Hz, 1H), 3.87 (s, 4H), 3.99 (q, *J* = 6.4 Hz, 1H), 7.11 (d, *J* = 8.0 Hz, 1H), 7.23 (d, *J* = 8.0 Hz, 1H), 7.30 (s, 1H); ^13^C NMR (101 MHz, MeOD) δ 24.93, 25.85, 26.46, 31.82, 32.34, 44.40, 56.42, 60.37, 119.43, 121.87, 125.53, 171.62; HRMS: calcd for C_22_H_34_N_4_O_1_ 371.2805, found 371.2810 [M + H]^+^.

#### (3,4-Bis(cyclohexylamino)phenyl)(piperazin-1-yl)methanone (**47**)

General procedure D was followed using compound **30** (0.202 g, 0.417 mmol), to provide (3-(benzylamino)-4-(cyclohexylamino)phenyl)(piperazin-1-yl)methanone **47** (0.152 g, 0.308 mmol, 74% yield). ^1^H NMR (400 MHz, MeOD) δ 1.20 – 1.60 (m, 10H), 1.70 (dd, *J* = 5.9, 9.9 Hz, 2H), 1.86 (ddd, *J* = 3.4, 7.9, 12.4 Hz, 4H), 2.00 – 2.12 (m, 4H), 3.30 – 3.38 (m, 4H), 3.43 – 3.58 (m, 2H), 3.89 (t, *J* = 5.2 Hz, 4H), 7.06 (d, *J* = 8.4 Hz, 1H), 7.32 – 7.40 (m, 2H); HRMS: calcd for C_23_H_36_N_4_O_1_ 385.2962, found 385.2953 [M + H]^+^.

#### (3-(Benzylamino)-4-(cyclohexylamino)phenyl)(piperazin-1-yl)methanone (**48**)

General procedure D was followed using compound **31** (0.125 g, 0.254 mmol), to provide (3-(benzylamino)-4-(cyclohexylamino)phenyl)(piperazin-1-yl)methanone **48** (0.059 g, 0.117 mmol, 46% yield). ^1^H NMR (400 MHz, MeOD) δ 1.27 – 1.40 (m, 3H), 1.48 (td, *J* = 3.2, 11.8 Hz, 2H), 1.71 (d, *J* = 11.2 Hz, 1H), 1.88 (dt, *J* = 3.2, 13.1 Hz, 2H), 2.06 (dd, *J* = 3.9, 12.4 Hz, 2H), 3.12 (br.s, 4H), 3.58 (ddt, *J* = 4.1, 7.5, 11.2 Hz, 1H), 3.62 – 3.99 (m, 4H), 4.53 (s, 2H), 6.79 (d, *J* = 1.8 Hz, 1H), 6.96 (dd, *J* = 1.8, 8.2 Hz, 1H), 7.25 (d, *J* = 8.2 Hz, 1H), 7.30 – 7.41 (m, 5H); ^13^C NMR (101 MHz, MeOD) δ 18.53, 24.36, 24.82, 30.03, 31.72, 42.81, 57.50, 114.48, 118.60, 122.15, 126.16, 127.53, 127.56, 128.63, 131.92, 136.95, 170.55; HRMS: calcd for C_24_H_32_N_4_O_1_ 393.2649, found 393.2654 [M + H]^+^.

#### (4-(Cyclohexylamino)-3-((4-fluorobenzyl)amino)phenyl)(piperazin-1-yl)methanone (**49**)

General procedure D was followed using compound **32** (0.165 g, 0.323 mmol), to provide (4-(cyclohexylamino)-3-((4-fluorobenzyl)amino)phenyl)(piperazin-1-yl)methanone **49** (0.119 g, 0.290 mmol, 90% yield). ^1^H NMR (400 MHz,DMSO-*d*_*6*_) δ 1.06 – 1.34 (m, 3H), 1.45 (q, *J* = 12.0 Hz, 2H), 1.60 (d, 1H), 1.76 (dt, *J* = 3.5, 13.2 Hz, 2H), 1.94 – 2.02 (m, 2H), 3.02 (br. s, 4H), 3.31 – 3.48 (m, 5H), 4.37 (s, 2H), 6.71 (s, 1H), 6.82 (d, *J* = 8.0 Hz, 1H), 7.02 - 7.12 (m, 1H), 7.16 (t, *J* = 8.7 Hz, 2H), 7.45 (dd, *J* = 5.5, 8.4 Hz, 2H), 9.35 (br. s, 2H); ^13^C NMR (101 MHz, DMSO-*d*_*6*_) δ 24.21, 25.00, 25.23, 29.95, 42.41, 46.18, 66.36, 115.10 (d, *J* = 21.3 Hz), 129.80 (d, *J* = 7.4 Hz), 132.58, 133.96, 145.79, 156.59, 161.36 (d, *J* = 243.0 Hz), 168.95; HRMS: calcd for C_24_H_31_N_4_O_1_F_1_ 411.2555 found 411.2564 [M + H]^+^.

#### (3-Aminopyrrolidin-1-yl)(3,4-bis(cyclohexylamino)phenyl)methanone (**50**)

General procedure D was followed using compound **33** (0.089 g, 0.184 mmol), to provide (3-aminopyrrolidin-1-yl)(3,4-bis(cyclohexylamino)phenyl)methanone **50** (0.090 g, 0.182 mmol, 94% yield). ^1^H NMR (400 MHz, MeOD) δ 1.13 – 1.60 (m, 10H), 1.67 (d, *J* = 10.5 Hz, 2H), 1.75 – 1.84 (m, 4H), 1.96 – 2.03 (m, 4H), 2.10 – 2.25 (m, 1H), 2.35 – 2.53 (m, 1H), 3.46 (br. s, 2H), 3.64 – 3.89 (m, 3H), 3.93 – 4.14 (m, 2H), 7.05 (d, *J* = 8.5 Hz, 1H), 7.31 – 7.52 (m, 2H); HRMS: calcd for C_23_H_36_N_4_O_1_ 385.2962, found 385.2966 [M + H]^+^.

#### (3-Aminopyrrolidin-1-yl)(3-(benzylamino)-4-(cyclohexylamino)phenyl)methanone (**51**)

General procedure D was followed using compound **34** (0.115 g, 0.233 mmol), to provide (3-aminopyrrolidin-1-yl)(3-(benzylamino)-4-(cyclohexylamino)phenyl)methanone **51** (0.111 g, 0.206mmol, 88% yield). ^1^H NMR (400 MHz, D_2_O) δ 1.11 – 1.22 (m, 3H), 1.33 (qd, *J* = 7.4, 12.6 Hz, 2H), 1.54 (d, *J* = 12.3 Hz, 1H), 1.66 – 1.75 (m, 2H), 1.85 – 1.91 (m, 2H), 2.07 – 2.29 (m, 1H), 2.95 – 3.14 (m, 2H), 3.20 – 3.39 (m, 1H), 3.42 – 3.57 (m, 2H), 3.73 (ddd, *J* = 6.6, 10.3, 13.2 Hz, 1H), 3.90 (t, *J* = 5.6 Hz, 1H), 4.39 (d, *J* = 9.6 Hz, 2H), 6.71 (dd, *J* = 1.8, 5.4 Hz, 1H), 6.81 – 6.89 (m, 1H), 7.16 (dd, *J* = 5.5, 8.2 Hz, 1H), 7.21 – 7.29 (m, 5H); HRMS: calcd for C_24_H_32_N_4_O_1_ 393.2649, found 393.2660 [M + H]^+^.

#### 4-(Cyclobutylamino)-3-nitrobenzoic acid (**53**)

General procedure J was followed using **52** (3.000 g, 14.88 mmol) and cyclobutanamine (3.000 mL, 35.20 mmol) to obtain 4-(cyclobutylamino)-3-nitrobenzoic acid **53** (2.900 g, 11.29 mmol, 96% yield). ^1^H NMR (400 MHz, DMSO-*d*_6_) δ 1.72 – 1.85 (m, 2H), 1.99 – 2.07 (m, 2H), 2.45 (dt, *J* = 3.5, 9.4 Hz, 2H), 4.20 (dd, *J* = 5.4, 10.7 Hz, 1H), 6.99 (d, *J* = 9.0 Hz, 1H), 7.96 (dd, *J* = 2.1, 9.0 Hz, 1H), 8.33 (d, *J* = 6.0 Hz, 1H), 8.60 (d, *J* = 2.0 Hz, 1H), 12.94 (br. s, 1H); MS (ESI) *m*/*z* 237.0 [M+H] ^+^.

#### 4-(Cyclopentylamino)-3-nitrobenzoic acid (**54**)

General procedure J was followed using **52** (2.000 g, 9.930 mmol) and cyclopentanamine **54** (4.900 mL, 49.70 mmol) to obtain 4-(cyclopentylamino)-3-nitrobenzoic acid **2** (2.320 g, 9.270 mmol, 93% yield). ^1^H NMR (400 MHz, DMSO-*d*_6_) δ 1.50 – 1.78 (m, 6H), 2.07 (dt, *J* = 12.0, 6.5 Hz, 2H), 4.08 (h, *J* = 6.3 Hz, 1H), 7.13 (d, *J* = 9.1 Hz, 1H), 7.95 (dd, *J* = 9.1, 2.1 Hz, 1H), 8.23 (d, *J* = 6.7 Hz, 1H), 8.58 (d, *J* = 2.0 Hz, 1H), 12.90 (s, 1H); MS (ESI) *m*/*z* 251 [M+H]^+^.

#### 4-(Cyclohexylamino)-3-nitrobenzoic acid (**55**)

General procedure J was followed using **52** (2.000 g, 9.930 mmol) and cyclohexanamine (3.410 mL, 29.90 mmol) to obtain 4-(cyclohexylamino)-3-nitrobenzoic acid **55** (2.530 g, 9.570 mmol, 96% yield). ^1^H NMR (400 MHz, DMSO-*d*_6_) δ 1.05 – 1.31 (m, 3H), 1.31 – 1.49 (m, 2H), 1.50 – 1.68 (m, 1H), 1.75 (td, *J* = 5.3, 10.1 Hz, 2H), 1.96 – 2.08 (m, 2H), 3.00 – 3.10 (m, 1H), 6.82 (d, *J* = 9.1 Hz, 1H), 7.97 (dd, *J* = 2.1, 9.1 Hz, 1H), 8.37 (d, *J* = 7.5 Hz, 1H), 8.84 (d, *J* = 2.0 Hz, 1H). MS (ESI) *m*/*z* 265.1 [M+H]^+^.

#### 4-(Cyclooctylamino)-3-nitrobenzoic acid (**56**)

General procedure J was followed using **52** (3.000 g, 14.88 mmol) and cyclooctanamine (6.120 mL, 44.70 mmol) to obtain 4-(cyclooctylamino)-3-nitrobenzoic acid **56** (2.600 g, 8.890 mmol, 60% yield). ^1^H NMR (400 MHz, MeOD) δ 1.59 – 1.88 (m, 12H), 1.91 – 2.09 (m, 2H), 3.91 (tt, *J* = 8.4, 3.8 Hz, 1H), 7.02 (d, *J* = 9.0 Hz, 1H), 8.05 (dd, *J* = 9.1, 2.1 Hz, 1H), 8.81 (d, *J* = 2.1 Hz, 1H); MS (ESI) *m*/*z* 293.0 [M+H]^+^.

#### 4-(Adamantan-2-yl)amino)-3-nitrobenzoic acid (**57**)

General procedure J was followed using **52** (2.200 g, 10.91 mmol) and adamantan-2-amine (4.950 g, 32.70 mmol) to obtain adamantan-2-yl)amino)-3-nitrobenzoic acid **57** (1.410 g, 4.430 mmol, 40% yield). ^1^H NMR (400 MHz, DMSO-*d*_6_) δ 1.66 (d, *J* = 12.7 Hz, 2H), 1.71 – 1.76 (m, 2H), 1.78 – 1.95 (m, 8H), 1.98 – 2.10 (m, 2H), 3.88 – 4.09 (m, 1H), 7.17 (d, *J* = 9.2 Hz, 1H), 7.96 (dd, *J* = 2.1, 9.1 Hz, 1H), 8.63 (d, *J* = 2.0 Hz, 1H), 8.78 (d, *J* = 7.6 Hz, 1H), 12.96 (s, 1H); MS (ESI) *m*/*z* 316.9 [M+H]^+^.

#### 4-(Cyclobutylamino)-3-nitrobenzoyl chloride (**58**)

General procedure K was followed using compound **53** (2.800 g, 9.263 mmol) to obtain 4-(cyclobutylamino)-3-nitrobenzoyl chloride **58** (3.020 g, 11.85 mmol) as crude mixture. MS (ESI) *m*/*z* 251.1 [M-Cl+OCH_3_]^+^.

#### 4-(Cyclopentylamino)-3-nitrobenzoyl chloride (**59**)

General procedure K was followed using compound **54** (2.318 g, 11.77 mmol) to obtain 4-(cyclohexylamino)-3-nitrobenzoic acid **59** (2.489 g, 9.623 mmol) as crude mixture. MS (ESI) *m*/*z* 265 [M-Cl+OCH_3_]^+^.

#### 4-(Cyclohexylamino)-3-nitrobenzoyl chloride (**60**)

General procedure K was followed using compound **55** (2.520 g, 9.540 mmo) to obtain 4-(Cyclohexylamino)-3-nitrobenzoyl chloride **60** (2.700 g, 9.540 mmol) as crude mixture. MS (ESI) *m*/*z* 279 [M-Cl+OCH_3_]^+^.

#### 4-(Cyclooctylamino)-3-nitrobenzoyl chloride (**61**)

General procedure K was followed using compound **56** (2.580 g, 8.830 mmol) to obtain 4-(cyclooctylamino)-3-nitrobenzoyl chloride **61** (2.740 g, 8.830 mmol) as crude mixture. MS (ESI) *m*/*z* 307.0 [M-Cl+OCH_3_]^+^.

#### 4-((Adamantan-2-yl)amino)-3-nitrobenzoyl chloride (**62**)

General procedure K was followed using compound **57** (1.400 g, 3.790 mmol) to obtain 4-((Adamantan-2-yl)amino)-3-nitrobenzoyl chloride **62** (1.482 g, 4.430 mmol) as crude mixture. MS (ESI) *m*/*z* 331.2 [M-Cl+OCH_3_]^+^.

#### *N*-Cyclobutyl-4-(5-methyloxazol-2-yl)-2-nitroaniline (**68**)

General procedure L and M were followed using compound **58** (2.500 g, 9.820 mmol) to obtain the crude compound **63** (2.680 g, 9.810 mmol) and then *N*-cyclopentyl-4-(5-methyloxazol-2-yl)-2-nitroaniline **68** (0.445 g, 1.628 mmol, 16% yield). ^1^H NMR (400 MHz, DMSO-*d*_6_) δ 1.78 (p, *J* = 8.8 Hz, 2H), 2.05 (p, *J* = 9.7 Hz, 2H), 2.36 (s, 3H), 2.40 – 2.47 (m, 2H), 4.18 (q, *J* = 7.5 Hz, 1H), 6.93 (s, 1H), 7.05 (d, *J* = 9.1 Hz, 1H), 7.98 (d, *J* = 9.0 Hz, 1H), 8.25 (d, *J* = 5.9 Hz, 1H), 8.49 (t, *J* = 1.8 Hz, 1H); MS (ESI) *m*/*z* 274.1 [M+H]^+^.

#### *N*-Cyclopentyl-4-(5-methyloxazol-2-yl)-2-nitroaniline (**69**)

General procedure L and M were followed using compoun **59** (2.480 g, 9.230 mmol) to obtain the crude compound **64** (2.650 g, 9.230 mmol) and then *N*-cyclopentyl-4-(5-methyloxazol-2-yl)-2-nitroaniline **69** (0.805 g, 2.800 mmol, 30% yield). ^1^H NMR (400 MHz, Acetone-*d*_6_) δ 1.61 – 1.77 (m, 4H), 1.77 – 1.87 (m, 2H), 2.13 – 2.26 (m, 2H), 2.39 (s, 3H), 4.18 (dq, *J* = 5.4, 7.1 Hz, 1H), 6.84 (q, *J* = 1.3 Hz, 1H), 7.22 (d, *J* = 9.1 Hz, 1H), 8.05 (dd, *J* = 0.6, 9.1 Hz, 1H), 8.26 (br. s, *J* = 6.9 Hz, 1H), 8.64 (d, *J* = 2.2 Hz, 1H); MS (ESI) *m*/*z* 288 [M+H]^+^.

#### *N*-Cyclohexyl-4-(5-methyloxazol-2-yl)-2-nitroaniline (**70**)

General procedure L and M were followed using compound **60** (2.700 g, 9.550 mmol) to obtain the crude compound **65** (2.880 g, 9.550 mmol) and then *N-*cyclohexyl-4-(5-methyloxazol-2-yl)-2-nitroaniline **70** (0.900 g, 2.990 mmol, 31% yield). ^1^H NMR (400 MHz, Acetone-*d*_6_) δ 1.26 - 1.58 (m, 5H), 1.62 - 1.71 (m, 1H), 1.75 - 1.86 (m, 2H), 2.08 - 2.13 (m, 2H), 2.39 (d, *J* = 1.3 Hz, 3H), 3.65 - 3.79 (m, 1H), 6.83 (q, *J* = 1.2 Hz, 1H), 7.19 (dd, *J* = 0.6, 9.1 Hz, 1H), 8.00 (ddd, *J* = 0.7, 2.2, 9.0 Hz, 1H), 8.26 (d, *J* = 7.7 Hz, 1H), 8.62 (d, *J* = 2.1 Hz, 1H); MS (ESI) *m*/*z* 302 [M+H]^+^.

#### *N*-(4-(5-Methyloxazol-2-yl)-2-nitrophenyl)cyclooctanamine (**71**)

General procedure L and M were followed using compound **61** (2.740 g, 9.230 mmol) to obtain the crude compound **66** (2.910 g, 9.230 mmol) and then *N*-(4-(5-methyloxazol-2-yl)-2-nitrophenyl)cyclooctanamine **71** (0.339g, 1.029 mmol, 13% yield). ^1^H NMR (400 MHz, CDCl_3_) δ 1.50 – 1.73 (m, 11H), 1.71 – 1.88 (m, 5H), 1.87 – 2.03 (m, 3H), 2.38 (d, *J* = 1.3 Hz, 3H), 3.78 (dt, *J* = 4.1, 8.2 Hz, 1H), 6.79 (q, *J* = 1.2 Hz, 1H), 6.87 (d, *J* = 9.2 Hz, 1H), 8.05 (dd, *J* = 2.1, 9.1 Hz, 1H), 8.39 (d, *J* = 7.5 Hz, 1H), 8.77 (d, *J* = 2.1 Hz, 1H); MS (ESI) *m*/*z* 330.2 [M+H]^+^.

#### *N*-(4-(5-Methyloxazol-2-yl)-2-nitrophenyl)adamantan-2-amine (**72**)

General procedure L and M were followed using compound **62** (1.270 g, 3.790 mmol) to obtain the crude compound **67** (1.341 g, 3.790 mmol) and then *N*-(4-(5-methyloxazol-2-yl)-2-nitrophenyl)adamantan-2-amine **72** (0.845 g, 2.391 mmol, 60% yield). ^1^H NMR (400 MHz, DMSO-*d*_*6*_) δ 1.67 (d, *J* = 13.1 Hz, 2H), 1.75 (s, 2H), 1.80 – 1.98 (m, 8H), 2.04 (s, 2H), 2.38 (d, *J* = 1.2 Hz, 3H), 4.01 (dt, *J* = 3.1, 6.7 Hz, 1H), 6.95 (q, *J* = 1.1 Hz, 1H), 7.26 (d, *J* = 9.2 Hz, 1H), 8.00 (dd, *J* = 2.1, 9.1 Hz, 1H), 8.55 (d, *J* = 2.1 Hz, 1H), 8.75 (d, *J* = 7.6 Hz, 1H); MS (ESI) *m*/*z* 354.2 [M+H]^+^

#### *N*1-Cyclobutyl-4-(5-methyloxazol-2-yl)benzene-1,2-diamine (**73**)

General procedure C was followed using compound **68** (0.430 g, 1.573 mmol) to obtain *N*1-cyclobutyl-4-(5-methyloxazol-2-yl)benzene-1,2-diamine **73** (0.313 g, 1.286 mmol, 82% yield). ^1^H NMR (400 MHz, MeOD) δ 1.76 – 2.01 (m, 4H), 2.30 – 2.40 (m, 3H), 2.45 (td, *J* = 7.7, 4.4 Hz, 2H), 3.98 (p, *J* = 7.5 Hz, 1H), 6.51 (d, *J* = 8.1 Hz, 1H), 6.70 – 6.80 (m, 1H), 7.22 – 7.35 (m, 2H); ^13^C NMR (101 MHz, MeOD) δ 10.68, 16.17, 31.13, 31.76, 50.11, 111.75, 114.17, 117.36, 119.55, 123.63, 135.29, 139.86, 149.32, 163.78; HRMS (ESI) *m*/*z* [M+H]^+^ calcd for C_14_H_17_N_3_O 244.1444, found 244.1453.

#### *N*1-Cyclopentyl-4-(5-methyloxazol-2-yl)benzene-1,2-diamine (**74**)

General procedure C was followed using compound **69** (0.800 g, 2.780 mmol) to obtain *N*1-cyclopentyl-4-(5-methyloxazol-2-yl)benzene-1,2-diamine **74** (0.440 g, 1.711 mmol, 61% yield). ^1^H NMR (400 MHz, DMSO-*d*_6_) δ 1.43 – 1.61 (m, 4H), 1.63 – 1.74 (m, 2H), 1.90 – 2.01 (m, 2H), 2.30 (d, *J* = 1.3 Hz, 3H), 3.75 (h, *J* = 6.0 Hz, 1H), 4.72 – 4.78 (m, 1H), 4.80 (s, 2H), 6.46 (d, *J* = 8.2 Hz, 1H), 6.77 (q, *J* = 1.2 Hz, 1H), 7.09 (dd, *J* = 2.0, 8.2 Hz, 1H), 7.12 (d, *J* = 2.0 Hz, 1H); ^13^C NMR (101 MHz, DMSO-*d*_6_) δ 11.13, 24.35, 33.09, 33.13, 54.10, 54.19, 110.04, 111.20, 115.81, 115.84, 116.36, 123.95, 135.41, 137.88, 147.36, 161.70; HRMS (ESI) *m*/*z* [M+H]^+^ calcd for C_15_H_19_N_3_O 258.1601, found 258.1602.

#### *N*1-Cyclohexyl-4-(5-methyloxazol-2-yl)benzene-1,2-diamine (**75**)

General procedure C was followed using compound **70** (0.900 g, 2.990 mmol) to obtain *N1*-cyclohexyl-4-(5-methyloxazol-2-yl)benzene-1,2-diamine **75** (0.677 g, 2.495 mmol, 84% yield). ^1^H NMR (400 MHz, Acetone-*d*_*6*_) δ 1.13 - 1.31 (m, 3H), 1.32 - 1.48 (m, 2H), 1.60 - 1.68 (m, 1H), 1.71 - 1.79 (m, 2H), 2.07 - 2.11 (m, 2H), 2.34 (d, *J* = 1.3 Hz, 3H), 3.36 (dtd, *J* = 3.6, 6.9, 10.5 Hz, 1H), 4.23 (d, *J* = 7.5 Hz, 1H), 4.31 (s, 2H), 6.65 (d, *J* = 8.2 Hz, 1H), 6.77 (q, *J* = 1.2 Hz, 1H), 7.37 - 7.43 (m, 2H); ^13^C NMR (101 MHz, Acetone-*d*_*6*_) δ 10.15, 24.94, 25.92, 33.11, 51.27, 110.03, 113.06, 116.57, 117.78, 123.63, 134.68, 138.23, 147.17, 161.78; HRMS (ESI) *m*/*z* [M+H]^+^ calcd for C_16_H_21_N_3_O 272.1757, found 272.1755.

#### *N*1-Cyclooctyl-4-(5-methyloxazol-2-yl)benzene-1,2-diamine (**76**)

General procedure C was followed using compound **71** (0.330 g, 1.000 mmol) to obtain *N*1-cyclooctyl-4-(5-methyloxazol-2-yl)benzene-1,2-diamine **76** (0.168 g, 0.561 mmol, 56% yield). ^1^H NMR (400 MHz, MeOD) δ 1.53 – 1.75 (m, 10H), 1.80 (q, *J* = 9.4 Hz, 2H), 1.92 (tt, *J* = 2.5, 10.7 Hz, 2H), 2.36 (d, *J* = 1.2 Hz, 3H), 3.58 (tt, *J* = 3.5, 8.5 Hz, 1H), 6.54 (d, *J* = 8.4 Hz, 1H), 6.76 (d, *J* = 1.4 Hz, 1H), 7.28 (d, *J* = 2.0 Hz, 1H), 7.33 (dd, *J* = 2.1, 8.3 Hz, 1H); ^13^C NMR (101 MHz, MeOD) δ 9.30, 23.88, 25.70, 26.82, 32.17, 52.25, 110.23, 113.21, 115.25, 118.42, 122.21, 133.79, 138.69, 147.83, 162.42; HRMS (ESI) *m*/*z* [M+H]^+^ calcd for C_18_H_25_N_3_O 300.2070, found 300.2069.

#### *N*1-(Adamantan-2-yl)-4-(5-methyloxazol-2-yl)benzene-1,2-diamine (**77**)

General procedure C was followed using compound **72** (0.834 g, 2.360 mmol) to obtain *N*1-(adamantan-2-yl)-4-(5-methyloxazol-2-yl)benzene-1,2-diamine **77** (0.720 g, 2.226 mmol, 94% yield). ^1^H NMR (400 MHz, DMSO-*d*_*6*_) δ 1.48 (d, *J* = 12.5 Hz, 2H), 1.70 (d, *J* = 3.0 Hz, 2H), 1.74 – 1.80 (m, 1H), 1.80 – 1.89 (m, 5H), 1.92 – 2.01 (m, 2H), 2.07 (d, *J* = 13.0 Hz, 2H), 2.29 (d, *J* = 1.2 Hz, 3H), 3.54 (dd, *J* = 3.0, 6.1 Hz, 1H), 4.52 (d, *J* = 6.4 Hz, 1H), 4.88 (s, 2H), 6.42 (d, *J* = 8.4 Hz, 1H), 6.77 (q, *J* = 1.2 Hz, 1H), 7.08 (dd, *J* = 2.1, 8.3 Hz, 1H), 7.17 (d, *J* = 2.0 Hz, 1H); ^13^C NMR (101 MHz, DMSO-*d*_*6*_) δ 11.13, 27.30, 27.47, 31.77, 37.26, 37.72, 56.46, 109.87, 112.13, 115.87, 116.70, 123.97, 135.58, 137.61, 147.39, 161.64; HRMS (ESI) *m*/*z* [M+H]^+^ calcd for C_20_H_25_N_3_O 324.2070, found 324.2084.

#### *N*2-Benzyl-*N*1-cyclobutyl-4-(5-methyloxazol-2-yl)benzene-1,2-diamine (**78**)

General procedure N was followed using compound **73** (0.100 g, 0.411 mmol) to obtain *N*2-benzyl-*N*1-cyclobutyl-4-(5-methyloxazol-2-yl)benzene-1,2-diamine **78** (0.015 g, 0.045 mmol, 15% yield). ^1^H NMR (400 MHz, MeOD) δ 1.77 – 1.87 (m, 2H), 1.88 – 1.99 (m, 2H), 2.33 (s, 3H), 2.40 – 2.50 (m, 2H), 3.92 – 4.03 (m, 1H), 4.38 (s, 2H), 6.52 (d, *J* = 8.2 Hz, 1H), 6.72 (t, *J* = 1.1 Hz, 1H), 7.14 (d, *J* = 1.9 Hz, 1H), 7.19 – 7.28 (m, 2H), 7.31 (t, *J* = 7.6 Hz, 2H), 7.41 (d, *J* = 7.4 Hz, 2H); ^13^C NMR (101 MHz, MeOD) δ 9.30, 14.83, 30.33, 47.77, 48.87, 108.03, 110.00, 116.22, 117.01, 122.20, 126.62, 127.40, 128.05, 135.95, 138.12, 139.54, 147.86, 162.63; HRMS (ESI) *m*/*z* [M+H]^+^ calcd for C_21_H_23_N_3_O 334.1914, found 334.1906.

#### *N*2-Benzyl-*N*1-cyclopentyl-4-(5-methyloxazol-2-yl)benzene-1,2-diamine (**79**)

General procedure N was followed using compound **74** (0.120 g, 0.480 mmol) to obtain *N*2-benzyl-*N*1-cyclopentyl-4-(5-methyloxazol-2-yl)benzene-1,2-diamine **79** (0.040 g, 0.115 mmol, 25% yield).^1^H NMR (400 MHz, DMSO-*d*_6_) δ 1.46 – 1.62 (m, 4H), 1.63 – 1.76 (m, 2H), 1.92 – 2.04 (m, 2H), 2.28 (d, *J* = 1.3 Hz, 3H), 3.79 (s, 1H), 4.33 (s, 2H), 5.03 (s, 1H), 5.53 (s, 1H), 6.52 (d, *J* = 8.3 Hz, 1H), 6.74 (d, *J* = 1.4 Hz, 1H), 6.91 (d, *J* = 1.9 Hz, 1H), 7.12 (dd, *J* = 1.9, 8.2 Hz, 1H), 7.20 – 7.26 (m, 1H), 7.30 – 7.41 (m, 4H); ^13^C NMR (101 MHz, DMSO-*d*_6_) δ 11.12, 24.39, 33.08, 47.41, 54.33, 106.92, 109.72, 115.92, 116.28, 123.89, 127.21, 127.74, 128.79, 135.68, 138.13, 140.32, 147.46, 161.69; HRMS (ESI) *m*/*z* [M+H]^+^ calcd for C_22_H_25_N_3_O 348.2070, found 348.2081.

#### *N*2-Benzyl-*N*1-cyclohexyl-4-(5-methyloxazol-2-yl)benzene-1,2-diamine (**80**)

General procedure N was followed using compound **75** (0.200 g, 0.720 mmol) to obtain *N*2-benzyl-*N*1-cyclohexyl-4-(5-methyloxazol-2-yl)benzene-1,2-diamine **80** (0.040 g, 0.115 mmol, 16% yield). ^1^H NMR (400 MHz, Acetone-*d*_6_) δ 1.20 - 1.32 (m, 4H), 1.37 - 1.51 (m, 2H), 1.62 - 1.70 (m, 1H), 1.73 - 1.82 (m, 2H), 2.10 - 2.13 (m, 1H), 2.33 (d, *J = 1*.3 Hz, 3H), 3.31 - 3.46 (m, 1H), 4.39 (d, *J* = 5.6 Hz, 2H), 4.42 (d, *J* = 7.4 Hz, 1H), 4.63 (t, *J* = 5.7 Hz, 1H), 6.71 (d, *J* = 8.3 Hz, 1H), 6.73 (q, *J = 1*.2 Hz, 1H), 7.23 - 7.28 (m, 1H), 7.29 (d, *J* = 1.9 Hz, 1H), 7.31 - 7.38 (m, 3H), 7.42 - 7.47 (m, 2H); 13C NMR (101 MHz, Acetone-*d*_6_) δ 10.04, 24.92, 25.88, 33.08, 48.09, 51.42, 108.94, 109.91, 116.95, 117.26, 123.59, 126.86, 127.68, 128.32, 136.03, 138.21, 139.88, 147.19, 161.79; HRMS (ESI) *m*/*z* [M+H]^+^ calcd for C_23_H_27_N_3_O 362.2227, found 362.2209.

#### *N*2-Benzyl-*N*1-cyclooctyl-4-(5-methyloxazol-2-yl)benzene-1,2-diamine (**81**)

General procedure N was followed using compound **76** (0.075 g, 0.250 mmol) to obtain *N*2-benzyl-*N*1-cyclooctyl-4-(5-methyloxazol-2-yl)benzene-1,2-diamine **81** (0.041 g, 0.105 mmol, 42% yield). ^1^H NMR (400 MHz, MeOD) δ 1.53 – 1.76 (m, 10H), 1.73 – 1.83 (m, 2H), 1.84 – 1.93 (m, 2H), 2.32 (s, 3H), 3.57 (tt, *J* = 3.5, 8.3 Hz, 1H), 4.36 (s, 2H), 6.56 (d, *J* = 8.3 Hz, 1H), 6.72 (t, *J* = 1.2 Hz, 1H), 7.16 (d, *J* = 1.9 Hz, 1H), 7.18 – 7.26 (m, 1H), 7.26 – 7.34 (m, 3H), 7.40 (d, *J* = 7.2 Hz, 2H); ^13^C NMR (101 MHz, MeOD) δ 10.70, 25.29, 27.10, 28.21, 33.52, 49.30, 53.74, 110.38, 111.46, 116.93, 118.84, 123.58, 127.97, 128.74, 129.42, 137.30, 139.94, 141.04, 149.17, 164.04; HRMS (ESI) *m*/*z* [M+H]^+^ calcd for C_25_H_31_N_3_O 390.2540, found 390.2556.

#### *N*1-(Adamantan-2-yl)-*N*2-benzyl-4-(5-methyloxazol-2-yl)benzene-1,2-diamine (**82**)

General procedure N was followed using compound **77** (0.100 g, 0.309 mmol) to obtain *N*1-(adamantan-2-yl)-*N*2-benzyl-4-(5-methyloxazol-2-yl)benzene-1,2-diamine **82** (0.048 g, 0.116 mmol, 38% yield). ^1^H NMR (400 MHz, MeOD) δ 1.54 – 1.67 (m, 2H), 1.74 – 1.85 (m, 3H), 1.85 – 1.99 (m, 5H), 2.07 (t, *J* = 10.4 Hz, 4H), 2.32 (d, *J* = 1.2 Hz, 3H), 3.31 (p, *J* = 1.6 Hz, 1H), 4.37 (s, 2H), 6.60 (d, *J* = 8.3 Hz, 1H), 6.72 (d, *J* = 1.5 Hz, 1H), 7.18 – 7.27 (m, 2H), 7.26 – 7.36 (m, 3H), 7.41 (d, *J* = 7.4 Hz, 2H); ^13^C NMR (101 MHz, MeOD) δ 9.30, 27.50, 27.65, 31.24, 31.59, 37.01, 37.44, 48.19, 56.64, 109.97, 110.60, 115.57, 118.23, 122.21, 126.56, 127.23, 128.04, 135.87, 139.54, 139.83, 147.82, 162.55; HRMS (ESI) *m*/*z* [M+H]^+^ calcd for C_27_H_31_N_3_O 414.2540, found 414.2527.

#### *N*1-Cyclopentyl-*N*2-(4-fluorobenzyl)-4-(5-methyloxazol-2-yl)benzene-1,2-diamine (**83**)

General procedure N was followed using compound **74** (0.170 g, 0.650 mmol), to obtain *N*1-cyclopentyl-*N*2-(4-fluorobenzyl)-4-(5-methyloxazol-2-yl)benzene-1,2-diamine **83** (0.020 g, 0.048 mmol, 8% yield). ^1^H NMR (400 MHz, DMSO-*d*_6_) δ 1.45 – 1.63 (m, 4H), 1.63 – 1.75 (m, 2H), 1.97 (dt, *J* = 6.7, 11.8 Hz, 2H), 2.28 (d, *J* = 1.3 Hz, 3H), 3.80 (p, *J* = 5.9 Hz, 1H), 4.31 (d, *J* = 5.4 Hz, 2H), 5.01 (d, *J* = 5.9 Hz, 1H), 5.52 (t, *J* = 5.6 Hz, 1H), 6.52 (d, *J* = 8.3 Hz, 1H), 6.74 (q, *J* = 1.1 Hz, 1H), 6.88 (d, *J* = 1.9 Hz, 1H), 7.10 – 7.20 (m, 3H), 7.36 – 7.44 (m, 2H); ^**1**3^C NMR (101 MHz, DMSO-*d*_6_) δ 11.13, 24.40, 33.09, 46.66, 54.32, 106.95, 109.72, 115.51 (d, *J* = 21.2 Hz), 115.91, 116.38, 123.92, 129.59 (d, *J* = 8.0 Hz), 135.51, 136.40 (d, *J* = 2.9 Hz), 138.18, 147.47, 161.64 (d, *J* = 241.5 Hz), 161.67; HRMS (ESI) *m*/*z* [M+H]^+^ calcd for C_22_H_24_FN_3_O 366.1976, found 366.1972.

#### *N*1-Cyclohexyl-*N*2-(4-fluorobenzyl)-4-(5-methyloxazol-2-yl)benzene-1,2-diamine (**84**)

Procedure N was followed, using compound **75** (0.080 g, 0.290 mmol) to obtain *N*1-cyclohexyl-*N*2-(4-fluorobenzyl)-4-(5-methyloxazol-2-yl)benzene-1,2-diamine **84** (0.0503 g, 0.133 mmol, 46% yield). ^1^H NMR (400 MHz, DMSO-*d*_6_) δ 1.22 (p, *J* = 12.2 Hz, 3H), 1.30 – 1.40 (m, 2H), 1.63 (d, *J* = 12.6 Hz, 1H), 1.74 (d, *J* = 12.6 Hz, 2H), 2.00 (d, *J* = 12.3 Hz, 2H), 2.29 (s, 3H), 3.25 – 3.32 (m, 1H), 4.31 (d, *J* = 5.4 Hz, 2H), 4.89 (d, *J* = 7.3 Hz, 1H), 5.49 (t, *J* = 5.5 Hz, 1H), 6.53 (d, *J* = 8.3 Hz, 1H), 6.75 (s, 1H), 6.91 (d, *J* = 2.0 Hz, 1H), 7.10 – 7.21 (m, 3H), 7.41 (t, *J* = 5.6 Hz, 2H); ^13^C NMR (101 MHz, DMSO-*d*_6_) δ 11.13, 25.20, 26.11, 33.13, 46.74, 51.35, 107.43, 109.23, 115.50 (d, *J* = 22.4 Hz), 115.67, 116.54, 123.89, 129.63 (d, *J* = 8.7 Hz), 135.36, 136.39 (d, *J* = 3.4 Hz), 137.60, 147.41, 160.42 (d, *J* = 244.3 Hz), 161.64; HRMS (ESI) *m*/*z* [M+H]^+^ calcd for C_23_H_26_FN_3_O 380.2133, found 380.2133.

#### *N*1-Cyclopentyl-4-(5-methyloxazol-2-yl)-*N*2-(pyridin-4-ylmethyl)benzene-1,2-diamine (**85**)

General procedure N was followed using compound **74** (0.130 g, 0.500 mmol) to obtain *N*1-cyclopentyl-4-(5-methyloxazol-2-yl)-*N*2-(pyridin-4-ylmethyl)benzene-1,2-diamine **85** (0.030 g, 0.092 mmol, 19% yield). ^1^H NMR (400 MHz, DMSO-*d*_6_) δ 1.49 – 1.63 (m, 4H), 1.68 – 1.75 (m, 2H), 2.01 (q, *J* = 5.6 Hz, 2H), 2.20 – 2.32 (m, 3H), 3.76 – 3.87 (m, 1H), 4.40 (d, *J* = 5.2 Hz, 2H), 5.02 (d, *J* = 5.7 Hz, 1H), 5.68 (t, *J* = 5.7 Hz, 1H), 6.55 (d, *J* = 8.3 Hz, 1H), 6.76 (dd, *J* = 1.5, 23.3 Hz, 2H), 7.14 (dd, *J* = 1.9, 8.2 Hz, 1H), 7.38 (d, *J* = 5.9 Hz, 2H), 8.51 (d, *J* = 9.6 Hz, 2H); ^13^C NMR (101 MHz, DMSO-*d*_6_) δ 11.11, 24.39, 33.11, 40.37, 54.35, 106.94, 115.93, 116.61, 117.47, 122.91, 123.90, 124.42, 135.18, 138.24, 147.52, 149.33, 161.54; HRMS (ESI) *m*/*z* [M+H]^+^ calcd for C_21_H_24_N_4_O 349.2023, found 349.2025.

#### *N*1-Cyclohexyl-4-(5-methyloxazol-2-yl)-*N*2-(pyridin-4-ylmethyl)benzene-1,2-diamine (**86**)

General procedure N was followed using compound **75** (0200 g, 0.737 mmol) to obtain *N*1-cyclohexyl-4-(5-methyloxazol-2-yl)-*N*2-(pyridin-4-ylmethyl)benzene-1,2-diamine **86** (0.110 g, 0.303 mmol, 41% yield). ^1^H NMR (400 MHz, DMSO-*d*_*6*_) δ 1.17 - 1.31 (m, 3H), 1.32 - 1.45 (m, 2H), 1.59 - 1.70 (m, 1H), 1.77 (dt, *J* = 3.7, 12.9 Hz, 2H), 2.03 (d, *J* = 12.2 Hz, 2H), 2.64 (s, 3H), 3.35 - 3.39 (m, 1H), 4.42 (d, *J* = 5.6 Hz, 2H), 5.04 (d, *J* = 7.2 Hz, 1H), 5.77 (t, *J* = 5.7 Hz, 1H), 6.58 (d, *J* = 8.3 Hz, 1H), 6.78 (d, *J* = 2.1 Hz, 1H), 7.07 (dd, *J* = 8.2, 2.0 Hz, 1H), 7.38 (d, *J* = 7.6 Hz, 2H), 8.51 (d, *J* = 6.4 Hz, 2H); ^13^C NMR (101 MHz, MeOD) δ 9.26, 24.86, 25.74, 32.91, 46.30, 51.48, 108.54, 109.92, 115.52, 117.62, 122.18, 122.74, 134.99, 138.51, 147.84, 148.45, 151.17, 162.45; HRMS (ESI) *m*/*z* [M+H]^+^ calcd for C_22_H_26_N_4_O 363.2179, found 363.2196.

#### *N*-Cyclobutyl-2-nitro-4-(oxazol-5-yl)aniline (**89**)

General procedure J was followed using **88** (0.330 g, 1.469 mmol) and cyclobutanamine (0.104 g, 1.469 mmol) to obtain *N*-cyclobutyl-2-nitro-4-(oxazol-5-yl)aniline **89** (0.314 g, 1.211 mmol, 82% yield). ^1^H NMR (400 MHz, CDCl_3_) δ 1.83 – 2.01 (m, 2H), 2.01 – 2.13 (m, 2H), 2.50 – 2.60 (m, 2H), 4.06 – 4.16 (m, 1H), 6.81 (d, *J* = 9.0 Hz, 1H), 7.28 (s, 1H), 7.68 (dd, *J* = 2.1, 9.0 Hz, 1H), 7.89 (s, 1H), 8.47 (d, *J* = 2.1 Hz, 1H); t_R_ 1.91 min. MS (ESI) *m*/*z* 260.1 [M+H]^+^ (100%).

#### *N*-Cyclopentyl-2-nitro-4-(oxazol-5-yl)aniline (**90**)

General procedure J was followed using **88** (0.330 g, 1.469 mmol) and cyclopentanamine (0.375 g, 4.410 mmol) to obtain *N*-cyclopentyl-2-nitro-4-(oxazol-5-yl)aniline **90** (0.390 g, 1.427 mmol, 97% yield). ^1^H NMR (400 MHz, CDCl_3_) δ 1.54 – 1.75 (m, 4H), 1.75 – 1.87 (m, 2H), 2.06 – 2.20 (m, 2H), 4.01 (ddd, *J* = 5.2, 6.8, 10.0 Hz, 1H), 6.96 (d, *J* = 9.1 Hz, 1H), 7.24 (s, 1H), 7.67 (dd, *J* = 0.7, 9.0 Hz, 1H), 7.88 (s, 1H), 8.45 (d, *J* = 2.2 Hz, 1H); t_R_ 2.02 min. MS (ESI) *m*/*z* 274.1 [M+H]^+^ (100%).

#### *N*-Cyclohexyl-2-nitro-4-(oxazol-5-yl)aniline (**91**)

General procedure J was followed using **88** (1.000 g, 4.450 mmol) and cyclohexanamine (3.060 mL, 26.70 mmol) to obtain *N-*cyclohexyl-2-nitro-4-(oxazol-5-yl)aniline **91** (1.200 g, 4.180 mmol, 94% yield). ^1^H NMR (400 MHz, DMSO-*d*_6_) δ 1.02 – 1.86 (m, 8H), 1.89 – 2.04 (m, 2H), 3.65 – 3.77 (m, 1H), 7.25 (d, *J* = 9.2 Hz, 1H), 7.64 (s, 1H), 7.86 (dd, *J* = 2.3, 9.1 Hz, 1H), 8.16 (d, *J* = 7.8 Hz, 1H), 8.32 (d, *J* = 2.2 Hz, 1H), 8.40 (s, 1H); t_R_ 2.12 min. MS (ESI) *m*/*z* 288.1 [M+H]^+^ (100%).

#### *N*-(2-Nitro-4-(oxazol-5-yl)phenyl)cyclooctanamine (**92**)

General procedure J was followed using **88** (0.330 g, 1.469 mmol) and cyclooctanamine (0.561 g, 4.410 mmol) to obtain *N*-(2-nitro-4-(oxazol-5-yl)phenyl)cyclooctanamine **92** (0.439 g, 1.392 mmol, 95% yield). ^1^H NMR (400 MHz, CDCl_3_) δ 1.43 – 1.91 (m, 12H), 1.92 – 2.04 (m, 2H), 3.77 (ddt, *J* = 4.1, 7.8, 12.0 Hz, 1H), 6.90 (d, *J* = 9.1 Hz, 1H), 7.29 (s, 1H), 7.69 (dd, *J* = 2.1, 9.0 Hz, 1H), 7.89 (s, 1H), 8.49 (d, *J* = 2.2 Hz, 1H); t_R_ 2.39 min. MS (ESI) *m*/*z* 316.2 [M+H]^+^ (100%).

#### *N*-(2-Nitro-4-(oxazol-5-yl)phenyl)adamantan-2-amine (**93**)

General procedure J was followed using **88** (0.240 g, 1.069 mmol) and adamantan-2-amine hydrochloride (0.602 g, 3.210 mmol) to obtain *N*-(2-nitro-4-(oxazol-5-yl)phenyl)adamantan-2-amine **93** (0.293 g, 0.863 mmol, 81% yield). ^1^H NMR (400 MHz, CDCl_3_) δ 1.63 – 2.02 (m, 12H), 2.02 – 2.17 (m, 2H), 3.76 – 3.85 (m, 1H), 6.90 (d, *J* = 9.0 Hz, 1H), 7.23 (s, 1H), 7.63 (d, *J* = 9.2 Hz, 1H), 7.87 (s, 1H), 8.45 (s, 1H), 8.79 (d, *J* = 7.4 Hz, 1H); t_R_ 2.79 min MS (ESI) *m*/*z* 340.2 [M+H] ^+^ (100%).

#### *N*1-Cyclobutyl-4-(oxazol-5-yl)benzene-1,2-diamine (**94**)

General procedure C was followed using compound **89** (0.314 g, 1.211 mmol) to obtain *N*1-cyclobutyl-4-(oxazol-5-yl)benzene-1,2-diamine **94** (0.050 g, 0.218 mmol, 18% yield). ^1^H NMR (400 MHz, MeOD) δ 1.78 – 2.00 (m, 4H), 2.45 (ddt, *J* = 9.7, 6.2, 2.7 Hz, 2H), 3.97 (p, *J* = 7.4 Hz, 1H), 6.52 (d, *J* = 8.0 Hz, 1H), 7.02 (d, *J* = 2.0 Hz, 1H), 7.05 (s, 1H), 7.18 (s, 1H), 8.10 (s, 1H); ^13^C NMR (101 MHz, MeOD) δ 16.16, 31.81, 50.28, 112.48, 112.89, 117.57, 118.33, 118.62, 135.76, 138.30, 151.40, 154.66; t_R_ 1.40 min, 100%; HRMS (ESI) *m*/*z* [M+H]^+^ calcd for C_13_H_15_N_3_O 230.1288 found 230.1289.

#### *N*1-Cyclopentyl-4-(oxazol-5-yl)benzene-1,2-diamine (**95**)

General procedure C was followed using compound **90** (0.390 g, 1.427 mmol), to obtain *N*1-cyclopentyl-4-(oxazol-5-yl)benzene-1,2-diamine **95** (0.031 g, 0.127 mmol, 9% yield). ^1^H NMR (400 MHz, MeOD) δ 1.50 – 1.70 (m, 4H), 1.70 – 1.84 (m, 2H), 2.00 – 2.11 (m, 2H), 3.84 (tq, *J* = 11.9, 6.0 Hz, 1H), 6.65 (d, *J* = 7.9 Hz, 1H), 7.03 – 7.08 (m, 2H), 7.17 (s, 1H), 8.10 (s, 1H); ^13^C NMR (101 MHz, MeOD) δ 25.22, 34.27, 55.72, 112.65, 113.01, 117.69, 117.89, 118.53, 135.71, 139.29, 151.36, 154.70; t_R_ 1.43 min, 100%; HRMS (ESI) *m*/*z* [M+H]^+^ calcd for C_14_H_17_N_3_O 244.1444 found 244.1437.

#### *N*1-Cyclohexyl-4-(oxazol-5-yl)benzene-1,2-diamine (**96**)

General procedure C was followed using compound **91** (2.320 g, 8.080 mmol), to obtain *N1*-cyclohexyl-4-(oxazol-5-yl)benzene-1,2-diamine **96** (0.618 g, 2.402 mmol, 30% yield). ^1^H NMR (400 MHz, DMSO-*d*_6_) δ 1.11 – 1.26 (m, 3H), 1.35 (qt, *J* = 3.3, 12.8 Hz, 2H), 1.62 (dt, *J* = 3.7, 12.7 Hz, 1H), 1.73 (dt, *J* = 3.7, 13.3 Hz, 2H), 1.91 – 2.02 (m, 2H), 3.17 – 3.29 (m, 1H), 4.50 (d, *J* = 7.5 Hz, 1H), 4.75 (br. s, 2H), 6.49 (d, *J* = 8.1 Hz, 1H), 6.83 – 6.87 (m, 1H), 6.88 (d, *J* = 2.0 Hz, 1H), 7.20 (s, 1H), 8.23 (s, 1H); ^13^C NMR (101 MHz, DMSO-*d*_6_) δ 25.19, 26.13, 33.21, 51.26, 110.23, 110.30, 114.78, 115.83, 118.34, 135.65, 136.05, 150.44, 152.66; t_R_ 1.79 min, 100%; HRMS (ESI) *m*/*z* [M+H]^+^ calcd for C_15_H_19_N_3_O 258.1601, found 258.1602.

#### *N*1-Cyclooctyl-4-(oxazol-5-yl)benzene-1,2-diamine (**97**)

General procedure C was followed using compound **92** (0.830 g, 2.630 mmol) to obtain *N*1-cyclooctyl-4-(oxazol-5-yl)benzene-1,2-diamine **97** (0.158 g, 0.554 mmol, 21% yield). ^1^H NMR (400 MHz, MeOD) δ 1.53 – 1.73 (m, 10H), 1.73 – 1.85 (m, 2H), 1.86 – 1.95 (m, 2H), 3.54 (ddt, *J* = 12.3, 8.5, 3.5 Hz, 1H), 6.55 (d, *J* = 8.8 Hz, 1H), 7.04 – 7.08 (m, 2H), 7.16 (s, 1H), 8.09 (s, 1H); ^13^C NMR (101 MHz, MeOD) δ 25.32, 27.12, 28.22, 33.63, 53.78, 112.58, 113.34, 117.72, 117.79, 118.51, 135.75, 138.51, 151.34, 154.69; HRMS (ESI) *m*/*z* [M+H]^+^ calcd for C_17_H_23_N_3_O 286.1914, found 286.1904.

#### *N*1-(Adamantan-2-yl)-4-(oxazol-5-yl)benzene-1,2-diamine (**98**)

General procedure C was followed using compound **93** (0.363 g, 1.070 mmol) to obtain *N*1-(adamantan-2-yl)-4-(oxazol-5-yl)benzene-1,2-diamine **98** (0.166 g, 0.536 mmol, 50% yield). ^1^H NMR (400 MHz, CDCl_3_) δ 1.61 (d, *J* = 12.8 Hz, 2H), 1.70 – 2.11 (m, 13H), 3.39 (s, 2H), 3.59 (s, 1H), 6.62 (d, *J* = 8.2 Hz, 1H), 7.03 (d, *J* = 2.0 Hz, 1H), 7.08 – 7.18 (m, 2H), 7.82 (s, 1H); ^13^C NMR (101 MHz, CDCl_3_) δ 27.78, 27.91, 32.12, 32.16, 37.77, 38.10, 57.04, 77.68, 111.58, 113.25, 117.82, 118.87, 133.85, 137.94, 149.35, 152.39, 156.90; HRMS (ESI) *m*/*z* [M+H]^+^ calcd for C_19_H_23_N_3_O 310.1914, found 310.1919.

#### *N*2-Benzyl-*N*1-cyclobutyl-4-(oxazol-5-yl)benzene-1,2-diamine (**99**)

General procedure N was followed using compound **94** (0.102 g, 0.445 mmol) to obtain *N*2-benzyl-*N*1-cyclobutyl-4-(oxazol-5-yl)benzene-1,2-diamine **99** (0.013 g, 0.043 mmol, 10% yield). ^1^H NMR (400 MHz, MeOD) δ 1.57 – 1.68 (m, 2H), 1.78 – 1.90 (m, 2H), 2.00 – 2.11 (m, 2H), 3.60 – 3.71 (m, 1H), 4.03 (s. 2H), 6.72 (dd, *J* = 2.4, 8.3 Hz, 1H), 6.91 (dt, *J* = 1.9, 8.2 Hz, 1H), 7.02 – 7.08 (m, 2H), 7.09 (d, *J* = 2.0 Hz, 1H), 7.14 – 7.22 (m, 3H), 7.31 (d, *J* = 1.9 Hz, 1H), 8.16 (d, *J* = 2.9 Hz, 1H); ^13^C NMR (101 MHz, MeOD) δ 13.73, 27.59, 53.58, 55.65, 110.61, 113.53, 119.32, 123.80, 124.51, 126.59, 127.48, 129.20, 135.47, 137.46, 144.35, 150.80, 152.53; HRMS (ESI) *m*/*z* [M+H]^+^ calcd for C_20_H_21_N_3_O 320.1757, found 320.1752.

#### *N*2-Benzyl-*N*1-cyclopentyl-4-(oxazol-5-yl)benzene-1,2-diamine (**100**)

General procedure N was followed using compound **95** (0.038 g, 0.156 mmol) to obtain *N*2-benzyl-*N*1-cyclopentyl-4-(oxazol-5-yl)benzene-1,2-diamine **100** (0.008 g, 0.024 mmol, 16% yield). ^1^H NMR (400 MHz, MeOD) δ 1.52 – 1.68 (m, 4H), 1.70 – 1.85 (m, 2H), 1.99 – 2.12 (m, 2H), 3.86 (dq, *J* = 5.7, 12.1 Hz, 1H), 4.38 (s, 2H), 6.66 (d, *J* = 8.2 Hz, 1H), 6.82 (d, *J* = 1.9 Hz, 1H), 7.02 (dd, *J* = 1.9, 8.2 Hz, 1H), 7.08 (s, 1H), 7.18 – 7.27 (m, 1H), 7.32 (dd, *J* = 6.8, 8.4 Hz, 2H), 7.37 – 7.44 (m, 2H), 8.05 (s, 1H); ^13^C NMR (101 MHz, MeOD) δ 25.26, 34.27, 55.84, 108.69, 112.46, 116.45, 118.17, 118.49, 127.97, 128.58, 129.47, 137.86, 139.00, 141.17, 151.30, 154.95; HRMS (ESI) *m*/*z* [M+H]^+^ calcd for C_21_H_23_N_3_O 334.1914, found 334.1922.

#### *N*2-Benzyl-*N*1-cyclohexyl-4-(oxazol-5-yl)benzene-1,2-diamine (**101**)

General procedure N was followed using compound **96** (0.160 g, 0.620 mmol) to otain *N*2-benzyl-*N*1-cyclohexyl-4-(oxazol-5-yl)benzene-1,2-diamine **101** (0.060 g, 0.168 mmol, 27% yield). ^1^H NMR (400 MHz, DMSO-*d*_6_) δ 1.10 – 1.28 (m, 4H), 1.35 (dd, *J* = 11.0, 14.2 Hz, 2H), 1.63 (d, *J* = 13.0 Hz, 1H), 1.69 – 1.79 (m, 1H), 2.00 (d, *J* = 12.2 Hz, 2H),3.19 – 3.33 (m, 1H), 4.33 (d, *J* = 5.2 Hz, 2H), 4.73 (d, *J* = 7.3 Hz, 1H), 5.48 (t, *J* = 5.6 Hz, 1H), 6.52 (d, *J* = 8.3 Hz, 1H), 6.67 (d, *J* = 2.0 Hz, 1H), 6.87 (dd, *J* = 8.1, 1.9 Hz, 1H), 7.16 – 7.43 (m, 6H), 8.19 (s, 1H); ^13^C NMR (101 MHz, DMSO-*d*_6_) δ 25.21, 26.15, 33.16, 47.45, 51.44, 106.31, 109.91, 114.50, 118.58, 122.29, 127.20, 127.91, 128.77, 129.27, 136.00, 140.46, 150.47, 152.72; HRMS (ESI) *m*/*z* [M+H]^+^ calcd for C_22_H_25_N_3_O 348.2070, found 348.2083.

#### *N*2-Benzyl-*N*1-cyclooctyl-4-(oxazol-5-yl)benzene-1,2-diamine (**102**)

General procedure N was followed using compound **97** (0.100 g, 0.350 mmol) to obtain *N*2-benzyl-*N*1-cyclooctyl-4-(oxazol-5-yl)benzene-1,2-diamine **102** (0.020 g, 0.053 mmol, 15% yield). ^1^H NMR (400 MHz, CDCl_3_) δ 1.48 – 1.82 (m, 13H), 1.85 – 1.94 (m, 2H), 3.54 (tt, *J* = 8.5, 3.5 Hz, 1H), 3.66 (br. s, 1H), 4.34 (s, 2H), 6.67 (d, *J* = 8.2 Hz, 1H), 6.98 (d, *J* = 1.9 Hz, 1H), 7.09 – 7.16 (m, 2H), 7.28 – 7.47 (m, 5H), 7.82 (s, 1H); ^13^C NMR (101 MHz, CDCl_3_) δ 24.18, 26.02, 26.56, 32.80, 48.97, 53.05, 77.28, 108.74, 112.95, 116.41, 119.15, 127.47, 127.97, 128.71, 129.48, 137.25, 139.15, 149.44, 152.64; HRMS (ESI) *m*/*z* [M+H]^+^ calcd for C_24_H_29_N_3_O 376.2383, found 376.2367.

#### *N*1-(Adamantan-2-yl)-*N*2-benzyl-4-(oxazol-5-yl)benzene-1,2-diamine (**103**)

General procedure N was followed using compound **98** (0.040 g, 0.129 mmol) to obtain *N*1-(adamantan-2-yl)-*N*2-benzyl-4-(oxazol-5-yl)benzene-1,2-diamine **103** (0.024 g, 0.060 mmol, 47% yield). ^1^H NMR (400 MHz, MeOD) δ 1.63 (d, *J* = 12.9 Hz, 2H), 1.82 (br. s, 3H), 1.88 – 2.00 (m, 5H), 2.01 – 2.17 (m, 4H), 3.65 (s, 1H), 4.39 (s, 2H), 6.63 (d, *J* = 8.2 Hz, 1H), 6.90 (d, *J* = 1.9 Hz, 1H), 7.05 (dd, *J* = 2.0, 8.2 Hz, 1H), 7.09 (s, 1H), 7.23 (t, *J* = 7.3 Hz, 1H), 7.32 (t, *J* = 7.5 Hz, 2H), 7.42 (d, *J* = 7.6 Hz, 2H), 8.07 (s, 1H); ^13^C NMR (101 MHz, DMSO-*d*_6_) δ 27.34, 27.51, 31.37, 31.53, 37.26, 37.73, 47.32, 56.63, 106.99, 110.35, 114.69, 116.28, 118.68, 127.10, 127.62, 128.75, 136.30, 136.58, 140.73, 150.52, 152.65; HRMS (ESI) *m*/*z* [M+H]^+^ calcd for C_26_H_29_N_3_O 400.2383, found 400.2374.

#### *N*1-Cyclohexyl-*N*2-(4-fluorobenzyl)-4-(oxazol-5-yl)benzene-1,2-diamine (**104**)

General procedure N was followed using compound **96** (0.160 g, 0.620 mmol) to obtain *N*1-cyclohexyl-*N*2-(4-fluorobenzyl)-4-(oxazol-5-yl)benzene-1,2-diamine **104** (0.043 g, 0.119 mmol, 19% yield). ^1^H NMR (400 MHz, DMSO-*d*_6_) δ 1.12 – 1.27 (m, 3H), 1.28 – 1.42 (m, 2H), 1.58 – 1.68 (m, 1H), 1.69 – 1.78 (m, 2H), 1.93 – 2.04 (m, 2H), 3.27 (s, 1H), 4.33 (d, *J* = 4.4 Hz, 2H), 4.73 (s, 1H), 5.49 (s, 1H), 6.53 (d, *J* = 8.2 Hz, 1H), 6.67 (d, *J* = 2.0 Hz, 1H), 6.89 (dd, *J* = 2.0, 8.1 Hz, 1H), 7.11 – 7.24 (m, 3H), 7.39 – 7.49 (m, 2H), 8.20 (s, 1H). ^13^C NMR (101 MHz, DMSO-*d*_6_) δ 25.19, 26.14, 33.13, 46.65, 51.47, 106.37, 109.98, 114.59, 115.48 (d, *J* = 21.0 Hz, 118.64, 129.77 (d, *J* = 8.1 Hz), 132.34, 135.24, 136.27, 136.53 (d, *J* = 2.9 Hz), 150.50, 152.66, 161.61 (d, *J* = 242.0 Hz); HRMS (ESI) *m*/*z* [M+H]^+^ calcd for C_22_H_24_FN_3_O 366.1976, found 366.1966.

#### *N*1-Cyclohexyl-4-(oxazol-5-yl)-*N*2-(pyridin-4-ylmethyl)benzene-1,2-diamine (**105**)

General procedure N was followed using compound **96** (0.080 g, 0.310 mmol) to obtain *N*1-cyclohexyl-4-(oxazol-5-yl)-*N*2-(pyridin-4-ylmethyl)benzene-1,2-diamine **54** (0.008 g, 0.022 mmol, 4% yield). ^1^H NMR (400 MHz, DMSO- *d*_6_) δ 1.07 – 1.45 (m, 8H), 1.69 (dd, *J* = 12.4, 46.8 Hz, 3H), 2.01 (d, *J* = 12.8 Hz, 2H), 4.40 (d, *J* = 5.5 Hz, 2H), 4.72 (d, *J* = 6.9 Hz, 1H), 5.63 (s, 1H), 6.43 – 6.63 (m, 2H), 6.89 (d, *J* = 8.2 Hz, 1H), 7.18 (s, 1H), 7.38 (d, *J* = 4.9 Hz, 2H), 8.18 (s, 1H), 8.50 (d, *J* = 4.8 Hz, 2H); ^13^C NMR (101 MHz, DMSO-*d*_6_) δ 24.62, 25.60, 32.62, 45.69, 50.86, 105.77, 109.57, 114.28, 115.46, 118.12, 122.35, 122.35, 134.98, 135.80, 149.48, 149.96, 152.02; HRMS (ESI) *m*/*z* [M+H]^+^ calcd for C_21_H_24_N_4_O 349.2023, found 349.2009.

### Inhibition of ML-162-Induced Ferroptosis in HT-1080 Human Fibrosarcoma Cells

Human fibrosarcoma cells HT1080 cells were obtained from American Type Culture Collection (ATCC). HT-1080 cells were cultured in EMEM medium supplemented with 10% FCS and L-glutamine (1 mM), sodium pyruvate and nonessential amino acids. Cell death was measured using Envision multimode plate reader (PE). In order to determine IC_50_ values, HT-1080 were seeded in a 384-well plate at a density of 3500 cells/well in 40 μL in an incubator overnight at 37 °C with 5% CO_2_. The next day, the cells were pretreated for 1h (in triplicates) with a 1/3 dilution series of ferrostatin-1 analogs ranging from 1 μM to 0.5 nM and Sytox Green (1.6 μM). All the compound’s stock solutions were prepared in DMSO at 100 mM concentration. After stimulating the cells with ML-162 1 μM the plate was transferred to the incubator. SytoxGreen intensity was measured after 8, 12, 16 and 24 h using an excitation filter of 485 nm and an emission filter of 535 nm. Dose–response curves were made as % cell death inhibition, taking ML-162 treated samples as 0% inhibition and Fer-1 (1 μM) + ML-162 samples as the 100% inhibition. Cell death percentage was calculates as 100 – ((x – 100%inh)/(0%inh – 100%inh))*100). Curves were plotted in Spotfire software, and IC_50_ values were calculated using logistic regression curves.

### Fluorescence-Enabled Inhibited Autoxidation (FENIX)^44^

Unless otherwise stated, laboratory reagent-grade solvents were used. Reagents were obtained from various commercial sources and were used without any prior purification. L-α-phosphatidylcholine (Egg, Chicken), the powder was purchased from Merck Life Science B.V.. DTUN and STY-BODIPY were synthesized according to methods described in Shah et al. (2019) Cell Chem. Biol. 26, 1594–1607. Fluorescence was measured using the UV/vis spectrophotometer Synergy MX, Biotek with Gen5. In order to quantify radical trapping antioxidant activity in phospholipid bilayers to a clear bottom black-walled 96-well plate for fluorescence-base assays (Invitrogen by Thermo Fisher Scientific) was added 250 μL of a solution containing liposomes (1 mM), styrene-conjugated BODIPY (STY-BODIPY) (1 μM), and the respective radical trapping antioxidant (RTA) (4 μM). The solutions were made in larger volumes in Eppendorfs, premixed and 250 μL aliquot was transferred to each well. The plate was incubated for 10 min at 37 °C in the BioTek SynergyMx plate reader, followed by a fast-mixing protocol for 5 min. The plate was ejected from the plate reader and autoxidation was initiated by the addition of a 50 μL aliquot of (E)-1,2-*bis*((2-methyldecan-2-yl)oxy)diazene (DTUN) (0.2 mM in EtOH/PBS (3/47, v/v), and the well were sealed with fluorescence compatible black film to avoid evaporation, followed by another mixing protocol for 5 min. Data were acquired by excitation probes at 488 nm and emission was measured at 518 nm (read intervals 1.0 min). Kinetic read parameters: (i) optic position: bottom, (ii) gain: 80, (iii) bandwidth: 9.0. The results of the FENIX assay are presented in the Supporting Information as inhibition rate constants (k_inh_), logk_inh_ and stoichiometries (n) of tested compounds. Data processing is reported in the Supporting Information.

### Kinetic Solubility

A turbidimetric method was used. First, a series of DMSO compound stock solutions were prepared (0.15–5 mM) from the stock solution of the compound in DMSO (10 mM). An aliquot of 4 μL stock solution was added to 196 μL PBS buffer (pH 7.4). A series of concentrations were prepared (3.13–200 μM), including a blank on a 96-well clear microtiter plate. The microtiter plate was shaken for 10 s and incubated for 2 h at 37 °C. Turbidity was measured using the UV/vis spectrophotometer Synergy MX, Biotek with Gen5. When there was no turbidity measured at a given concentration the sample was assumed to be dissolved.

### Central Nervous System Multiparameter Optimization (CNS MPO) Score^45^

In order to evaluate brain penetration, central nervous system multiparameter optimization (CNS MPO) score was calculated using CDD Vault software (Collaborative Drug Discovery Inc., Burlingame, California, USA). CNS MPO score consists of six fundamental physicochemical properties: lipophilicity, calculated partition coefficient (ClogP), calculated distribution coefficient at pH 7.4 (ClogD), molecular weight (MW), topological polar surface area (TPSA), number of hydrogen-bond donors (HBDs), and most basic center (p*K*_a_).

### Microsomal Stability in Human/Mouse Microsome

Human microsomal stability: Pooled liver microsomes (Ultrapool Human Liver microsomes) at 20 mg protein/ml were purchased from Corning Gentest (now Discovery Life Science – Gentest) and were stored at −80 °C prior to use. Microsomes (final protein concentration 0.5 mg/ml), 0.1 M phosphate buffer pH 7.4 and test compound (final substance concentration 1 μM) are preincubated at 37 °C for 10 min prior the addition of NADPH Regenerating System, (solution A and B from Corning Gentest, final concentration 1 mM to initiate the reaction). A minus cofactor control was included for each compound tested where 0.1 M phosphate buffer pH 7.4 was added instead of NADPH regenerating system. Two positive control compounds were also included to test every new batch of microsome: for human microsome, high (verapamil, Cl_int_ = 113.1 μL/min*mg) and low (dextromethorphan Cl_int_ = 28.5 μL/min*mg) clearance; for mouse microsome high (diazepam Cl_int_ = 549 μL/min*mg) and low (diphenhydramine Cl_int_ = 64.0 μL/min*mg) clearance. Each compound was incubated for 45 min at 37 °C and shake at 500 rpm. The reactions are stopped by transferring incubate into acetonitrile (containing an internal standard) at appropriate time point in a 1:3 ratio (0, 5, 10, 15, 30, 45 for compounds and 0, 15, 45 for negative control of those compounds). The termination plates are centrifuged at 3000 rpm for 20 min at 4 °C to precipitate the protein. Following protein precipitation, the sample supernatant at each time point was analyzed by UPLC MS/MS using our UPLC system described earlier. All obtained readouts were processed using Microsoft Excel. Data processing is reported in the Supporting Information.

### MDCK-MDR1 Permeability Assay (Unidirectional or Bidirectional)

Madin-Darby canine kidney (MDCK) cells are obtained from the NIH (Rockville, MD, USA). Cells are seeded onto Transwell plates at 3.4 × 105 cells/cm2. The cells are cultured in DMEM on day five the permeability study is performed. Cell culture and assay incubations are carried out at 37 °C in an atmosphere of 5% CO_2_ with a relative humidity of 95%. On the day of the assay, the monolayers are prepared by rinsing the apical and basolateral compartment with Hanks Balanced Salt Solution (HBSS) at the desired pH warmed to 37 °C. Cells are then incubated with HBSS at the desired pH in both apical and basolateral compartments for 30 min to stabilize physiological parameters. The dosing solutions are prepared by diluting compounds with assay buffer to give the desired final concentration (10 μM). Final DMSO concentration of 1% v/v. The fluorescent integrity marker lucifer yellow is also included in the dosing solution. Analytical standards are prepared from test article DMSO dilutions and transferred to buffer, maintaining a ≤ 1% v/v DMSO concentration. Typical assay buffer is composed of supplemented HBSS pH 7.4, but a range of other buffers and pH values can be used. For assessment of AB permeability, HBSS is removed from the apical compartment and replaced with compound dosing solution. The apical compartment insert is then placed into a companion plate containing fresh buffer (containing ≤ 1% v/v DMSO). For assessment of BA permeability, HBSS is removed from the companion plate and replaced with compound dosing solution. Fresh buffer (containing ≤ 1% v/v DMSO) is added to the apical compartment insert, which is then placed into the companion plate. At 60 min the apical compartment inserts, and the companion plates are separated and apical and basolateral samples diluted for analysis. compound permeability is assessed in duplicate. Test articles of known permeability characteristics are run as controls on each assay plate. Test articles are quantified by LC-MS/MS using a 7-point calibration with appropriate dilution of the samples. The starting concentration (C_0_) is determined from the dosing solution and the experimental recovery calculated from C_0_ and both apical and basolateral compartment concentrations. The integrity of the monolayer throughout the experiment is checked by monitoring lucifer yellow permeation using fluorimetric analysis. The experiments were perfomed at Cyprotex Discovery Ltd. (UK). Data processing is reported in the Supporting Information.

### PK Experimental

Compounds were dissolved in DMSO/Cremophor/PBS (10/10/80) to reach a final concentration of 2 mg/mL for an intravenous dose of 10 mg/kg (IV) and 1 mg/mL for an oral dose of 10 mg/kg. The study was performed in male CD-1 mice received one week prior to the study from Charles River, Italy. The volume of formulation administered to each animal was adjusted according to the individual body weights recorded on the day of dosing. Following IV dosing plasma samples were collected from 6 animals at 0.05 h and from 3 animals at 0.25, 0.5, 1, 2, 4, 8 and 24 h. Following PO dosing plasma samples were collected by serial sampling from 3 animals at 0.25, 0.5 1, 2, 4, 8 and 24 h. Prior to blood sampling from the tail vein, mice were placed in a warming cabinet (up to 10 min, maximum at 38 °C). Serial blood samples (ca. 100 μL) are collected following successful puncture of the lateral tail vein using a needle, into K2EDTA-coated tubes. For terminal sampling, before sacrifice, the mice were anesthetized with a cocktail of ketamine and xylazine administered IP. For each terminal time point, blood samples were collected via jugular vein bleeds; ca. 0.4 mL of blood were collected and transferred into K2EDTA-coated tubes. Within 30 min after blood collection, samples were centrifuged at 1560 rpm for 10 min at 4 °C and the resulting plasma samples were aliquoted (two aliquots of 20 uL) and stored at −20 °C until analysis. Following IV dosing, selected tissues (brain, lung, heart, kidney, liver) were collected at 0.5h, 4h and 24h, animals are euthanized by exsanguination, tissues were collected into Precellys tubes, weighed individually, frozen and stored at – 70°. Plasma and tissue concentrations are determined by research-qualified LC-MS/MS methods. Pharmacokinetic analysis was performed using WinNonlin Phoenix software (Certara, version 8.3) from animal plasma concentrations, noncompartmental analysis, and the target dose. The experiments were performed at Selvita Ltd., Zagreb, Croatia.

## Supporting Information Available

Molecular formula strings SMILES (CSV)
NMR spectra, detailed protocols, extended data series, additional charts and graphs for *in vitro* ADME analyses and *in vivo* PK (PDF)

## Abbreviations

1,2-DCE: 1,2-dichloroethane
ACN: acetonitrile
ACSL4: acyl-CoA synthetase long-chain family member 4
ATCC: American type culture collection
BBB: blood-brain barrier
CNS: central nervous system
DCM: dichloromethane
DIPEA: *N*,*N*-diisopropylethylamine
DMAP: 4-dimethylaminopyridine
DMSO-*d6*: dimethyl sulfoxide-*d*_6_
DTUN: (*E*)-1,2-bis((2-methyldecan-2-yl)oxy)diazene
EMEM: Eagle’s minimum essential medium
ESI: electrospray ionization
FCC: flash column chromatography
FCS: fetal calf serum
FENIX: fluorescence-enabled inhibited autoxidation assay
Fer-1: ferrostatin 1
FSP1: ferroptosis suppressor protein 1
GPX4: glutathione peroxidase 4
GSH: glutathione
GSSG: glutathione disulfide
HATU: hexafluorophosphate azabenzotriazole tetramethyl uronium
HBD: hydrogen bond donors
hep: heptane
HBSS: Hank’s balanced salt solution
IV: intravenous
LBF: liver blood flow
LOX: lipoxygenase
LPCAT3: lysophosphatidylcholine acyltransferase 3
MDCK: Madin-Darby canine kidney cells
MDR1: multidrug resistance protein 1
MS: mass spectrometry
MW: molecular weight
NMR: nuclear magnetic resonance
PK: pharmacokinetics
PO: oral administration
POR: cytochrome P450 oxidoreductase
PUFA: polyunsaturated fatty acids
RDC: regulated cell death
RTA: radical trapping antioxidant
SAR: structure–activity relationship
SD: standard deviation
TFA: trifluoroacetic acid
THF: tetrahydrofuran
UPLC: ultraperformance liquid chromatography
