# Supplementary material for: Oxazole-Based Ferroptosis Inhibitors with Promising Properties to Treat Central Nervous System Diseases
Source: J Med Chem. 2025 Feb 6;68(4):4908–28. doi: 10.1021/acs.jmedchem.4c03149 (PMC11874020; doi:10.1021/acs.jmedchem.4c03149)
Supplement: Supplementary file 2 — jm4c03149_si_002.pdf [file jm4c03149_si_002.pdf]

SUPPORTING INFORMATION

Oxazole-based ferroptosis inhibitors with promising properties to treat central nervous system (CNS) diseases

*Camilla Scarpellini[1]‡ & Greta Klejborowska[1]‡, Caroline Lanthier[1], Ariane Toye[1], Karolina Musiałek[1], Emily Van San[2], Magali Walravens[2], Maya Berg[3], Behrouz Hassannia[2,4,5], Pieter Van der Veken[1], Hans De Winter[1], Tom Vanden Berghe[2,3,4,5] and Koen Augustyns[1,3]**

[1] Laboratory of Medicinal Chemistry, Department of Pharmaceutical Sciences, Faculty of Pharmaceutical, Biomedical and Veterinary Sciences, University of Antwerp, Antwerp 2610, Belgium

[2] Cell Death Signaling Lab, Department of Biomedical Sciences, University of Antwerp, Antwerp 2610, Belgium

[3] Infla-Med Centre of Excellence, University of Antwerp, Antwerp 2610, Belgium

[4] Molecular Signalling and Cell Death Unit, VIB Center for Inflammation Research, Ghent 9052, Belgium

[5] Department of Biomedical Molecular Biology, Ghent University, Ghent 9000, Belgium

* **Corresponding author**: Koen Augustyns, (koen.augustyns@uantwerpen.be)

Table of Contents

^{1}H NMR spectra of all final compounds and ^{13}C NMR spectra of selected compounds S3

UPLC analyses of selected compounds S100

Inhibition of ML162-Induced Ferroptosis in HT1080 human fibrosarcoma cells – dose-response curves of selected compounds S103

Fluorescence-Enabled Inhibited Autoxidation (FENIX assay) – stoichiometry, k_{inh} and $Logk_{inh}$ S105

Experimental data from kinetic solubility for selected compounds S111

Microsomal stability - data processing description and detailed results for selected compounds S112

In vivo experimental mouse PK – experimental details and concentration in different tissues S115

^{1}H NMR spectra of all final compounds and ^{13}C NMR spectra of selected compounds

Compound 13

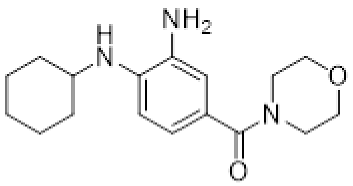

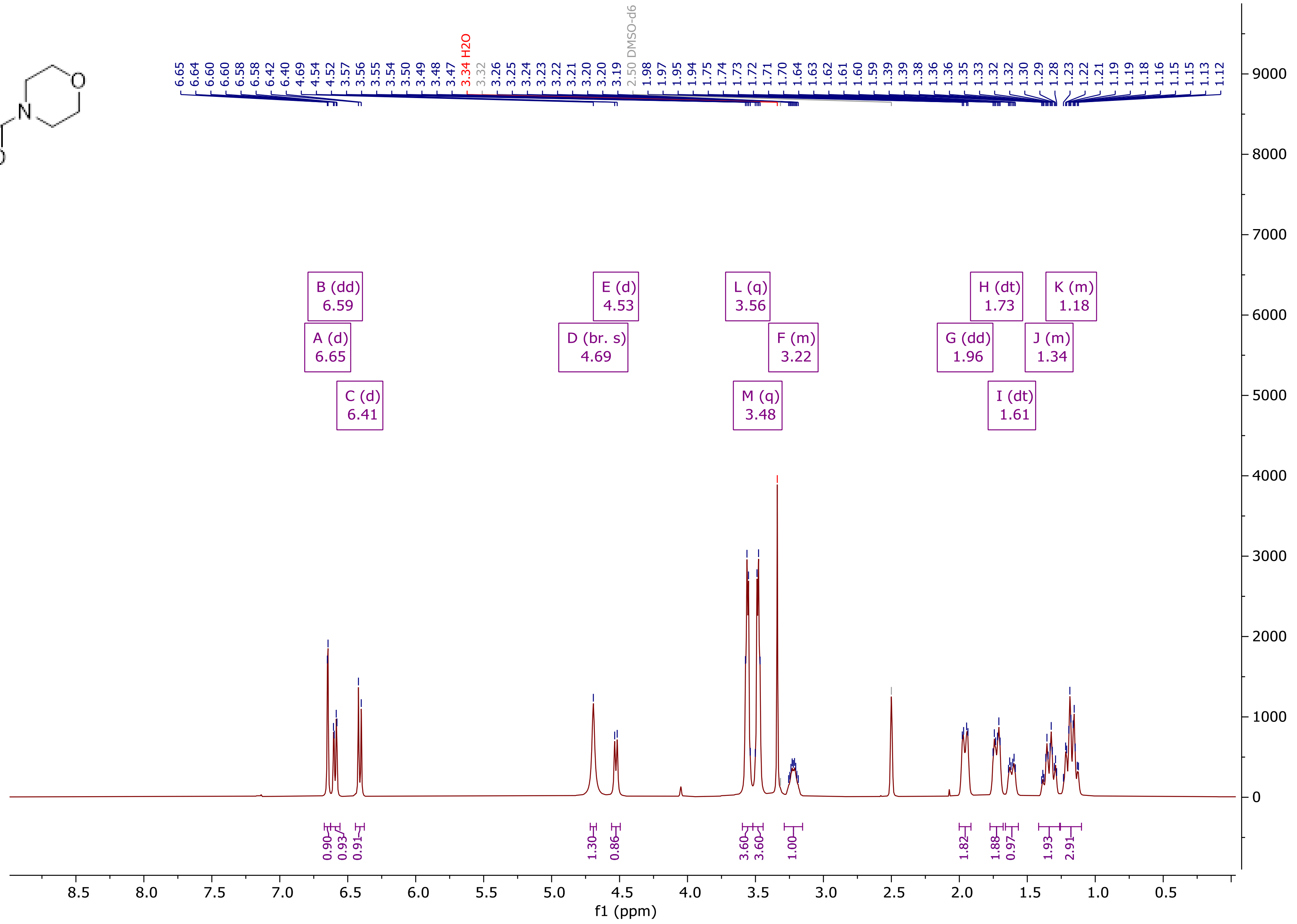

Compound 14

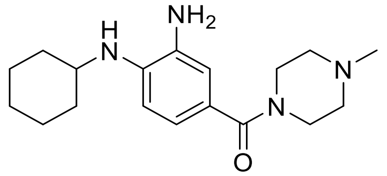

NH2
H
N
N
N
O

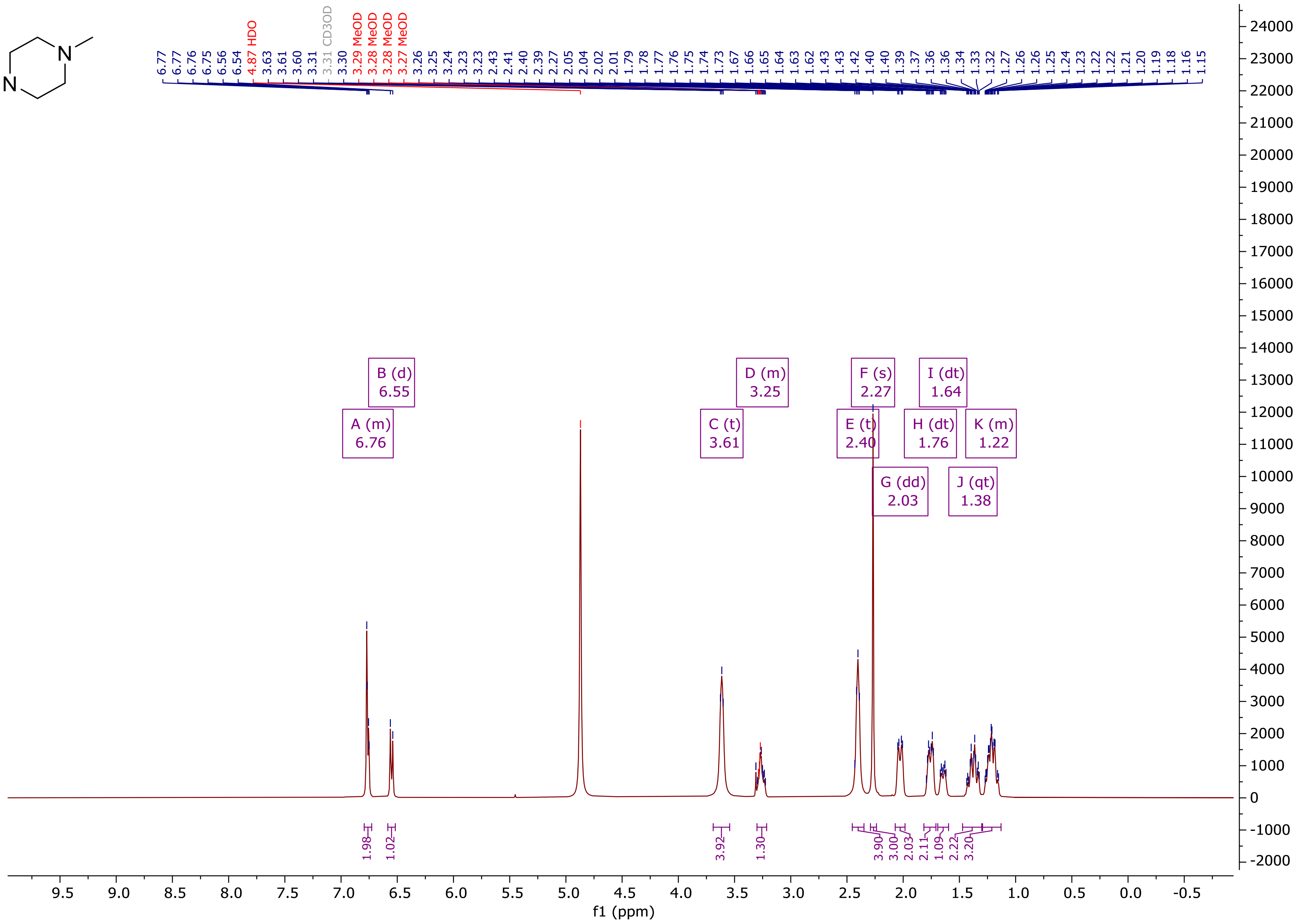

6.77
6.77
6.76
6.75
6.56
6.54
4.87 HDO
3.63
3.61
3.60
3.31
3.31 CD3OD
3.30
3.29 MeOD
3.28 MeOD
3.28 MeOD
3.27 MeOD
3.26
3.25
3.24
3.23
3.23
2.43
2.41
2.40
2.39
2.27
2.05
2.04
2.02
2.01
1.79
1.78
1.77
1.76
1.75
1.74
1.73
1.67
1.66
1.65
1.64
1.63
1.62
1.43
1.43
1.42
1.40
1.40
1.39
1.37
1.36
1.36
1.34
1.33
1.32
1.27
1.26
1.26
1.25
1.24
1.23
1.22
1.22
1.21
1.20
1.19
1.18
1.16
1.15
B (d) 6.55
A (m) 6.76
D (m) 3.25
C (t) 3.61
F (s) 2.27
E (t) 2.40
I (dt) 1.64
H (dt) 1.76
K (m) 1.22
G (dd) 2.03
J (qt) 1.38
1.98
1.02
3.92
1.30
3.90
3.00
2.03
2.11
1.09
2.22
3.20
24000
23000
22000
21000
20000
19000
18000
17000
16000
15000
14000
13000
12000
11000
10000
9000
8000
7000
6000
5000
4000
3000
2000
1000
0
-1000
-2000
9.5
9.0
8.5
8.0
7.5
7.0
6.5
6.0
5.5
5.0
4.5
4.0
3.5
3.0
2.5
2.0
1.5
1.0
0.5
0.0
-0.5
f1 (ppm)

Compound 15

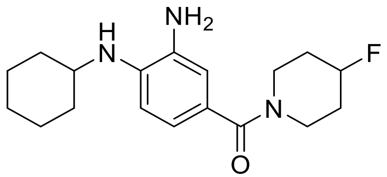
NH2
H
N
F
N
O

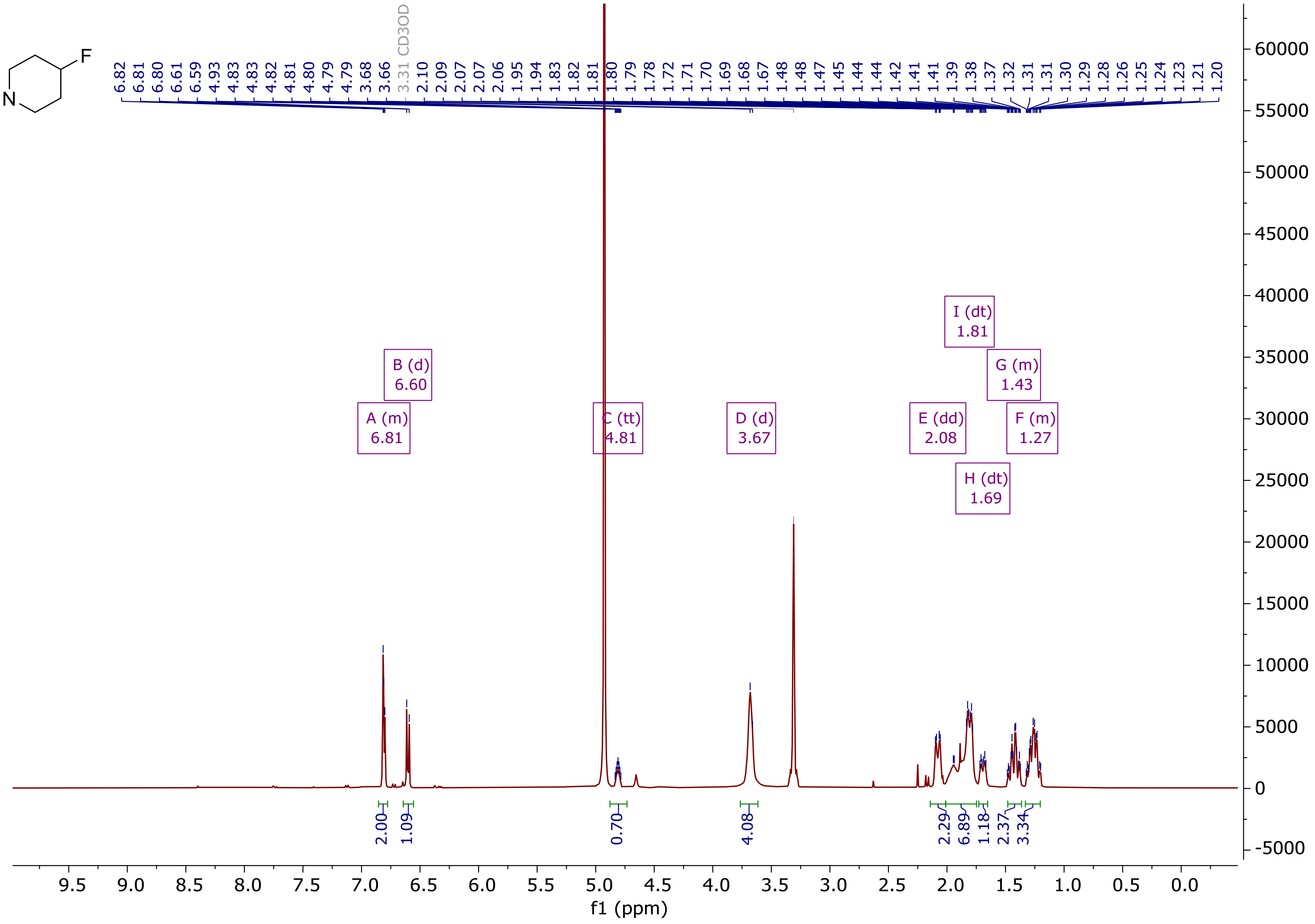
6.82
6.81
6.80
6.61
6.59
4.93
4.83
4.83
4.82
4.81
4.80
4.79
4.79
3.68
3.66
3.31 CD3OD
2.10
2.09
2.07
2.07
2.06
1.95
1.94
1.83
1.82
1.81
1.80
1.79
1.78
1.72
1.71
1.70
1.69
1.68
1.67
1.48
1.48
1.47
1.45
1.44
1.44
1.42
1.41
1.41
1.39
1.38
1.37
1.32
1.31
1.31
1.30
1.29
1.28
1.26
1.25
1.24
1.23
1.21
1.20
A (m) 6.81
B (d) 6.60
C (tt) 4.81
D (d) 3.67
E (dd) 2.08
I (dt) 1.81
H (dt) 1.69
G (m) 1.43
F (m) 1.27
2.00
1.09
0.70
4.08
2.29
6.89
1.18
2.37
3.34
f1 (ppm)

Compound 16

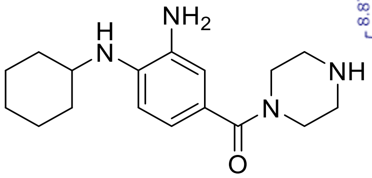
NH2
H
N
N
NH
O

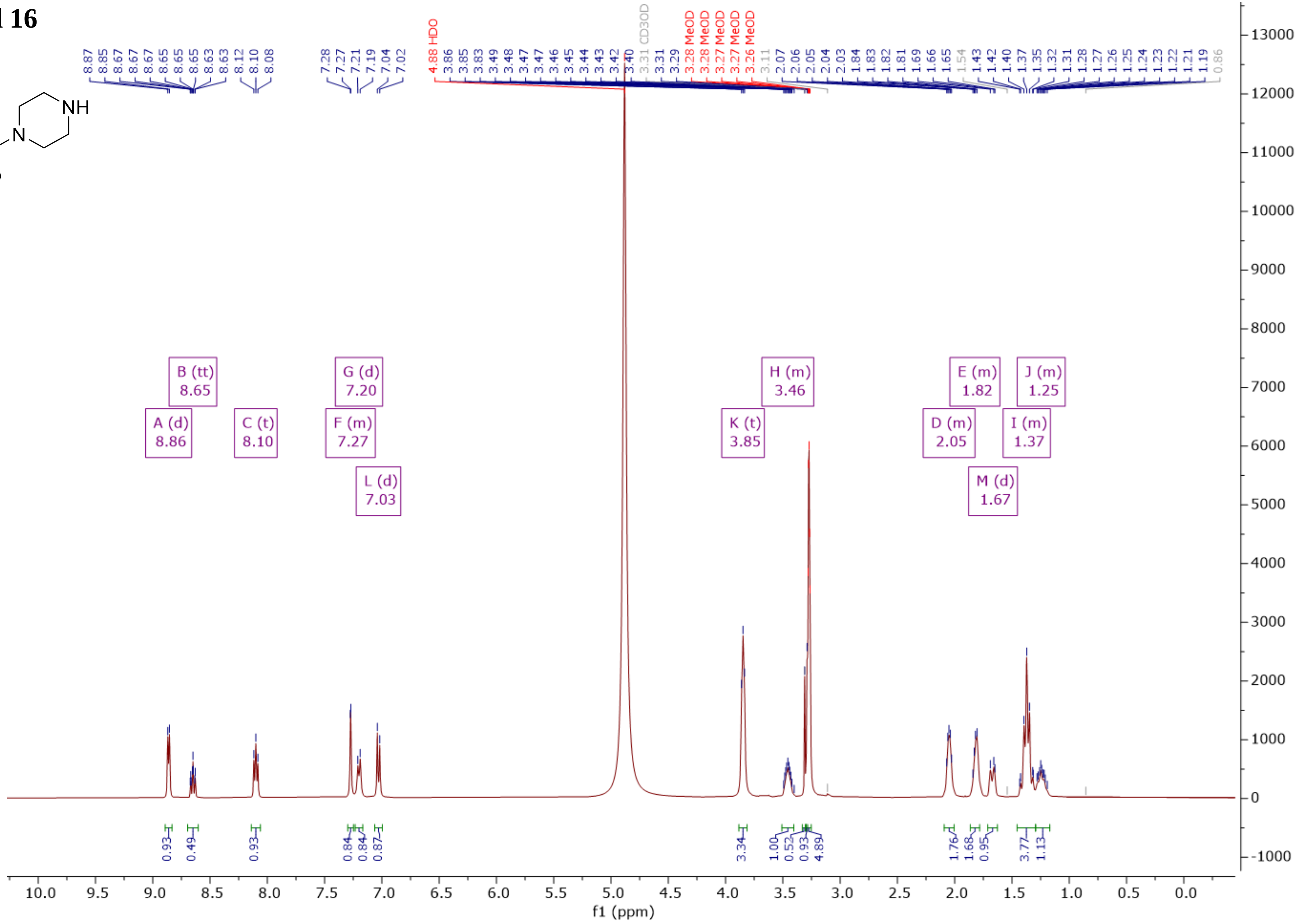
A (d)
8.86
B (tt)
8.65
C (t)
8.10
F (m)
7.27
G (d)
7.20
L (d)
7.03
K (t)
3.85
H (m)
3.46
D (m)
2.05
E (m)
1.82
M (d)
1.67
I (m)
1.37
J (m)
1.25
4.88 HDO
3.31 CD3OD
f1 (ppm)

Compound 35

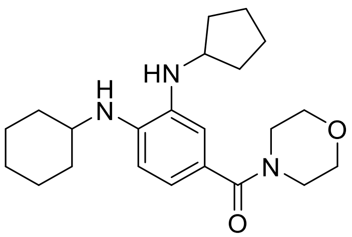
HN
H
N
N
O
O

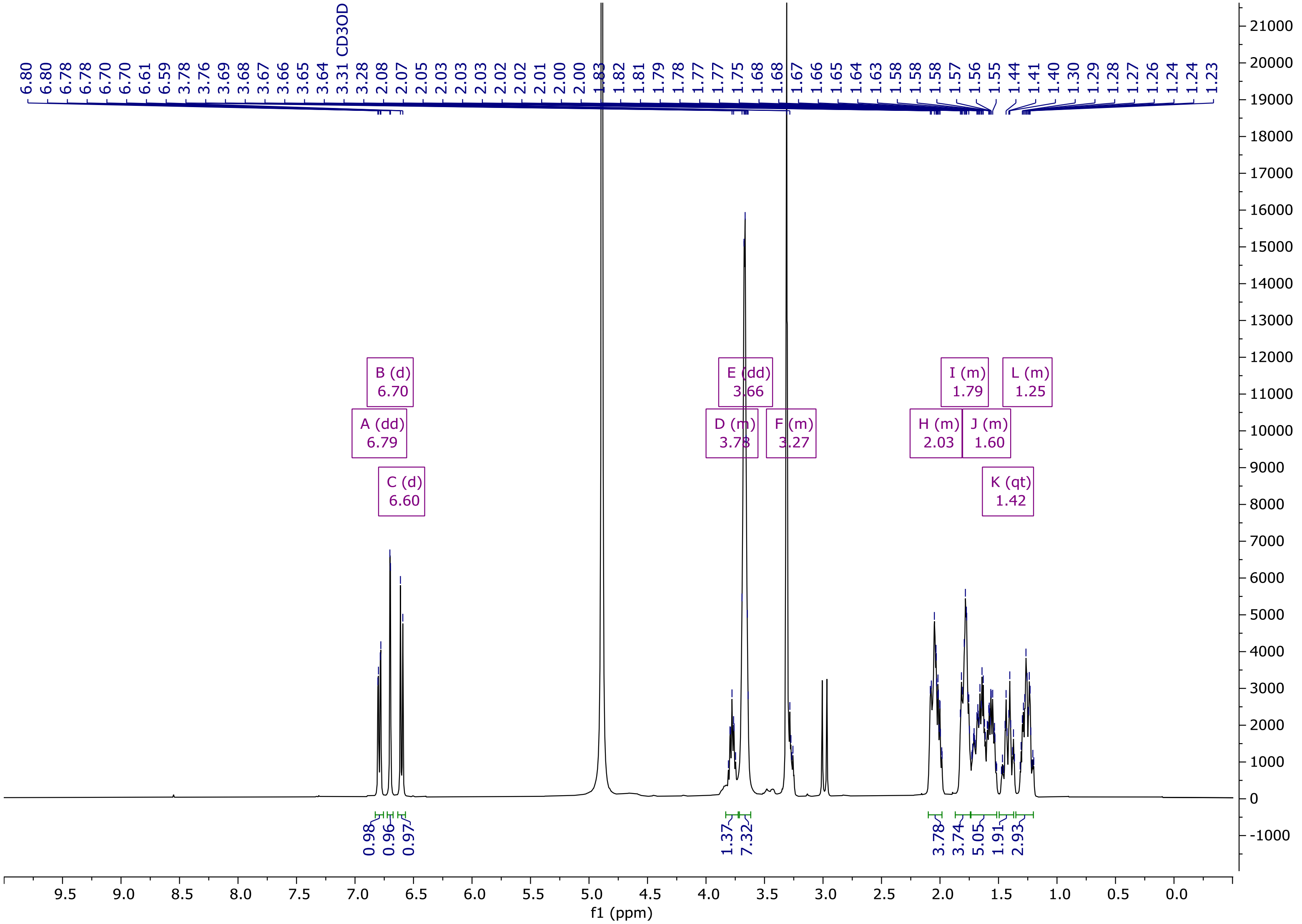
6.80 6.80 6.78 6.78 6.70 6.70 6.61 6.59 3.78 3.76 3.69 3.68 3.67 3.66 3.65 3.64 3.31 CD3OD 3.28 2.08 2.07 2.05 2.03 2.03 2.03 2.02 2.02 2.01 2.00 2.00 1.83 1.82 1.81 1.79 1.78 1.77 1.77 1.75 1.68 1.68 1.67 1.66 1.65 1.64 1.63 1.58 1.58 1.58 1.57 1.56 1.55 1.44 1.41 1.40 1.30 1.29 1.28 1.27 1.26 1.24 1.24 1.23
B (d) 6.70
A (dd) 6.79
C (d) 6.60
E (dd) 3.66
D (m) 3.78
F (m) 3.27
I (m) 1.79
L (m) 1.25
H (m) 2.03
J (m) 1.60
K (qt) 1.42
0.98 0.96 0.97
1.37 7.32
3.78 3.74 5.05 1.91 2.93
f1 (ppm)

Compound 36

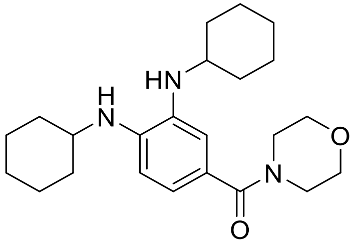
HN
H
N
N
O
O

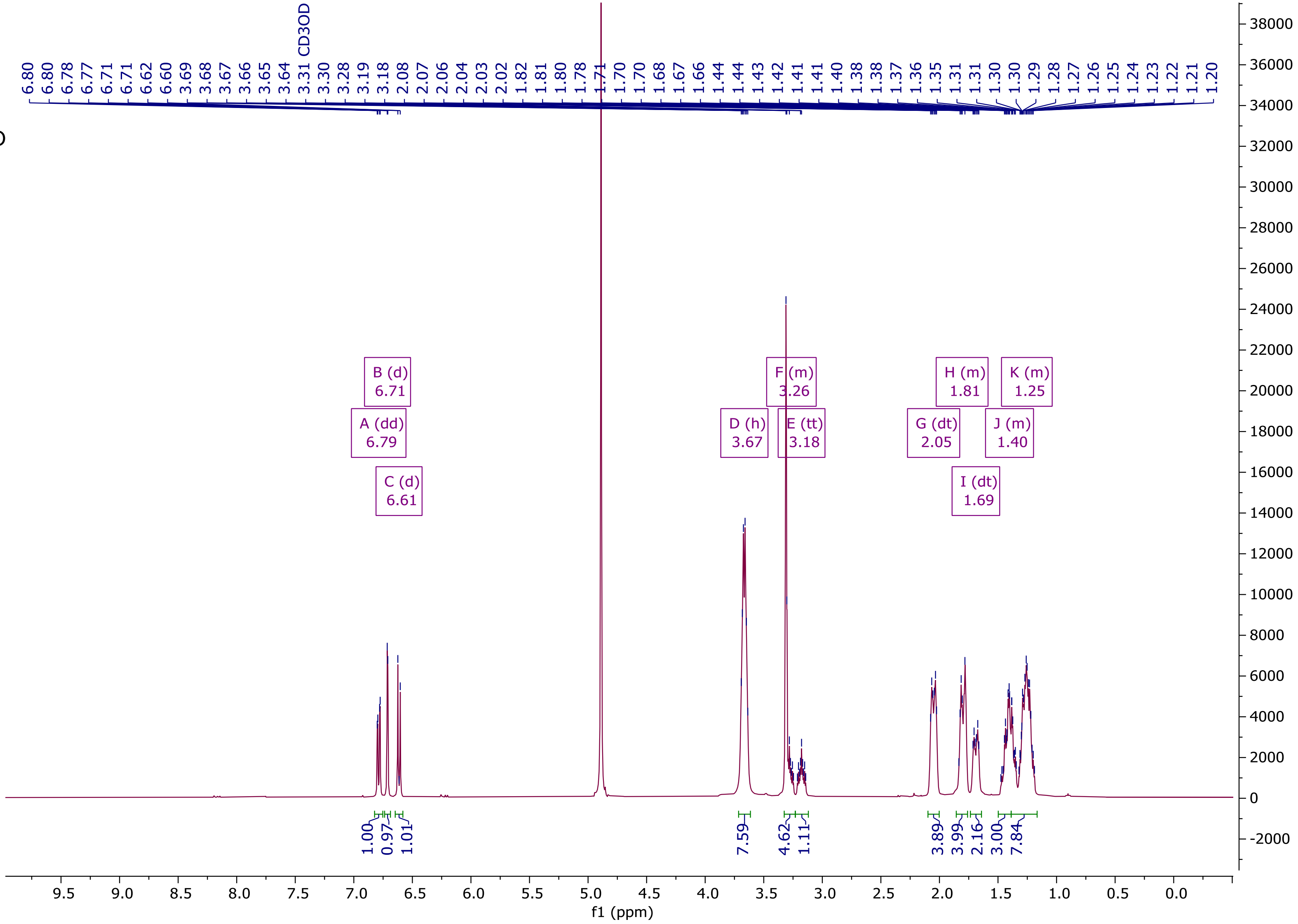
6.80
6.80
6.78
6.77
6.71
6.71
6.62
6.60
3.69
3.68
3.67
3.66
3.65
3.64
3.31 CD3OD
3.30
3.28
3.19
3.18
2.08
2.07
2.06
2.04
2.03
2.02
1.82
1.81
1.80
1.78
1.71
1.70
1.70
1.68
1.67
1.66
1.44
1.44
1.43
1.42
1.41
1.41
1.40
1.38
1.38
1.37
1.36
1.35
1.31
1.31
1.30
1.30
1.29
1.28
1.27
1.26
1.25
1.24
1.23
1.22
1.21
1.20
B (d) 6.71
A (dd) 6.79
C (d) 6.61
D (h) 3.67
F (m) 3.26
E (tt) 3.18
G (dt) 2.05
H (m) 1.81
I (dt) 1.69
J (m) 1.40
K (m) 1.25
1.00
0.97
1.01
7.59
4.62
1.11
3.89
3.99
2.16
3.00
7.84
9.5
9.0
8.5
8.0
7.5
7.0
6.5
6.0
5.5
5.0
4.5
4.0
3.5
3.0
2.5
2.0
1.5
1.0
0.5
0.0
f1 (ppm)
38000
36000
34000
32000
30000
28000
26000
24000
22000
20000
18000
16000
14000
12000
10000
8000
6000
4000
2000
0
-2000

Compound 37

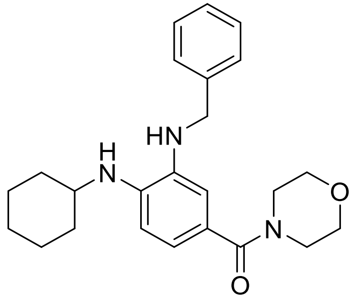

HN
H
N
N
O
O

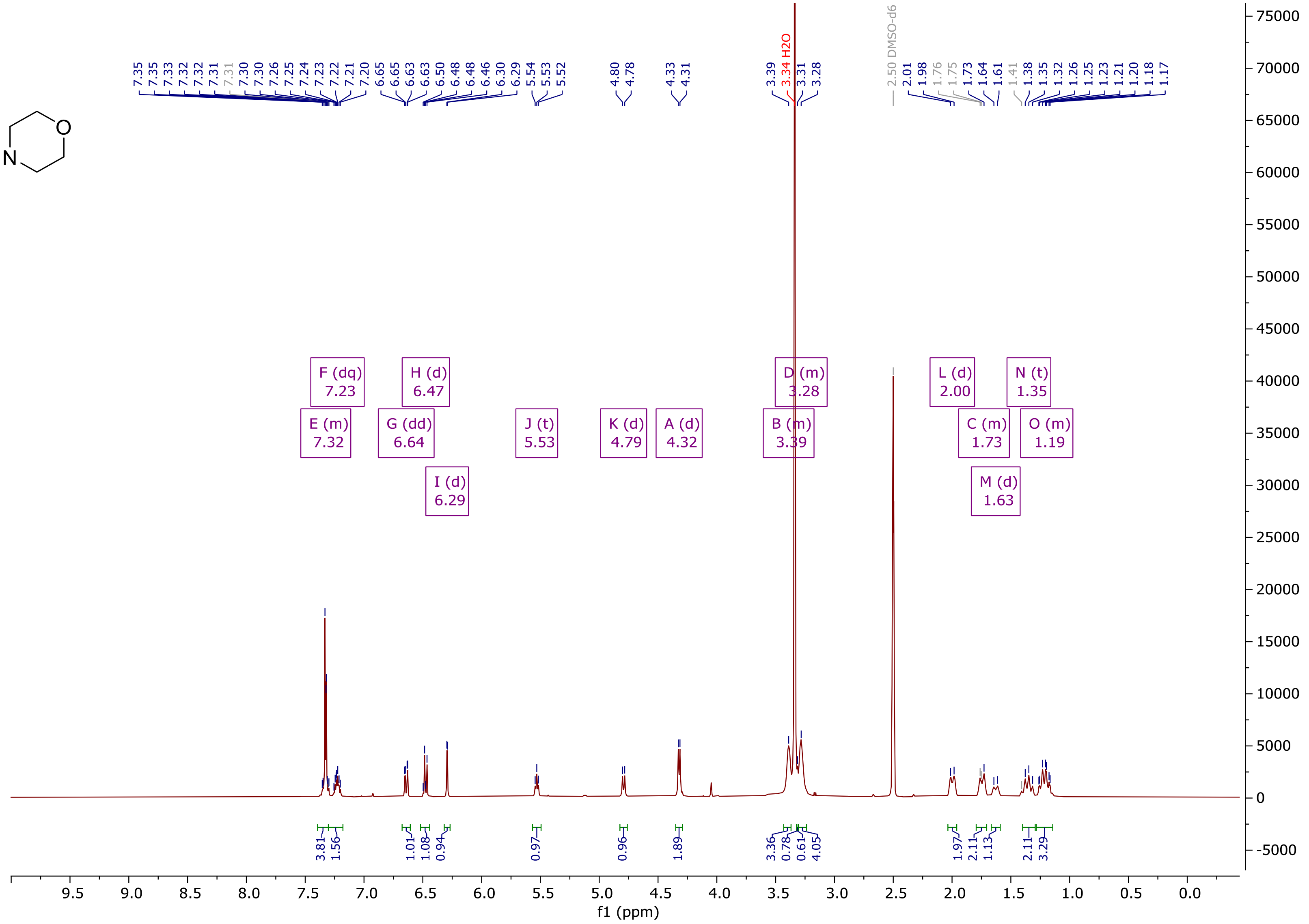

7.35 7.35 7.33 7.32 7.32 7.31 7.31 7.30 7.30 7.26 7.25 7.24 7.23 7.22 7.21 7.20 6.65 6.65 6.63 6.63 6.50 6.48 6.48 6.46 6.30 6.29 5.54 5.53 5.52 4.80 4.78 4.33 4.31 3.39 3.34 H2O 3.31 3.28 2.50 DMSO-d6 2.01 1.98 1.76 1.75 1.73 1.64 1.61 1.41 1.38 1.35 1.32 1.26 1.25 1.23 1.21 1.20 1.18 1.17
F (dq) 7.23
E (m) 7.32
H (d) 6.47
G (dd) 6.64
I (d) 6.29
J (t) 5.53
K (d) 4.79
A (d) 4.32
D (m) 3.28
B (m) 3.39
L (d) 2.00
C (m) 1.73
M (d) 1.63
N (t) 1.35
O (m) 1.19
3.81 1.56 1.01 1.08 0.94 0.97 0.96 1.89 3.36 0.78 0.61 4.05 1.97 2.11 1.13 2.11 3.29
9.5 9.0 8.5 8.0 7.5 7.0 6.5 6.0 5.5 5.0 4.5 4.0 3.5 3.0 2.5 2.0 1.5 1.0 0.5 0.0
f1 (ppm)
75000 70000 65000 60000 55000 50000 45000 40000 35000 30000 25000 20000 15000 10000 5000 0 -5000

Compound 38

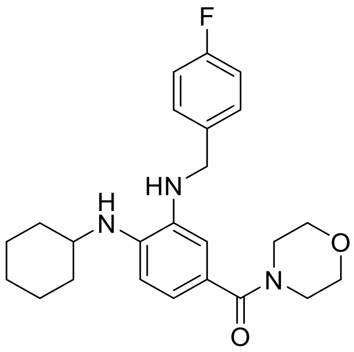

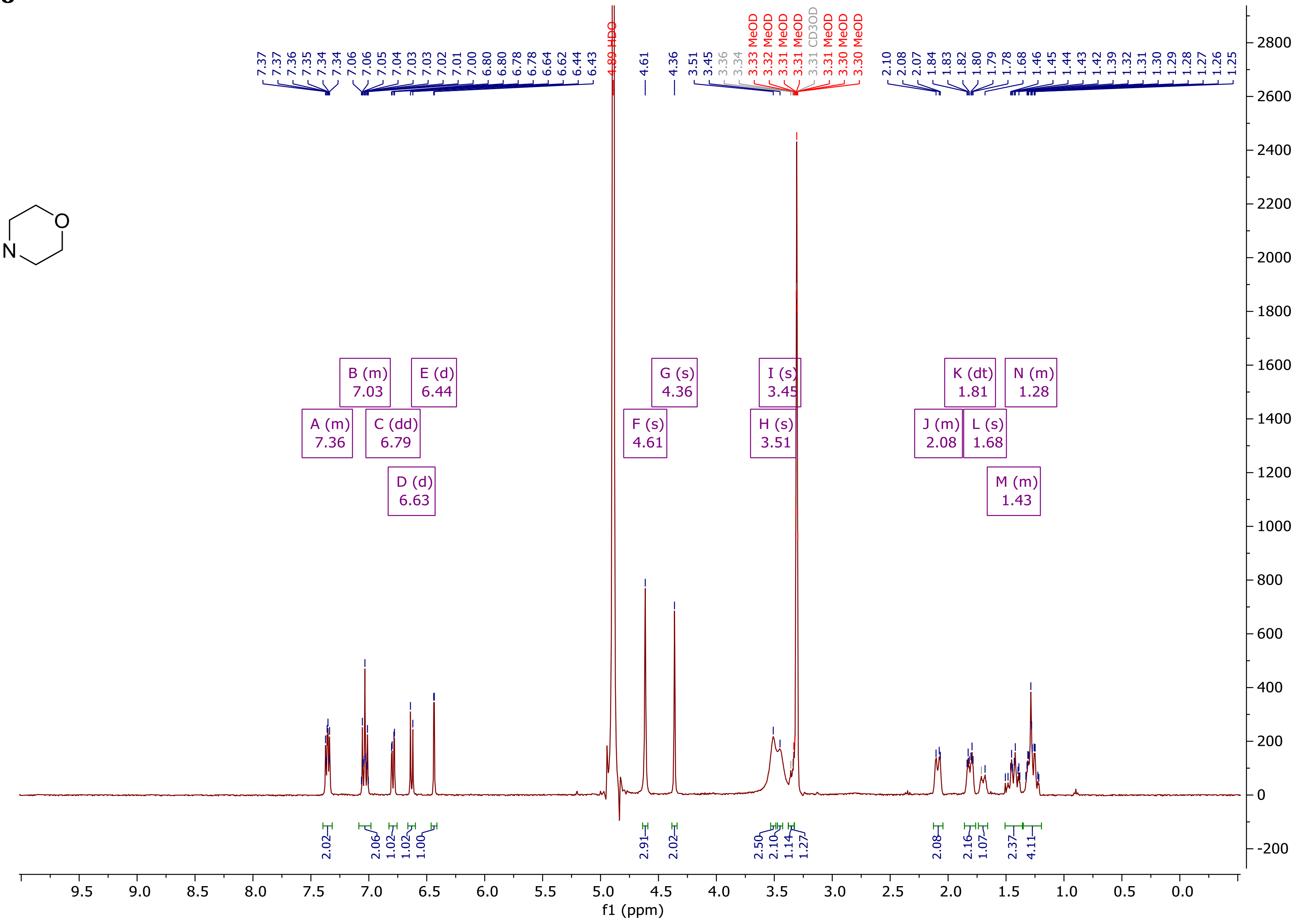

A (m) 7.36
B (m) 7.03
C (dd) 6.79
D (d) 6.63
E (d) 6.44
F (s) 4.61
G (s) 4.36
H (s) 3.51
I (s) 3.45
J (m) 2.08
K (dt) 1.81
L (s) 1.68
M (m) 1.43
N (m) 1.28
f1 (ppm)

Compound 39

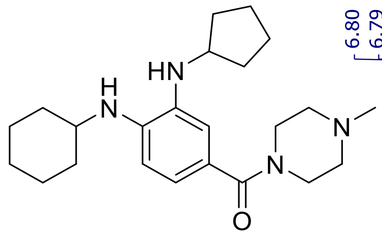

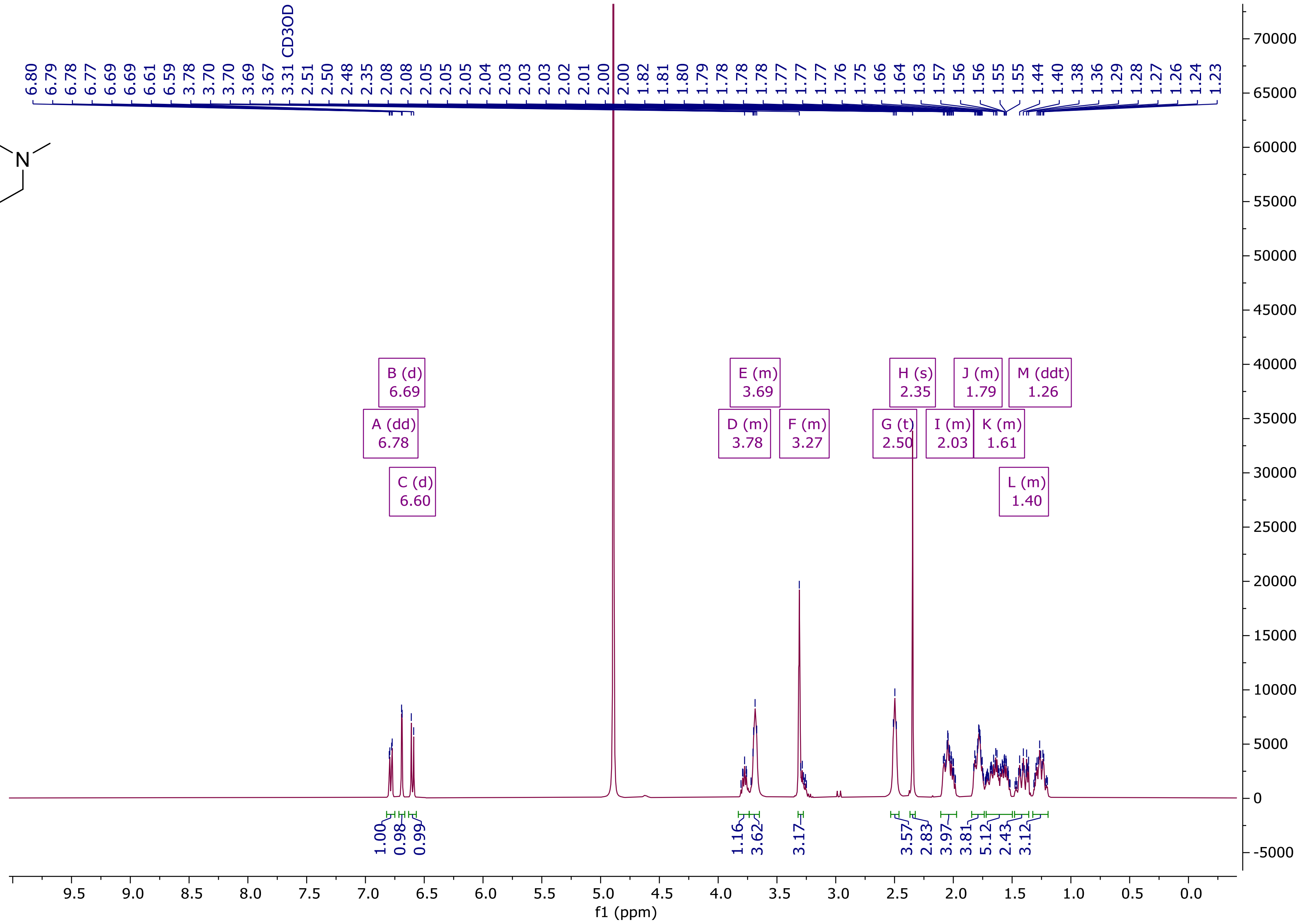

6.80 6.79 6.78 6.77 6.69 6.69 6.61 6.59 3.78 3.70 3.70 3.69 3.67 3.31 CD3OD 2.51 2.50 2.48 2.35 2.08 2.08 2.05 2.05 2.05 2.04 2.03 2.03 2.03 2.02 2.01 2.00 2.00 1.82 1.81 1.80 1.79 1.78 1.78 1.77 1.77 1.77 1.76 1.75 1.66 1.64 1.63 1.57 1.56 1.56 1.55 1.55 1.44 1.40 1.38 1.36 1.29 1.28 1.27 1.26 1.24 1.23
B (d) 6.69
A (dd) 6.78
C (d) 6.60
E (m) 3.69
D (m) 3.78
F (m) 3.27
H (s) 2.35
G (t) 2.50
I (m) 2.03
J (m) 1.79
K (m) 1.61
M (ddt) 1.26
L (m) 1.40
1.00 0.98 0.99 1.16 3.62 3.17 3.57 2.83 3.97 3.81 5.12 2.43 3.12
f1 (ppm)

Compound 40

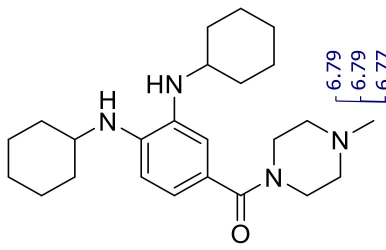

HN
H
N
N
N
O

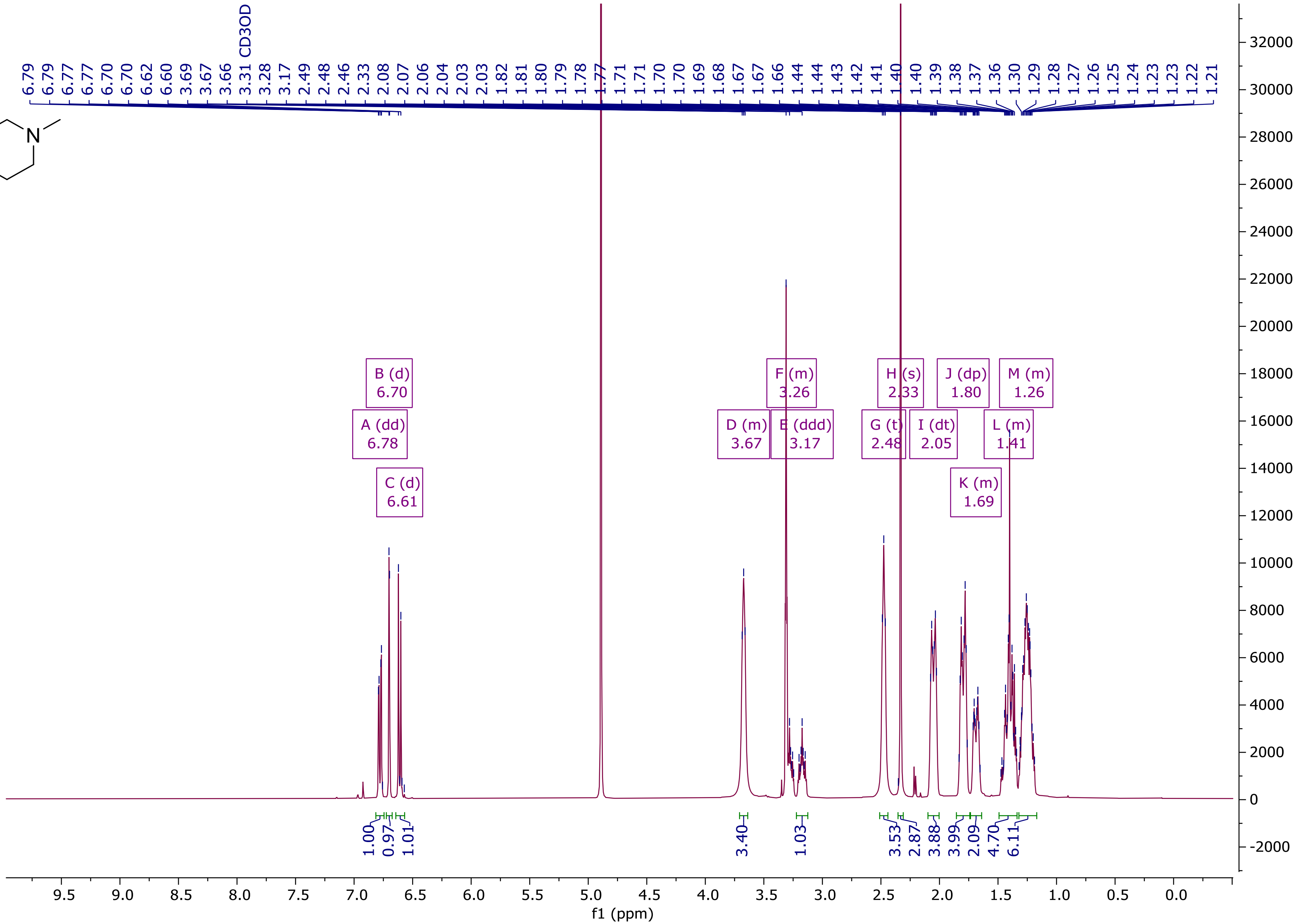

6.79 6.79 6.77 6.77 6.70 6.70 6.62 6.60 3.69 3.67 3.66 3.31 CD3OD 3.28 3.17 2.49 2.48 2.46 2.33 2.08 2.07 2.06 2.04 2.03 2.03 1.82 1.81 1.80 1.79 1.78 1.77 1.71 1.71 1.70 1.70 1.69 1.68 1.67 1.67 1.66 1.44 1.44 1.43 1.42 1.41 1.40 1.40 1.39 1.38 1.37 1.36 1.30 1.29 1.28 1.27 1.26 1.25 1.24 1.23 1.23 1.22 1.21
B (d) 6.70
A (dd) 6.78
C (d) 6.61
D (m) 3.67
F (m) 3.26
E (ddd) 3.17
H (s) 2.33
G (t) 2.48
I (dt) 2.05
J (dp) 1.80
K (m) 1.69
L (m) 1.41
M (m) 1.26
1.00 0.97 1.01 3.40 1.03 3.53 2.87 3.88 3.99 2.09 4.70 6.11
32000 30000 28000 26000 24000 22000 20000 18000 16000 14000 12000 10000 8000 6000 4000 2000 0 -2000
9.5 9.0 8.5 8.0 7.5 7.0 6.5 6.0 5.5 5.0 4.5 4.0 3.5 3.0 2.5 2.0 1.5 1.0 0.5 0.0
f1 (ppm)

Compound 41

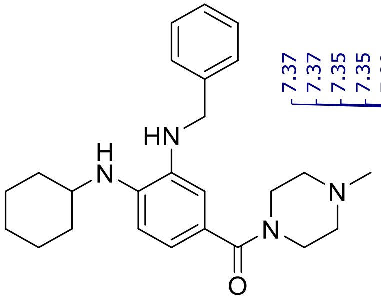

HN
H
N
N
N
O

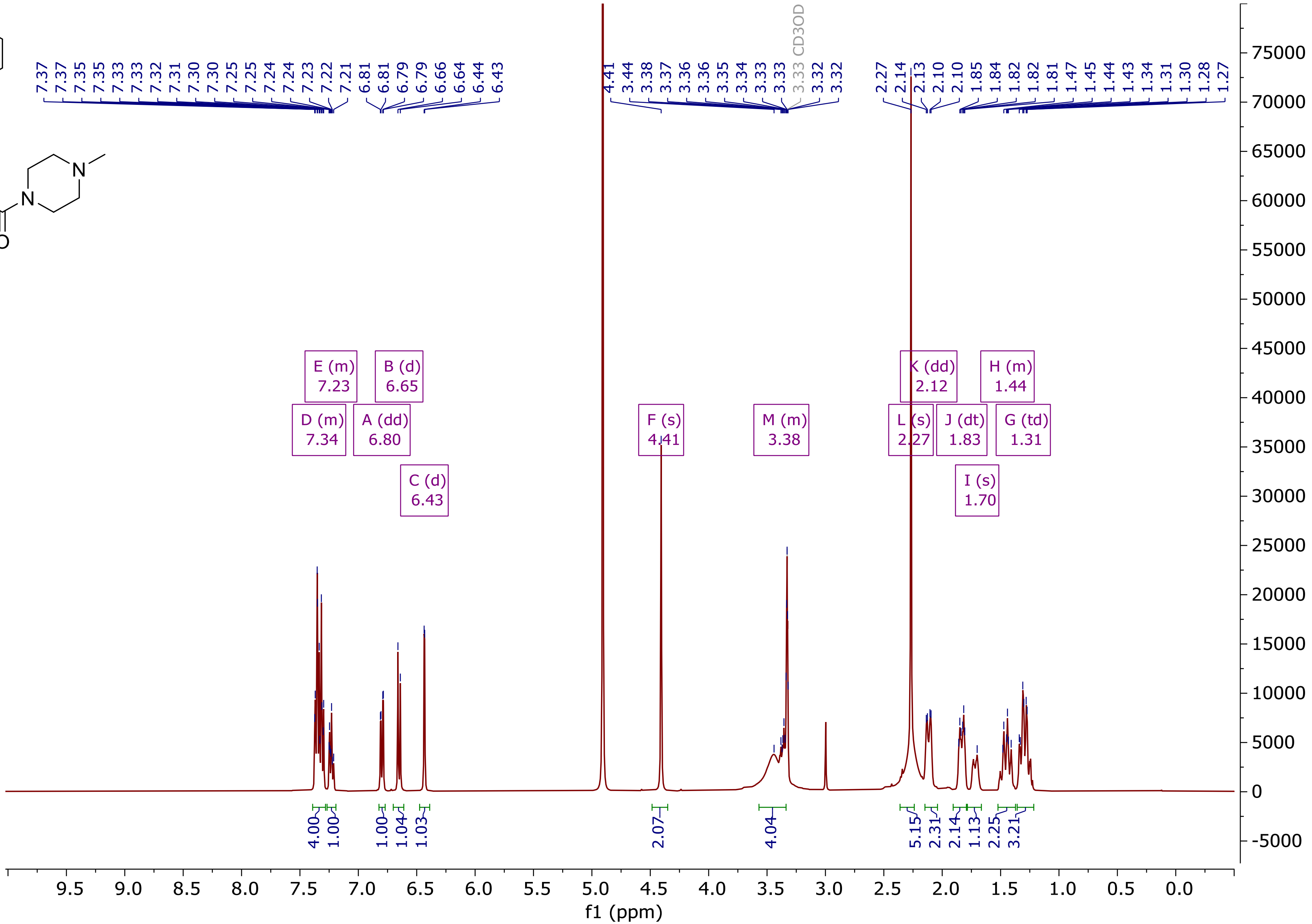

7.37 7.37 7.35 7.35 7.33 7.33 7.32 7.31 7.30 7.30 7.25 7.25 7.24 7.24 7.23 7.22 7.21 6.81 6.81 6.79 6.79 6.66 6.64 6.44 6.43
4.41 3.44 3.38 3.37 3.36 3.36 3.35 3.34 3.33 3.33 3.33 CD3OD 3.32 3.32
2.27 2.14 2.13 2.10 2.10 1.85 1.84 1.82 1.82 1.81 1.47 1.45 1.44 1.43 1.34 1.31 1.30 1.28 1.27
E (m) 7.23
B (d) 6.65
D (m) 7.34
A (dd) 6.80
C (d) 6.43
F (s) 4.41
M (m) 3.38
K (dd) 2.12
H (m) 1.44
L (s) 2.27
J (dt) 1.83
G (td) 1.31
I (s) 1.70
4.00 1.00 1.00 1.04 1.03 2.07 4.04 5.15 2.31 2.14 1.13 2.25 3.21
f1 (ppm)

Compound 42

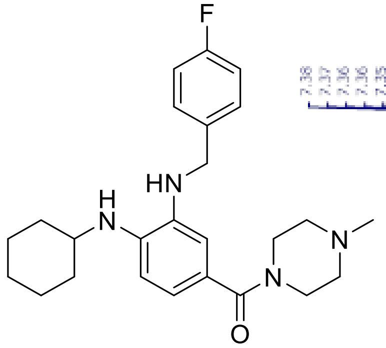
F
HN
H
N
N
N
O

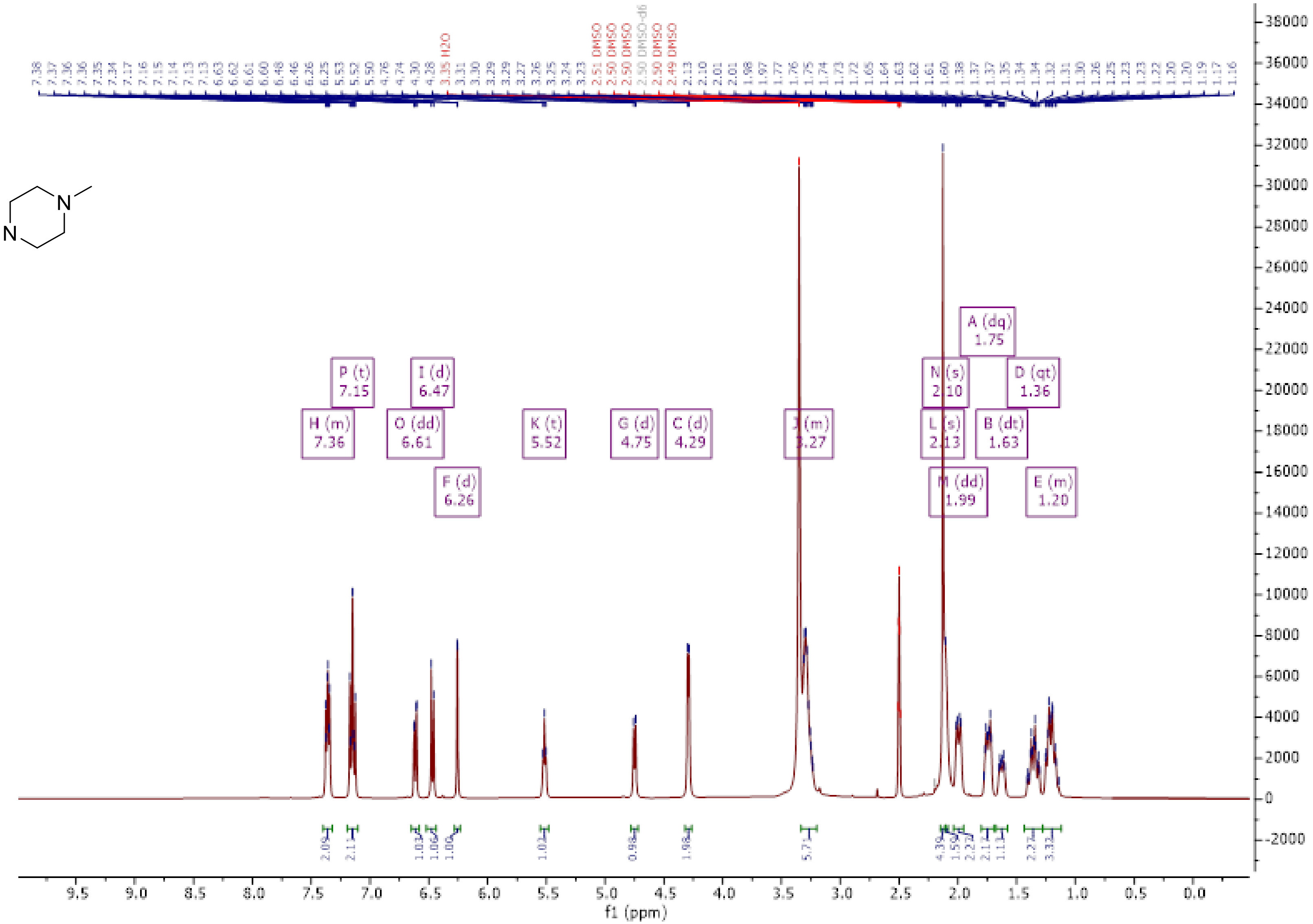
H (m)
7.36
P (t)
7.15
O (dd)
6.61
I (d)
6.47
F (d)
6.26
K (t)
5.52
G (d)
4.75
C (d)
4.29
J (m)
3.27
L (s)
2.13
N (s)
2.10
M (dd)
1.99
A (dq)
1.75
B (dt)
1.63
D (qt)
1.36
E (m)
1.20
f1 (ppm)

Compound 43

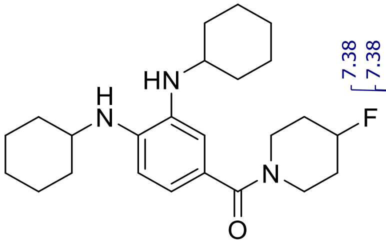

HN
H
N
F
N
O

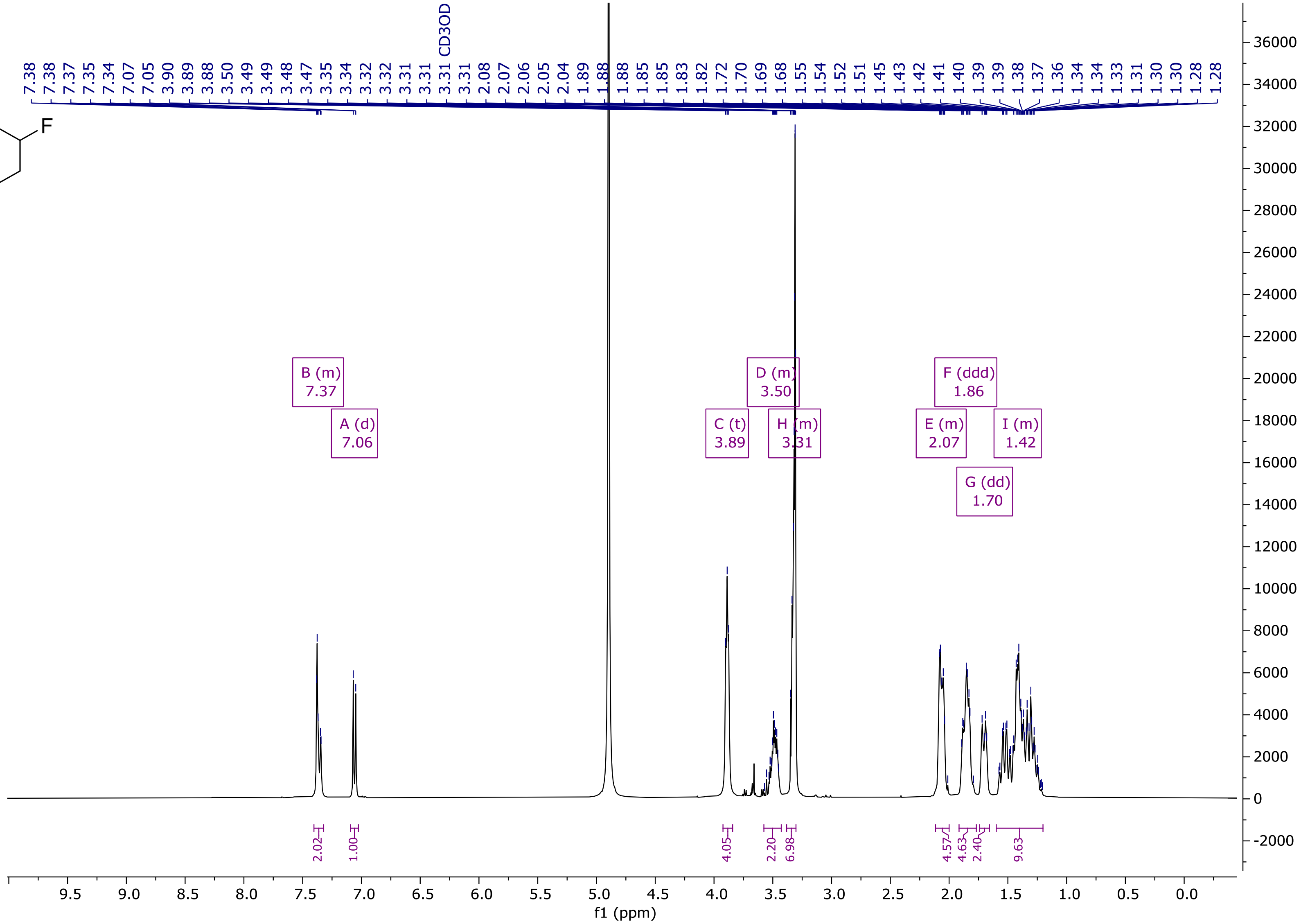

7.38 7.38 7.37 7.35 7.34 7.07 7.05 3.90 3.89 3.88 3.50 3.49 3.49 3.48 3.47 3.35 3.34 3.32 3.32 3.31 3.31 3.31 3.31 2.08 2.07 2.06 2.05 2.04 1.89 1.88 1.88 1.85 1.85 1.83 1.82 1.72 1.70 1.69 1.68 1.55 1.54 1.52 1.51 1.45 1.43 1.42 1.41 1.40 1.39 1.39 1.38 1.37 1.36 1.34 1.34 1.33 1.31 1.30 1.30 1.28 1.28
CD3OD
B (m) 7.37
A (d) 7.06
C (t) 3.89
D (m) 3.50
H (m) 3.31
F (ddd) 1.86
E (m) 2.07
I (m) 1.42
G (dd) 1.70
2.02
1.00
4.05
2.20
6.98
4.57
4.63
2.40
9.63
f1 (ppm)

Compound 44

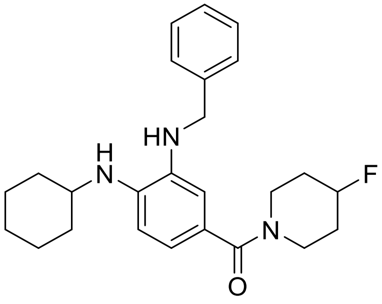

F
HN
H
N
N
O

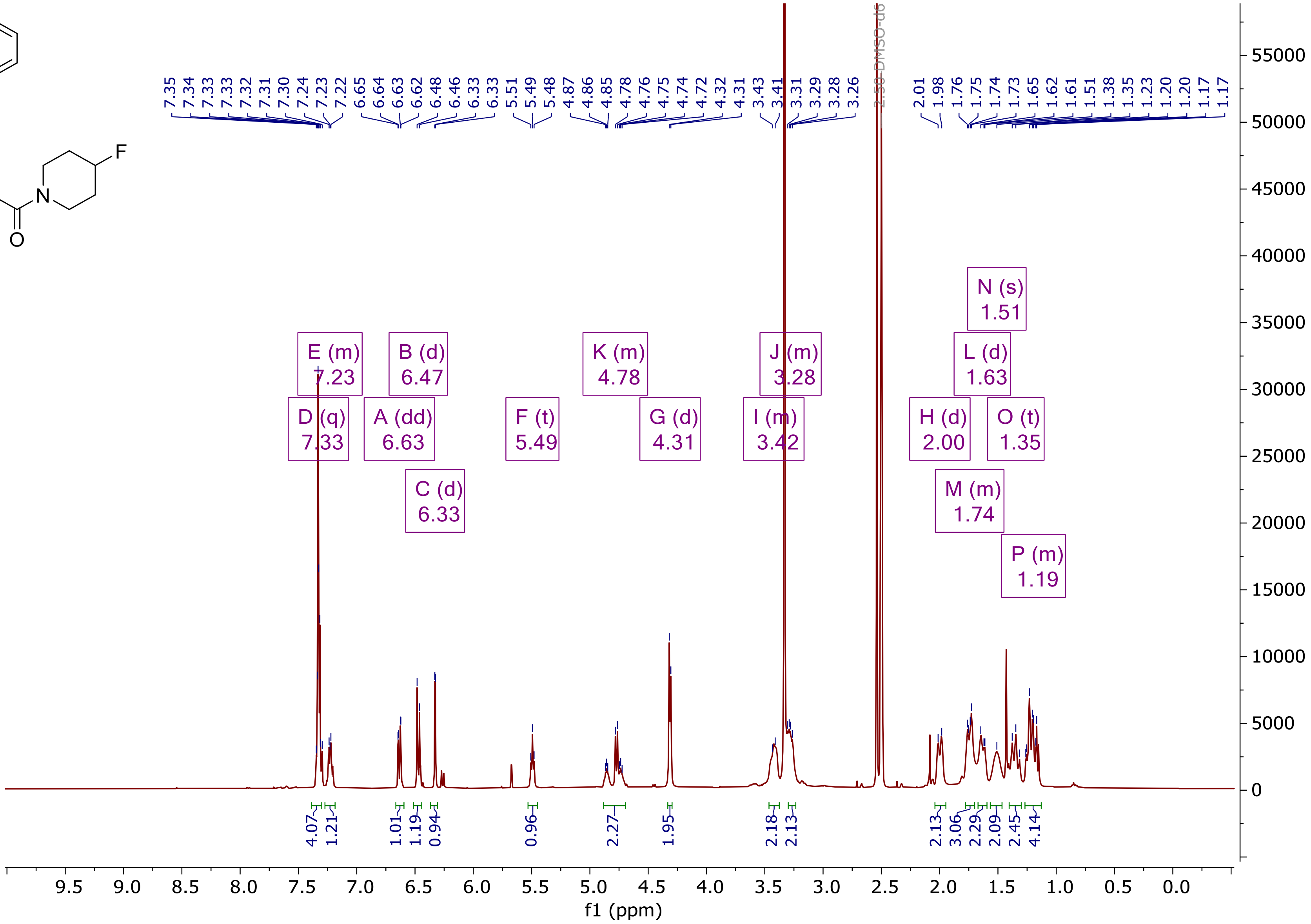

E (m) 7.23
B (d) 6.47
K (m) 4.78
J (m) 3.28
N (s) 1.51
L (d) 1.63
D (q) 7.33
A (dd) 6.63
F (t) 5.49
G (d) 4.31
I (m) 3.42
H (d) 2.00
O (t) 1.35
C (d) 6.33
M (m) 1.74
P (m) 1.19
2.50-DMSO-d6
f1 (ppm)

Compound 45

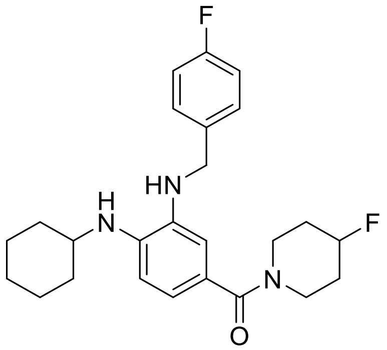

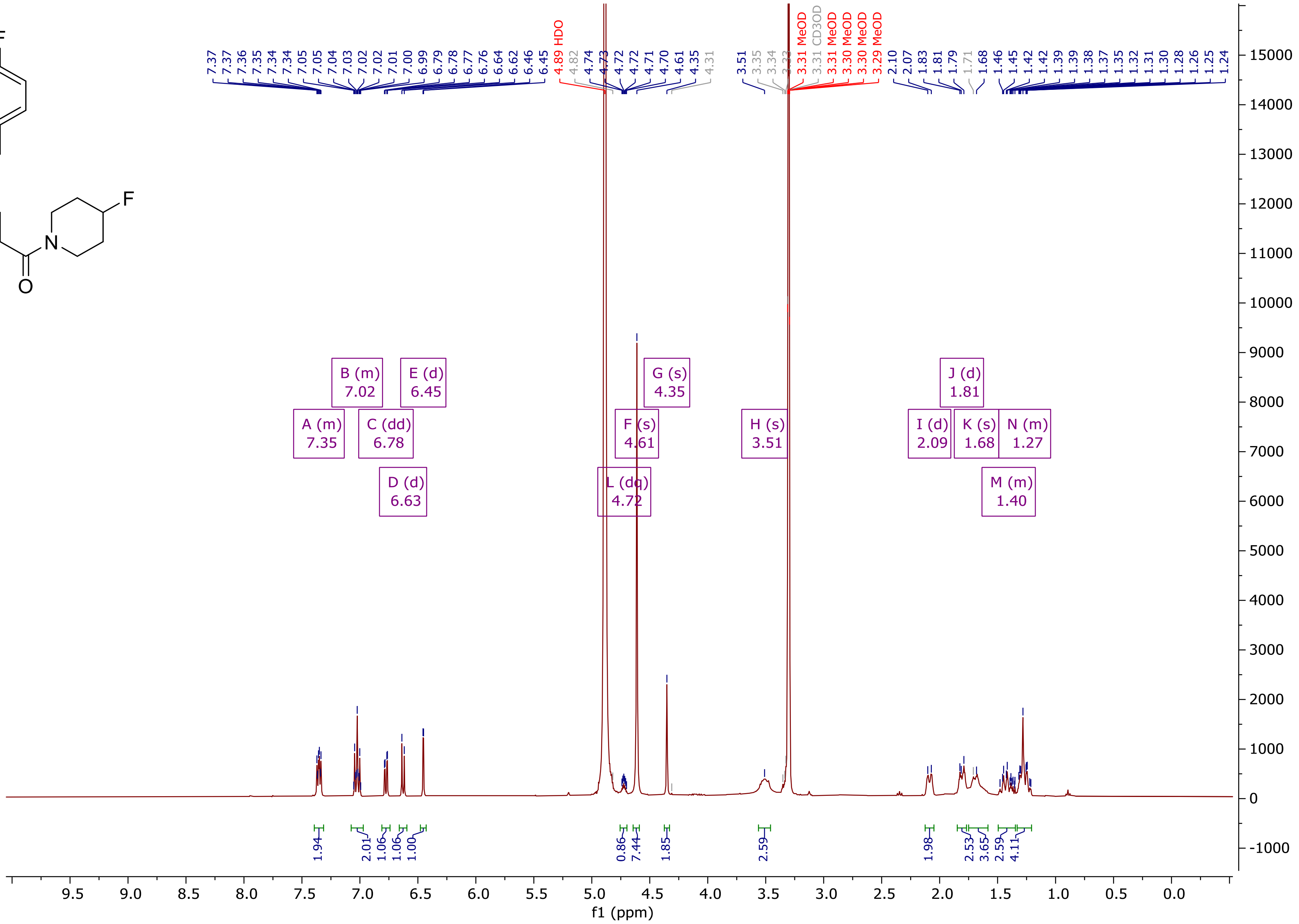

A (m)
7.35
B (m)
7.02
C (dd)
6.78
D (d)
6.63
E (d)
6.45
F (s)
4.61
G (s)
4.35
H (s)
3.51
I (d)
2.09
J (d)
1.81
K (s)
1.68
L (dq)
4.72
M (m)
1.40
N (m)
1.27
f1 (ppm)

Compound 46

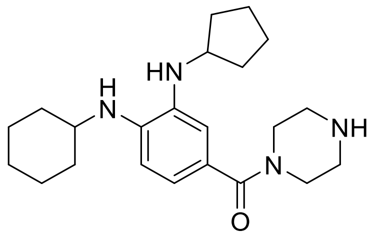

HN
H
N
NH
N
O

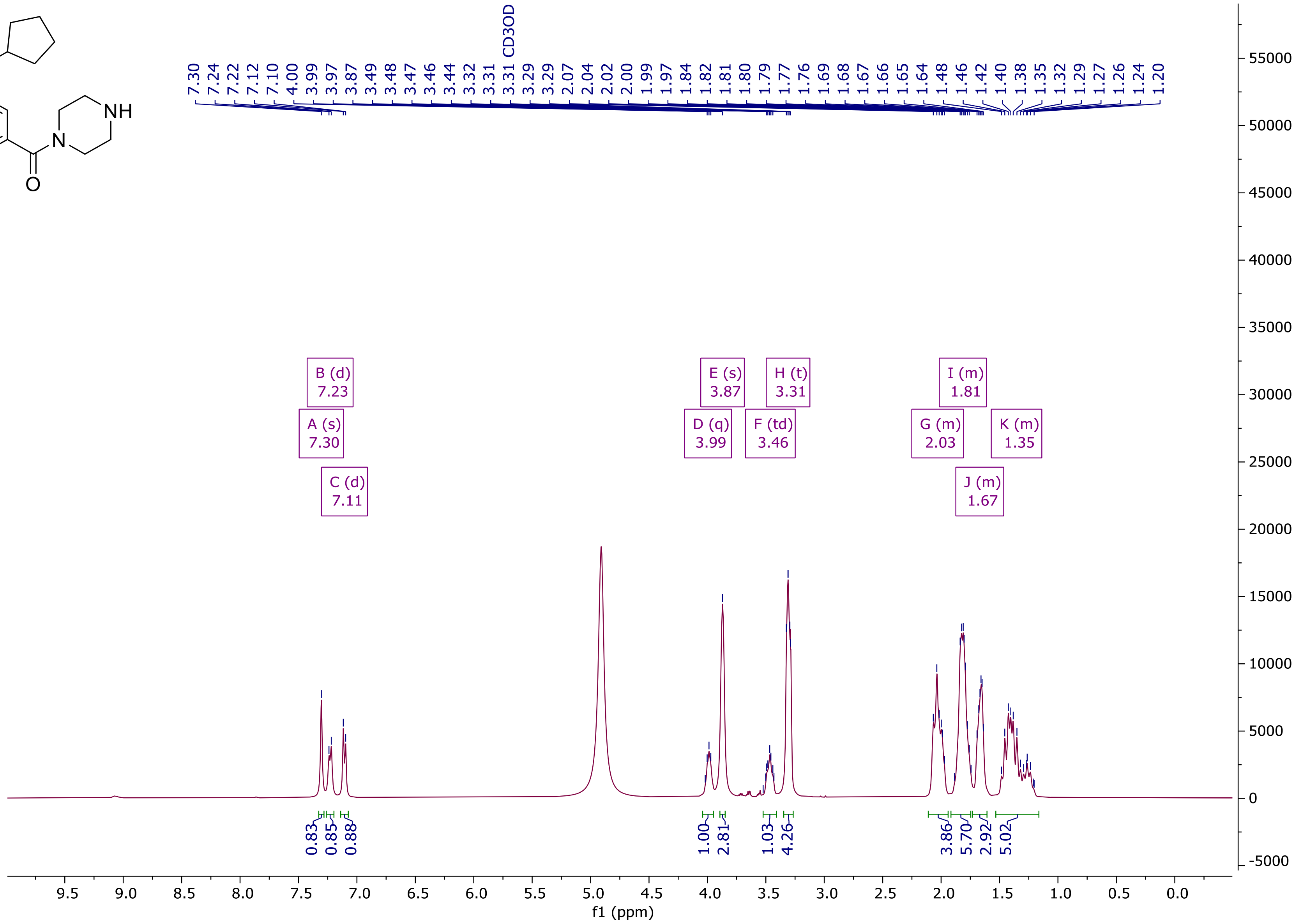

7.30 7.24 7.22 7.12 7.10 4.00 3.99 3.97 3.87 3.49 3.48 3.47 3.46 3.44 3.32 3.31 CD3OD 3.31 3.29 3.29 2.07 2.04 2.02 2.00 1.99 1.97 1.84 1.82 1.81 1.80 1.79 1.77 1.76 1.69 1.68 1.67 1.66 1.65 1.64 1.48 1.46 1.42 1.40 1.38 1.35 1.32 1.29 1.27 1.26 1.24 1.20
B (d) 7.23
A (s) 7.30
C (d) 7.11
E (s) 3.87
H (t) 3.31
D (q) 3.99
F (td) 3.46
I (m) 1.81
G (m) 2.03
K (m) 1.35
J (m) 1.67
0.83 0.85 0.88
1.00 2.81
1.03 4.26
3.86 5.70 2.92 5.02
9.5 9.0 8.5 8.0 7.5 7.0 6.5 6.0 5.5 5.0 4.5 4.0 3.5 3.0 2.5 2.0 1.5 1.0 0.5 0.0
f1 (ppm)
55000 50000 45000 40000 35000 30000 25000 20000 15000 10000 5000 0 -5000

Compound 47

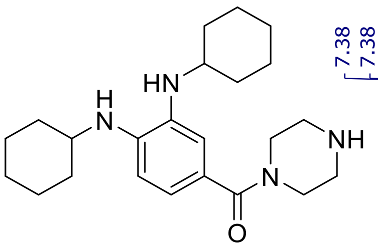

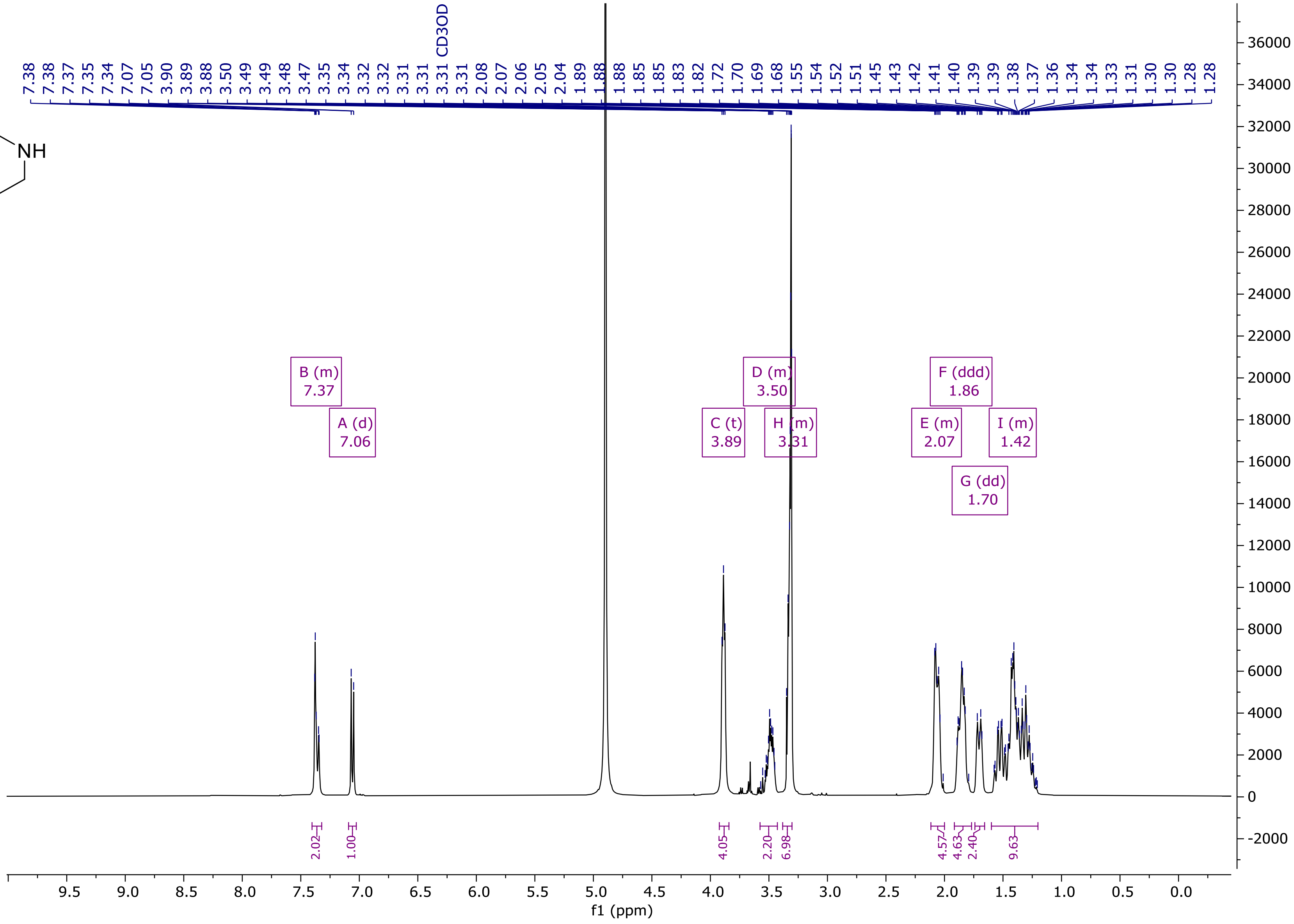

7.38 7.38 7.37 7.35 7.34 7.07 7.05 3.90 3.89 3.88 3.50 3.49 3.49 3.48 3.47 3.35 3.34 3.32 3.32 3.31 3.31 3.31 3.31 2.08 2.07 2.06 2.05 2.04 1.89 1.88 1.88 1.85 1.85 1.83 1.82 1.72 1.70 1.69 1.68 1.55 1.54 1.52 1.51 1.45 1.43 1.42 1.41 1.40 1.39 1.39 1.38 1.37 1.36 1.34 1.34 1.33 1.31 1.30 1.30 1.28 1.28
CD3OD
B (m) 7.37
A (d) 7.06
C (t) 3.89
D (m) 3.50
H (m) 3.31
E (m) 2.07
F (ddd) 1.86
G (dd) 1.70
I (m) 1.42
2.02
1.00
4.05
2.20
6.98
4.57
4.63
2.40
9.63
f1 (ppm)

Compound 48

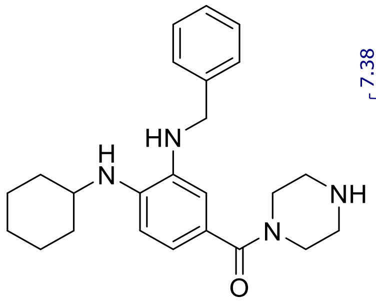

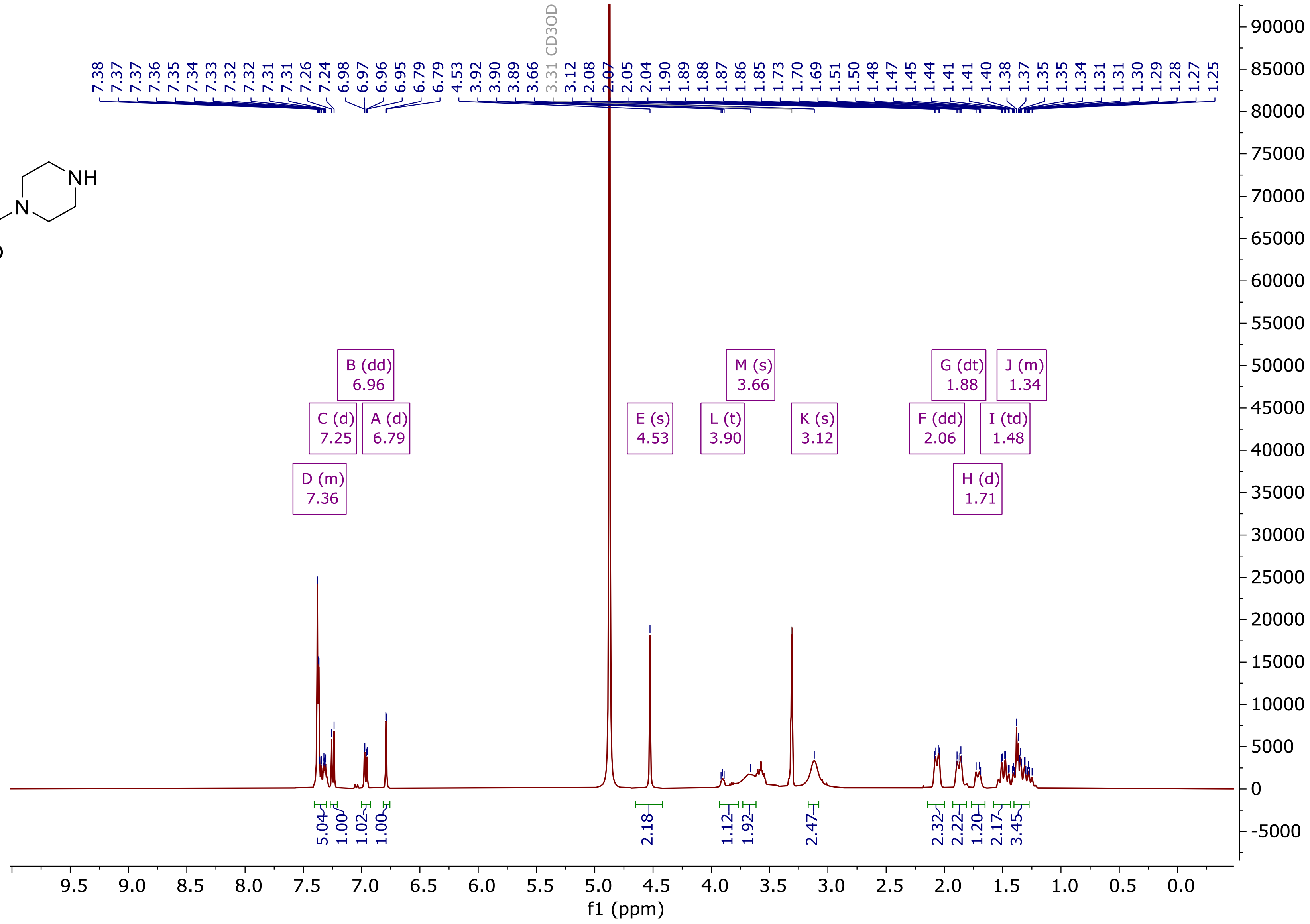

CD3OD
B (dd)
6.96
C (d)
7.25
A (d)
6.79
D (m)
7.36
E (s)
4.53
L (t)
3.90
M (s)
3.66
K (s)
3.12
F (dd)
2.06
G (dt)
1.88
H (d)
1.71
I (td)
1.48
J (m)
1.34
f1 (ppm)

Compound 49

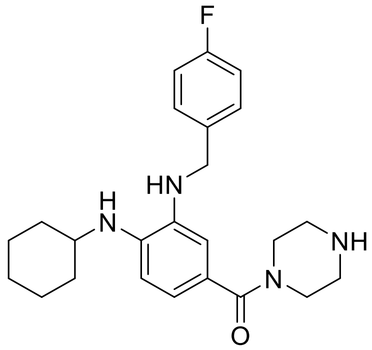

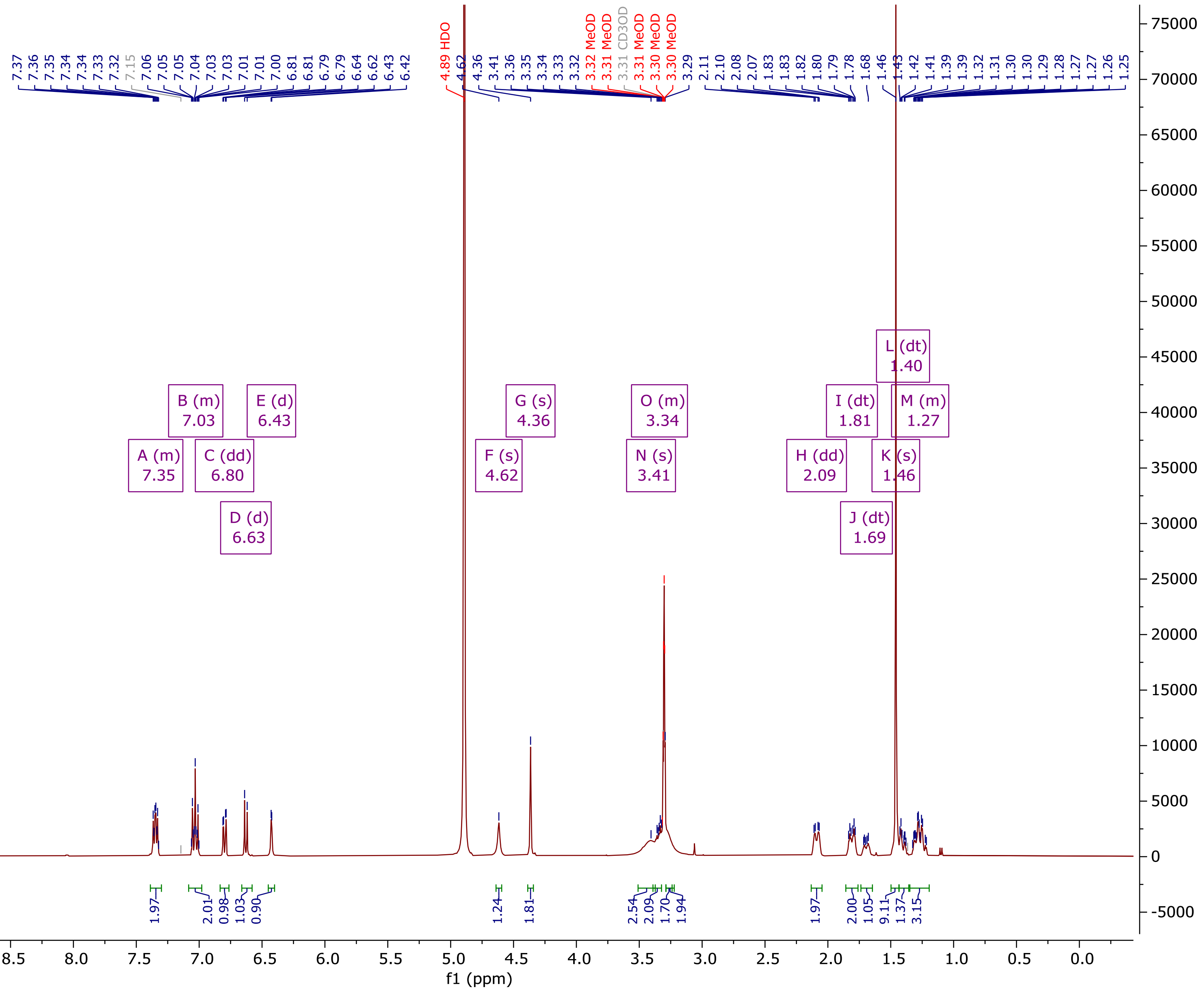

Compound 50

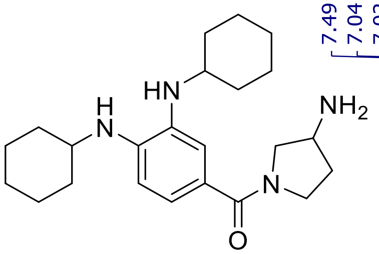
HN
H
N
NH2
N
O

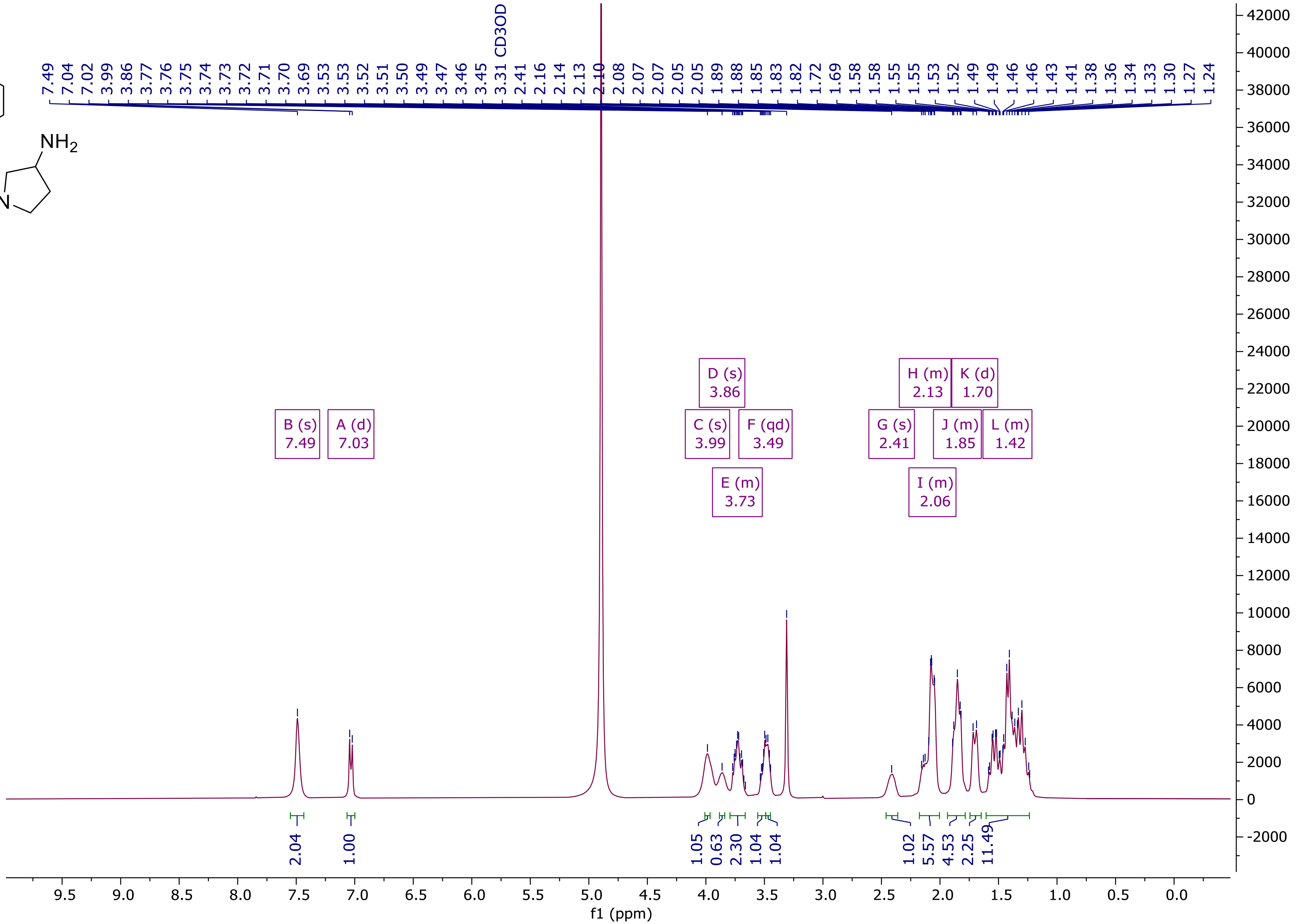
7.49
7.04
7.02
3.99
3.86
3.77
3.76
3.75
3.74
3.73
3.72
3.71
3.70
3.69
3.53
3.53
3.52
3.51
3.50
3.49
3.47
3.46
3.45
3.31 CD3OD
2.41
2.16
2.14
2.13
2.10
2.08
2.07
2.07
2.05
2.05
1.89
1.88
1.85
1.83
1.82
1.72
1.69
1.58
1.58
1.55
1.55
1.53
1.52
1.49
1.49
1.46
1.46
1.43
1.41
1.38
1.36
1.34
1.33
1.30
1.27
1.24
B (s) 7.49
A (d) 7.03
D (s) 3.86
C (s) 3.99
F (qd) 3.49
E (m) 3.73
H (m) 2.13
K (d) 1.70
G (s) 2.41
J (m) 1.85
L (m) 1.42
I (m) 2.06
2.04
1.00
1.05
0.63
2.30
1.04
1.04
1.02
5.57
4.53
2.25
11.49
f1 (ppm)

Compound 51

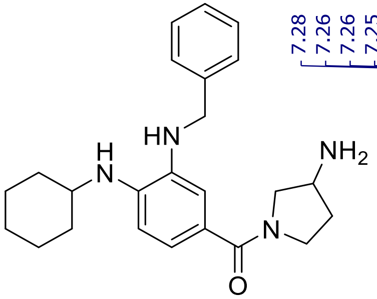
HN
H
N
NH2
N
O

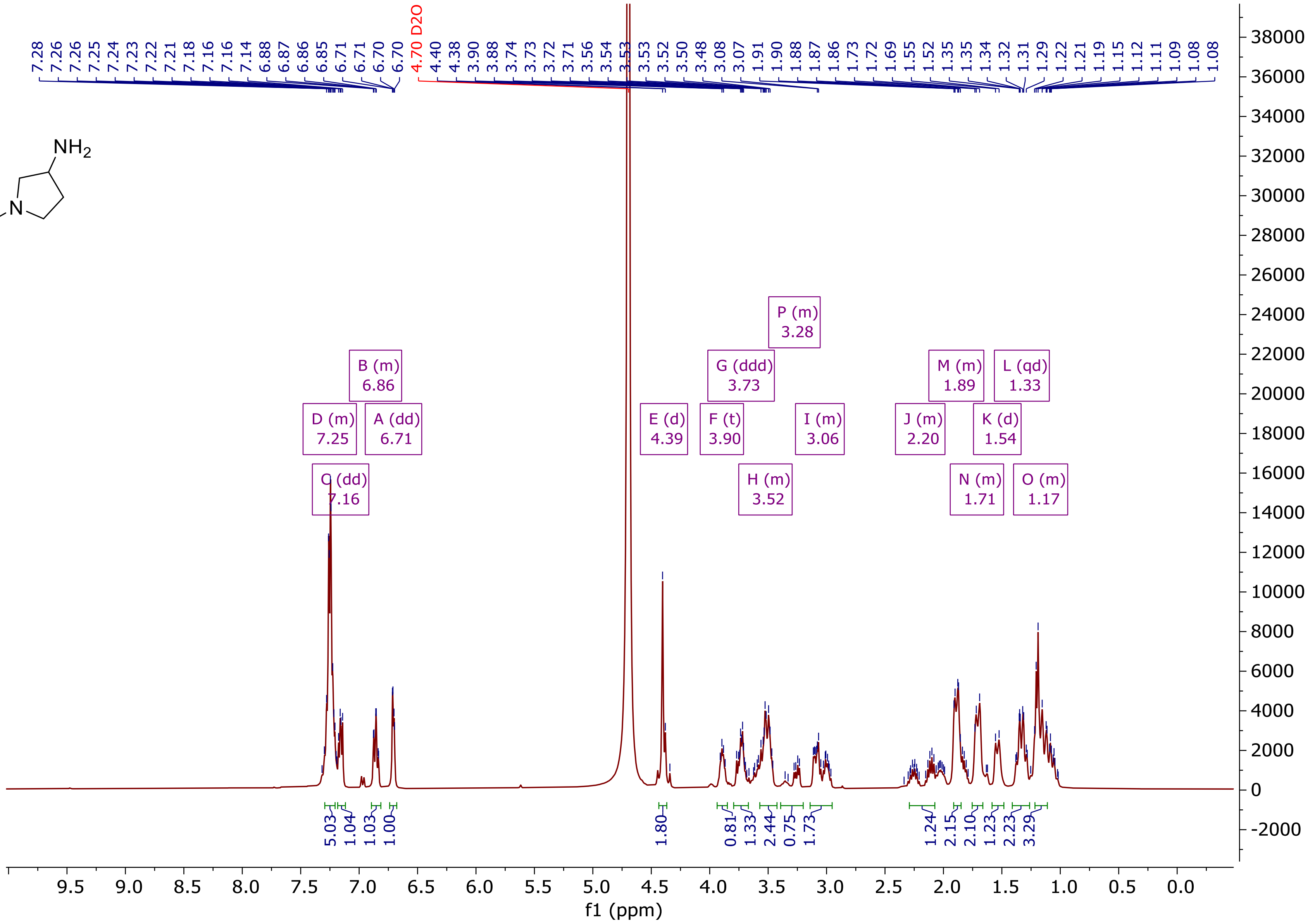
7.28
7.26
7.26
7.25
7.24
7.23
7.22
7.21
7.18
7.16
7.16
7.14
6.88
6.87
6.86
6.85
6.71
6.71
6.70
6.70
4.70 D2O
4.40
4.38
3.90
3.88
3.74
3.73
3.72
3.71
3.56
3.54
3.53
3.53
3.52
3.50
3.48
3.08
3.07
1.91
1.90
1.88
1.87
1.86
1.73
1.72
1.69
1.55
1.52
1.35
1.35
1.34
1.32
1.31
1.29
1.22
1.21
1.19
1.15
1.12
1.11
1.09
1.08
1.08
P (m) 3.28
B (m) 6.86
G (ddd) 3.73
M (m) 1.89
L (qd) 1.33
D (m) 7.25
A (dd) 6.71
E (d) 4.39
F (t) 3.90
I (m) 3.06
J (m) 2.20
K (d) 1.54
C (dd) 7.16
H (m) 3.52
N (m) 1.71
O (m) 1.17
5.03
1.04
1.03
1.00
1.80
0.81
1.33
2.44
0.75
1.73
1.24
2.15
2.10
1.23
2.23
3.29
f1 (ppm)

Compound 73

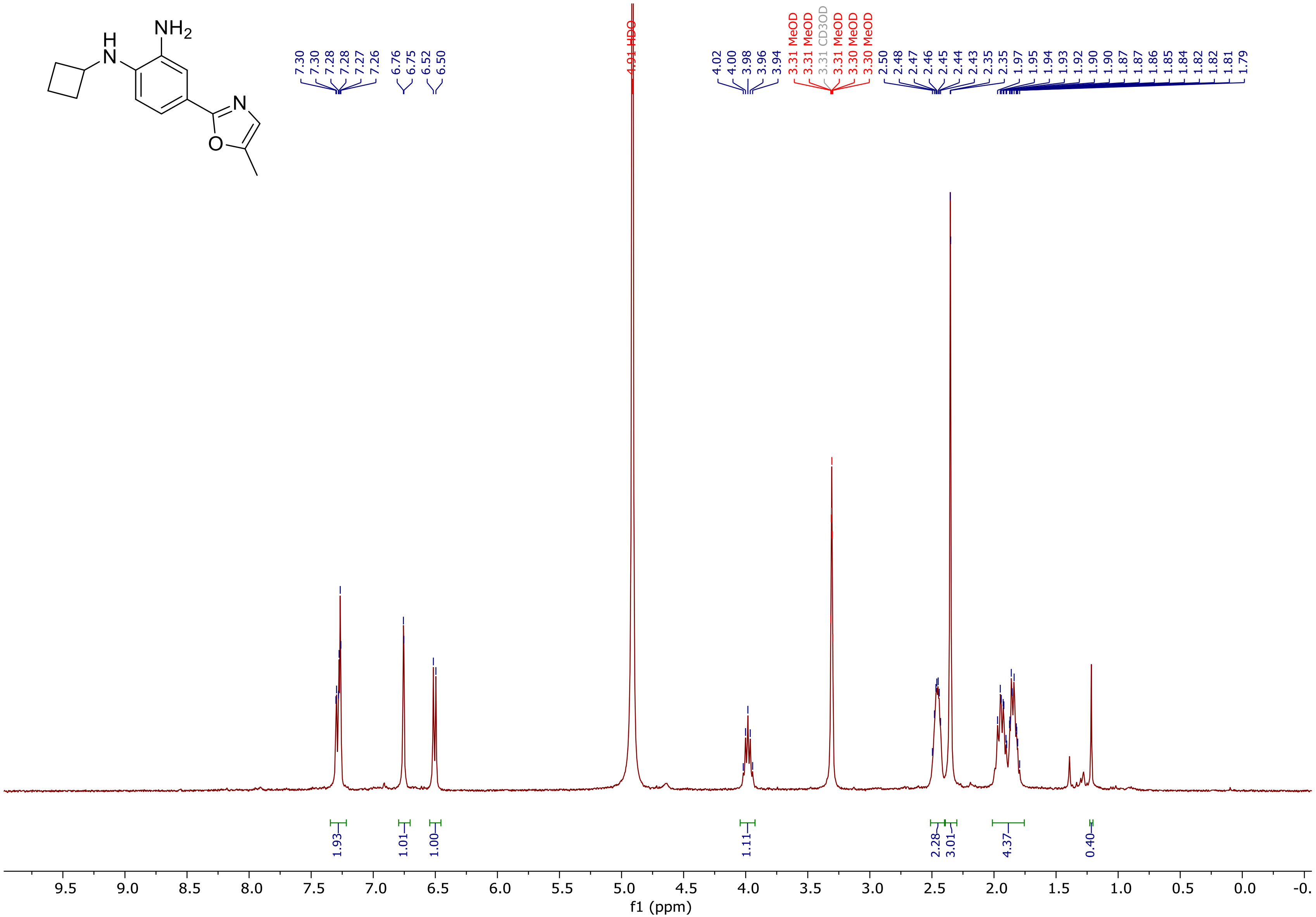
NH2
H
N
N
O
7.30
7.30
7.28
7.28
7.27
7.26
6.76
6.75
6.52
6.50
4.91 HDO
4.02
4.00
3.98
3.96
3.94
3.31 MeOD
3.31 MeOD
3.31 CD3OD
3.31 MeOD
3.30 MeOD
3.30 MeOD
2.50
2.48
2.47
2.46
2.45
2.44
2.43
2.35
2.35
1.97
1.95
1.94
1.93
1.92
1.90
1.90
1.87
1.87
1.86
1.85
1.84
1.82
1.82
1.81
1.79
1.93
1.01
1.00
1.11
2.28
3.01
4.37
0.40
9.5
9.0
8.5
8.0
7.5
7.0
6.5
6.0
5.5
5.0
4.5
4.0
3.5
3.0
2.5
2.0
1.5
1.0
0.5
0.0
-0.
f1 (ppm)

Compound 74

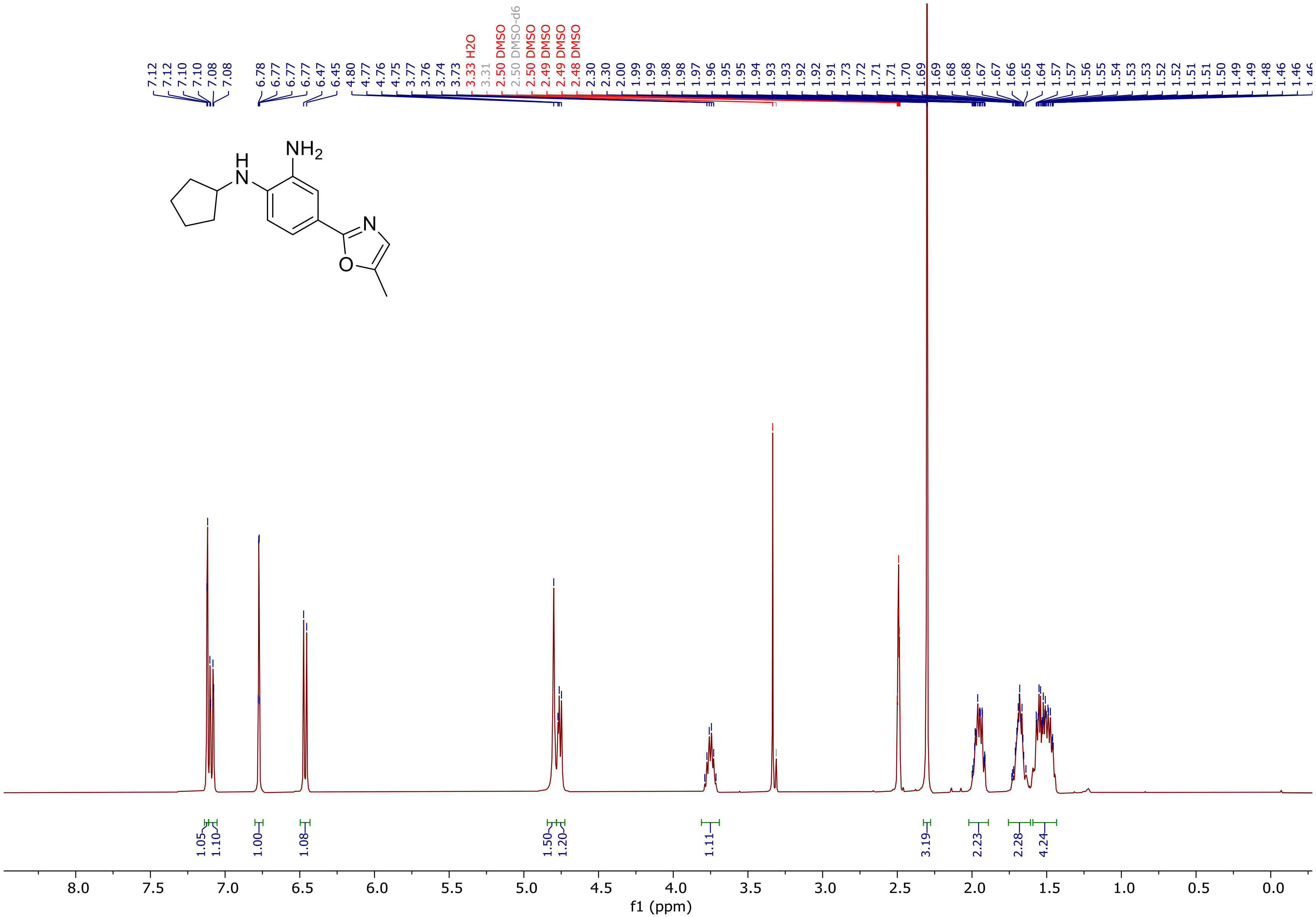

Compound 75

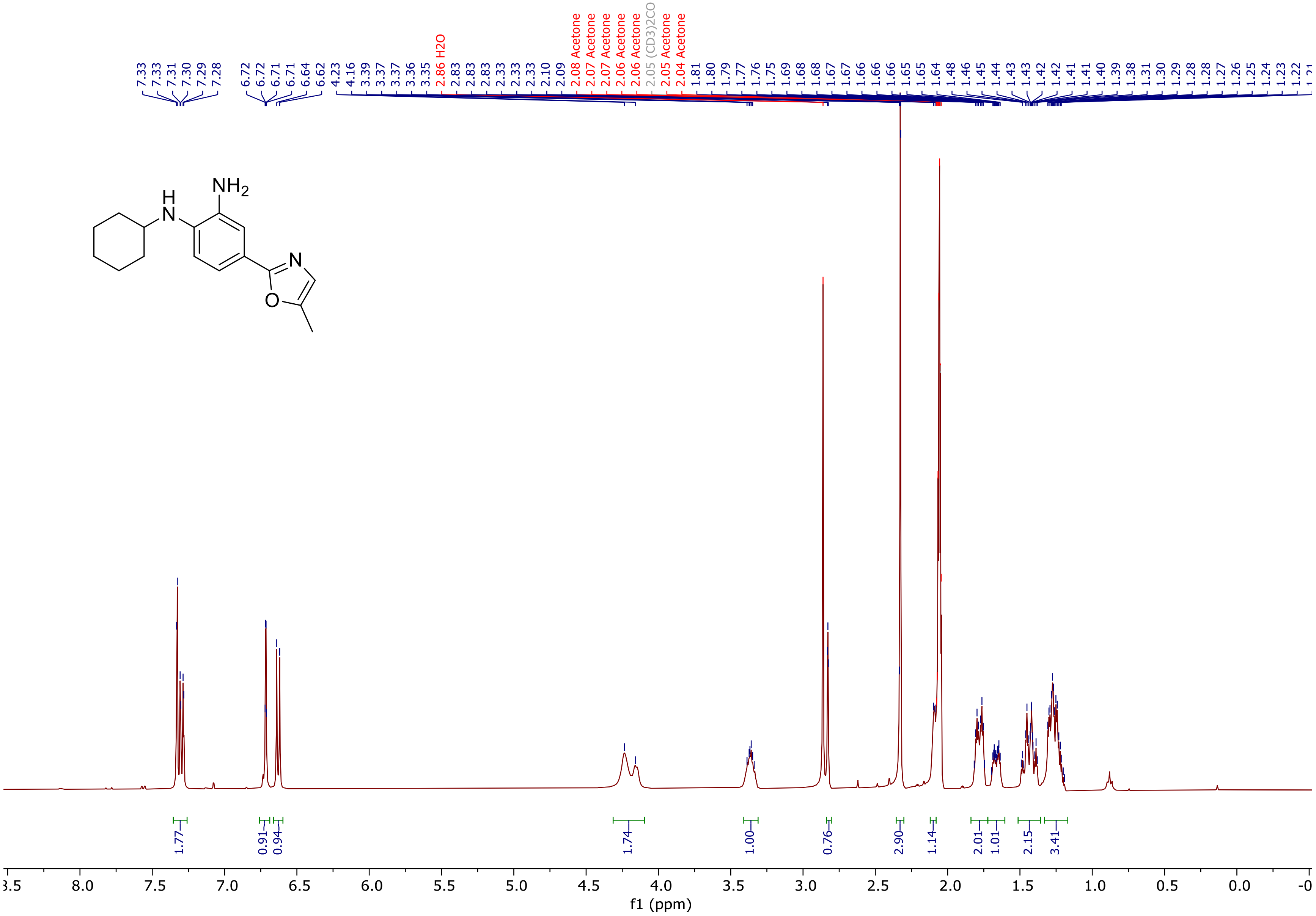

Compound 76

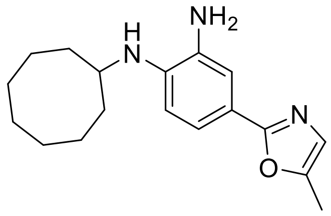

NH2
H
N
N
O

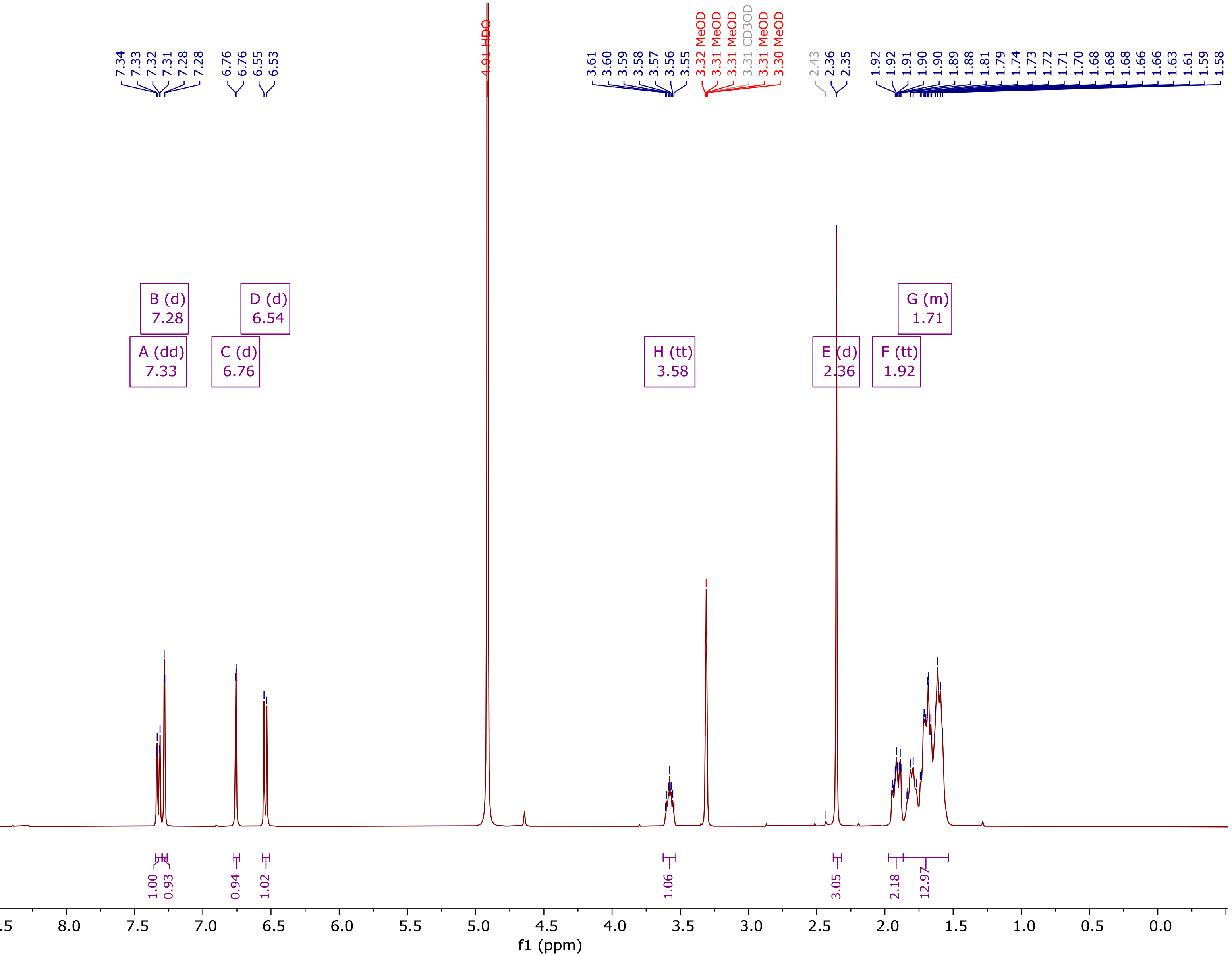

7.34 7.33 7.32 7.31 7.28 7.28
6.76 6.76 6.55 6.53
4.91 HDO
3.61 3.60 3.59 3.58 3.57 3.56 3.55
3.32 MeOD 3.31 MeOD 3.31 MeOD 3.31 CD3OD 3.31 MeOD 3.30 MeOD
2.43 2.36 2.35
1.92 1.92 1.91 1.90 1.90 1.89 1.88 1.81 1.79 1.74 1.73 1.72 1.71 1.70 1.68 1.68 1.68 1.66 1.66 1.63 1.61 1.59 1.58
B (d) 7.28
D (d) 6.54
G (m) 1.71
A (dd) 7.33
C (d) 6.76
H (tt) 3.58
E (d) 2.36
F (tt) 1.92
1.00 0.93 0.94 1.02 1.06 3.05 2.18 12.97
9.0 8.5 8.0 7.5 7.0 6.5 6.0 5.5 5.0 4.5 4.0 3.5 3.0 2.5 2.0 1.5 1.0 0.5 0.0
f1 (ppm)

Compound 77

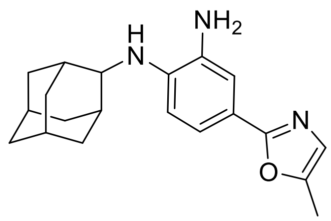

H
N
NH2
N
O

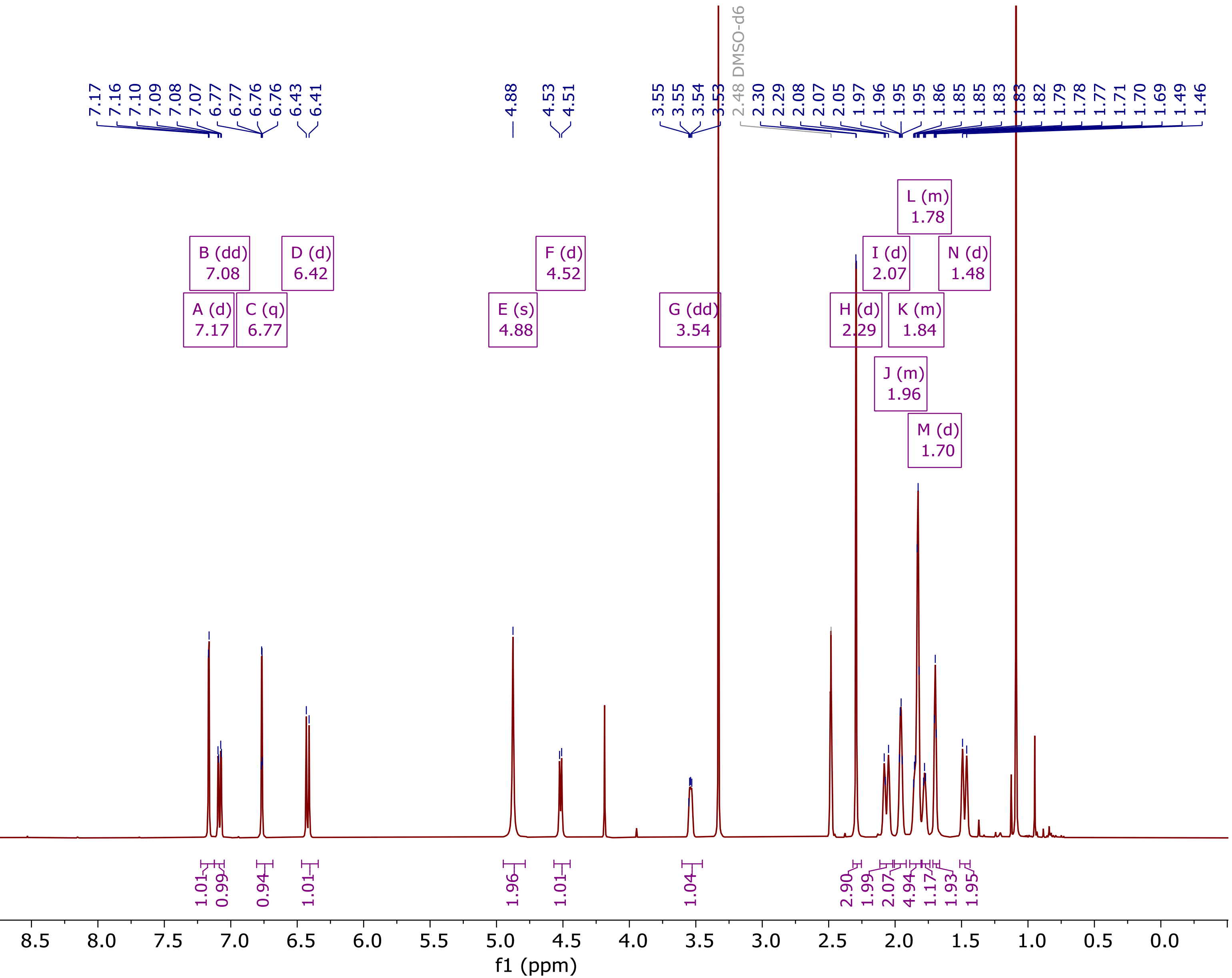

7.17
7.16
7.10
7.09
7.08
7.07
6.77
6.77
6.76
6.76
6.43
6.41
4.88
4.53
4.51
3.55
3.55
3.54
3.53
2.48 DMSO-d6
2.30
2.29
2.08
2.07
2.05
1.97
1.96
1.95
1.95
1.86
1.85
1.85
1.83
1.83
1.82
1.79
1.78
1.77
1.71
1.70
1.69
1.49
1.46
L (m) 1.78
B (dd) 7.08
D (d) 6.42
F (d) 4.52
I (d) 2.07
N (d) 1.48
A (d) 7.17
C (q) 6.77
E (s) 4.88
G (dd) 3.54
H (d) 2.29
K (m) 1.84
J (m) 1.96
M (d) 1.70
1.01
0.99
0.94
1.01
1.96
1.01
1.04
2.90
1.99
2.07
4.94
1.17
1.93
1.95
9.5
9.0
8.5
8.0
7.5
7.0
6.5
6.0
5.5
5.0
4.5
4.0
3.5
3.0
2.5
2.0
1.5
1.0
0.5
0.0
f1 (ppm)

Compound 78

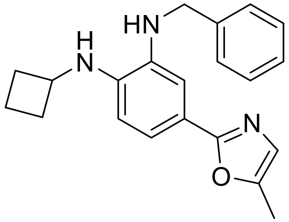

HN
H
N
N
O

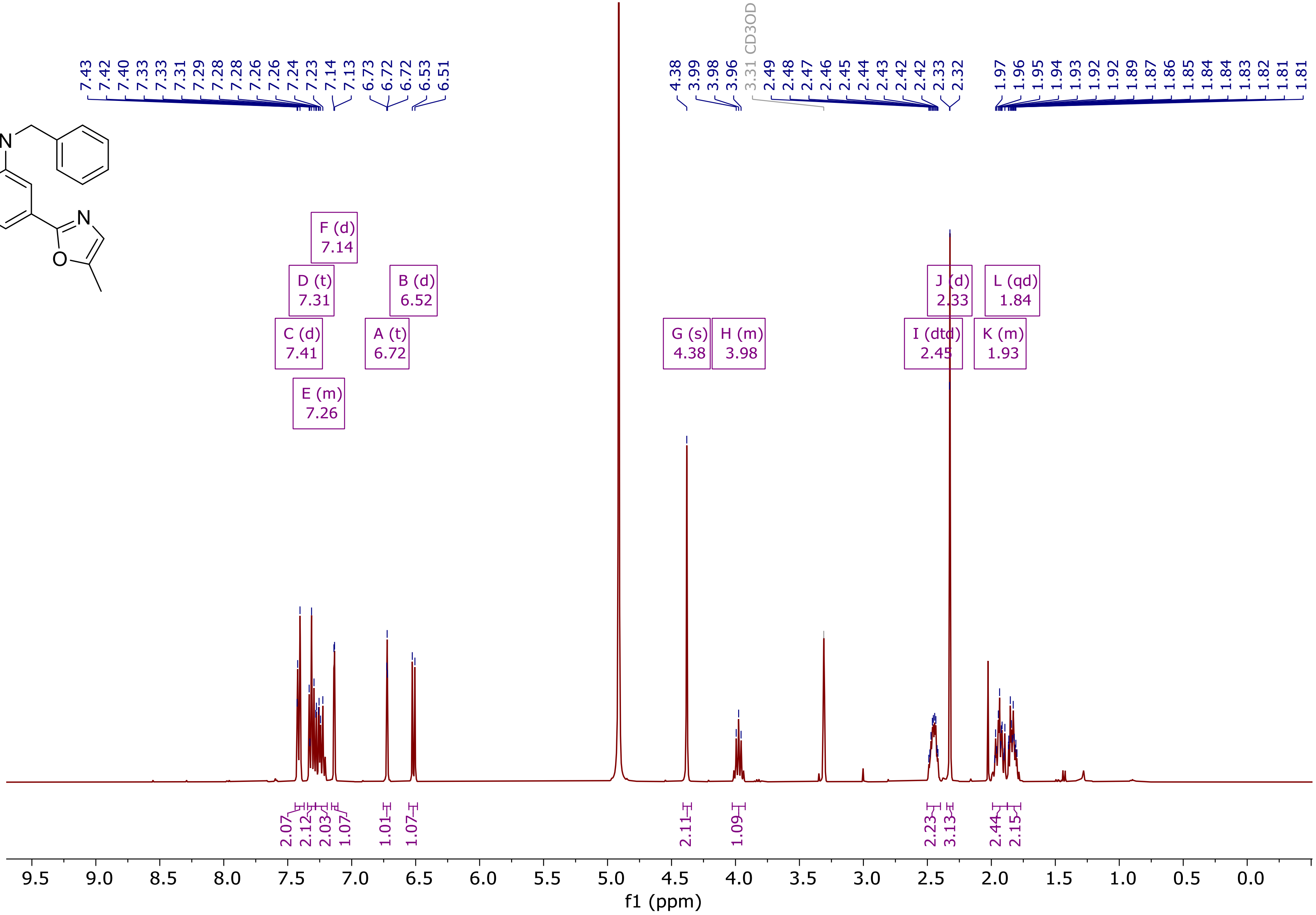

7.43 7.42 7.40 7.33 7.33 7.31 7.29 7.28 7.28 7.26 7.26 7.24 7.23 7.14 7.13 6.73 6.72 6.72 6.53 6.51
4.38 3.99 3.98 3.96 3.31 CD3OD 2.49 2.48 2.47 2.46 2.45 2.44 2.43 2.42 2.42 2.33 2.32
1.97 1.96 1.95 1.94 1.93 1.92 1.92 1.89 1.87 1.86 1.85 1.84 1.84 1.83 1.82 1.81 1.81 1.81
F (d) 7.14
D (t) 7.31
C (d) 7.41
E (m) 7.26
B (d) 6.52
A (t) 6.72
G (s) 4.38
H (m) 3.98
J (d) 2.33
I (dtd) 2.45
L (qd) 1.84
K (m) 1.93
2.07 2.12 2.03 1.07 1.01 1.07 2.11 1.09 2.23 3.13 2.44 2.15
9.5 9.0 8.5 8.0 7.5 7.0 6.5 6.0 5.5 5.0 4.5 4.0 3.5 3.0 2.5 2.0 1.5 1.0 0.5 0.0
f1 (ppm)

Compound 79

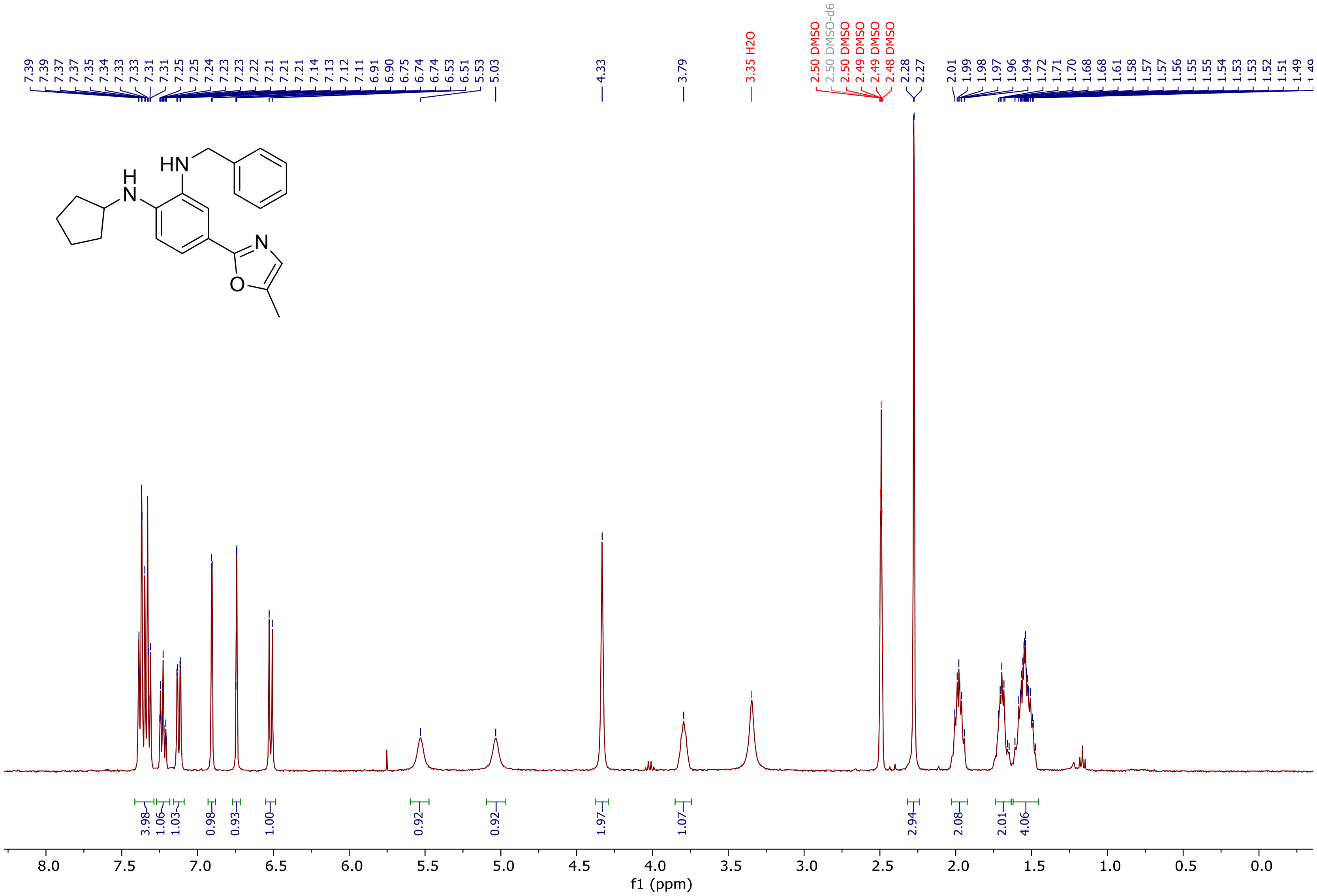

Compound 80

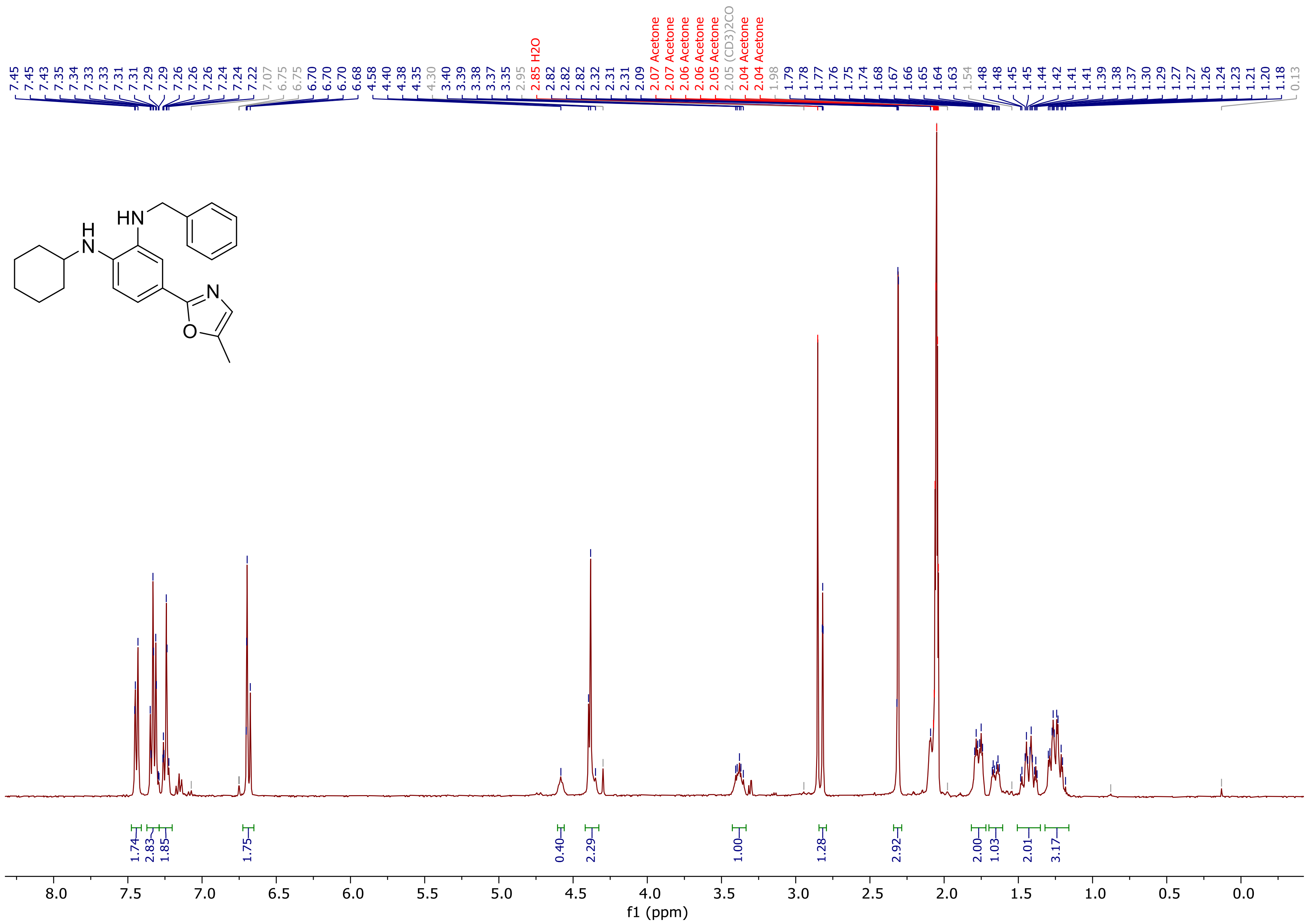

HN
H
N
N
O
7.45 7.45 7.43 7.35 7.34 7.33 7.33 7.31 7.31 7.29 7.29 7.26 7.26 7.24 7.24 7.22 7.07 6.75 6.75 6.70 6.70 6.68 4.58 4.40 4.38 4.35 4.30 3.40 3.39 3.38 3.37 3.35 2.95
2.85 H2O
2.82 2.82 2.82 2.32 2.31 2.31 2.09
2.07 Acetone
2.07 Acetone
2.06 Acetone
2.06 Acetone
2.05 Acetone
2.05 (CD3)2CO
2.04 Acetone
2.04 Acetone
1.98 1.79 1.78 1.77 1.76 1.75 1.74 1.68 1.67 1.66 1.65 1.64 1.63 1.54 1.48 1.48 1.45 1.45 1.44 1.42 1.41 1.41 1.39 1.38 1.37 1.30 1.29 1.27 1.27 1.26 1.24 1.23 1.21 1.20 1.18 0.13
1.74 2.83 1.85 1.75 0.40 2.29 1.00 1.28 2.92 2.00 1.03 2.01 3.17
8.0 7.5 7.0 6.5 6.0 5.5 5.0 4.5 4.0 3.5 3.0 2.5 2.0 1.5 1.0 0.5 0.0
f1 (ppm)

Compound 81

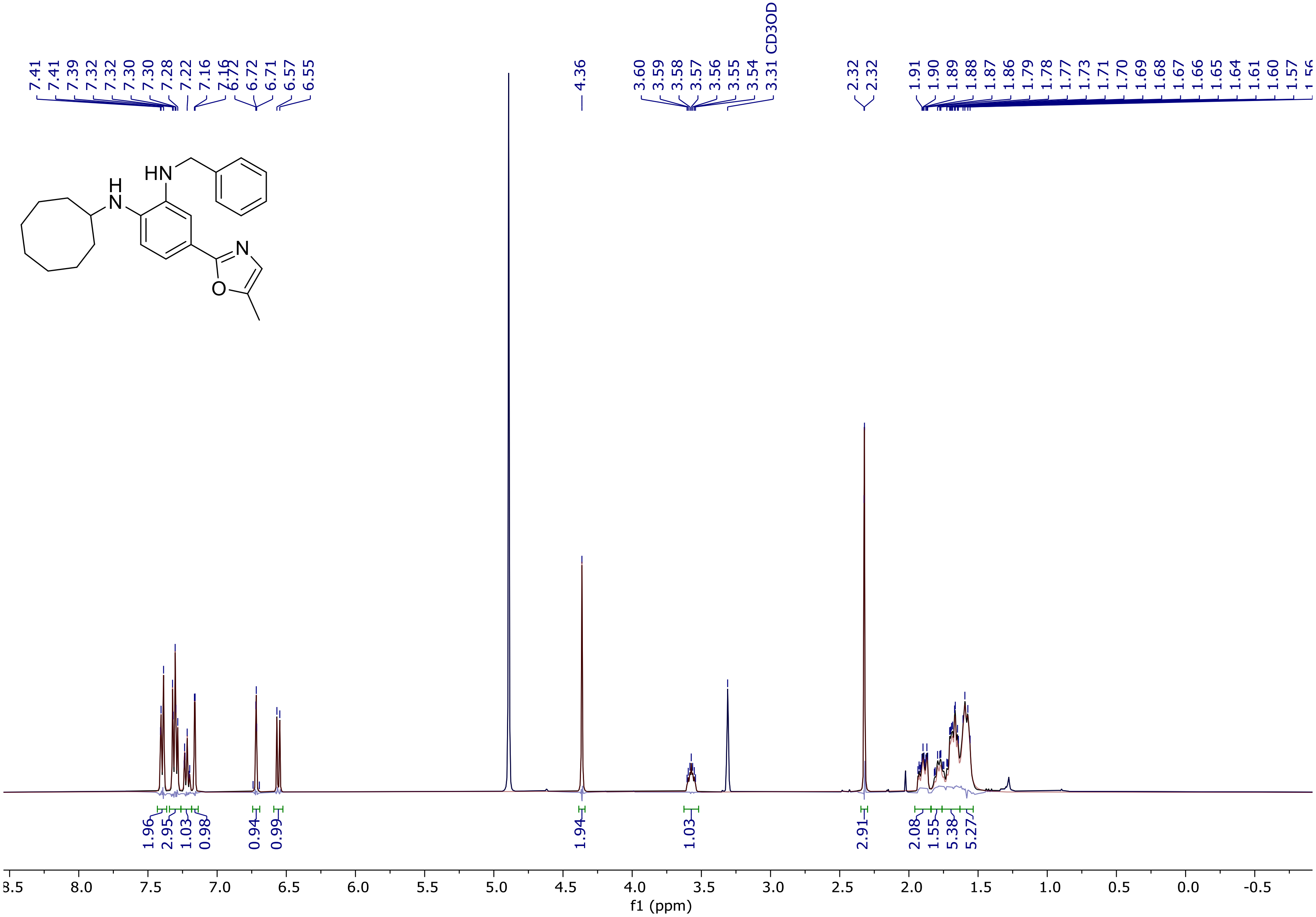

7.41
7.41
7.39
7.32
7.32
7.30
7.30
7.28
7.22
7.16
7.16
6.72
6.72
6.71
6.57
6.55
4.36
3.60
3.59
3.58
3.57
3.56
3.55
3.54
3.31
CD3OD
2.32
2.32
1.91
1.90
1.89
1.88
1.87
1.86
1.79
1.78
1.77
1.73
1.71
1.70
1.69
1.68
1.67
1.66
1.65
1.64
1.61
1.60
1.57
HN
H
N
N
O
1.96
2.95
1.03
0.98
0.94
0.99
1.94
1.03
2.91
2.08
1.55
5.38
5.27
8.5
8.0
7.5
7.0
6.5
6.0
5.5
5.0
4.5
4.0
3.5
3.0
2.5
2.0
1.5
1.0
0.5
0.0
-0.5
f1 (ppm)

Compound 82

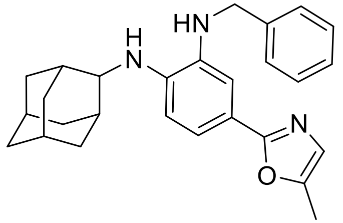

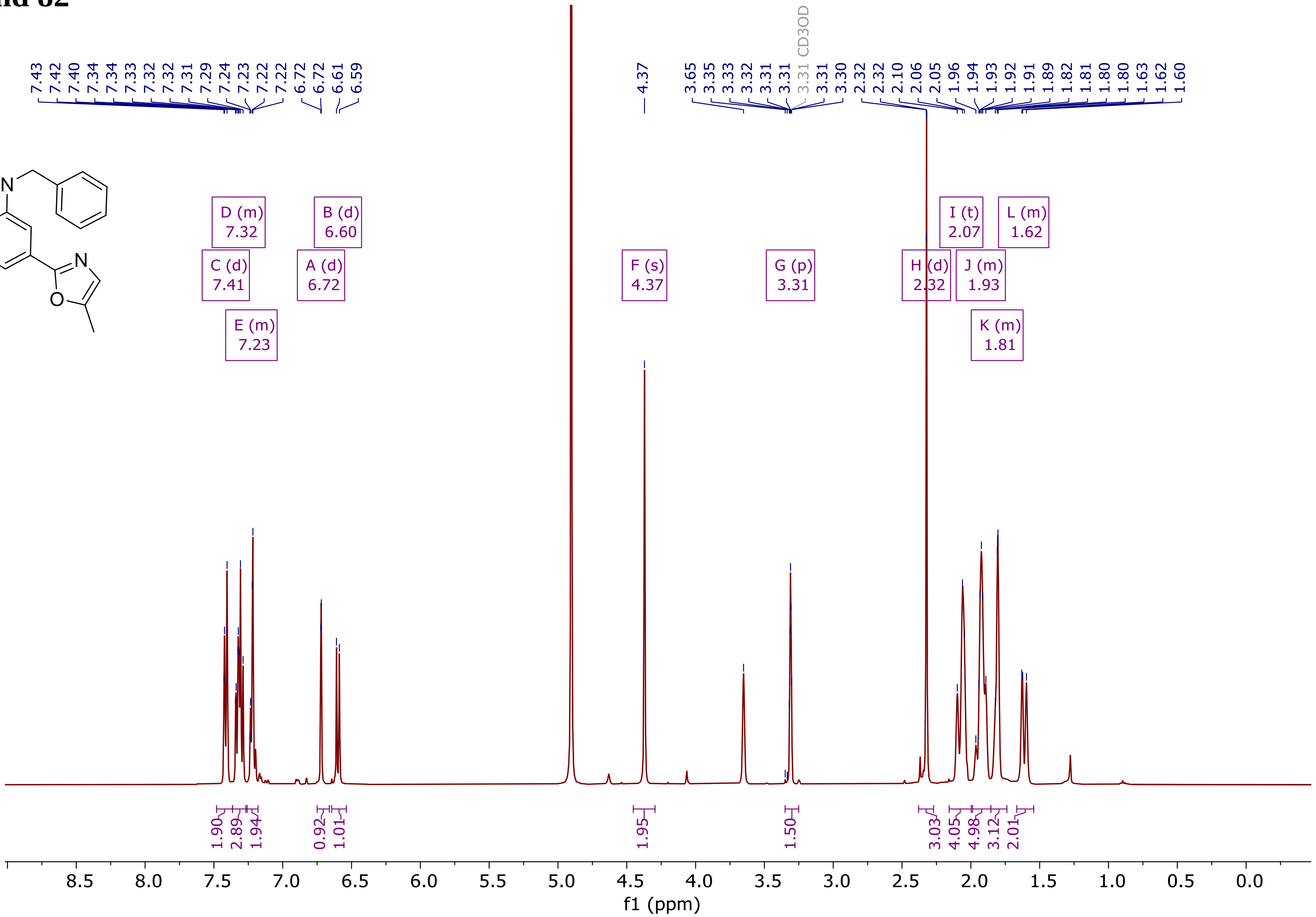

CD3OD
D (m) 7.32
B (d) 6.60
C (d) 7.41
A (d) 6.72
E (m) 7.23
F (s) 4.37
G (p) 3.31
H (d) 2.32
I (t) 2.07
J (m) 1.93
K (m) 1.81
L (m) 1.62
f1 (ppm)

Compound 83

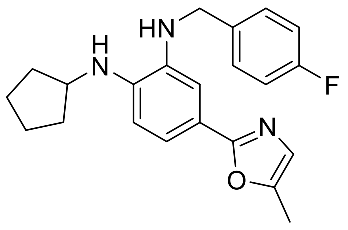

HN
H
N
F
N
O

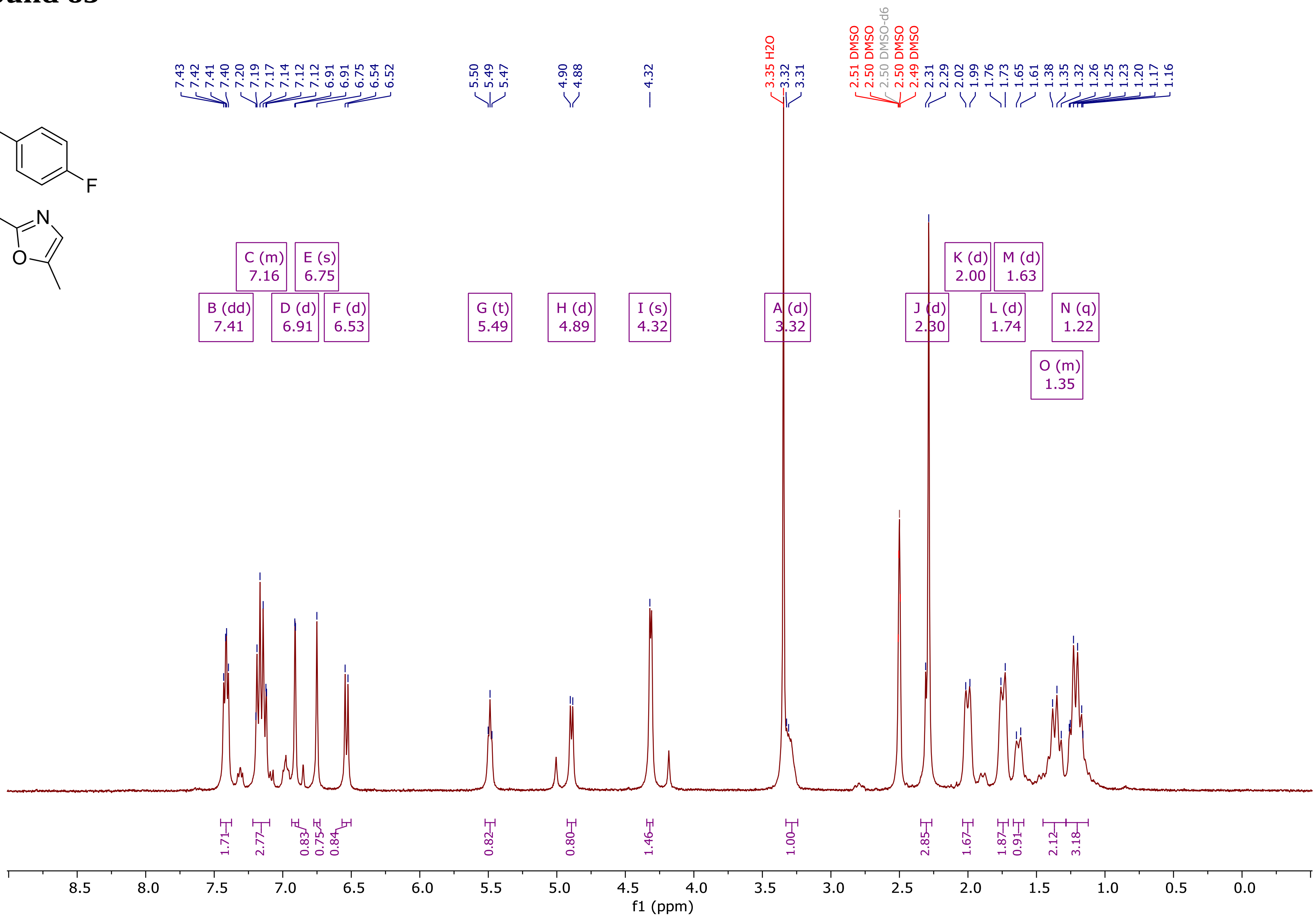

7.43 7.42 7.41 7.40 7.20 7.19 7.17 7.14 7.12 7.12 6.91 6.91 6.75 6.54 6.52
5.50 5.49 5.47
4.90 4.88
4.32
3.35 H2O 3.32 3.31
2.51 DMSO 2.50 DMSO 2.50 DMSO-d6 2.50 DMSO 2.49 DMSO
2.31 2.29 2.02 1.99 1.76 1.73 1.65 1.61 1.38 1.35 1.32 1.26 1.25 1.23 1.20 1.17 1.16
C (m) 7.16
E (s) 6.75
B (dd) 7.41
D (d) 6.91
F (d) 6.53
G (t) 5.49
H (d) 4.89
I (s) 4.32
A (d) 3.32
J (d) 2.30
K (d) 2.00
M (d) 1.63
L (d) 1.74
N (q) 1.22
O (m) 1.35
1.71 2.77 0.83 0.75 0.84 0.82 0.80 1.46 1.00 2.85 1.67 1.87 0.91 2.12 3.18
8.5 8.0 7.5 7.0 6.5 6.0 5.5 5.0 4.5 4.0 3.5 3.0 2.5 2.0 1.5 1.0 0.5 0.0
f1 (ppm)

Compound 84

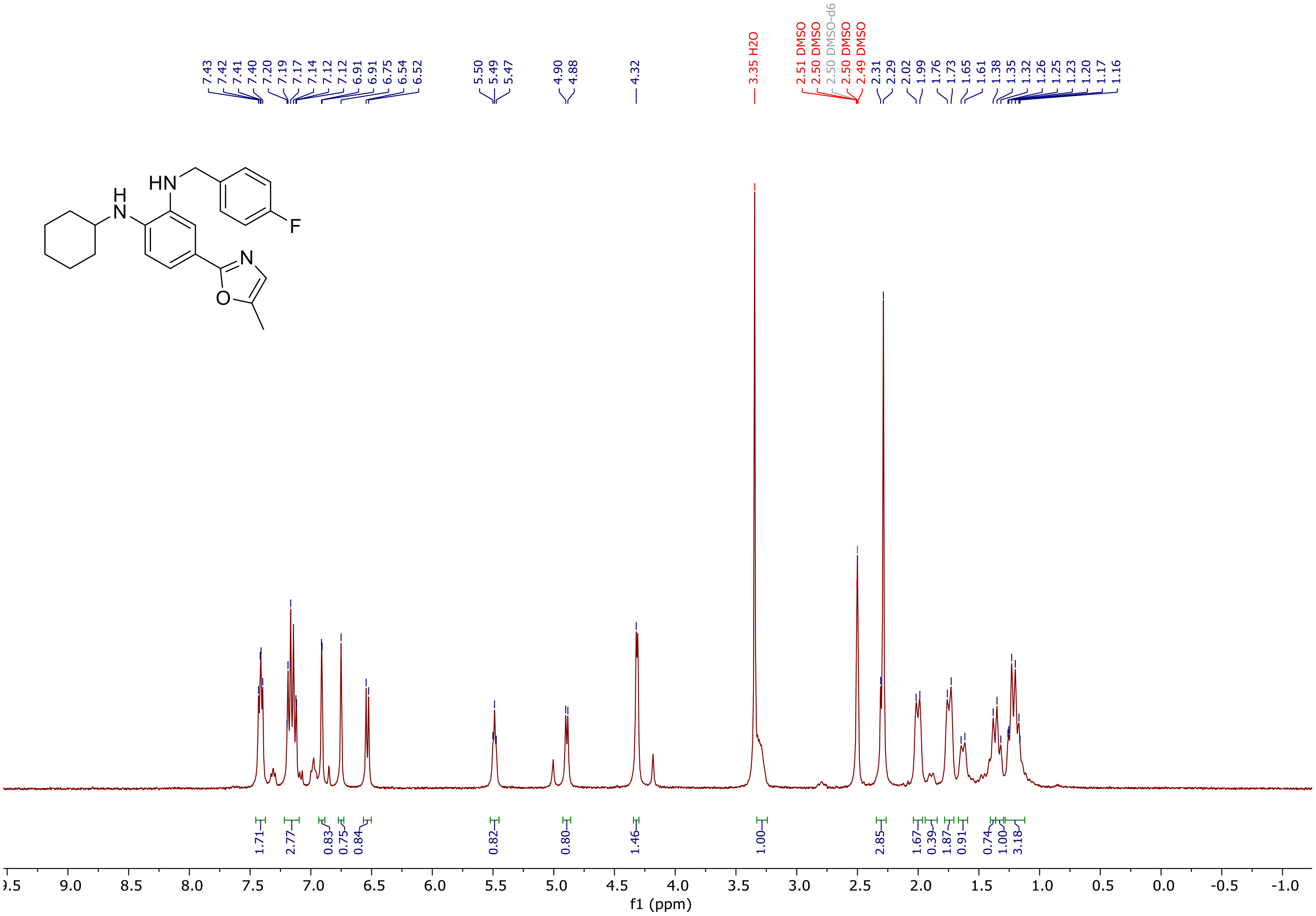

7.43
7.42
7.41
7.40
7.20
7.19
7.17
7.14
7.12
7.12
6.91
6.91
6.75
6.54
6.52
5.50
5.49
5.47
4.90
4.88
4.32
3.35 H2O
2.51 DMSO
2.50 DMSO
2.50 DMSO-d6
2.50 DMSO
2.49 DMSO
2.31
2.29
2.02
1.99
1.76
1.73
1.65
1.61
1.38
1.35
1.32
1.26
1.25
1.23
1.20
1.17
1.16
HN
H
N
F
N
O
1.71
2.77
0.83
0.75
0.84
0.82
0.80
1.46
1.00
2.85
1.67
0.39
1.87
0.91
0.74
1.00
3.18
f1 (ppm)

Compound 85

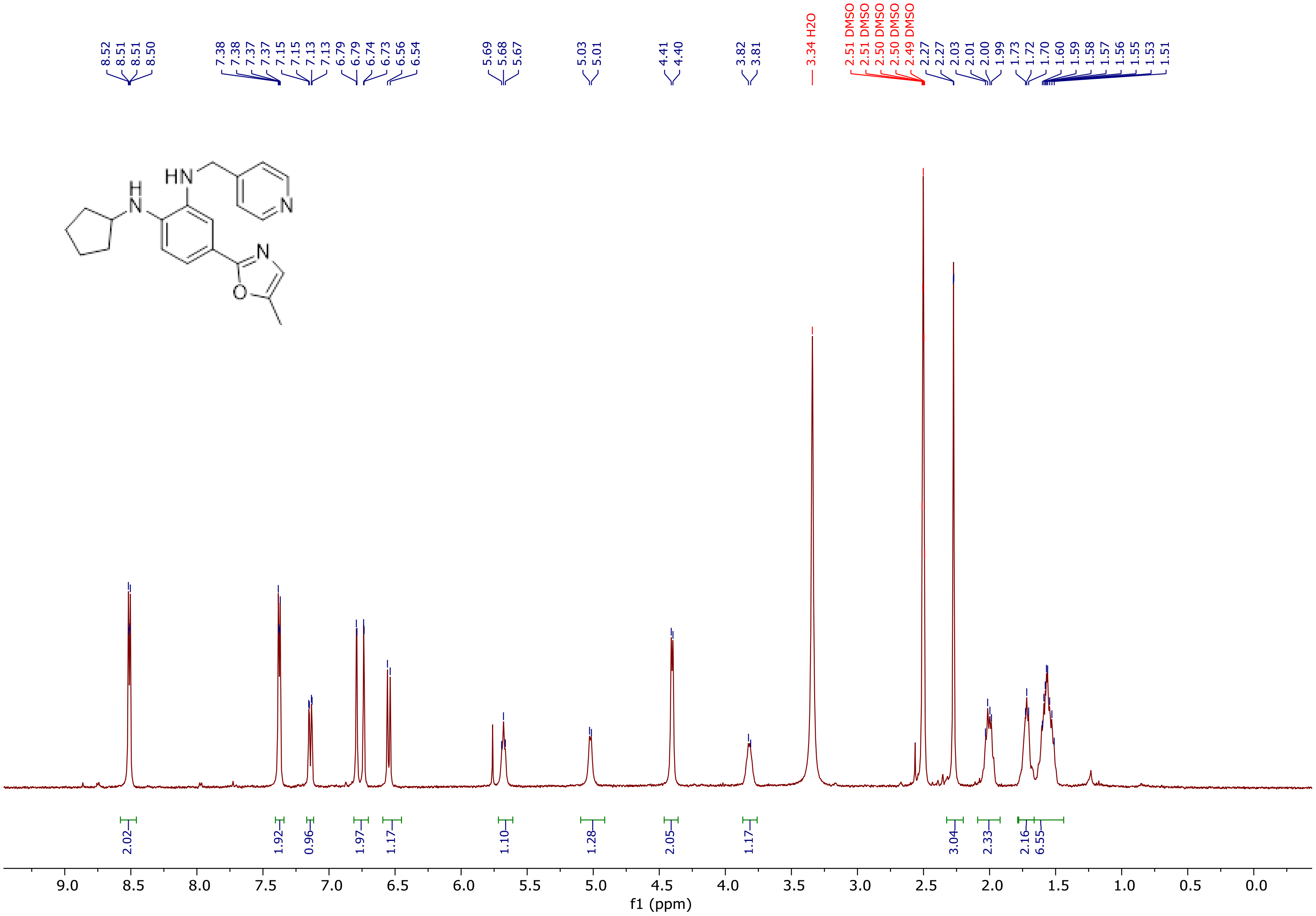

Compound 86

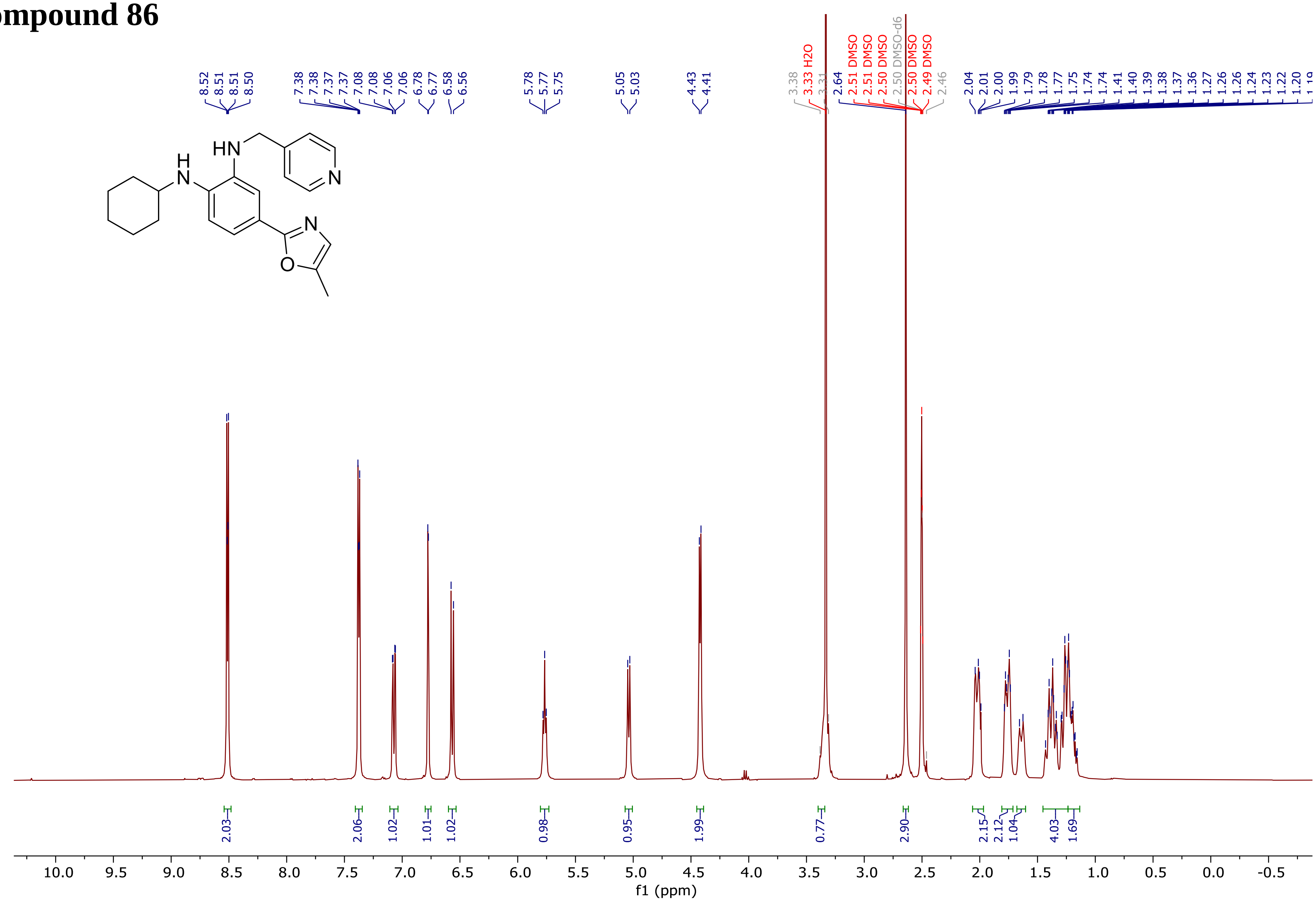

HN
H
N
N
N
O
8.52 8.51 8.51 8.50
7.38 7.38 7.37 7.37 7.08 7.08 7.06 7.06 6.78 6.77 6.58 6.56
5.78 5.77 5.75
5.05 5.03
4.43 4.41
3.38 3.33 H2O 3.31
2.64 2.51 DMSO 2.51 DMSO 2.50 DMSO 2.50 DMSO-d6 2.50 DMSO 2.49 DMSO 2.46
2.04 2.01 2.00 1.99 1.79 1.78 1.77 1.75 1.74 1.74 1.41 1.40 1.39 1.38 1.37 1.36 1.27 1.26 1.26 1.24 1.23 1.22 1.20
2.03 2.06 1.02 1.01 1.02 0.98 0.95 1.99 0.77 2.90 2.15 2.12 1.04 4.03 1.69
10.0 9.5 9.0 8.5 8.0 7.5 7.0 6.5 6.0 5.5 5.0 4.5 4.0 3.5 3.0 2.5 2.0 1.5 1.0 0.5 0.0 -0.5
f1 (ppm)

Compound 94 (UAMC-4749)

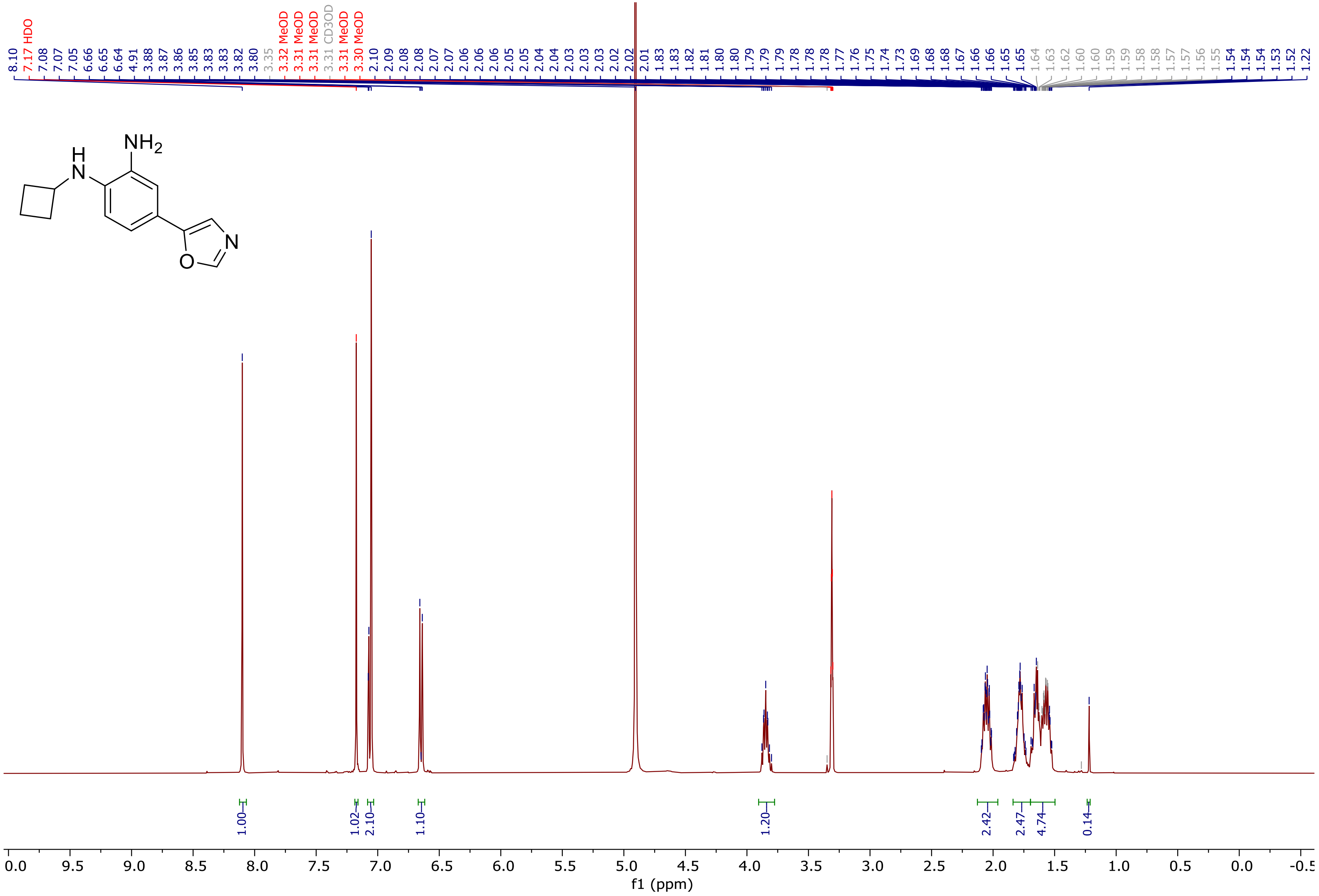

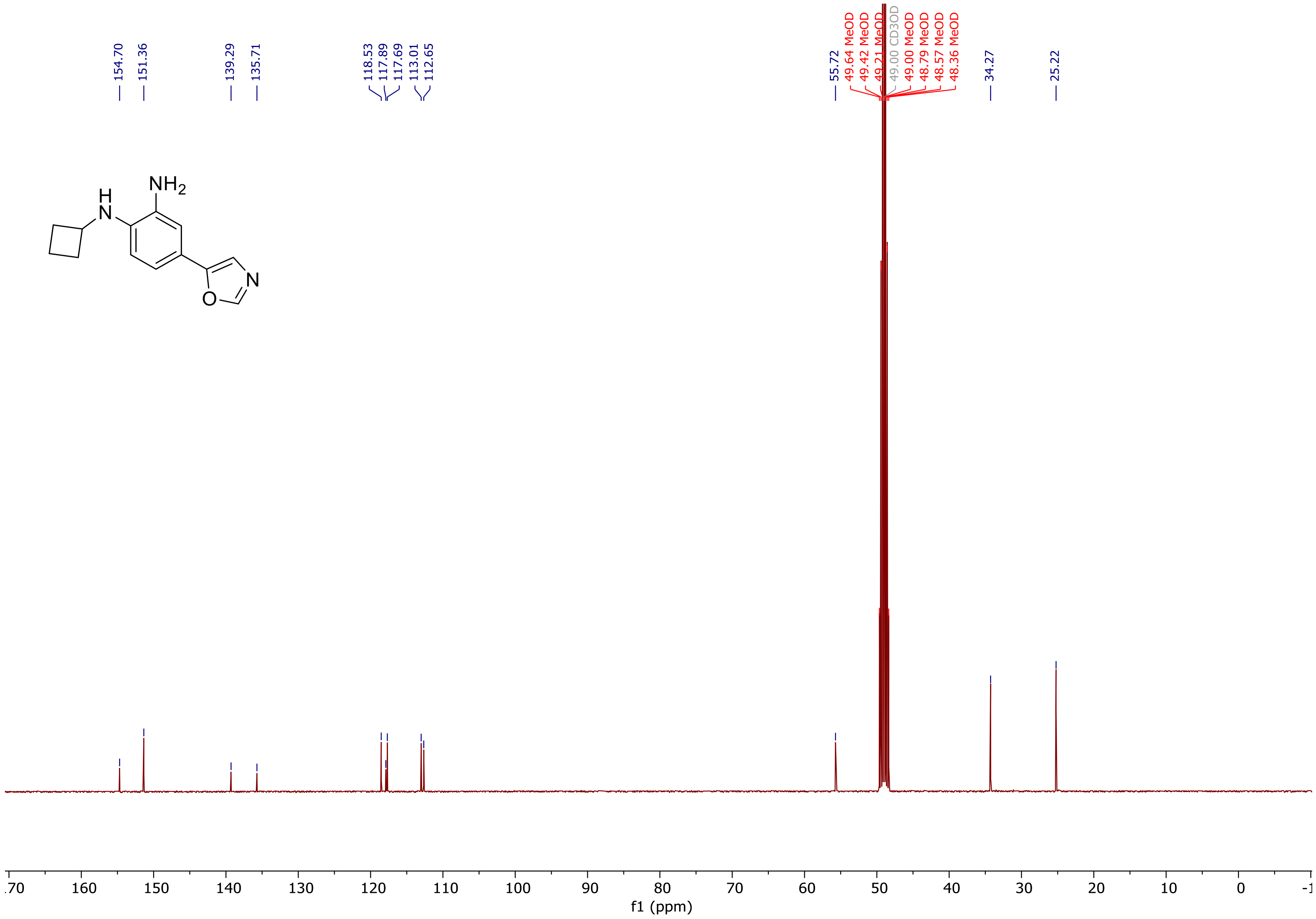

154.70
151.36
139.29
135.71
118.53
117.89
117.69
113.01
112.65
55.72
49.64 MeOD
49.42 MeOD
49.21 MeOD
49.00 CD3OD
49.00 MeOD
48.79 MeOD
48.57 MeOD
48.36 MeOD
34.27
25.22
NH2
H
N
N
O
f1 (ppm)

Compound 95

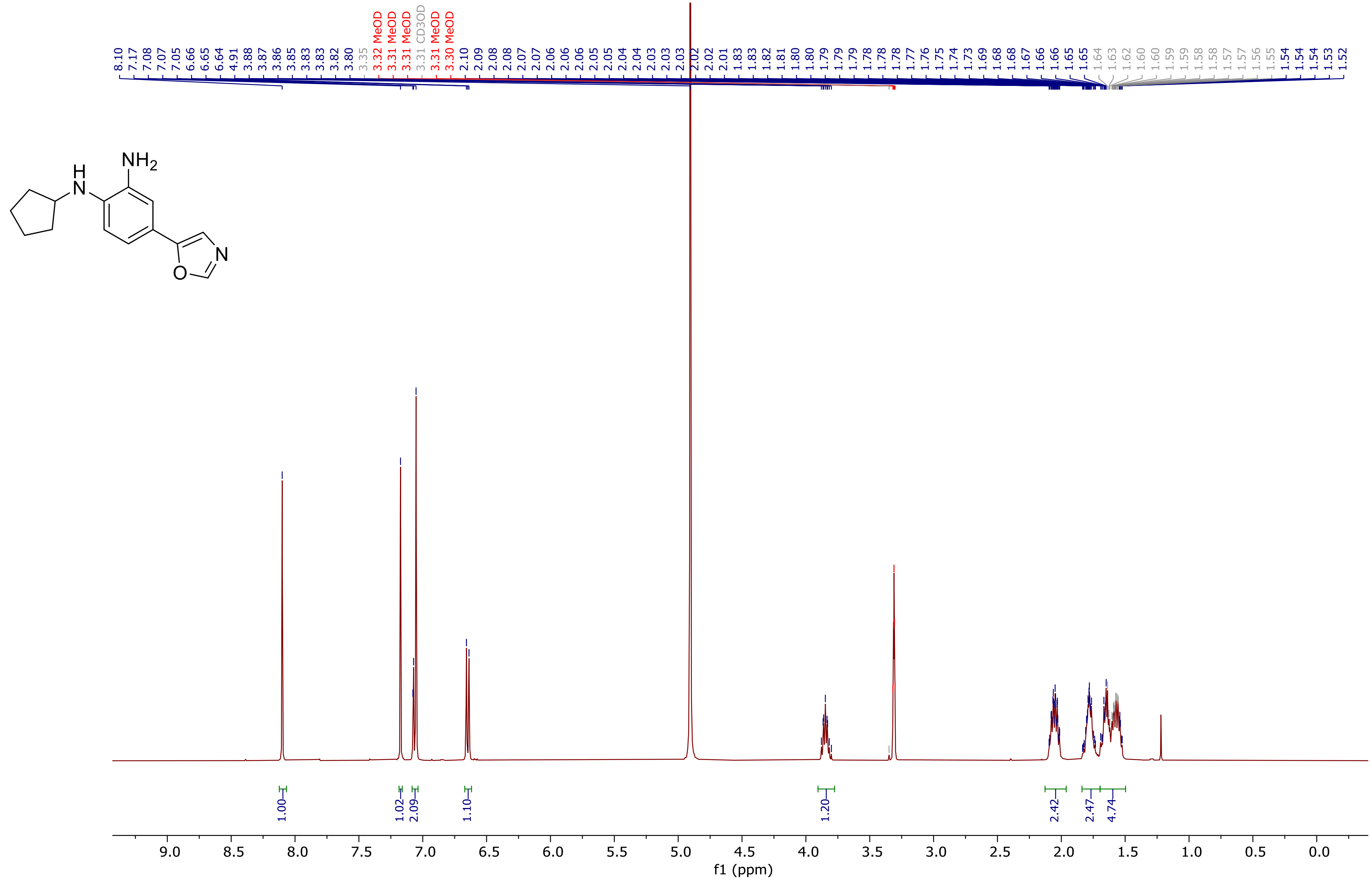

NH2
H
N
N
O
8.10 7.17 7.08 7.07 7.05 6.66 6.65 6.64 4.91 3.88 3.87 3.86 3.85 3.83 3.83 3.82 3.80 3.35
3.32 MeOD 3.31 MeOD 3.31 MeOD 3.31 CD3OD 3.31 MeOD 3.30 MeOD
2.10 2.09 2.08 2.08 2.07 2.07 2.06 2.06 2.06 2.05 2.05 2.04 2.04 2.03 2.03 2.03 2.02 2.02 2.01 1.83 1.83 1.82 1.81 1.80 1.80 1.79 1.79 1.79 1.78 1.78 1.77 1.76 1.75 1.74 1.73 1.69 1.68 1.68 1.67 1.66 1.66 1.65 1.65 1.64 1.63 1.62 1.60 1.60 1.59 1.59 1.58 1.58 1.57 1.57 1.56 1.55 1.54 1.54 1.54 1.53 1.52
1.00 1.02 2.09 1.10 1.20 2.42 2.47 4.74
9.0 8.5 8.0 7.5 7.0 6.5 6.0 5.5 5.0 4.5 4.0 3.5 3.0 2.5 2.0 1.5 1.0 0.5 0.0
f1 (ppm)

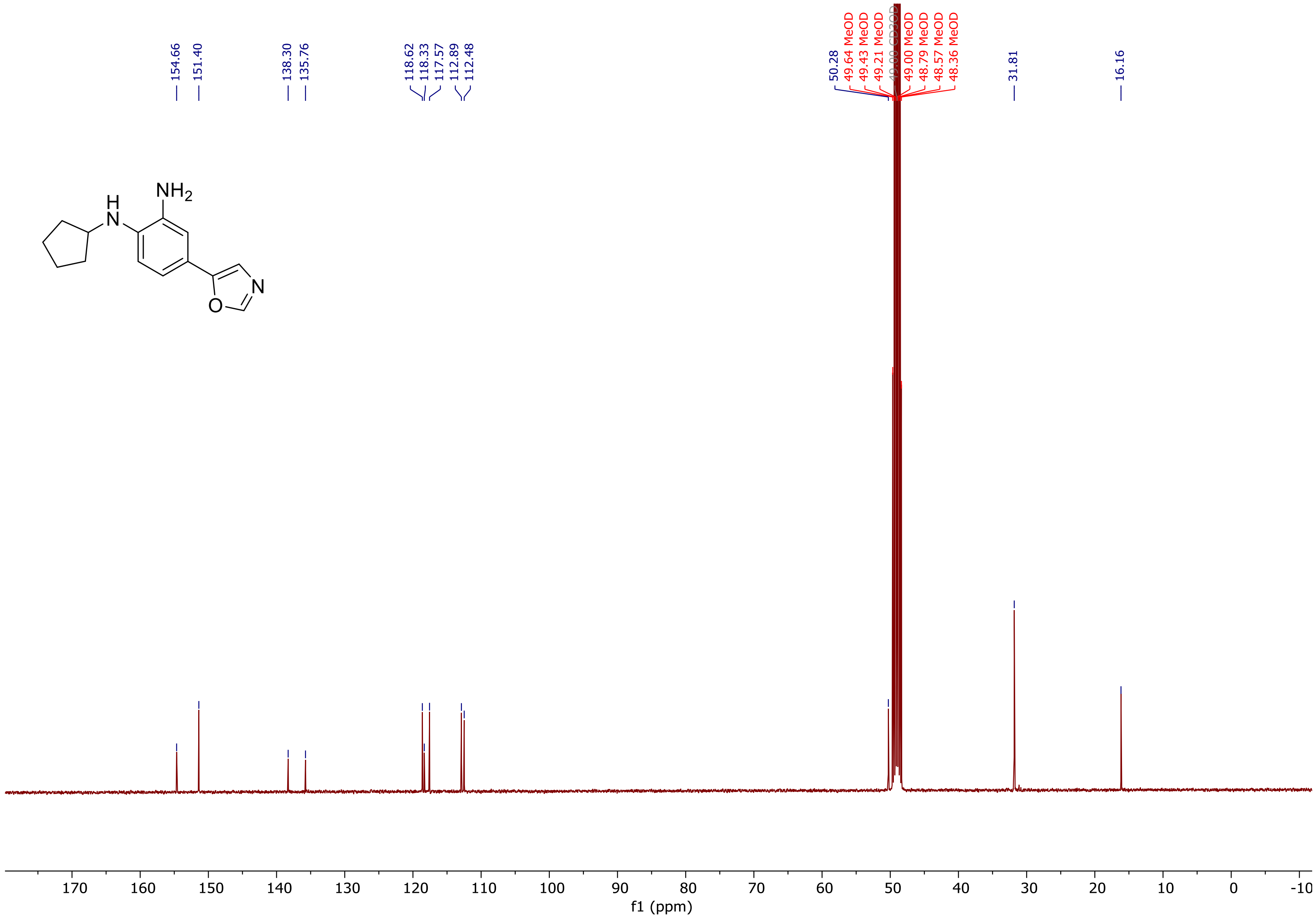

154.66
151.40
138.30
135.76
118.62
118.33
117.57
112.89
112.48
50.28
49.64 MeOD
49.43 MeOD
49.21 MeOD
49.00 MeOD
48.79 MeOD
48.57 MeOD
48.36 MeOD
31.81
16.16
NH2
H
N
N
O
f1 (ppm)

Compound 96 (UAMC-4821)

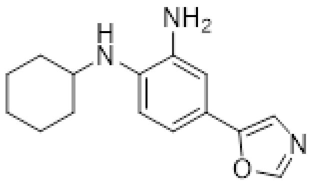

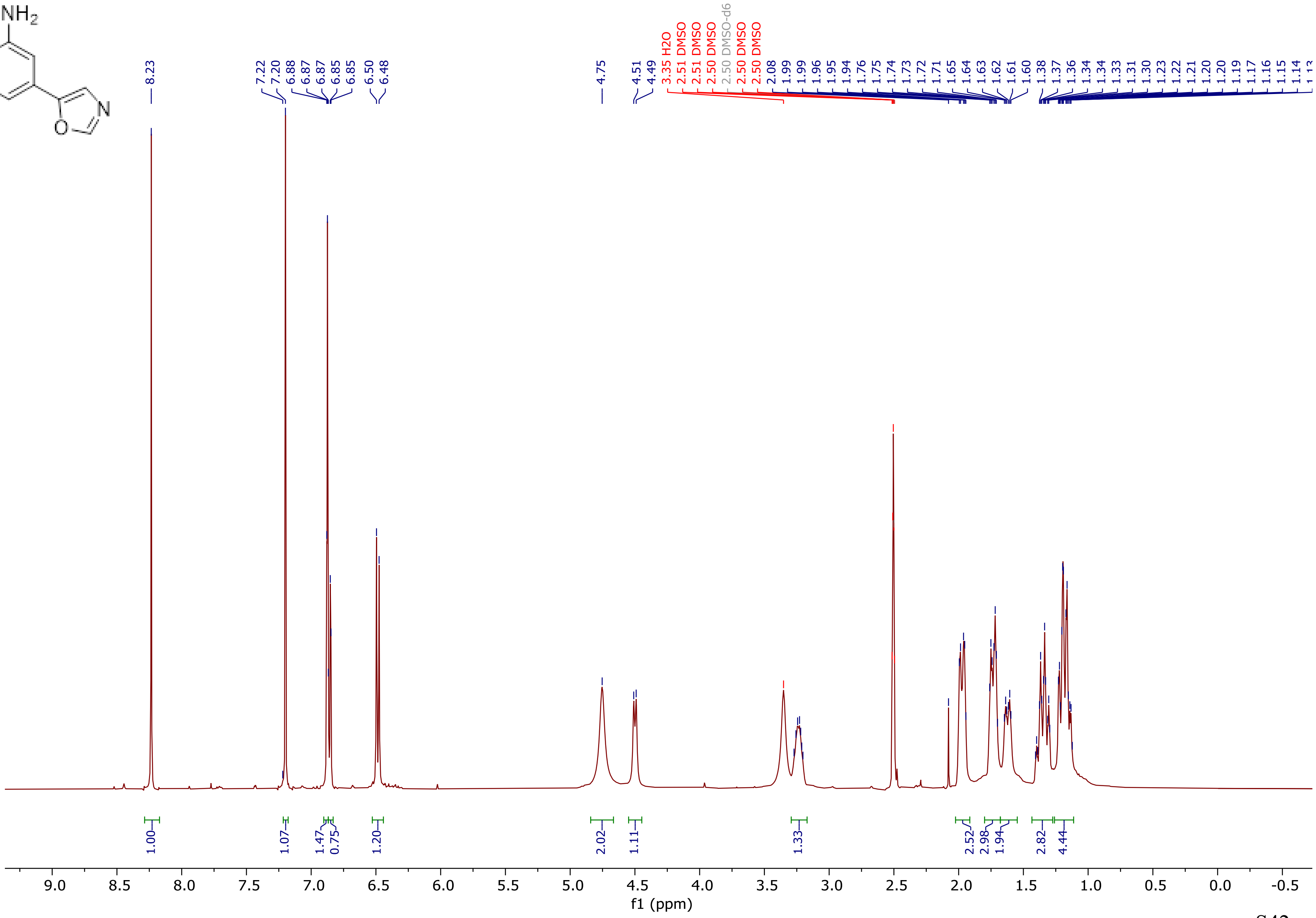

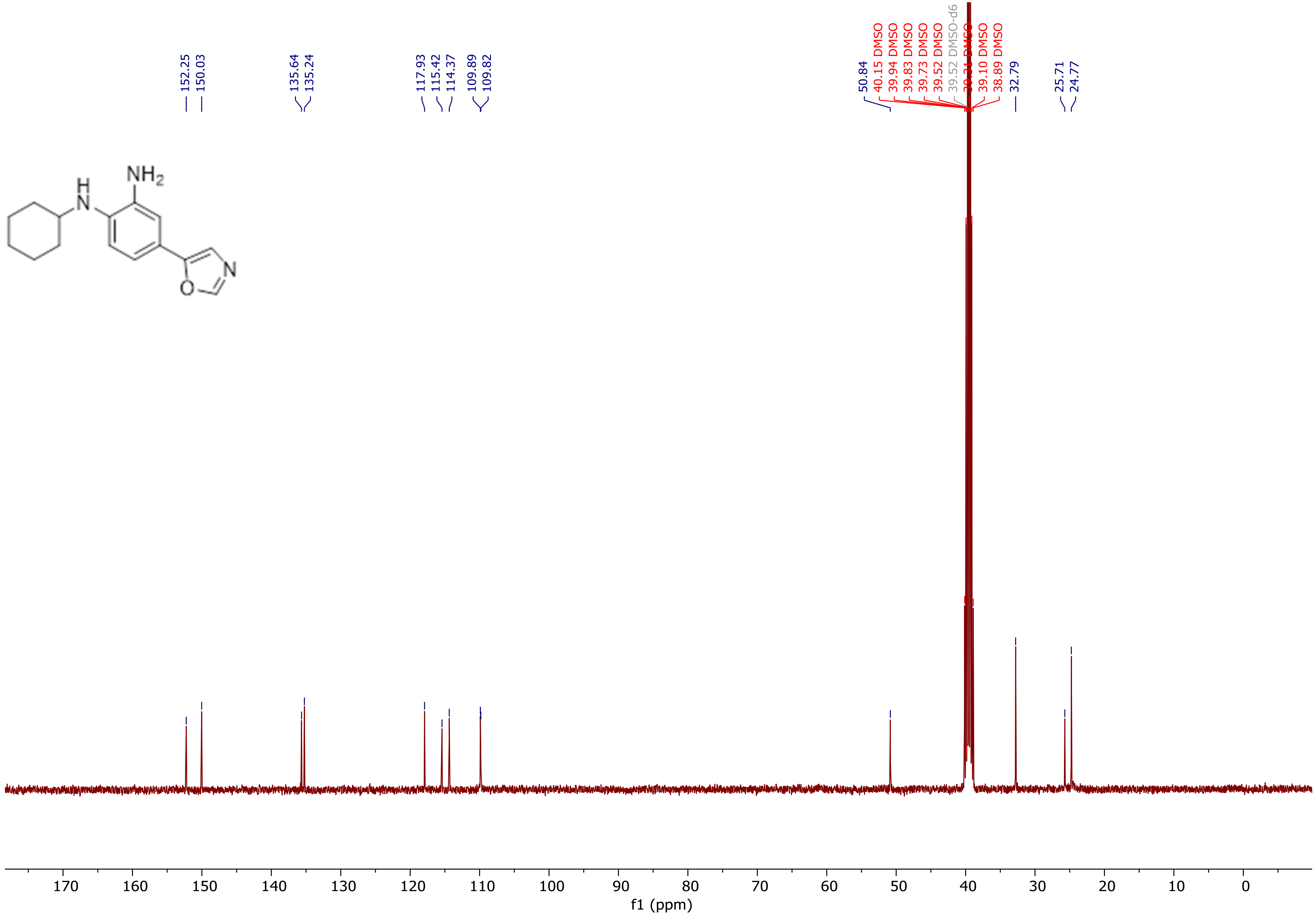
152.25
150.03
135.64
135.24
117.93
115.42
114.37
109.89
109.82
50.84
40.15 DMSO
39.94 DMSO
39.83 DMSO
39.73 DMSO
39.52 DMSO
39.52 DMSO-d6
39.10 DMSO
38.89 DMSO
32.79
25.71
24.77
NH2
H
N
N
O
170
160
150
140
130
120
110
100
90
80
70
60
50
40
30
20
10
0
f1 (ppm)

Compound 97

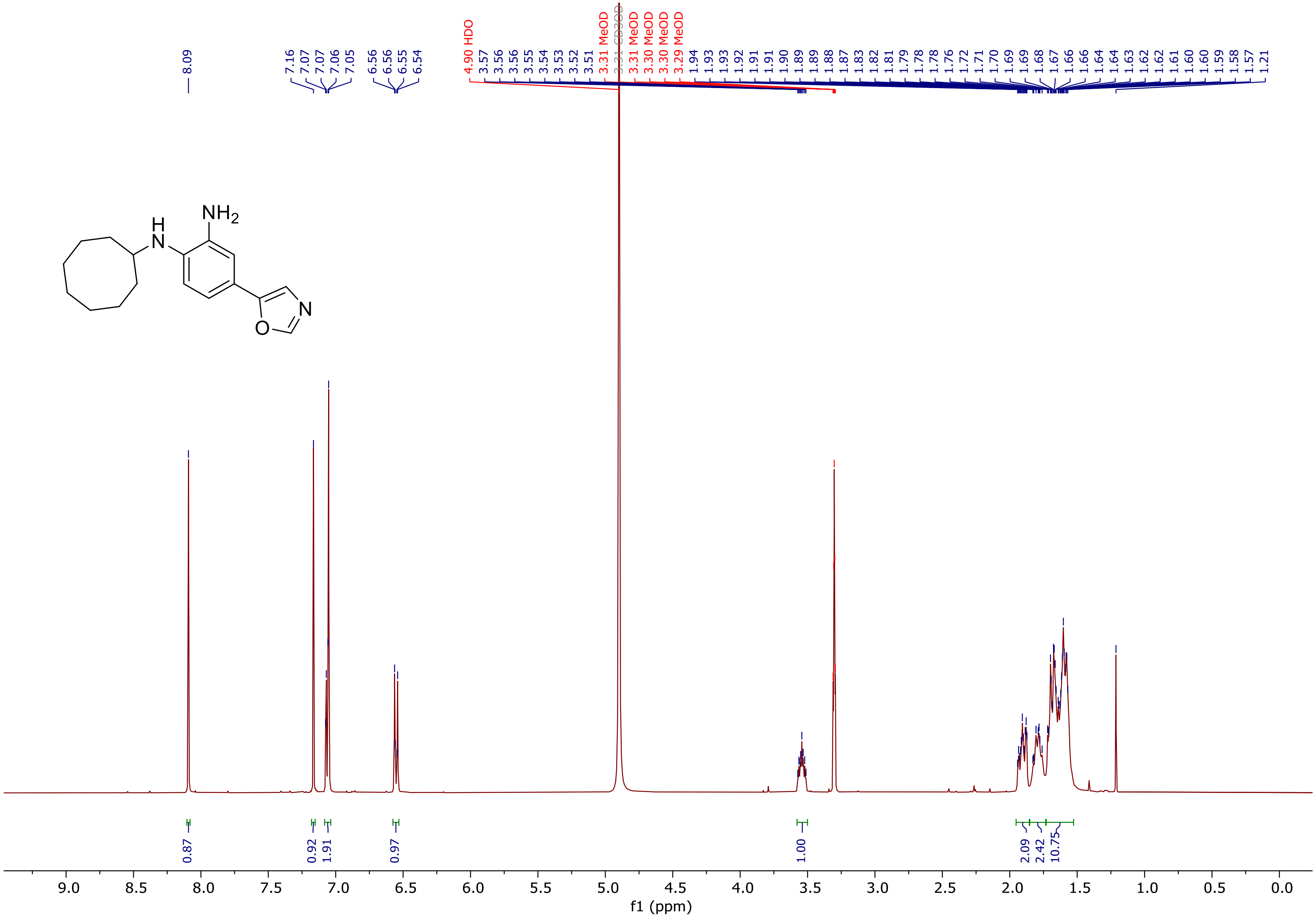

Compound 98

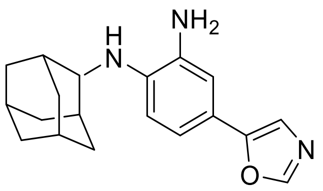

NH2
H
N
N
O

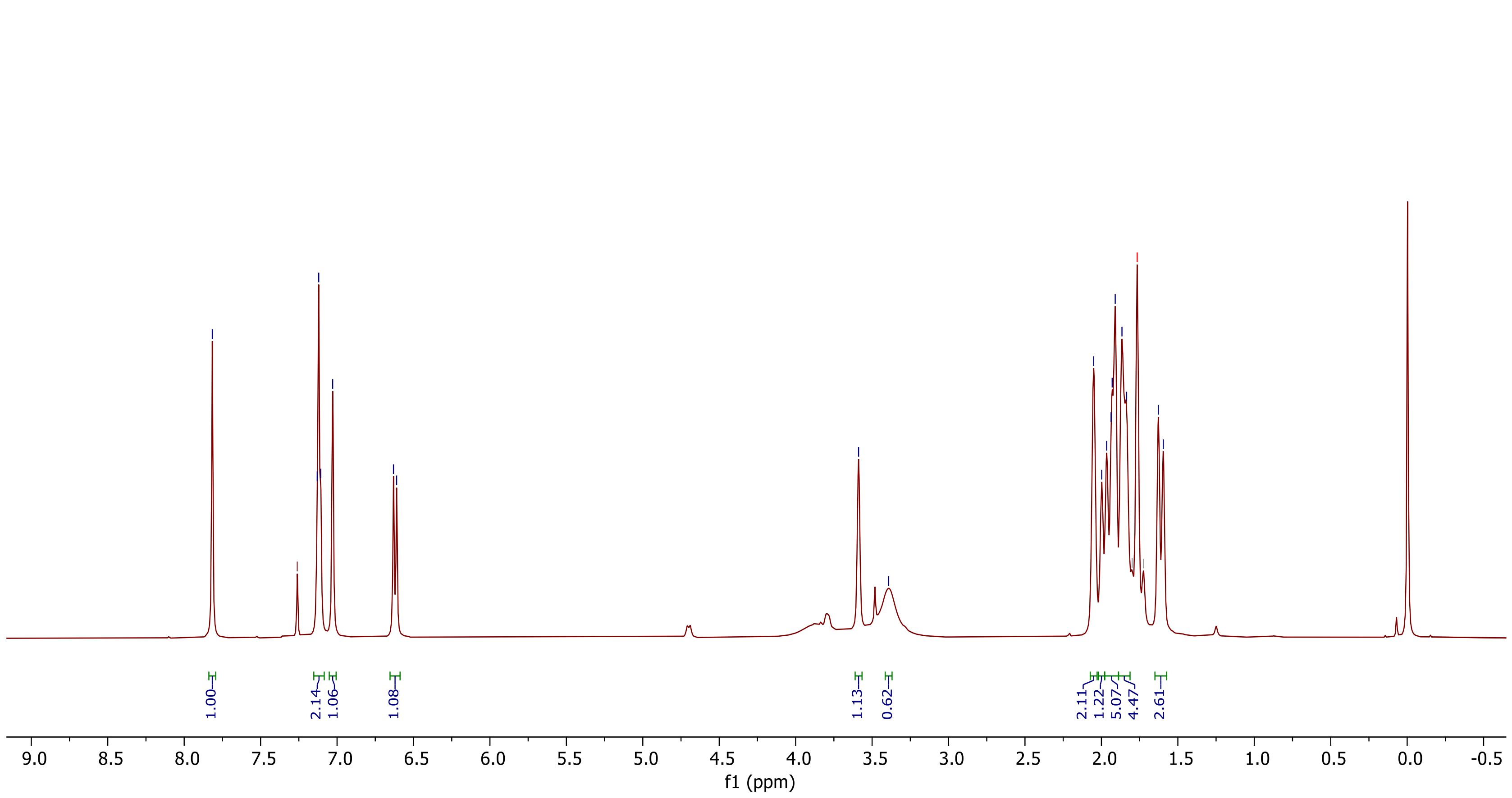

7.82
7.26 CDCl3
7.26 CDCl3
7.13
7.12
7.11
7.03
6.63
6.61
3.59
3.39
2.05
2.00
1.97
1.94
1.93
1.91
1.87
1.84
1.80
1.77 H2O
1.72
1.63
1.60
1.00
2.14
1.06
1.08
1.13
0.62
2.11
1.22
5.07
4.47
2.61
9.0
8.5
8.0
7.5
7.0
6.5
6.0
5.5
5.0
4.5
4.0
3.5
3.0
2.5
2.0
1.5
1.0
0.5
0.0
-0.5
f1 (ppm)

Compound 99

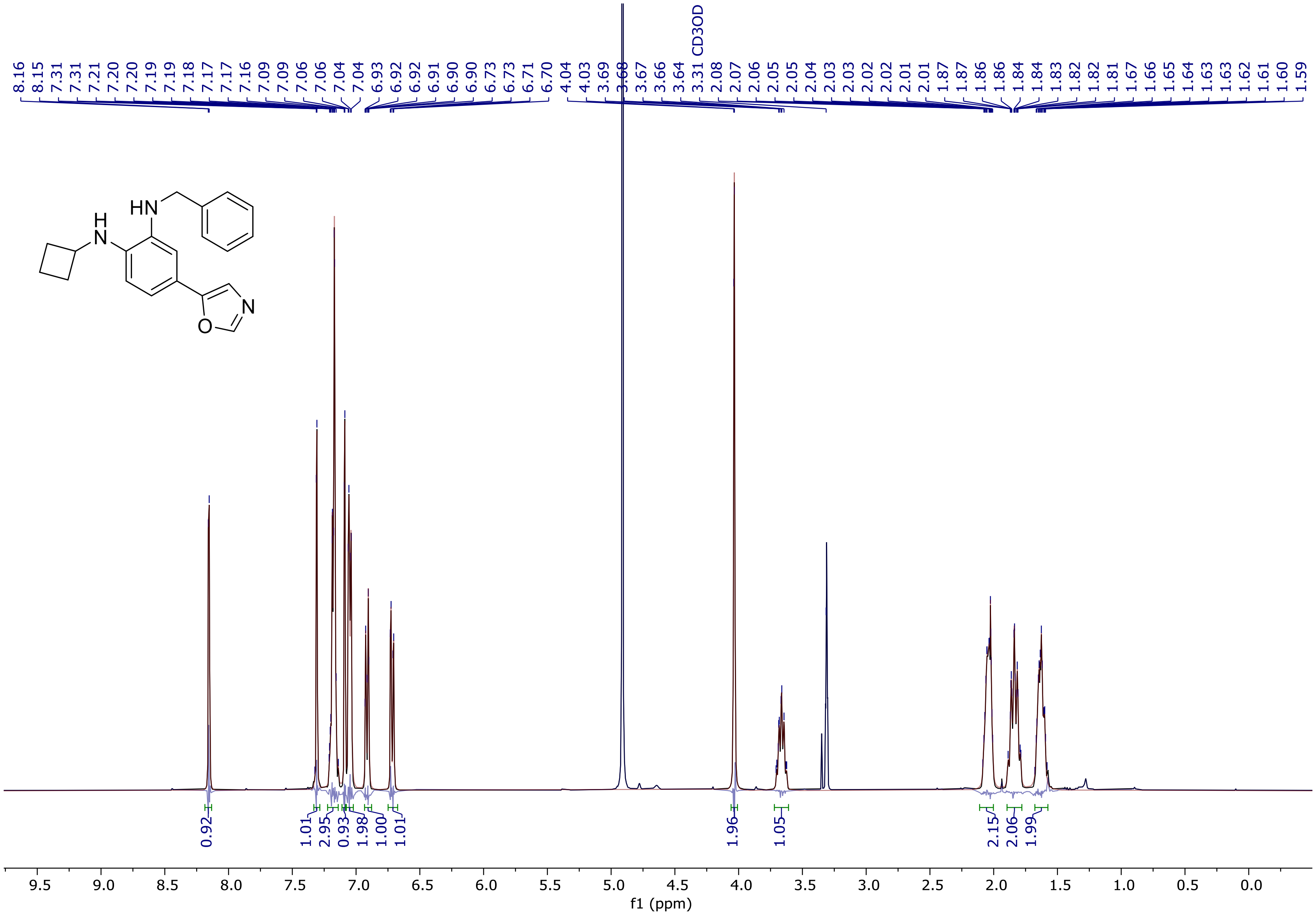

Compound 100

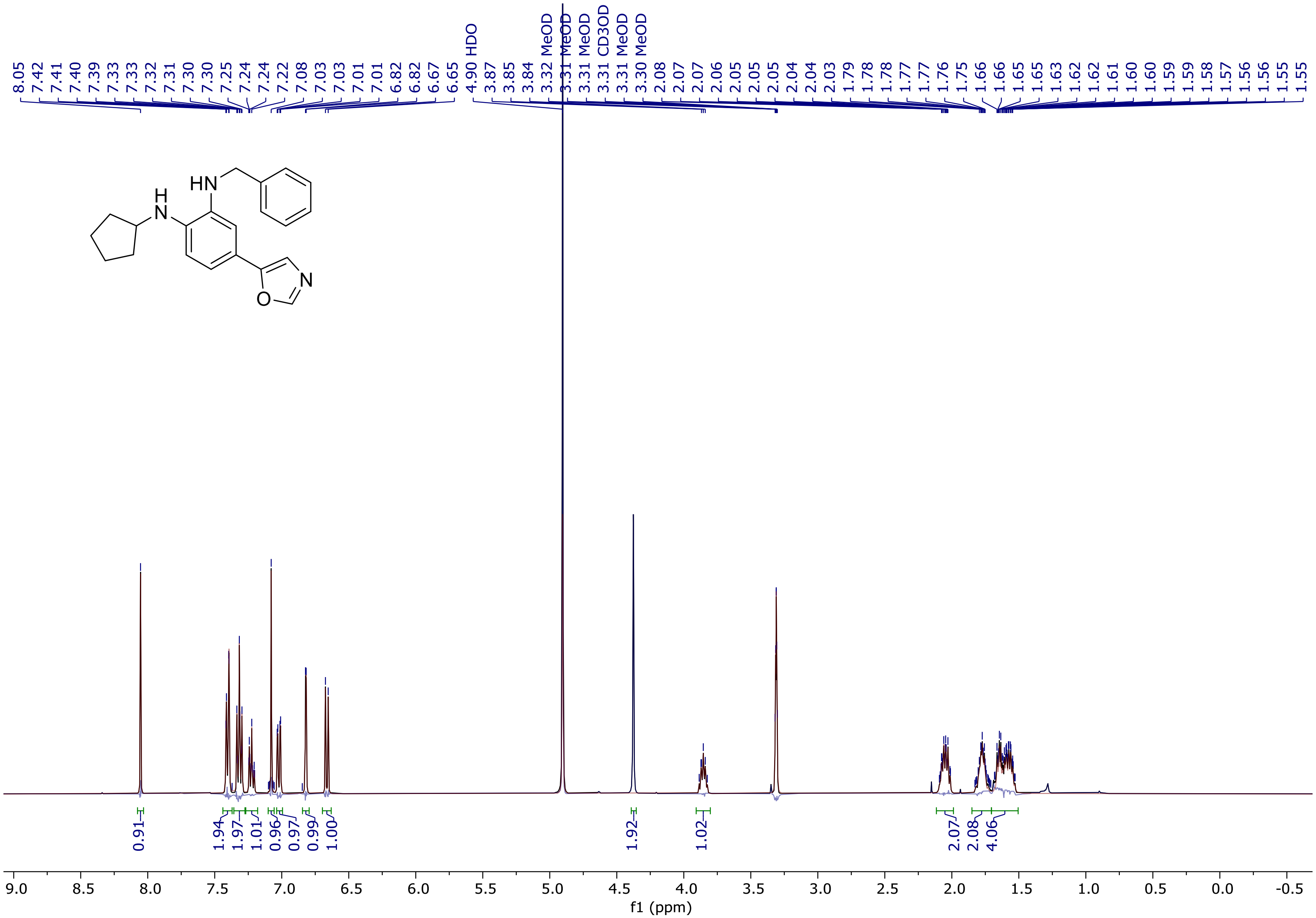

Compound 101

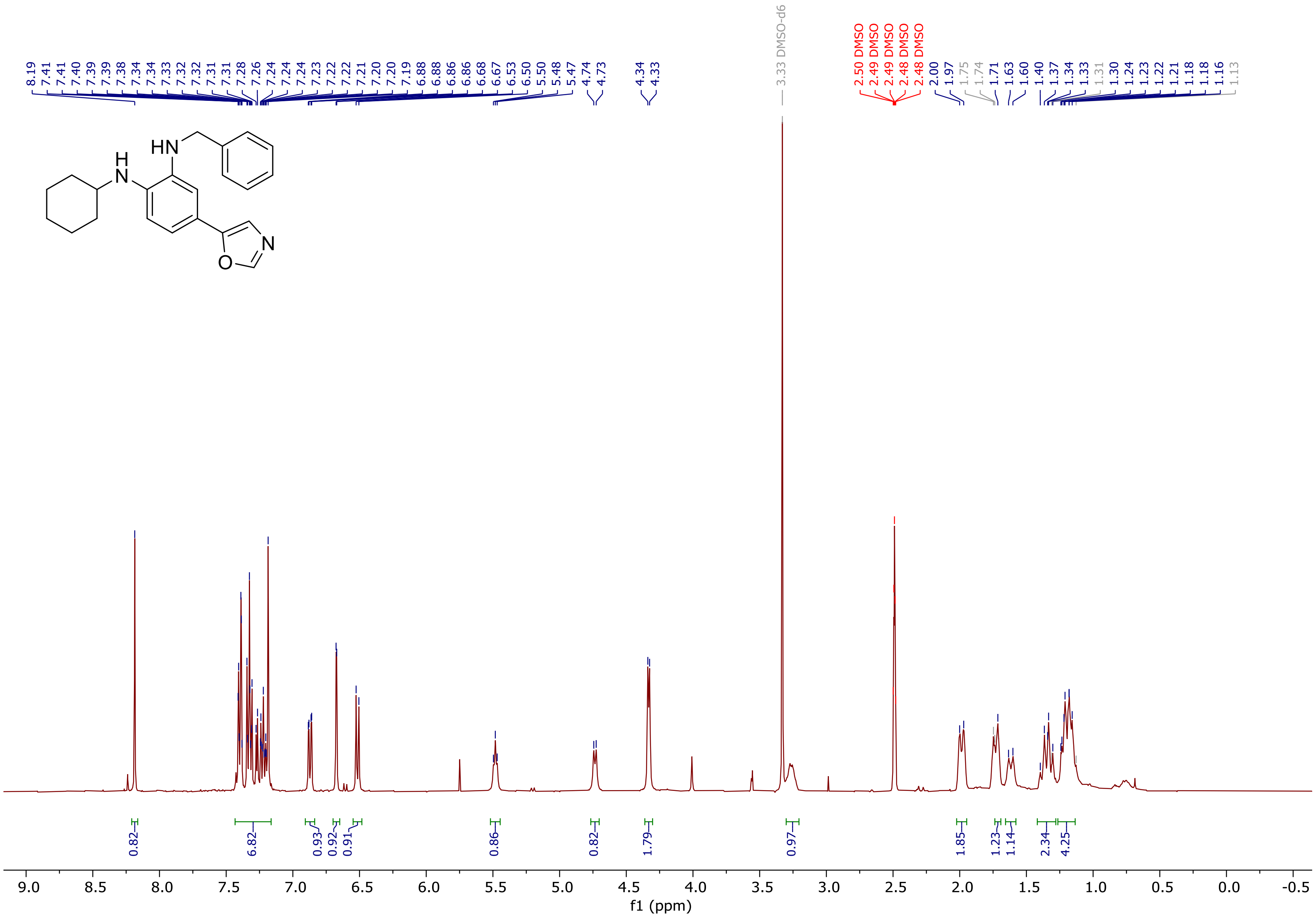

Compound 102

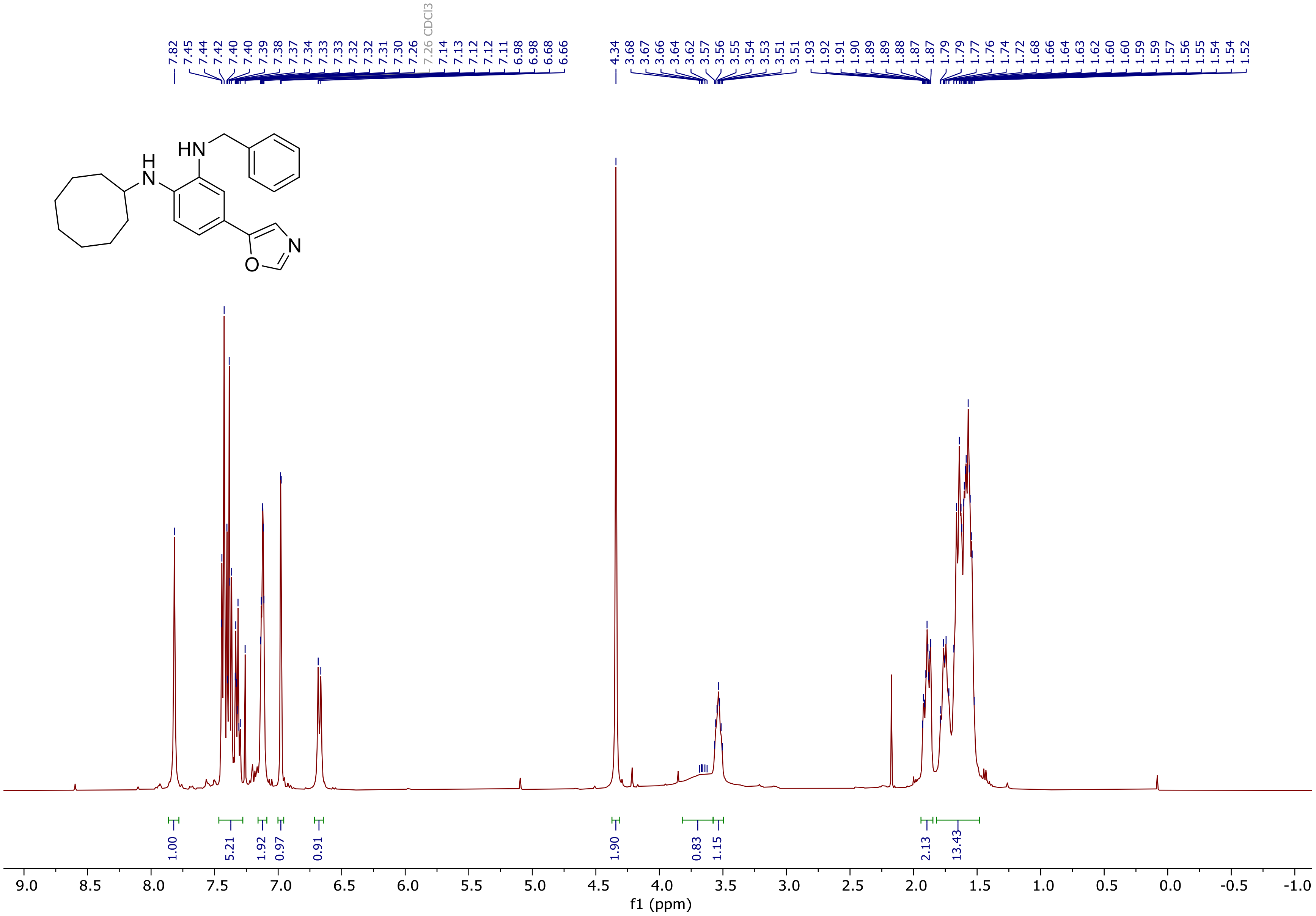

Compound 103

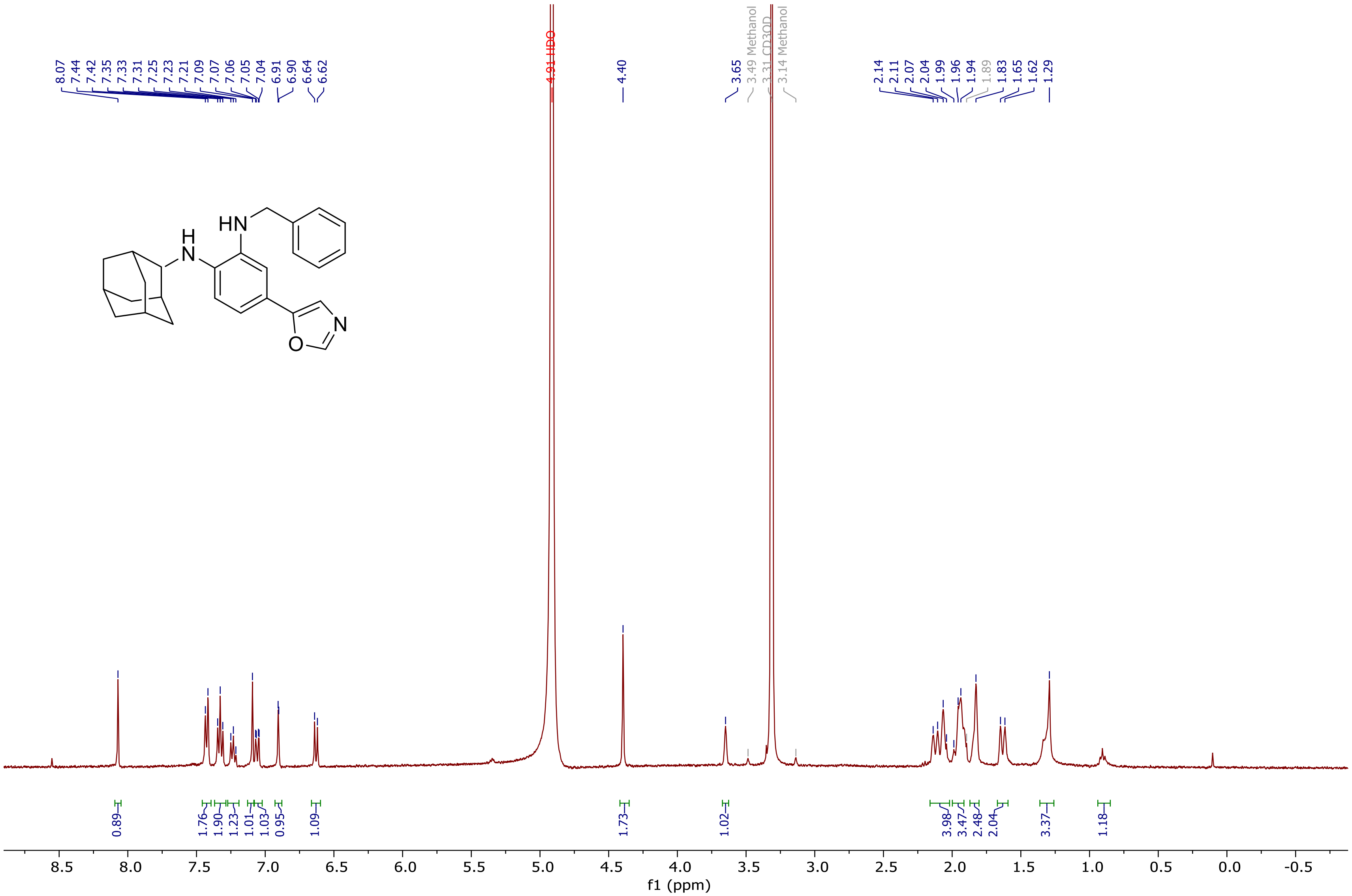

Compound 104

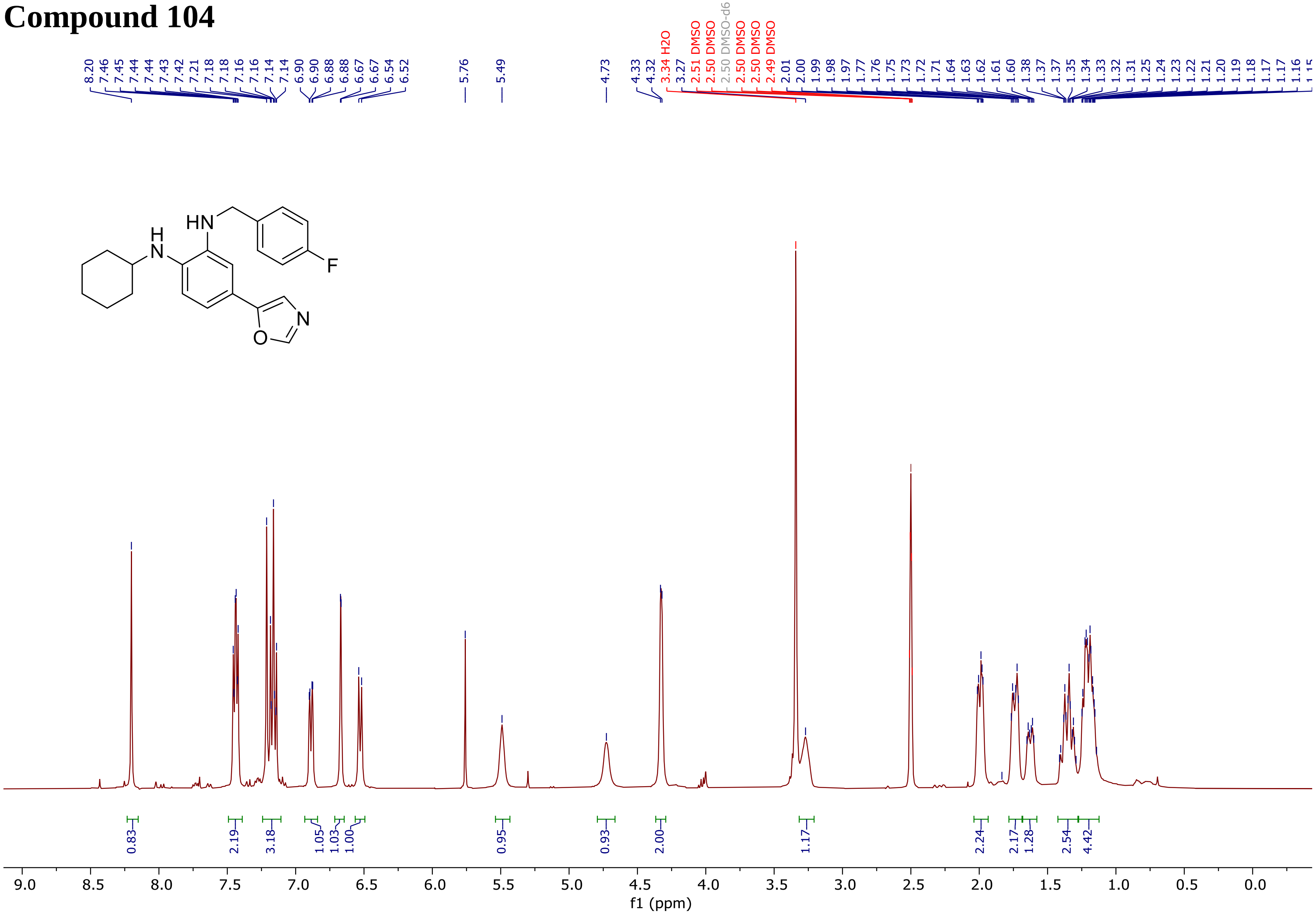

Compound 105

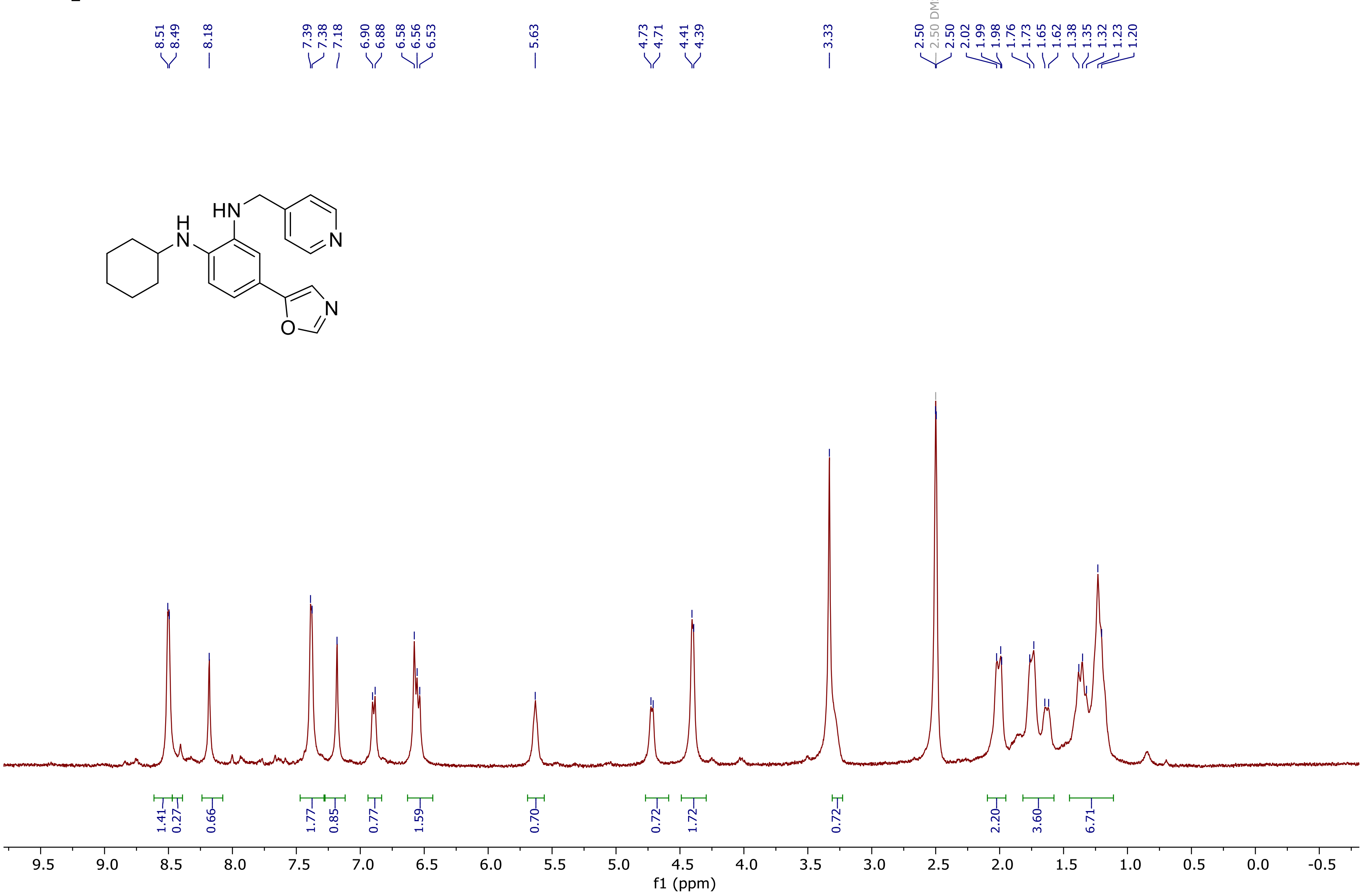

HRMS analyses of all final compounds

Compound 13

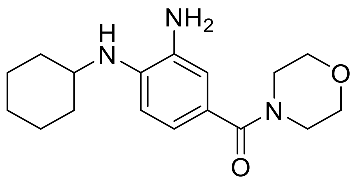

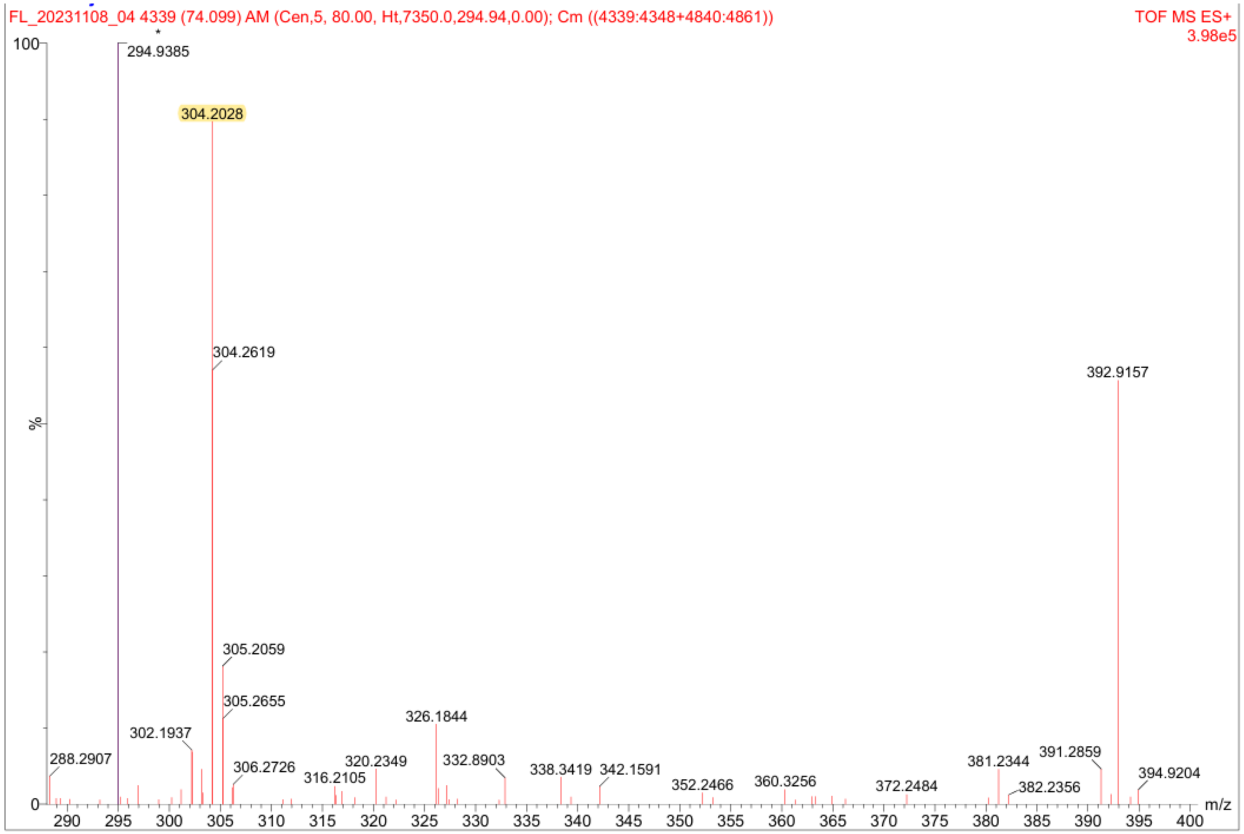

Compound 14

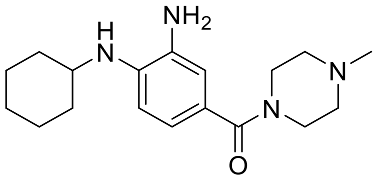

NH2
H
N
N
N
O

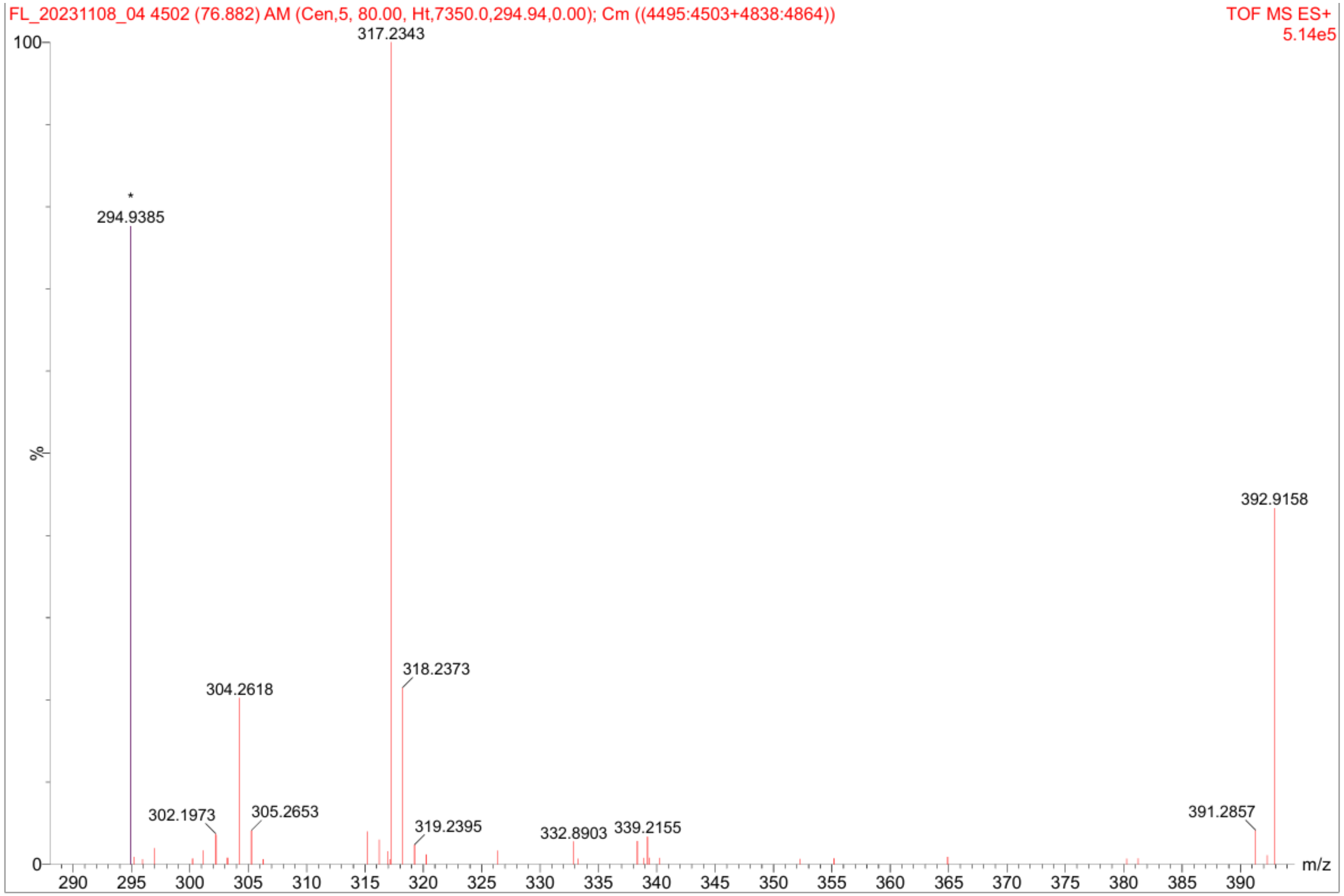

FL_20231108_04 4502 (76.882) AM (Cen,5, 80.00, Ht,7350.0,294.94,0.00); Cm ((4495:4503+4838:4864))
TOF MS ES+
5.14e5
100
%
0
317.2343
294.9385
392.9158
318.2373
304.2618
302.1973
305.2653
319.2395
332.8903
339.2155
391.2857
m/z
290
295
300
305
310
315
320
325
330
335
340
345
350
355
360
365
370
375
380
385
390

Compound 15

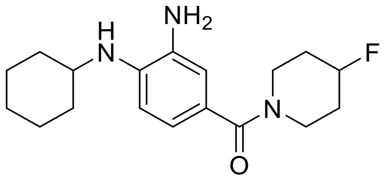

H
N
NH2
F
N
O

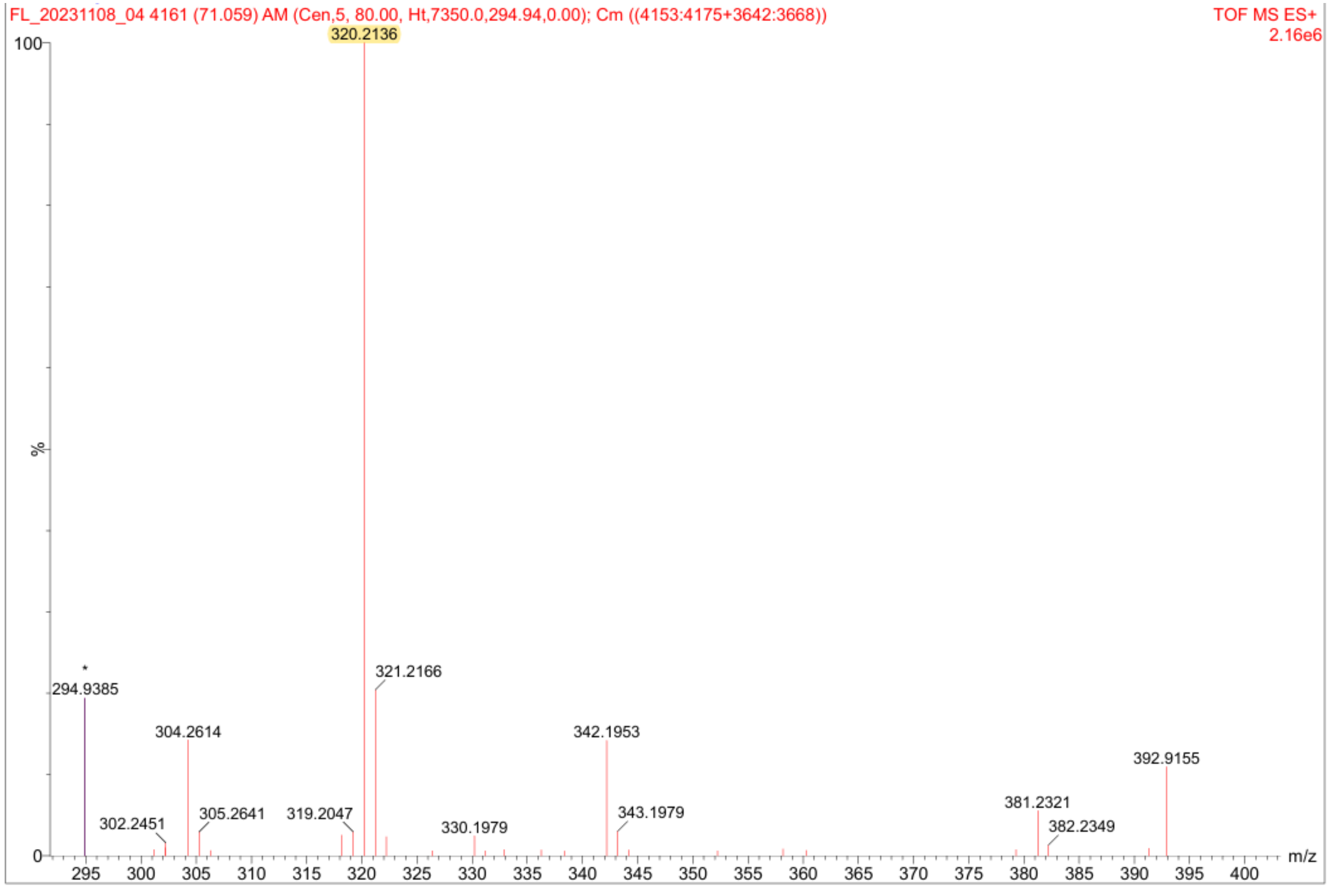

FL_20231108_04 4161 (71.059) AM (Cen,5, 80.00, Ht,7350.0,294.94,0.00); Cm ((4153:4175+3642:3668))
TOF MS ES+
2.16e6
320.2136
100
%
0
294.9385
321.2166
304.2614
342.1953
392.9155
381.2321
302.2451
305.2641
319.2047
330.1979
343.1979
382.2349
295 300 305 310 315 320 325 330 335 340 345 350 355 360 365 370 375 380 385 390 395 400
m/z

Compound 16

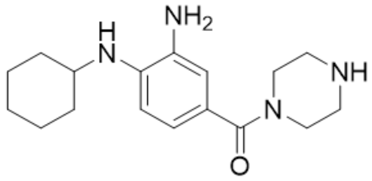

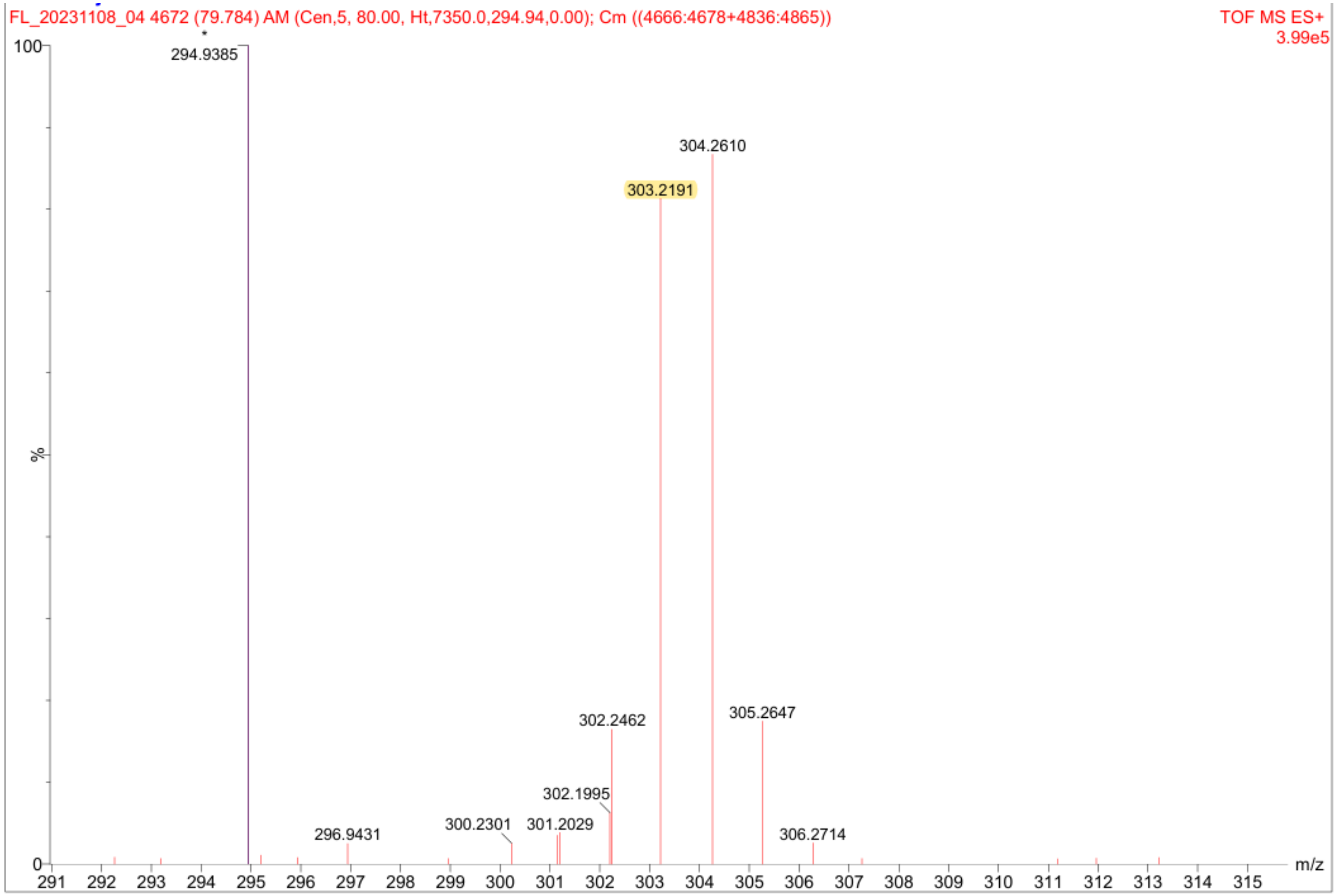

FL_20231108_04 4672 (79.784) AM (Cen,5, 80.00, Ht,7350.0,294.94,0.00); Cm ((4666:4678+4836:4865))
TOF MS ES+
3.99e5
100
%
0
294.9385
304.2610
303.2191
302.2462
305.2647
302.1995
300.2301
301.2029
296.9431
306.2714
m/z
291 292 293 294 295 296 297 298 299 300 301 302 303 304 305 306 307 308 309 310 311 312 313 314 315

Compound 35

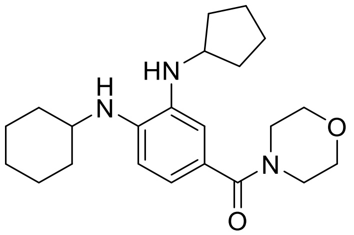

HN
H
N
N
O
O

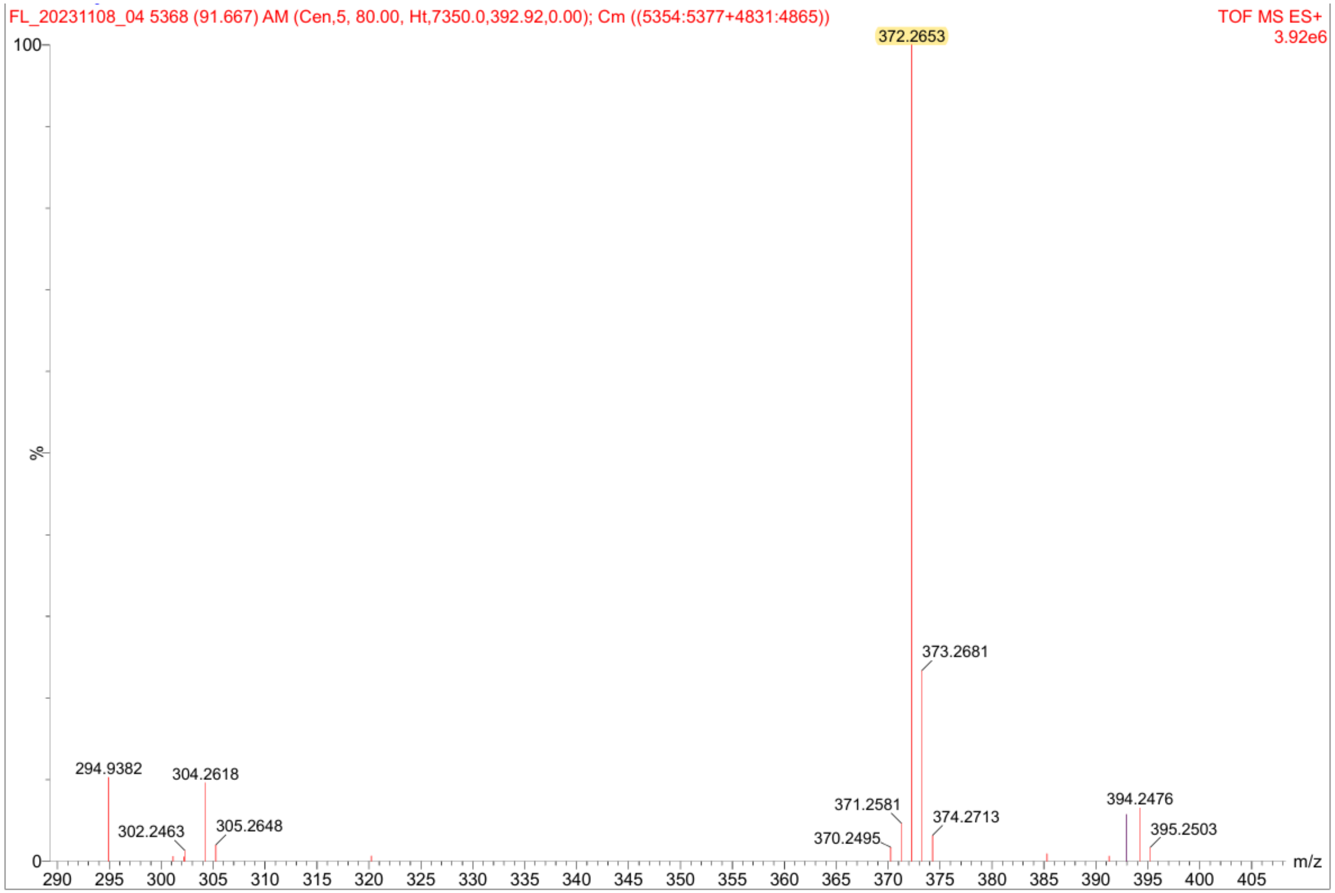

FL_20231108_04 5368 (91.667) AM (Cen,5, 80.00, Ht,7350.0,392.92,0.00); Cm ((5354:5377+4831:4865))
TOF MS ES+
3.92e6
372.2653
100
%
0
373.2681
294.9382
304.2618
302.2463
305.2648
371.2581
370.2495
374.2713
394.2476
395.2503
290 295 300 305 310 315 320 325 330 335 340 345 350 355 360 365 370 375 380 385 390 395 400 405
m/z

Compound 36

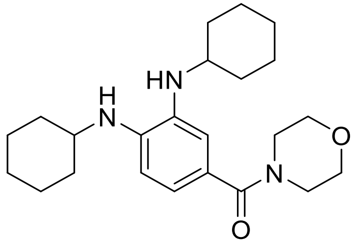

HN
H
N
N
O
O

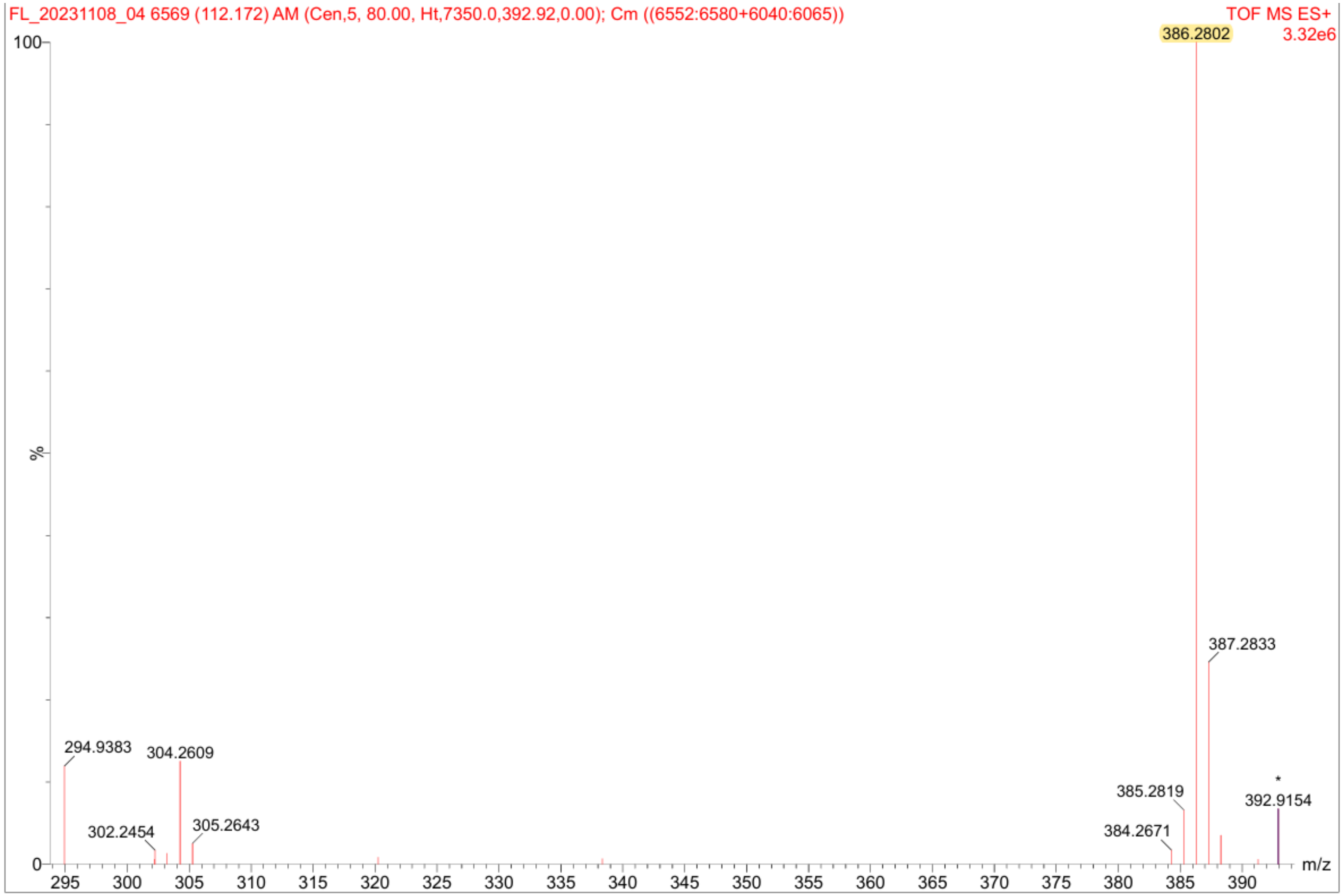

FL_20231108_04 6569 (112.172) AM (Cen,5, 80.00, Ht,7350.0,392.92,0.00); Cm ((6552:6580+6040:6065))
TOF MS ES+
3.32e6
386.2802
100
%
0
387.2833
294.9383
304.2609
385.2819
392.9154
302.2454
305.2643
384.2671
m/z
295
300
305
310
315
320
325
330
335
340
345
350
355
360
365
370
375
380
385
390

Compound 37

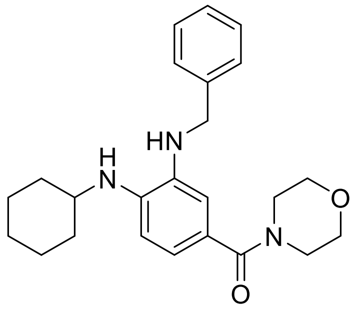

H
N
HN
N
O
O

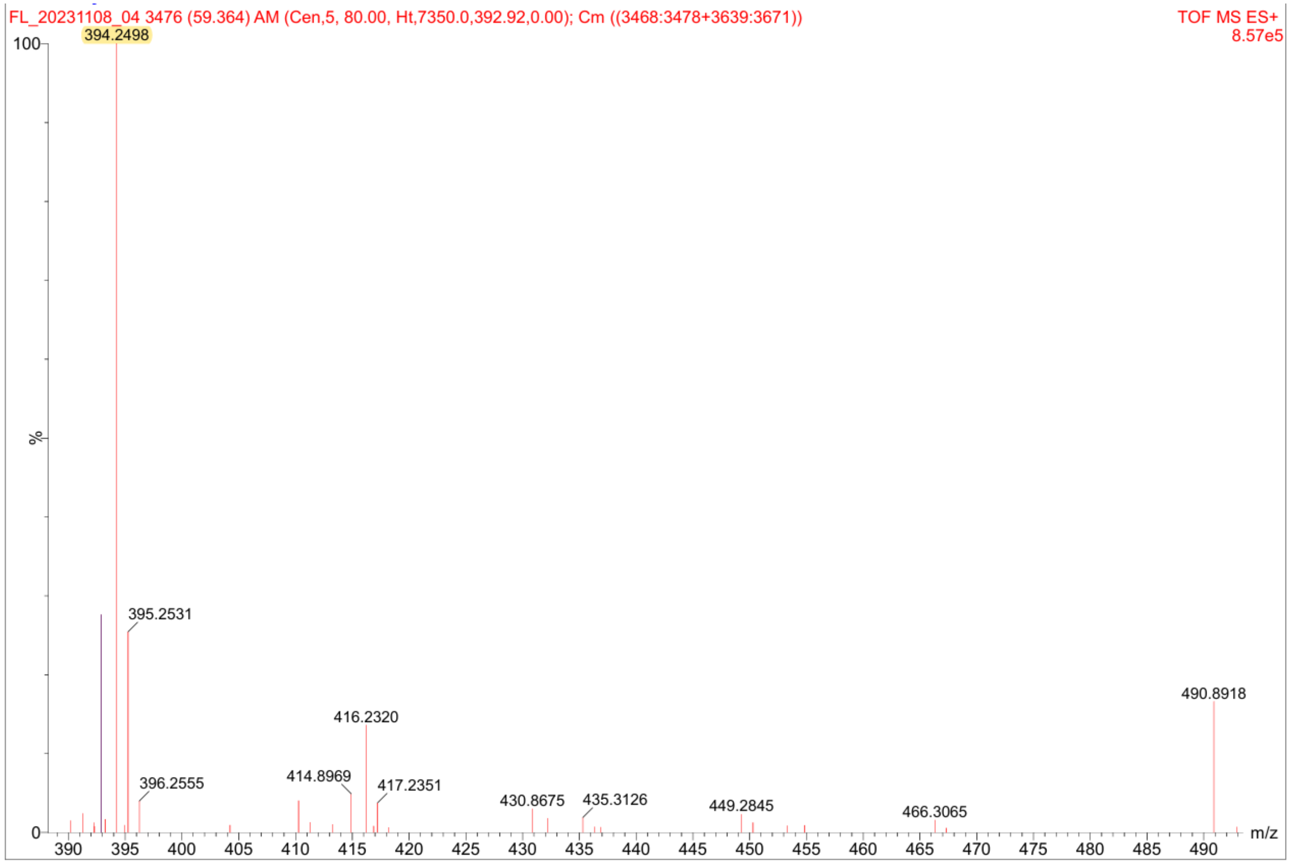

FL_20231108_04 3476 (59.364) AM (Cen,5, 80.00, Ht,7350.0,392.92,0.00); Cm ((3468:3478+3639:3671))
TOF MS ES+
8.57e5
100
%
0
394.2498
395.2531
396.2555
414.8969
416.2320
417.2351
430.8675
435.3126
449.2845
466.3065
490.8918
m/z
390
395
400
405
410
415
420
425
430
435
440
445
450
455
460
465
470
475
480
485
490

Compound 38

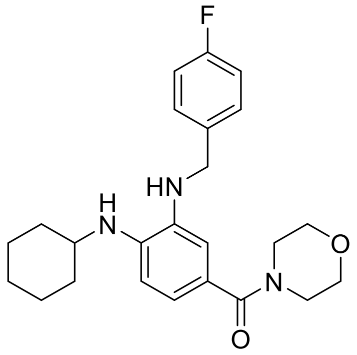

F
HN
H
N
N
O
O

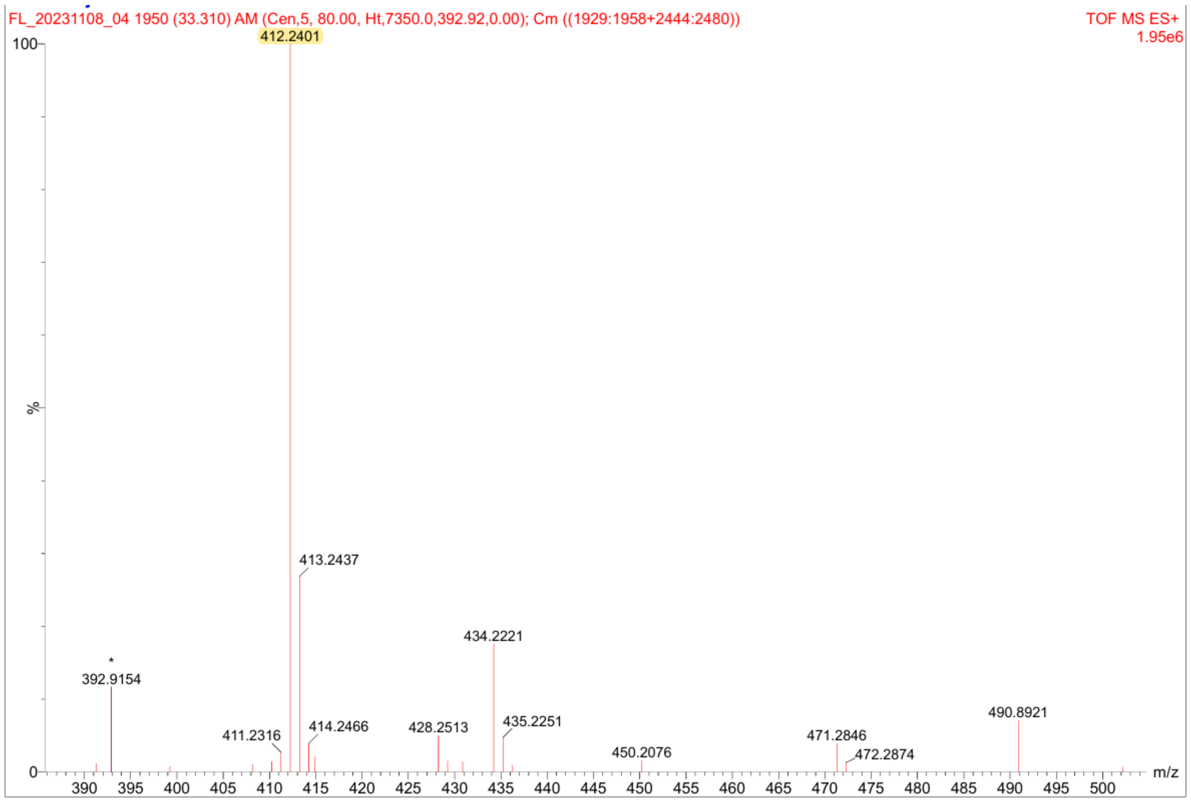

FL_20231108_04 1950 (33.310) AM (Cen,5, 80.00, Ht,7350.0,392.92,0.00); Cm ((1929:1958+2444:2480))
TOF MS ES+
1.95e6
412.2401
100
%
0
413.2437
434.2221
392.9154
411.2316
414.2466
428.2513
435.2251
450.2076
471.2846
472.2874
490.8921
390 395 400 405 410 415 420 425 430 435 440 445 450 455 460 465 470 475 480 485 490 495 500
m/z

Compound 39

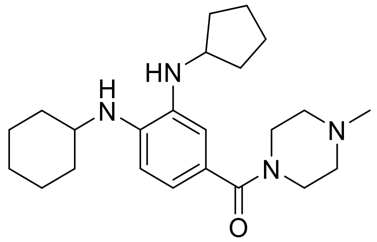
HN
H
N
N
N
O

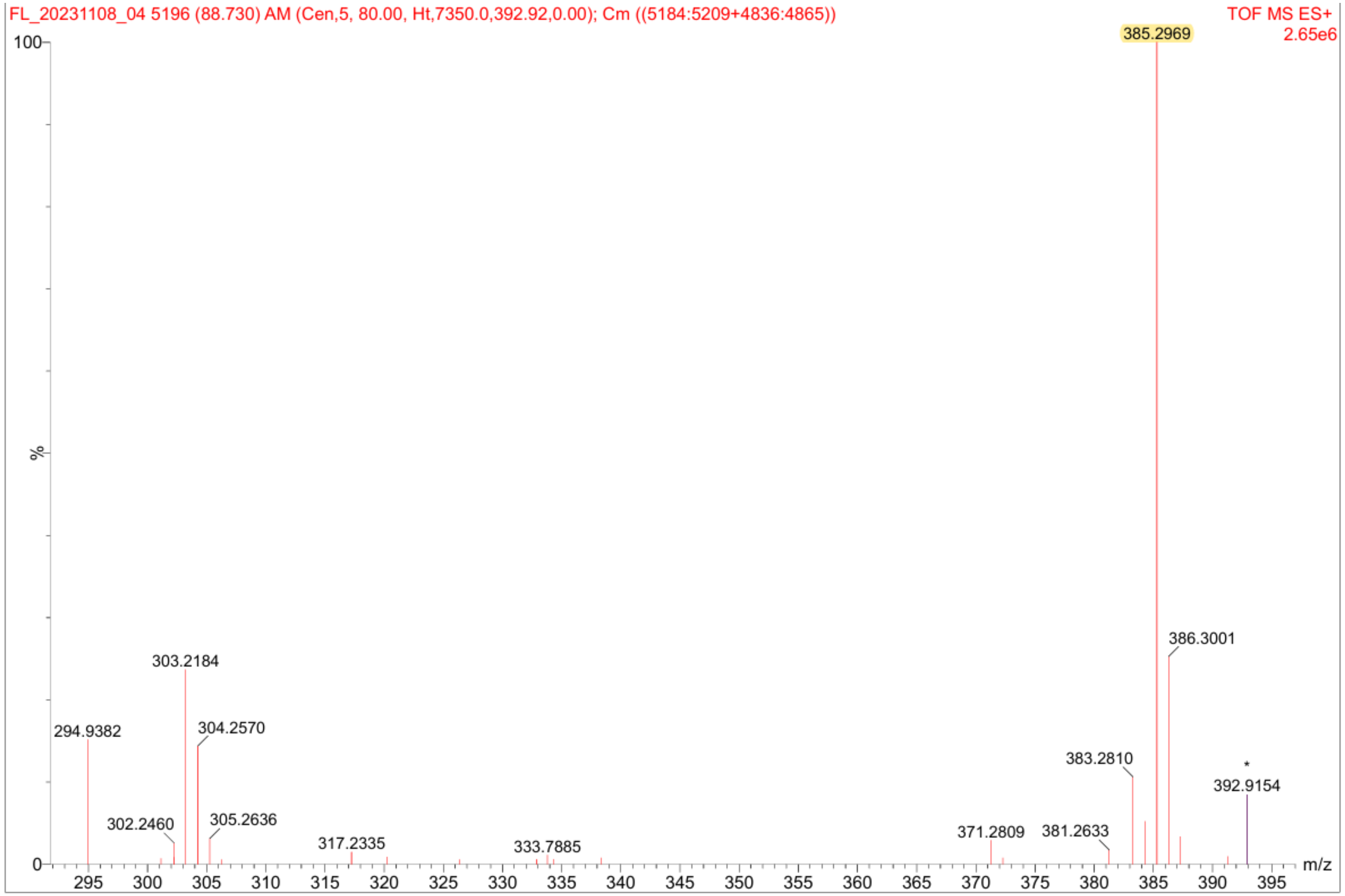
FL_20231108_04 5196 (88.730) AM (Cen,5, 80.00, Ht,7350.0,392.92,0.00); Cm ((5184:5209+4836:4865))
TOF MS ES+
2.65e6
385.2969
100
%
0
386.3001
303.2184
294.9382
304.2570
383.2810
392.9154
302.2460
305.2636
317.2335
333.7885
371.2809
381.2633
m/z
295
300
305
310
315
320
325
330
335
340
345
350
355
360
365
370
375
380
385
390
395

Compound 40

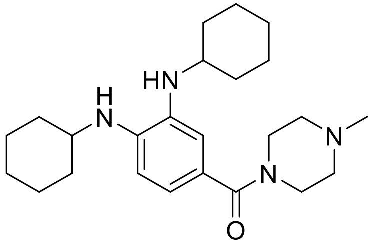

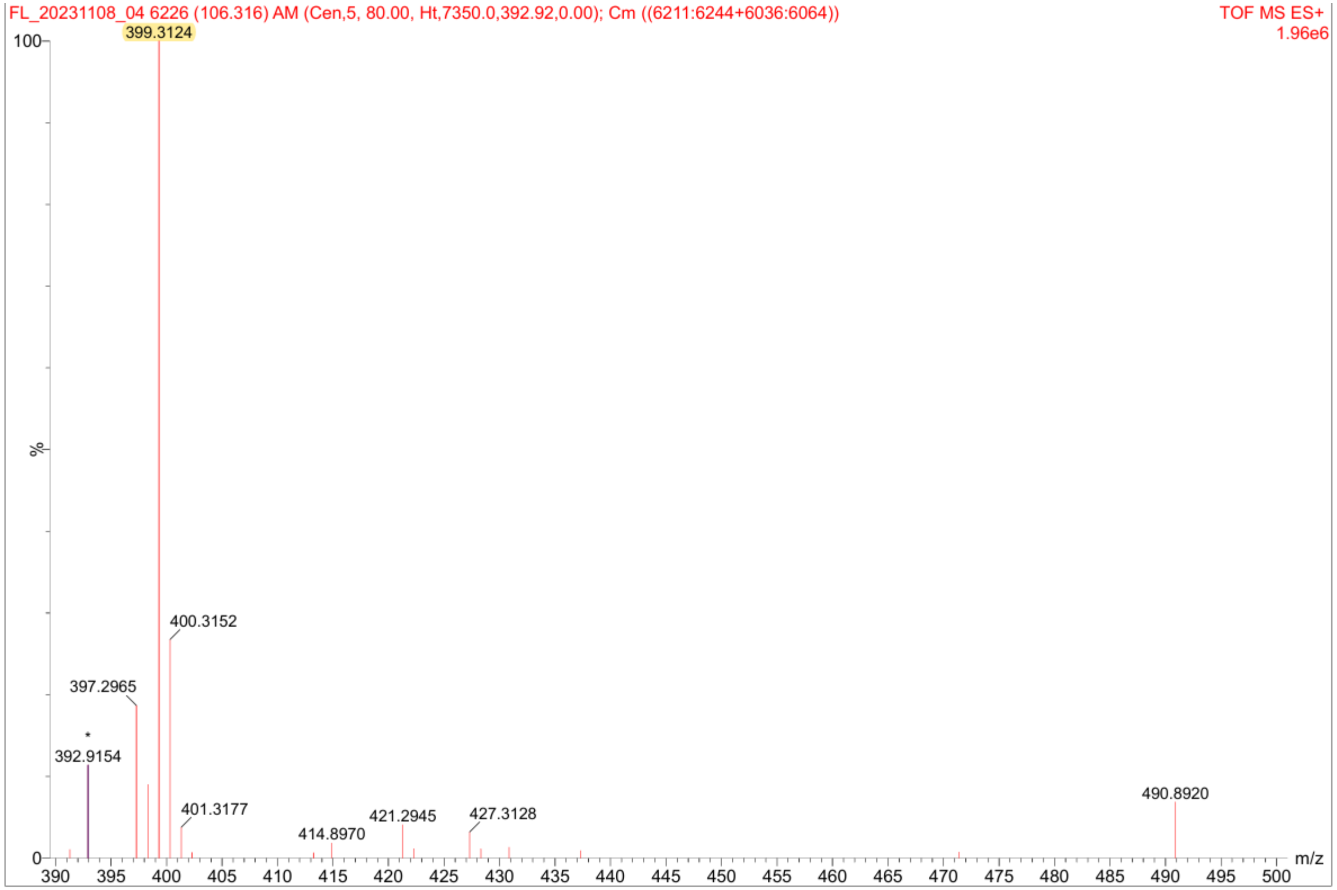
FL_20231108_04 6226 (106.316) AM (Cen,5, 80.00, Ht,7350.0,392.92,0.00); Cm ((6211:6244+6036:6064))
TOF MS ES+
1.96e6
399.3124
400.3152
397.2965
392.9154
401.3177
414.8970
421.2945
427.3128
490.8920
%
m/z
100
0
390 395 400 405 410 415 420 425 430 435 440 445 450 455 460 465 470 475 480 485 490 495 500

Ccompound 41

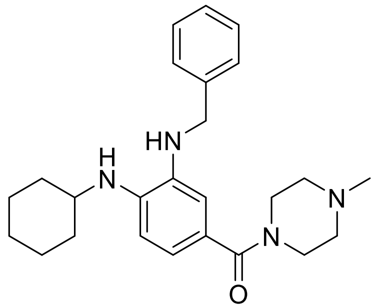

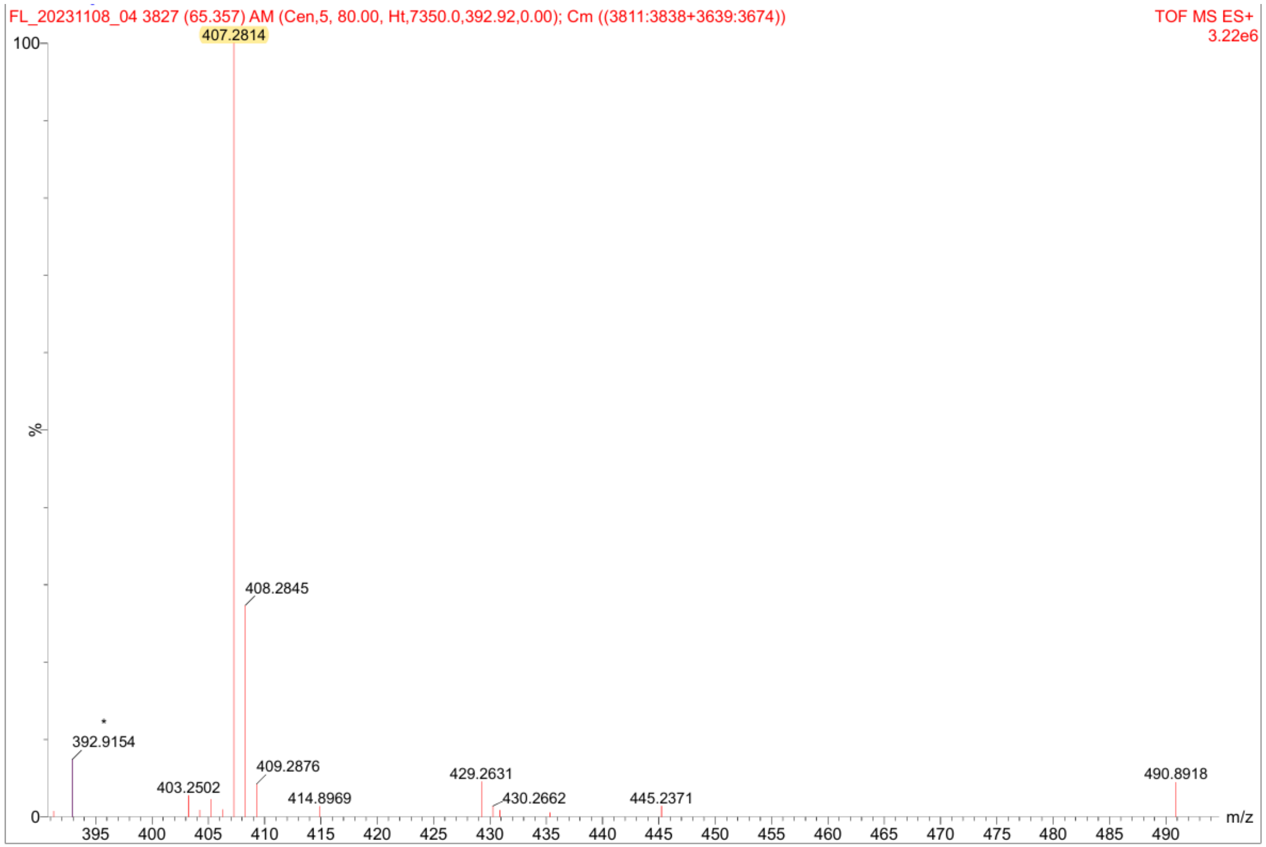

Compound 42

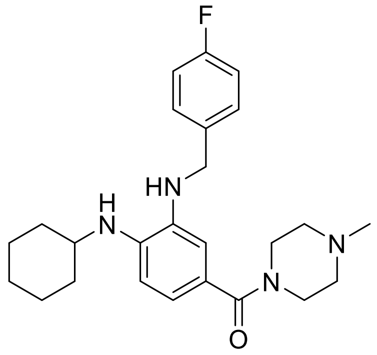

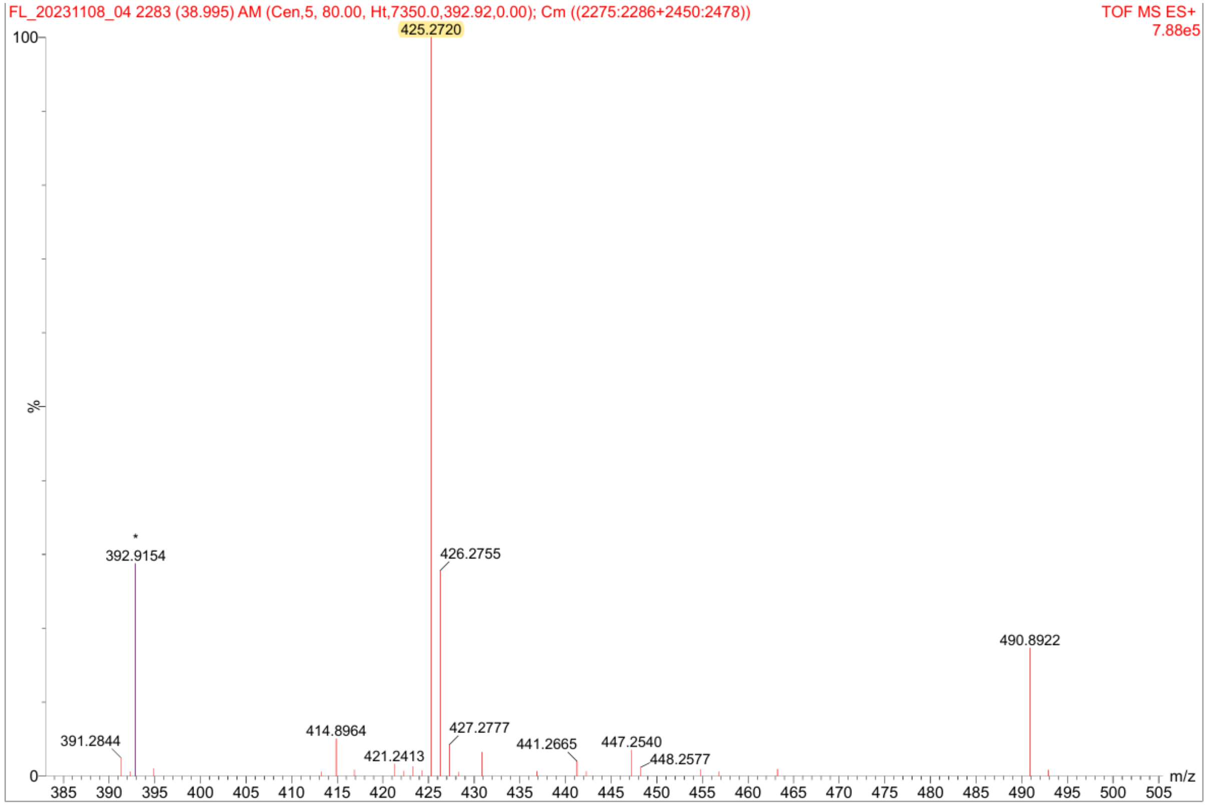

FL_20231108_04 2283 (38.995) AM (Cen,5, 80.00, Ht,7350.0,392.92,0.00); Cm ((2275:2286+2450:2478))
TOF MS ES+
7.88e5
425.2720
100
%
0
392.9154
426.2755
490.8922
391.2844
414.8964
421.2413
427.2777
441.2665
447.2540
448.2577
m/z
385 390 395 400 405 410 415 420 425 430 435 440 445 450 455 460 465 470 475 480 485 490 495 500 505

Compound 43

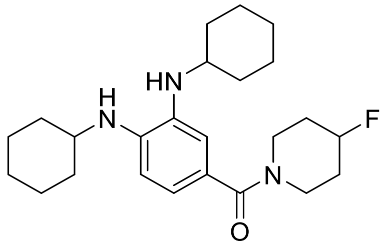

HN
H
N
F
N
O

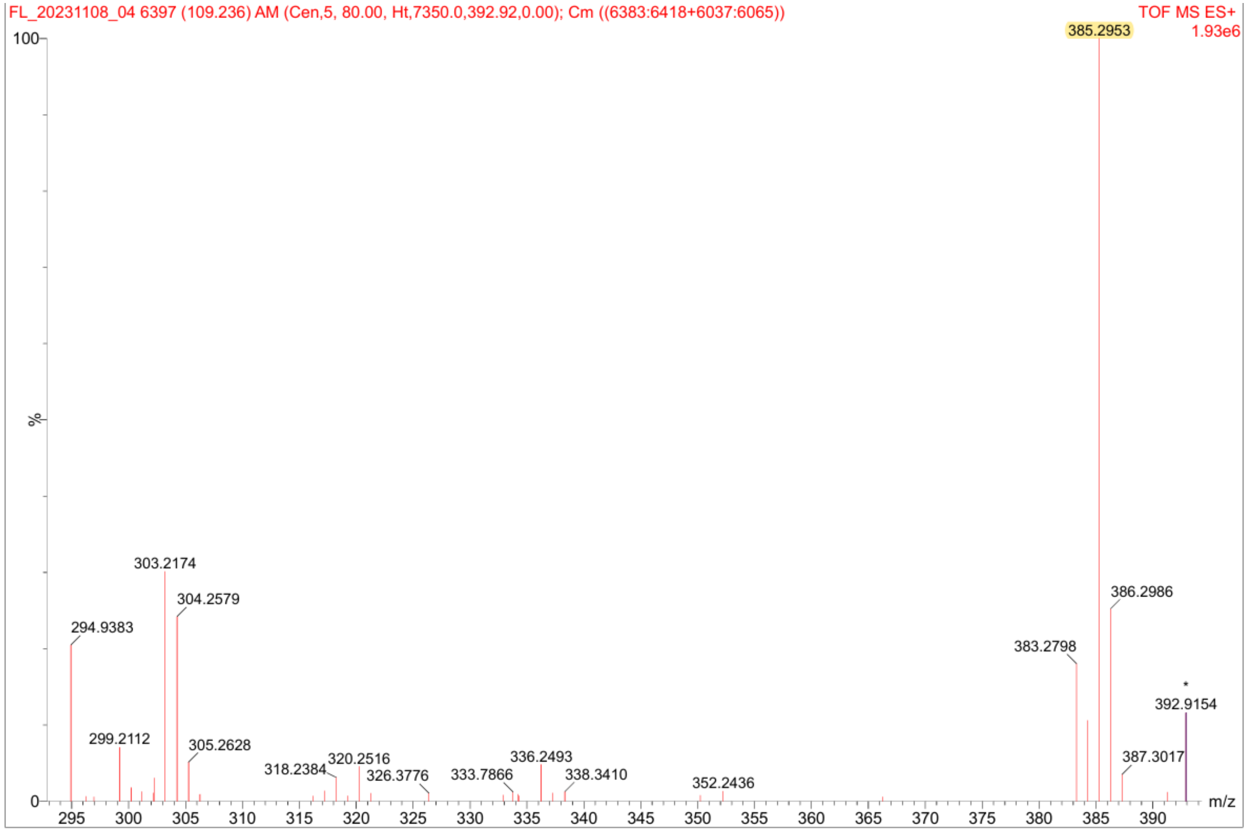

FL_20231108_04 6397 (109.236) AM (Cen,5, 80.00, Ht,7350.0,392.92,0.00); Cm ((6383:6418+6037:6065))
TOF MS ES+
1.93e6
385.2953
100
%
0
303.2174
304.2579
294.9383
386.2986
383.2798
392.9154
299.2112
305.2628
318.2384
320.2516
326.3776
333.7866
336.2493
338.3410
352.2436
387.3017
m/z
295
300
305
310
315
320
325
330
335
340
345
350
355
360
365
370
375
380
385
390

Compound 44

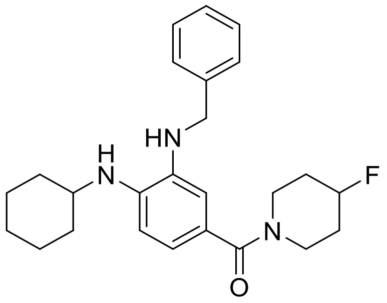

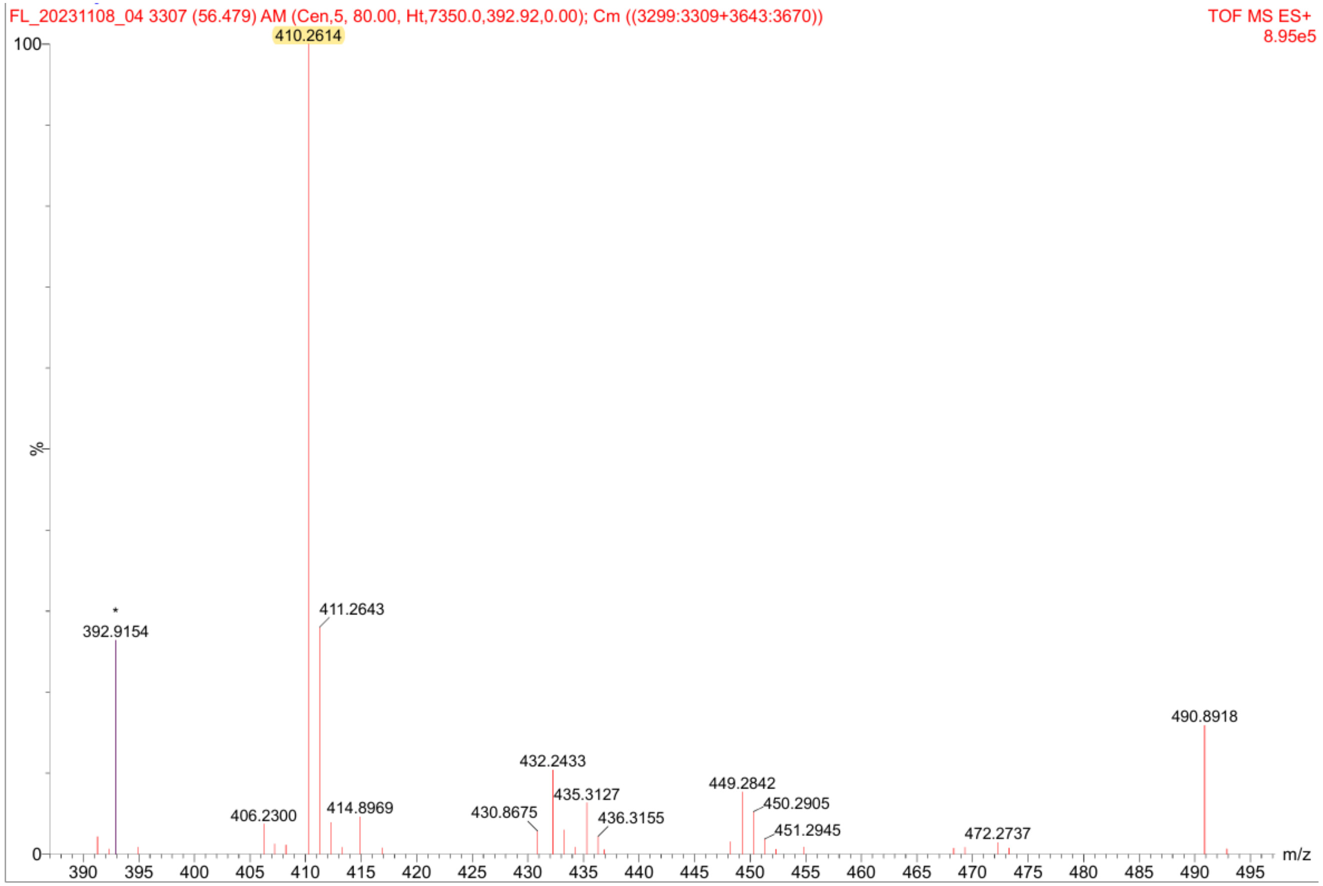

FL_20231108_04 3307 (56.479) AM (Cen,5, 80.00, Ht,7350.0,392.92,0.00); Cm ((3299:3309+3643:3670))
TOF MS ES+
8.95e5
100
%
0
410.2614
411.2643
392.9154
490.8918
432.2433
435.3127
449.2842
450.2905
406.2300
414.8969
430.8675
436.3155
451.2945
472.2737
m/z
390
395
400
405
410
415
420
425
430
435
440
445
450
455
460
465
470
475
480
485
490
495

Compound 45

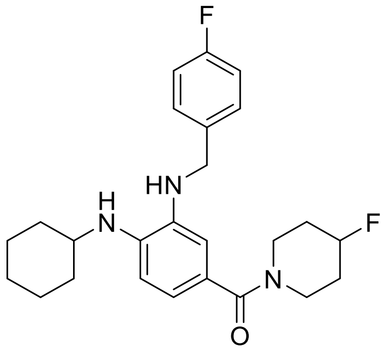

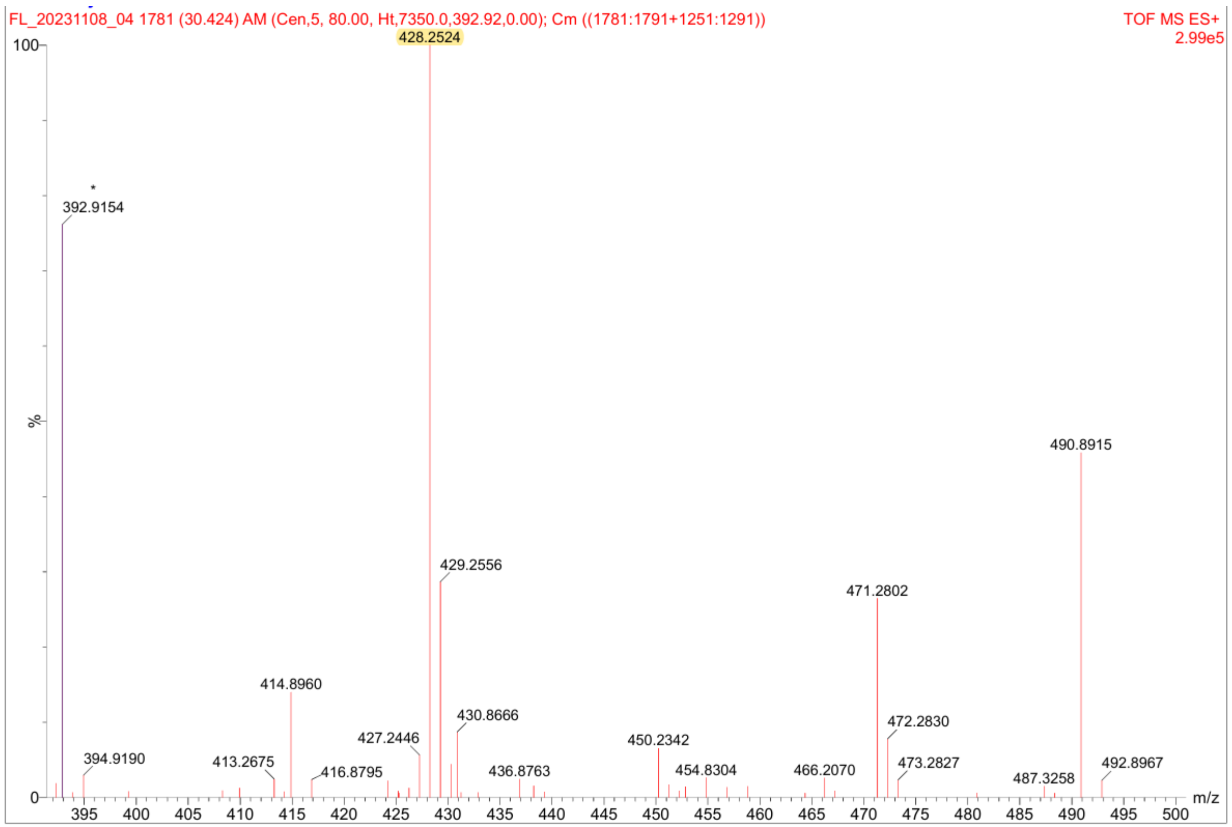
FL_20231108_04 1781 (30.424) AM (Cen,5, 80.00, Ht,7350.0,392.92,0.00); Cm ((1781:1791+1251:1291))
TOF MS ES+
2.99e5
100
%
0
428.2524
392.9154
490.8915
429.2556
471.2802
414.8960
430.8666
472.2830
427.2446
450.2342
394.9190
413.2675
416.8795
436.8763
454.8304
466.2070
473.2827
487.3258
492.8967
395
400
405
410
415
420
425
430
435
440
445
450
455
460
465
470
475
480
485
490
495
500
m/z

Compound 46

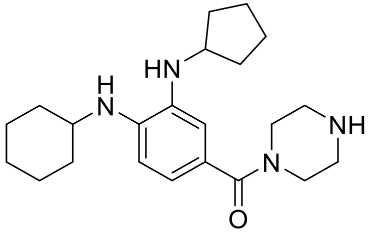

HN
H
N
NH
N
O

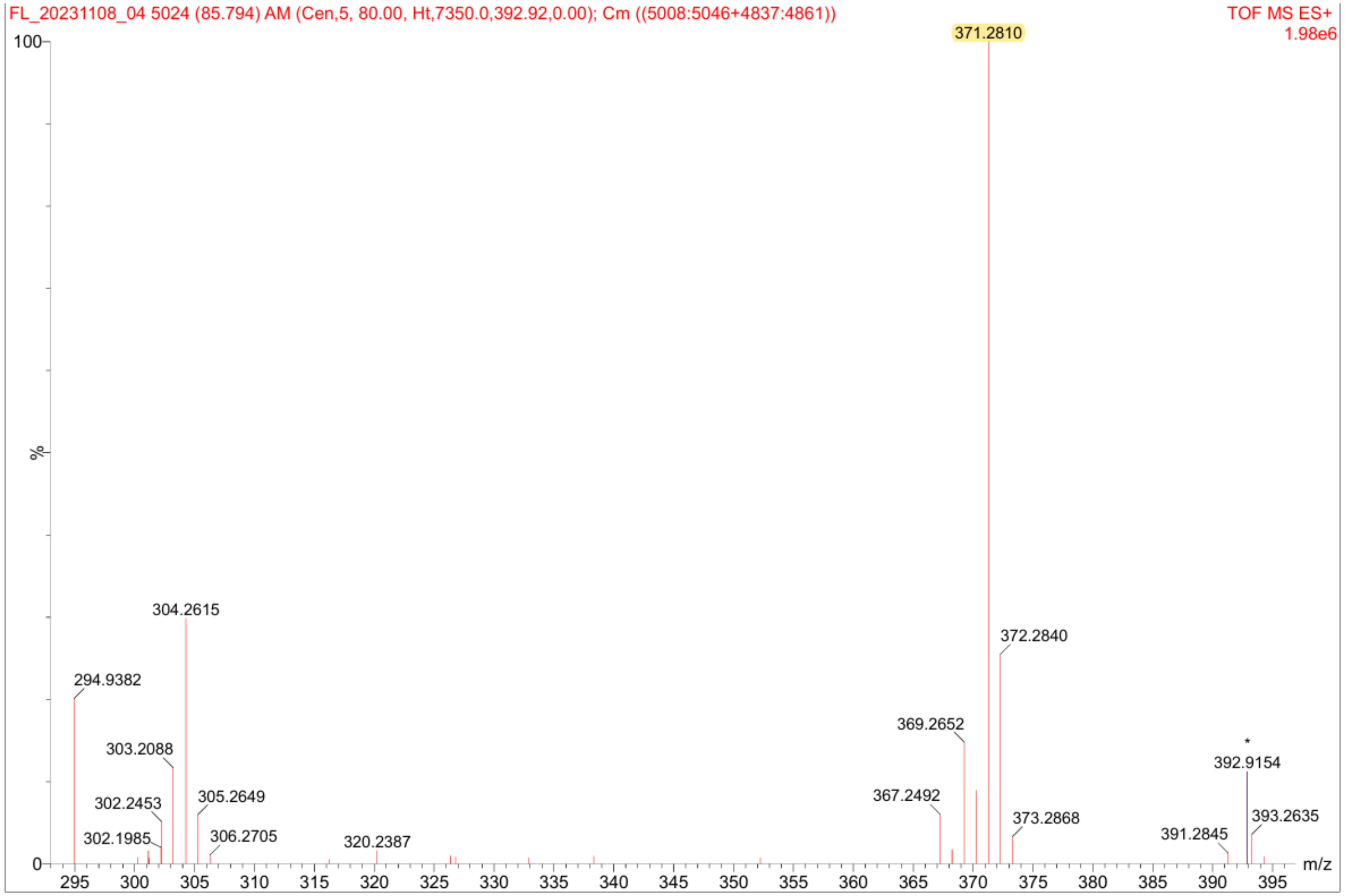

FL_20231108_04 5024 (85.794) AM (Cen,5, 80.00, Ht,7350.0,392.92,0.00); Cm ((5008:5046+4837:4861))
TOF MS ES+
1.98e6
371.2810
100
%
0
304.2615
294.9382
303.2088
302.2453
302.1985
305.2649
306.2705
320.2387
372.2840
369.2652
367.2492
373.2868
392.9154
393.2635
391.2845
m/z
295
300
305
310
315
320
325
330
335
340
345
350
355
360
365
370
375
380
385
390
395

Compound 47

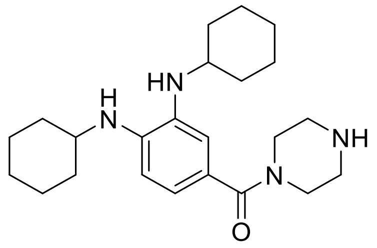
HN
H
N
NH
N
O

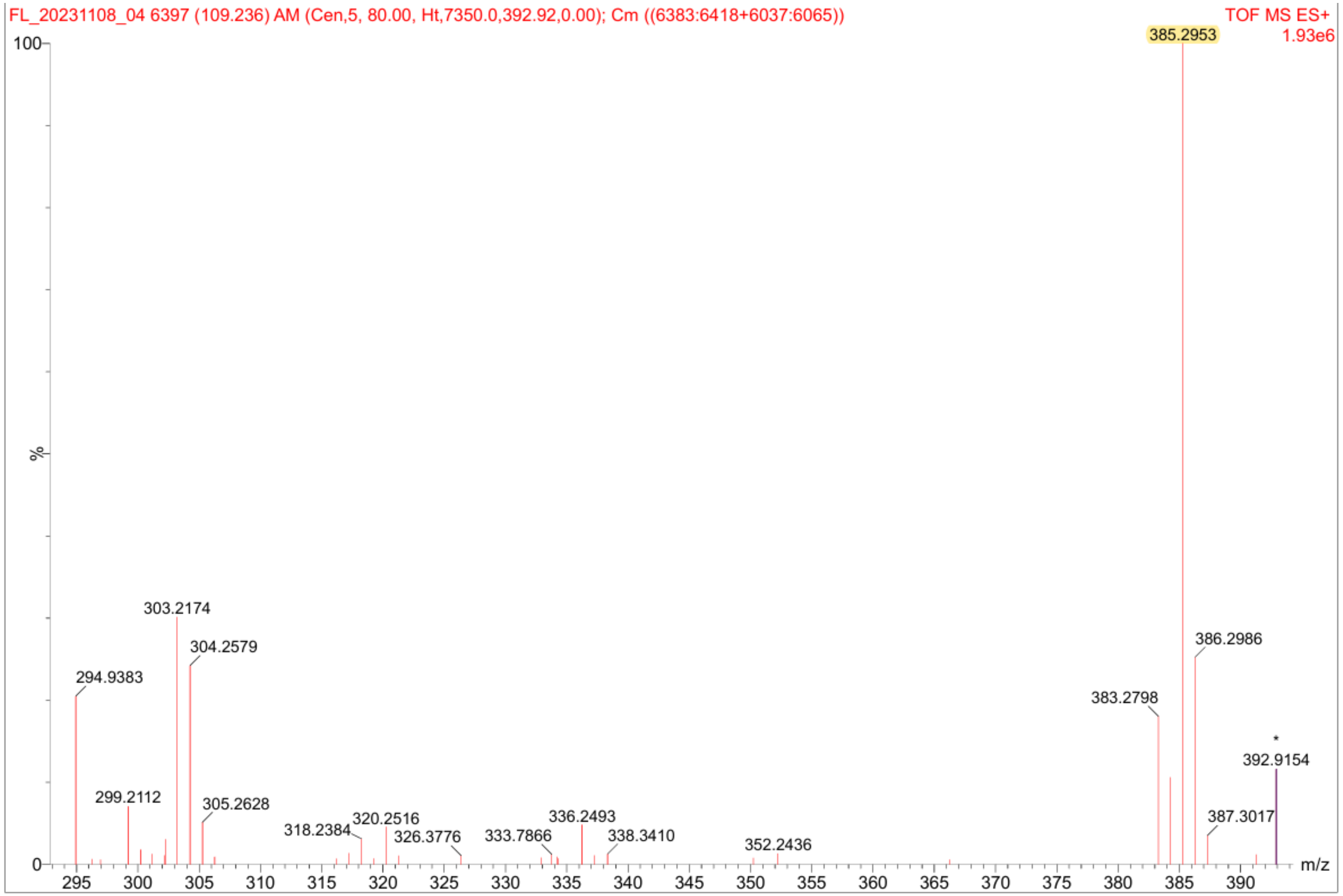
FL_20231108_04 6397 (109.236) AM (Cen,5, 80.00, Ht,7350.0,392.92,0.00); Cm ((6383:6418+6037:6065))
TOF MS ES+
1.93e6
385.2953
100
%
0
303.2174
304.2579
294.9383
299.2112
305.2628
318.2384
320.2516
326.3776
333.7866
336.2493
338.3410
352.2436
383.2798
386.2986
392.9154
387.3017
m/z
295
300
305
310
315
320
325
330
335
340
345
350
355
360
365
370
375
380
385
390

Compound 48

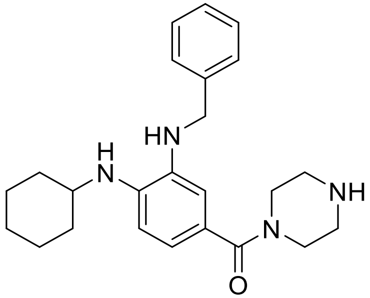

HN
H
N
NH
N
O

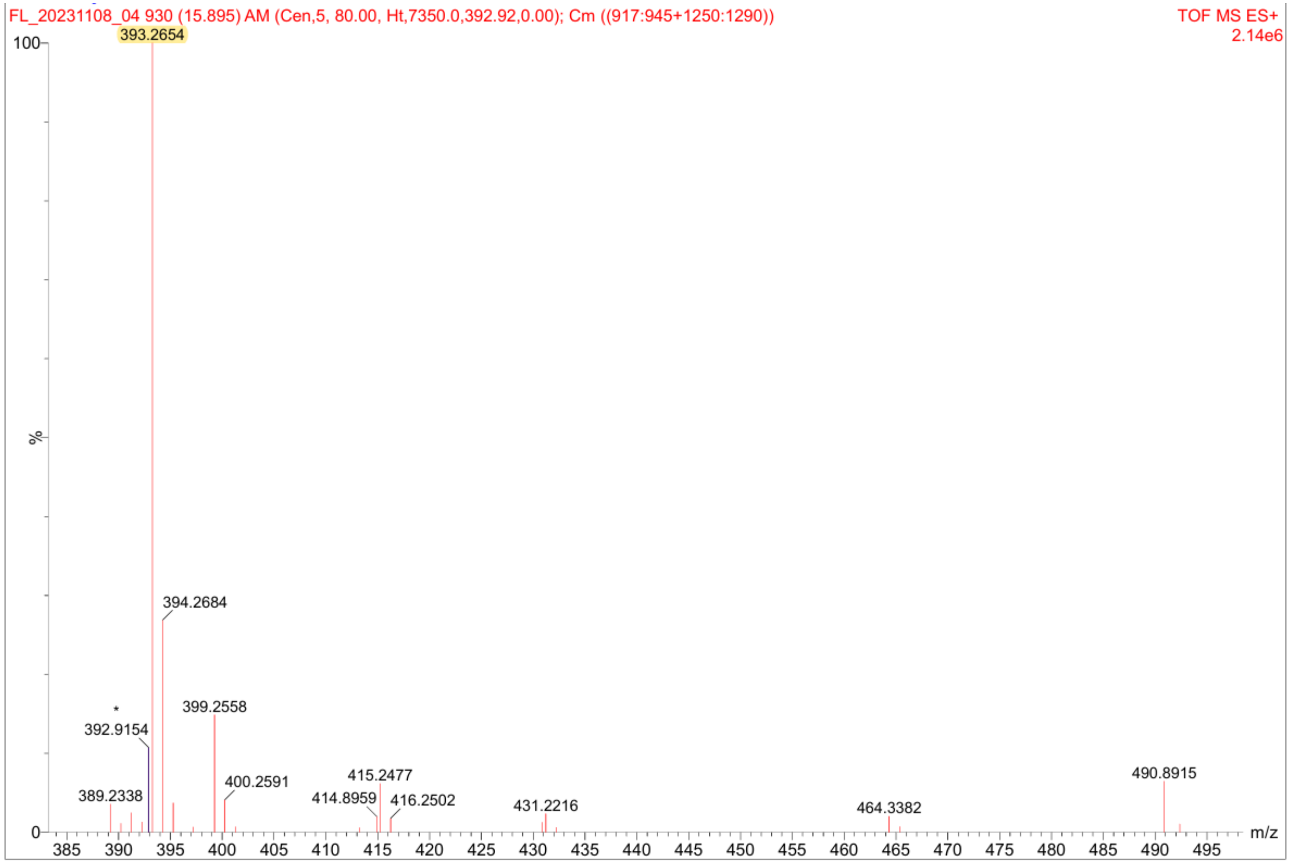

FL_20231108_04 930 (15.895) AM (Cen,5, 80.00, Ht,7350.0,392.92,0.00); Cm ((917:945+1250:1290))
TOF MS ES+
2.14e6
393.2654
100
%
0
394.2684
399.2558
392.9154
389.2338
400.2591
415.2477
414.8959
416.2502
431.2216
464.3382
490.8915
m/z
385 390 395 400 405 410 415 420 425 430 435 440 445 450 455 460 465 470 475 480 485 490 495

Compound 49

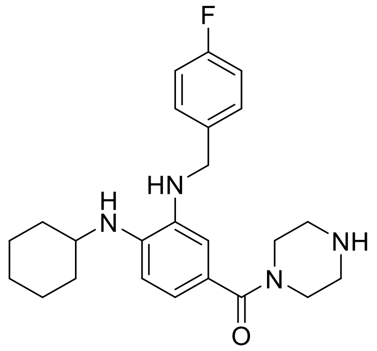

F
HN
H
N
NH
N
O

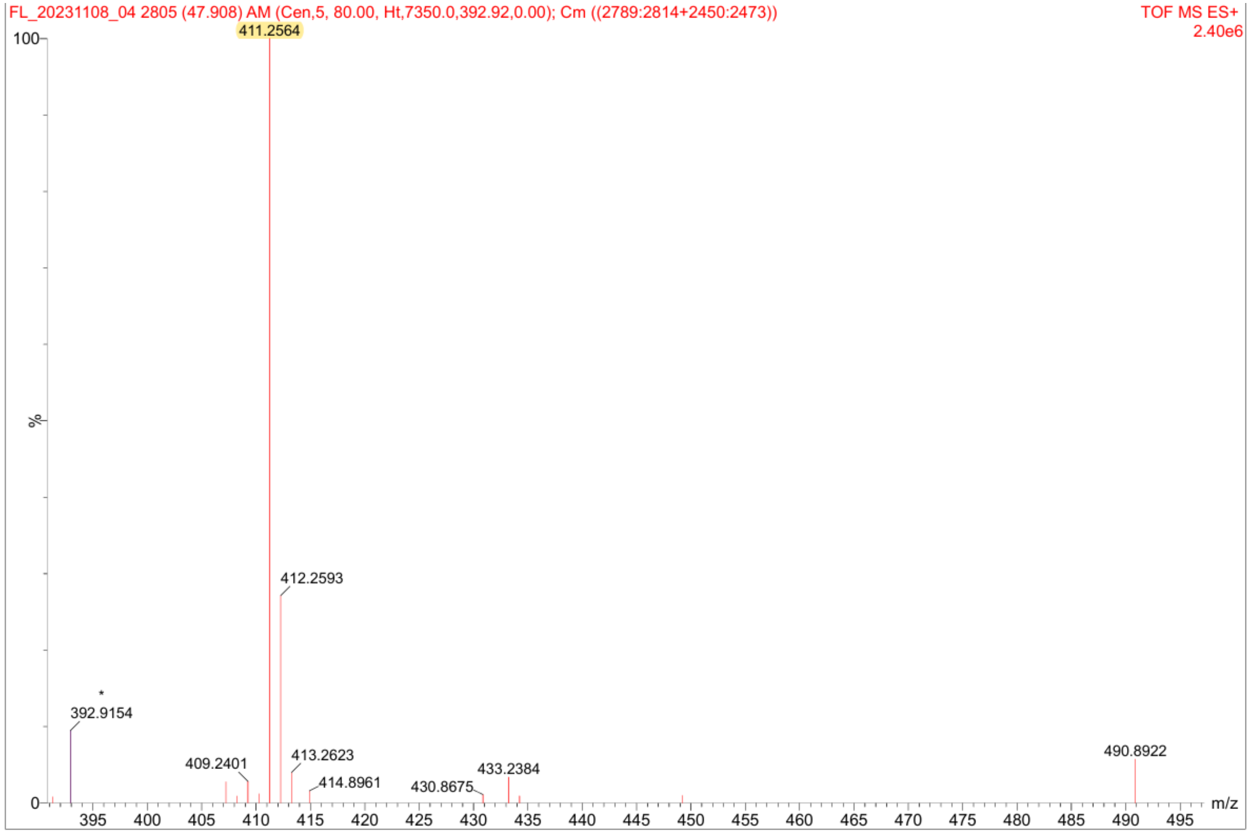

FL_20231108_04 2805 (47.908) AM (Cen,5, 80.00, Ht,7350.0,392.92,0.00); Cm ((2789:2814+2450:2473))
TOF MS ES+
2.40e6
411.2564
100
%
0
412.2593
392.9154
409.2401
413.2623
414.8961
430.8675
433.2384
490.8922
395
400
405
410
415
420
425
430
435
440
445
450
455
460
465
470
475
480
485
490
495
m/z

Compound 50

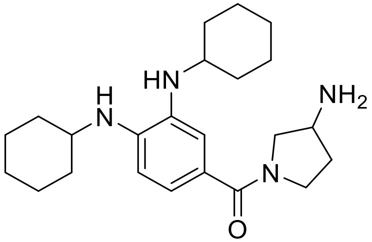

HN
H
N
NH2
N
O

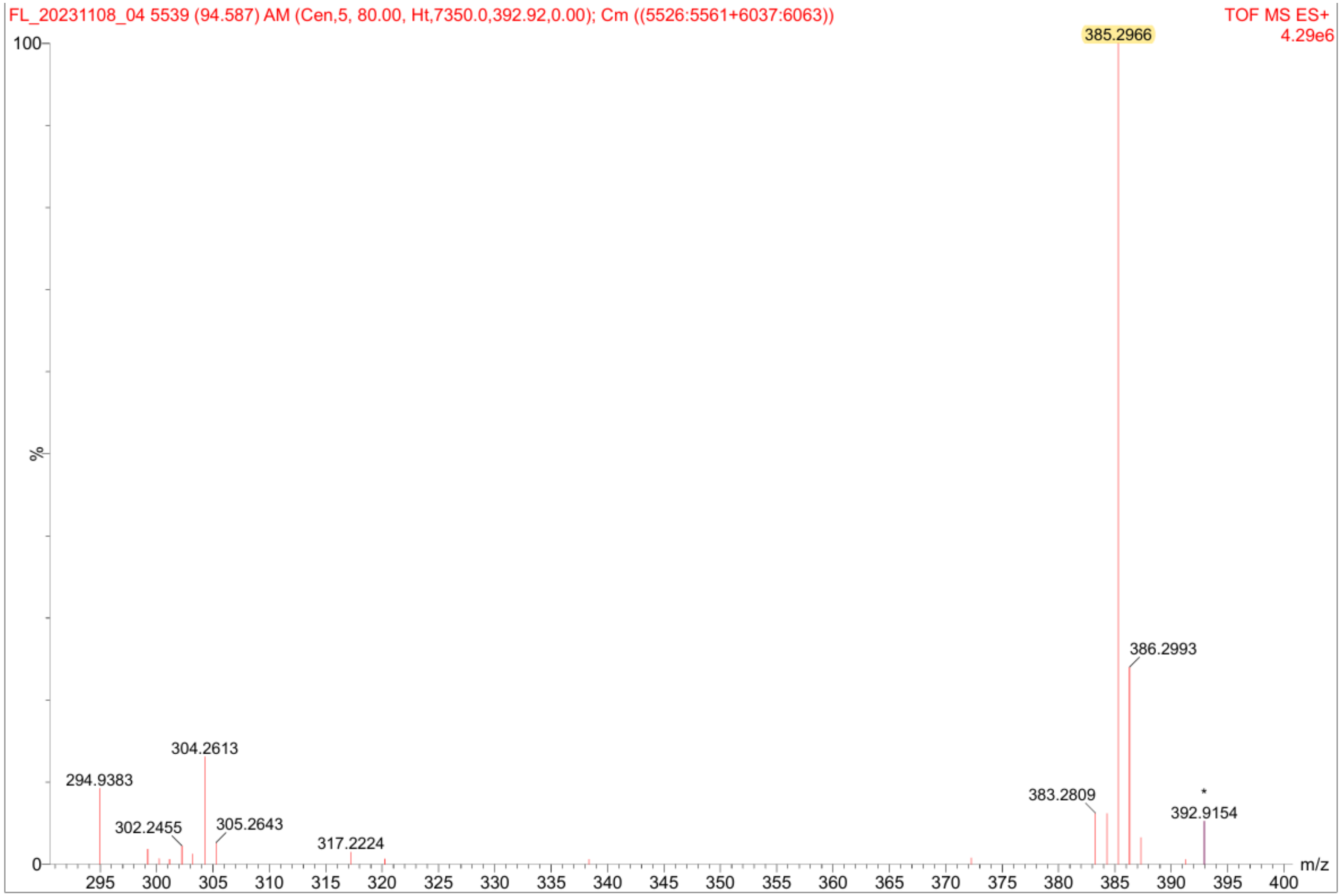

FL_20231108_04 5539 (94.587) AM (Cen,5, 80.00, Ht,7350.0,392.92,0.00); Cm ((5526:5561+6037:6063))
TOF MS ES+
4.29e6
385.2966
100
%
0
386.2993
304.2613
294.9383
383.2809
392.9154
302.2455
305.2643
317.2224
295 300 305 310 315 320 325 330 335 340 345 350 355 360 365 370 375 380 385 390 395 400
m/z

Compound 51

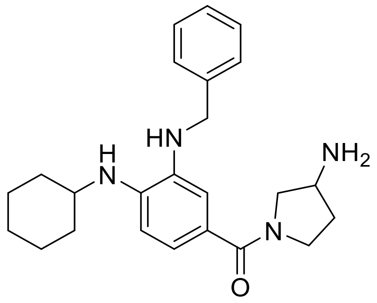
HN
H
N
NH2
N
O

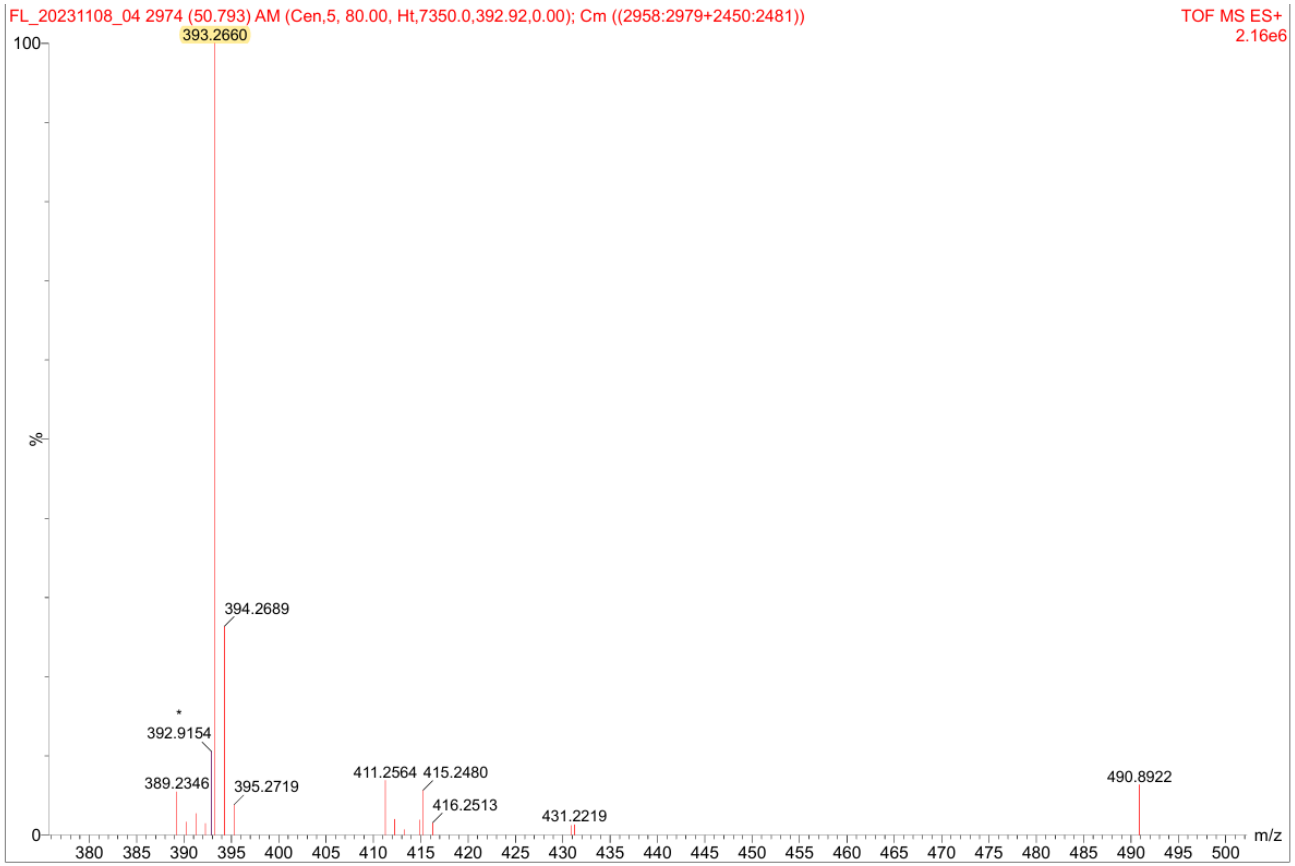
FL_20231108_04 2974 (50.793) AM (Cen,5, 80.00, Ht,7350.0,392.92,0.00); Cm ((2958:2979+2450:2481))
TOF MS ES+
2.16e6
100
%
0
393.2660
394.2689
392.9154
389.2346
395.2719
411.2564
415.2480
416.2513
431.2219
490.8922
380 385 390 395 400 405 410 415 420 425 430 435 440 445 450 455 460 465 470 475 480 485 490 495 500
m/z

Compound 73

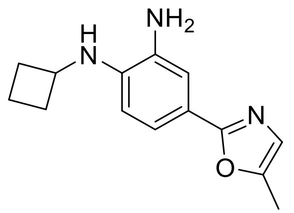

NH2
H
N
N
O

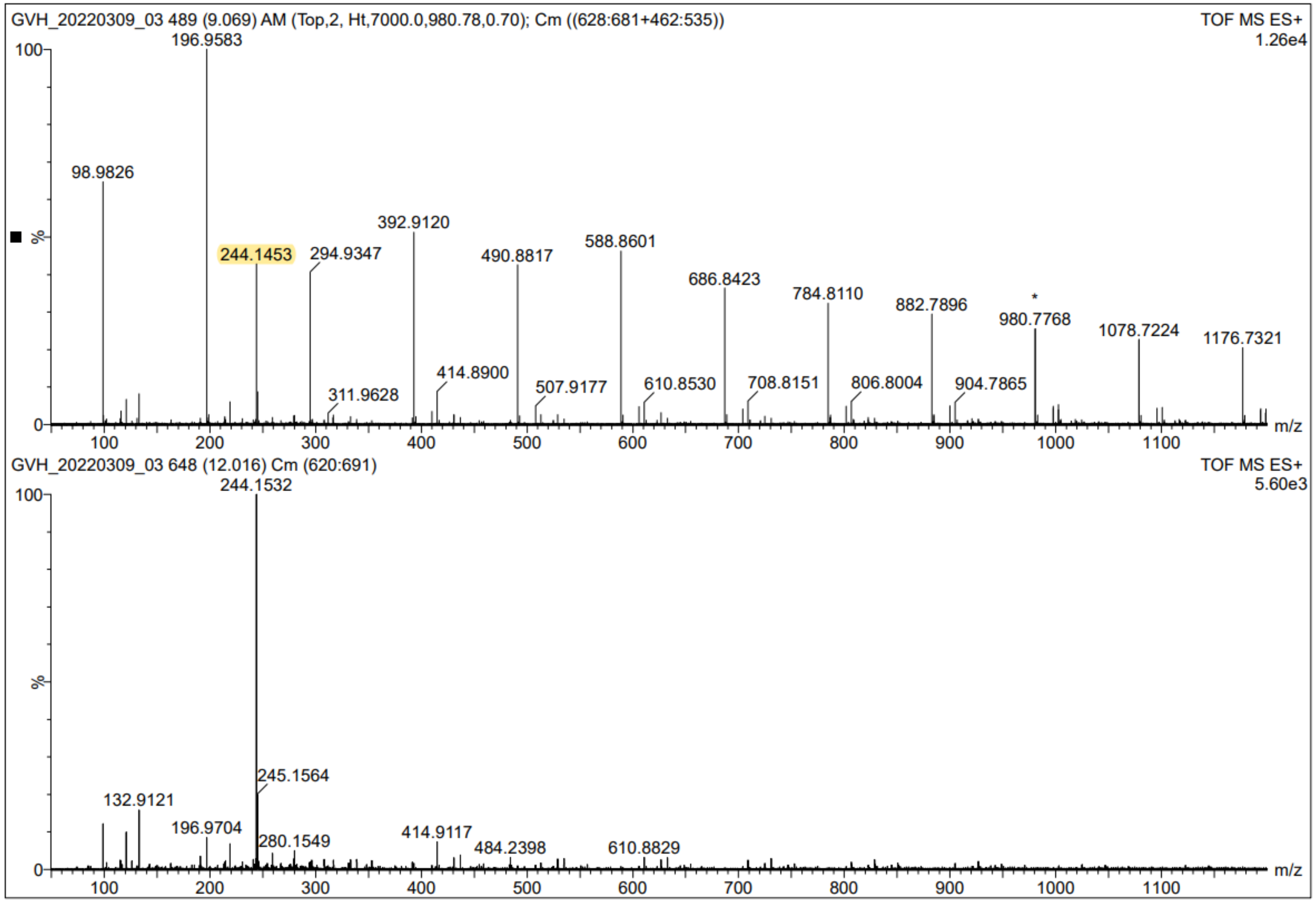

GVH_20220309_03 489 (9.069) AM (Top,2, Ht,7000.0,980.78,0.70); Cm ((628:681+462:535))
TOF MS ES+
1.26e4
196.9583
98.9826
392.9120
244.1453
294.9347
490.8817
588.8601
686.8423
784.8110
882.7896
980.7768
1078.7224
1176.7321
414.8900
311.9628
507.9177
610.8530
708.8151
806.8004
904.7865
m/z
GVH_20220309_03 648 (12.016) Cm (620:691)
TOF MS ES+
5.60e3
244.1532
245.1564
132.9121
196.9704
280.1549
414.9117
484.2398
610.8829

Compound 74

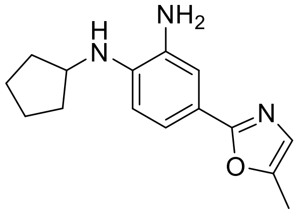

NH2
H
N
N
O

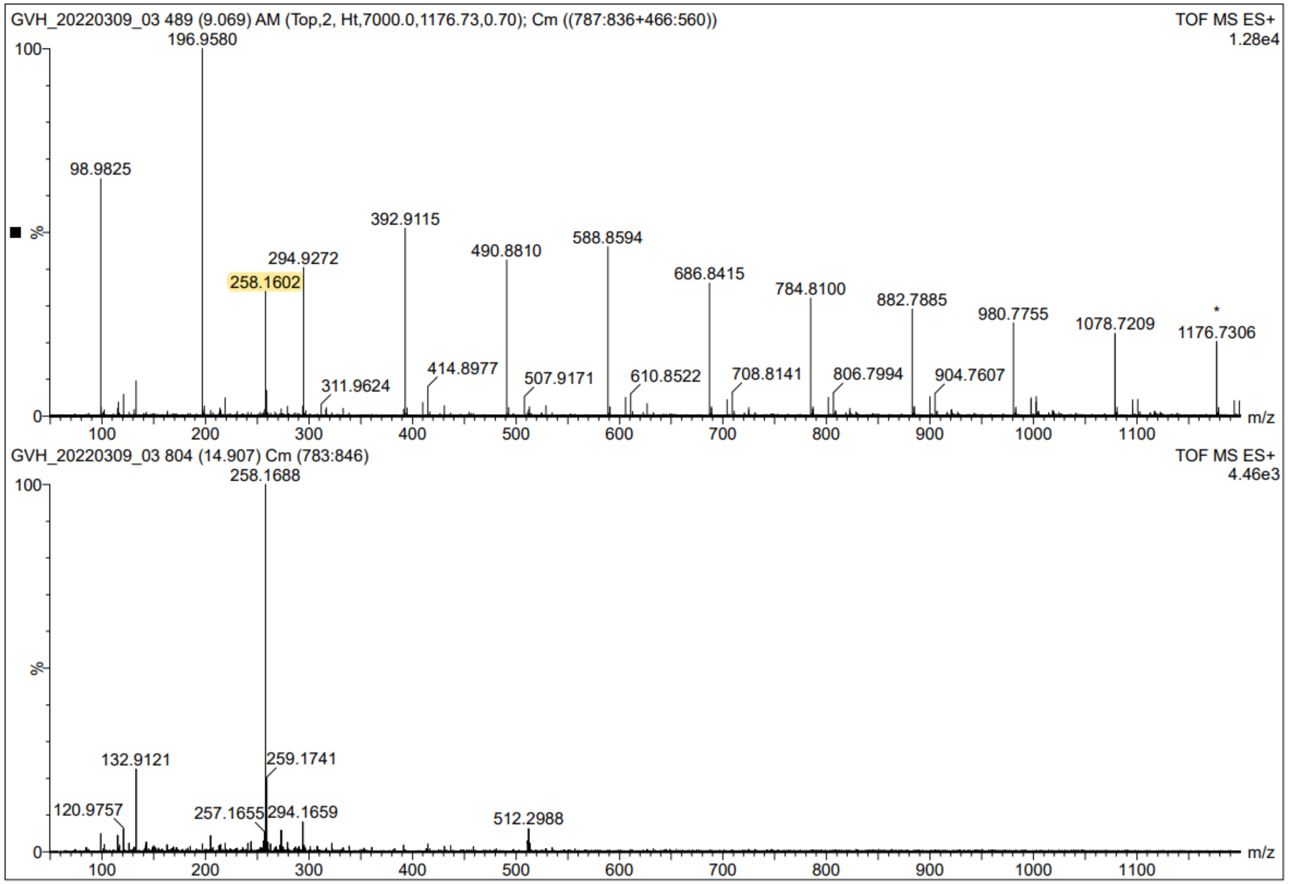

GVH_20220309_03 489 (9.069) AM (Top,2, Ht,7000.0,1176.73,0.70); Cm ((787:836+466:560))
TOF MS ES+
1.28e4
196.9580
98.9825
392.9115
490.8810
588.8594
294.9272
258.1602
686.8415
784.8100
882.7885
980.7755
1078.7209
1176.7306
311.9624
414.8977
507.9171
610.8522
708.8141
806.7994
904.7607
m/z
GVH_20220309_03 804 (14.907) Cm (783:846)
TOF MS ES+
4.46e3
258.1688
132.9121
259.1741
120.9757
257.1655
294.1659
512.2988
m/z

Compound 75

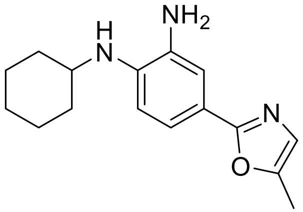

NH2
H
N
N
O

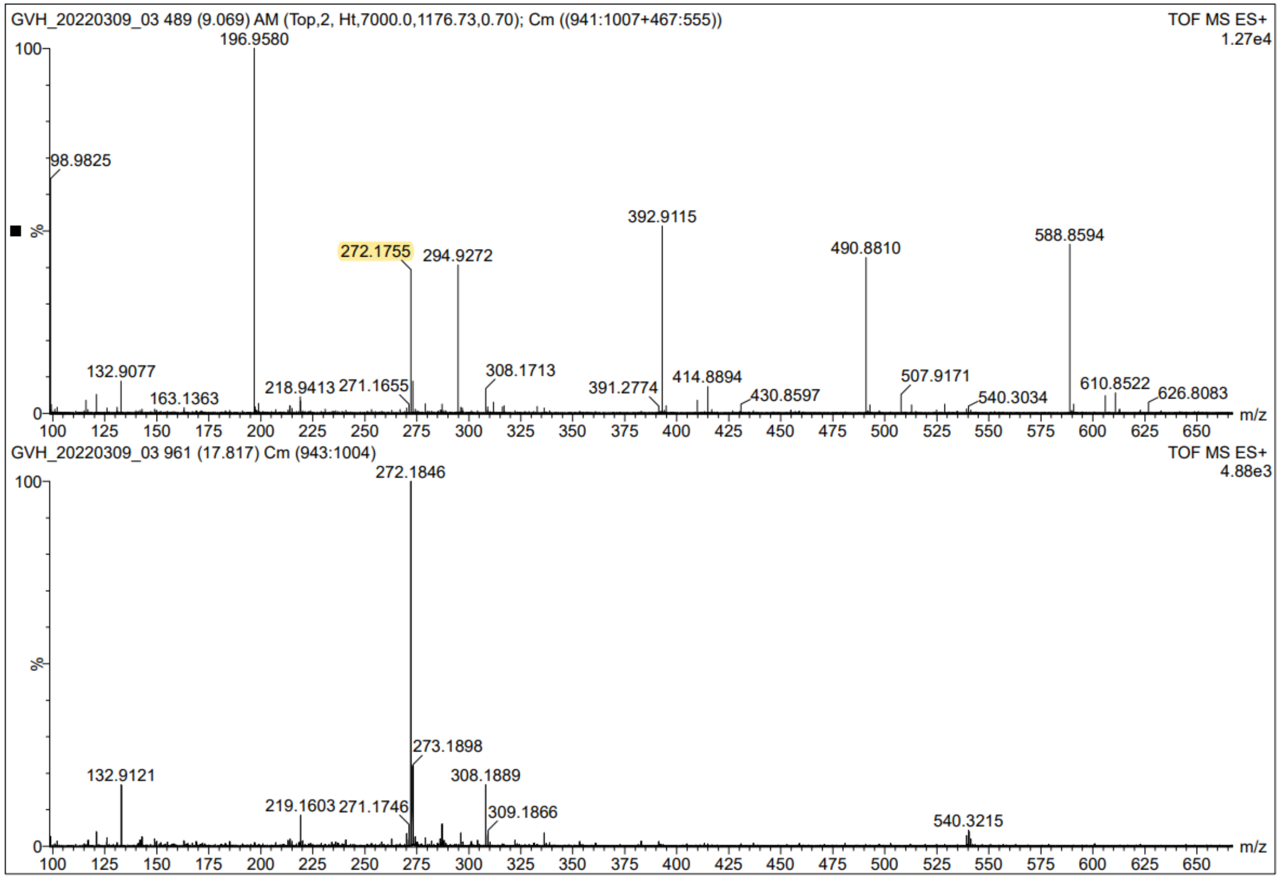

GVH_20220309_03 489 (9.069) AM (Top,2, Ht,7000.0,1176.73,0.70); Cm ((941:1007+467:555))
TOF MS ES+
1.27e4
196.9580
98.9825
392.9115
588.8594
490.8810
272.1755
294.9272
132.9077
308.1713
414.8894
507.9171
610.8522
626.8083
163.1363
218.9413
271.1655
391.2774
430.8597
540.3034
%
m/z
GVH_20220309_03 961 (17.817) Cm (943:1004)
TOF MS ES+
4.88e3
272.1846
273.1898
132.9121
308.1889
219.1603
271.1746
309.1866
540.3215
%
m/z

Compound 76

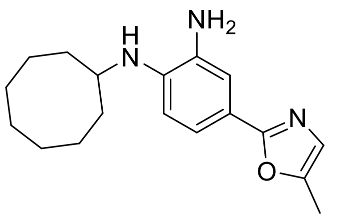

H
N
NH2
N
O

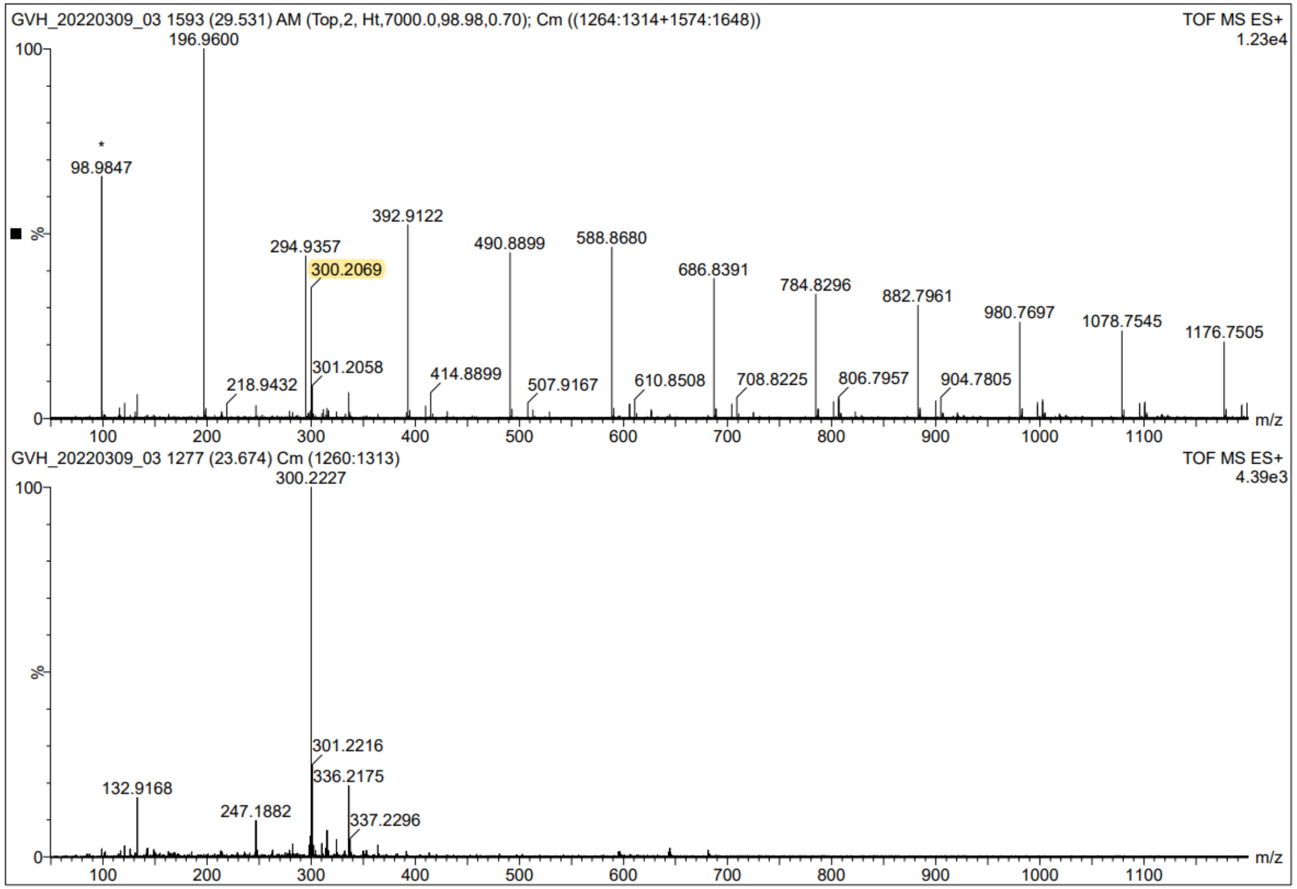

GVH_20220309_03 1593 (29.531) AM (Top,2, Ht,7000.0,98.98,0.70); Cm ((1264:1314+1574:1648))
TOF MS ES+
1.23e4
196.9600
98.9847
392.9122
294.9357
300.2069
490.8899
588.8680
686.8391
784.8296
882.7961
980.7697
1078.7545
1176.7505
218.9432
301.2058
414.8899
507.9167
610.8508
708.8225
806.7957
904.7805
%
m/z
GVH_20220309_03 1277 (23.674) Cm (1260:1313)
TOF MS ES+
4.39e3
300.2227
301.2216
336.2175
337.2296
132.9168
247.1882

Compound 77

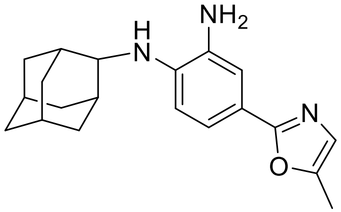

NH2
H
N
N
O

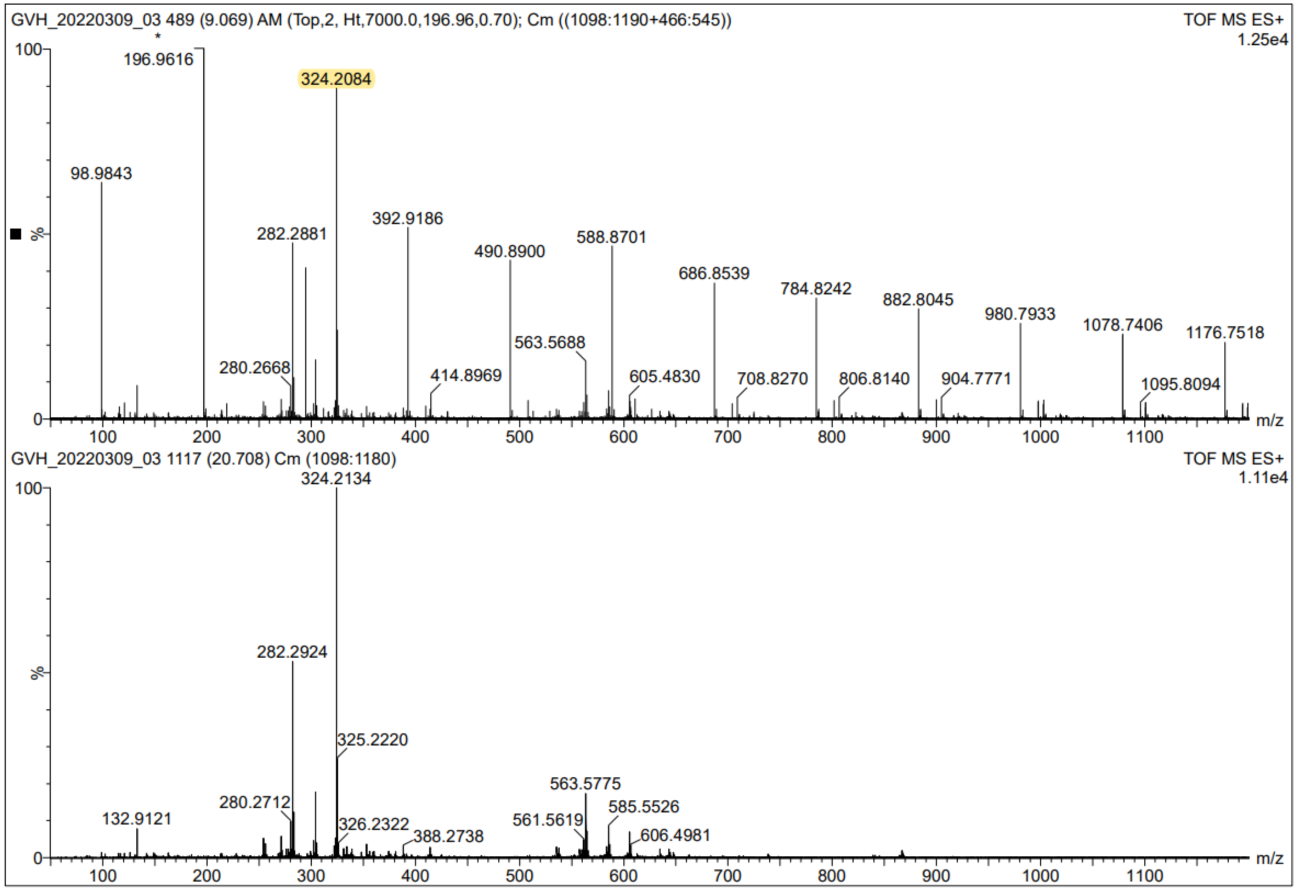

GVH_20220309_03 489 (9.069) AM (Top,2, Ht,7000.0,196.96,0.70); Cm ((1098:1190+466:545))
TOF MS ES+
1.25e4
196.9616
324.2084
98.9843
282.2881
392.9186
490.8900
588.8701
686.8539
784.8242
882.8045
980.7933
1078.7406
1176.7518
563.5688
280.2668
414.8969
605.4830
708.8270
806.8140
904.7771
1095.8094
%
m/z
GVH_20220309_03 1117 (20.708) Cm (1098:1180)
TOF MS ES+
1.11e4
324.2134
282.2924
325.2220
563.5775
585.5526
132.9121
280.2712
326.2322
561.5619
388.2738
606.4981

Compound 78

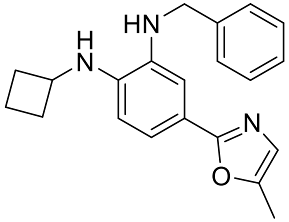
HN
H
N
N
O

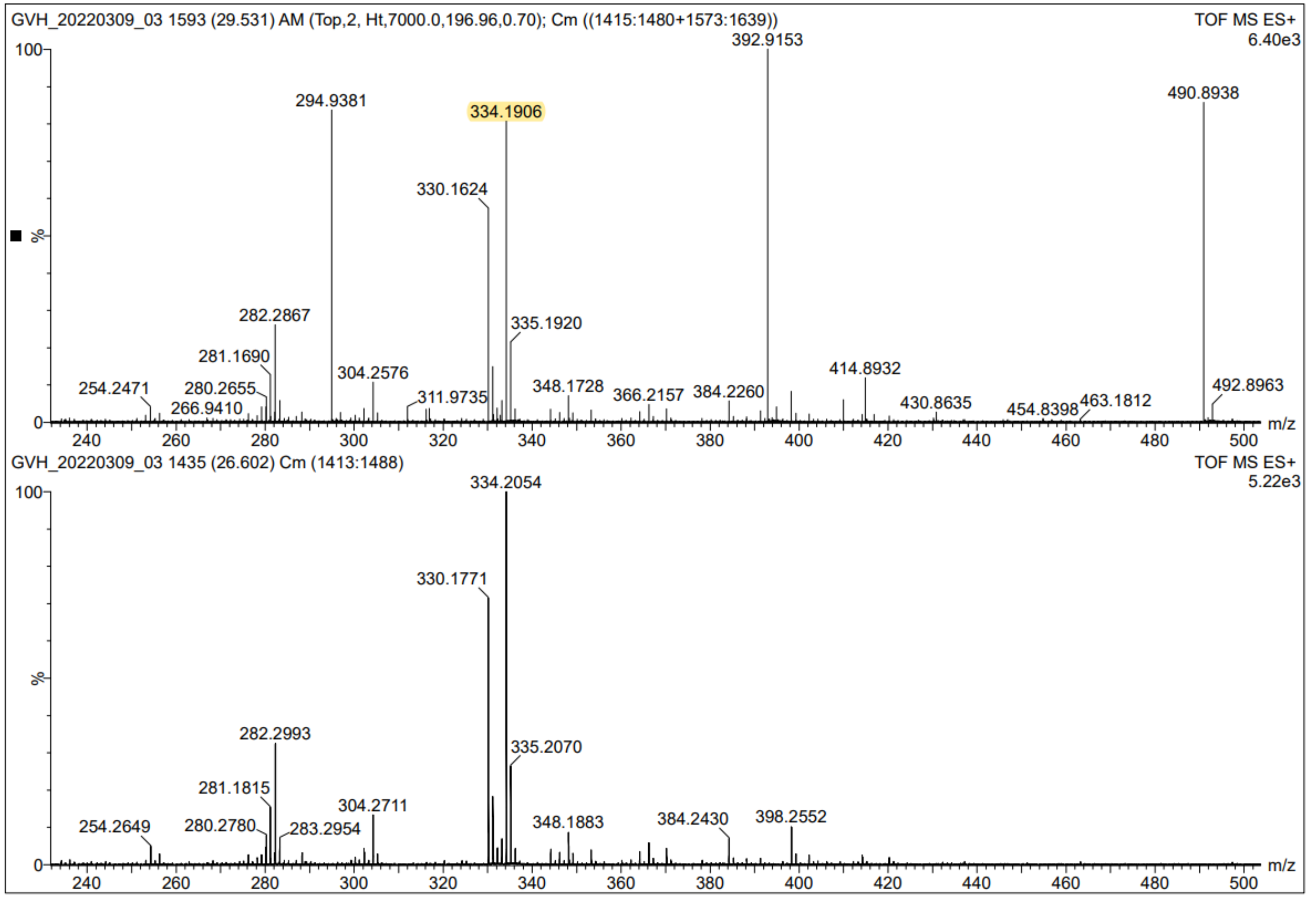
GVH_20220309_03 1593 (29.531) AM (Top,2, Ht,7000.0,196.96,0.70); Cm ((1415:1480+1573:1639))
TOF MS ES+
6.40e3
392.9153
294.9381
334.1906
490.8938
330.1624
282.2867
335.1920
281.1690
304.2576
254.2471
280.2655
266.9410
311.9735
348.1728
366.2157
384.2260
414.8932
430.8635
454.8398
463.1812
492.8963
%
m/z
GVH_20220309_03 1435 (26.602) Cm (1413:1488)
TOF MS ES+
5.22e3
334.2054
330.1771
282.2993
335.2070
281.1815
304.2711
254.2649
280.2780
283.2954
348.1883
384.2430
398.2552

Compound 79

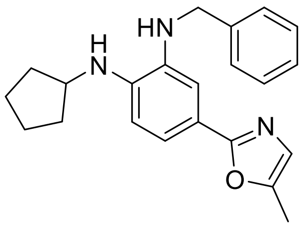
HN
H
N
N
O

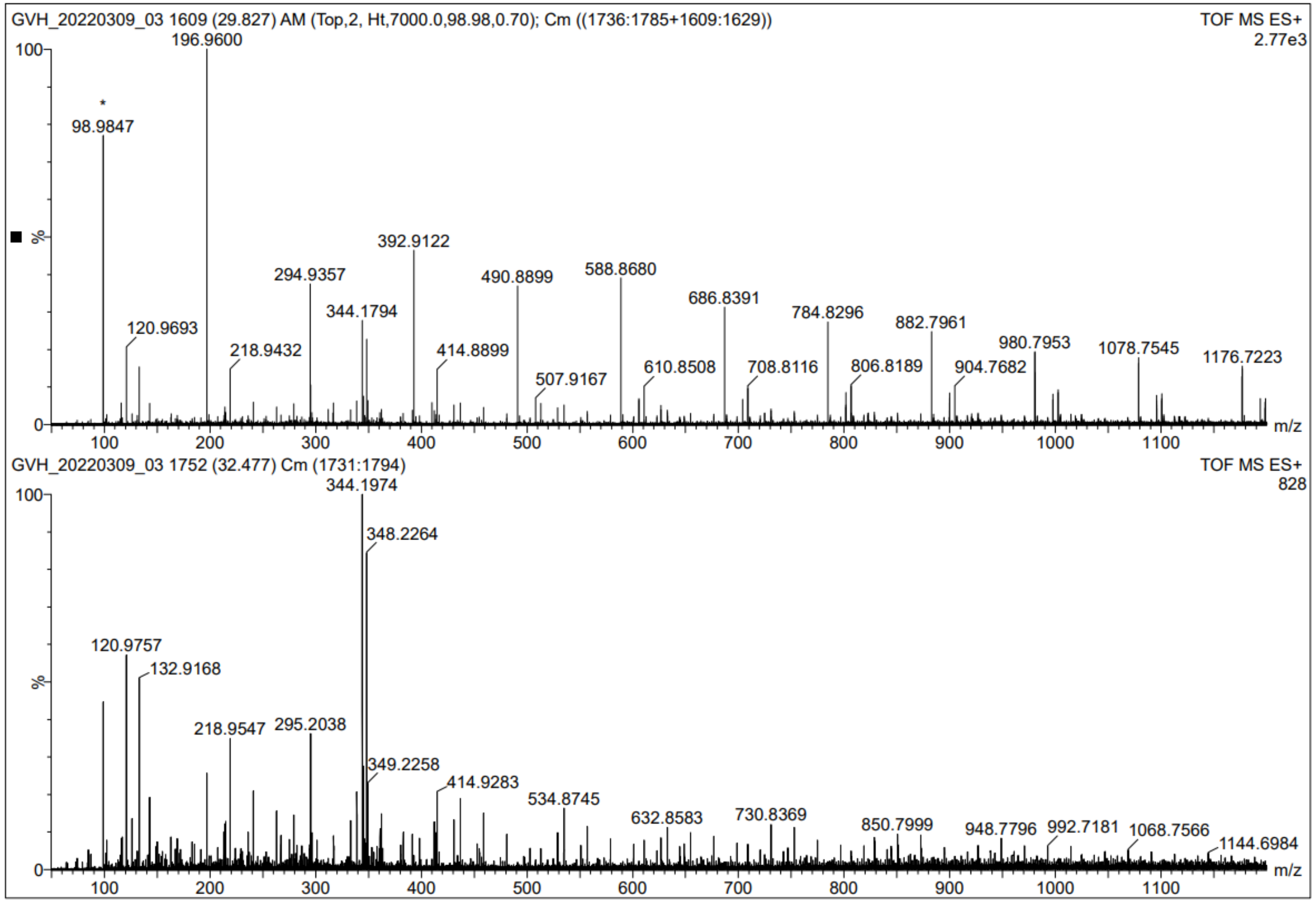
GVH_20220309_03 1609 (29.827) AM (Top,2, Ht,7000.0,98.98,0.70); Cm ((1736:1785+1609:1629))
TOF MS ES+
2.77e3
196.9600
98.9847
392.9122
294.9357
490.8899
588.8680
686.8391
784.8296
882.7961
980.7953
1078.7545
1176.7223
344.1794
120.9693
218.9432
414.8899
507.9167
610.8508
708.8116
806.8189
904.7682
%
m/z
GVH_20220309_03 1752 (32.477) Cm (1731:1794)
TOF MS ES+
828
344.1974
348.2264
120.9757
132.9168
218.9547
295.2038
349.2258
414.9283
534.8745
632.8583
730.8369
850.7999
948.7796
992.7181
1068.7566
1144.6984

Compound 80

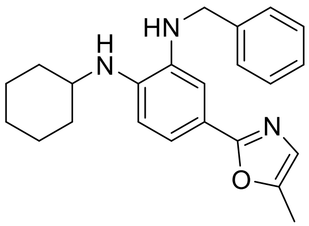
HN
H
N
N
O

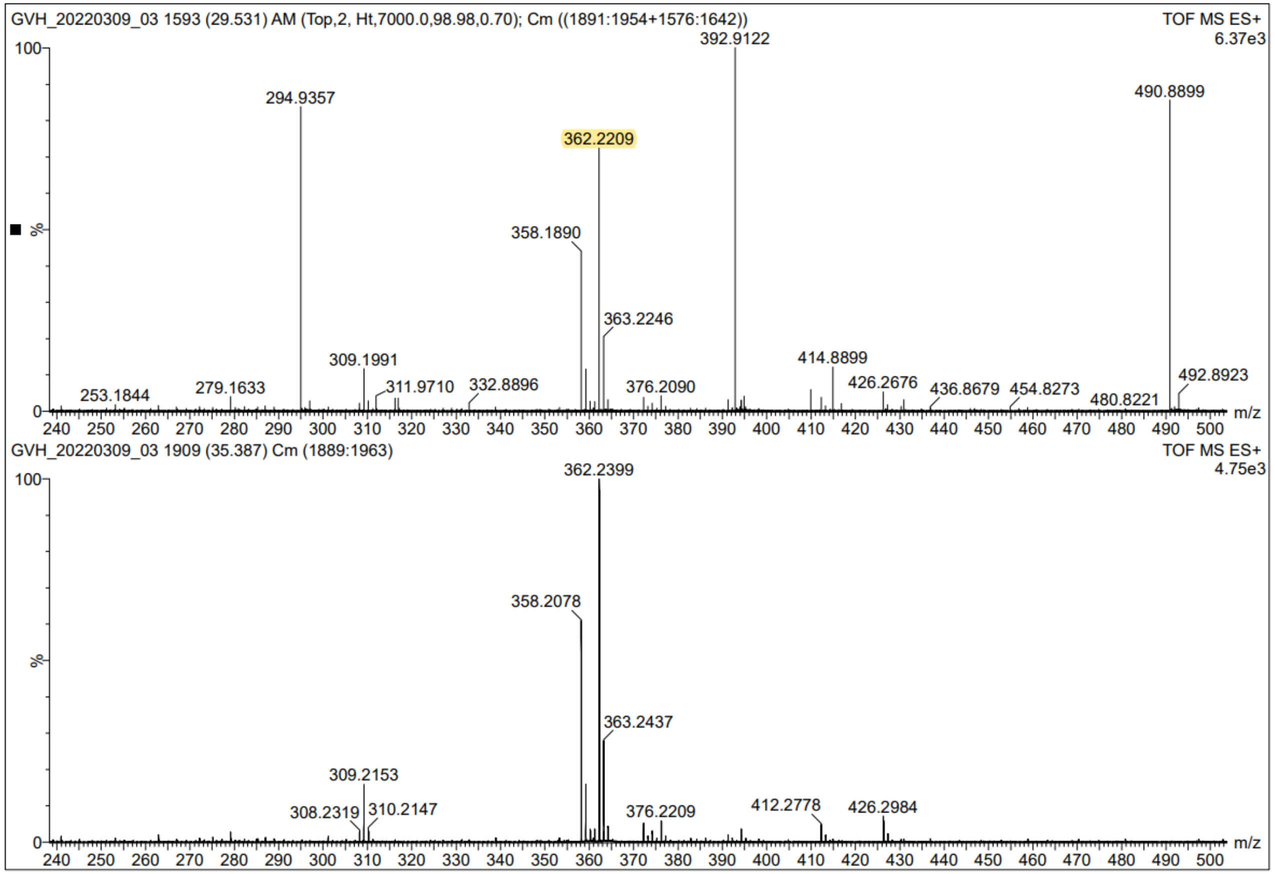
GVH_20220309_03 1593 (29.531) AM (Top,2, Ht,7000.0,98.98,0.70); Cm ((1891:1954+1576:1642))
TOF MS ES+
6.37e3
392.9122
294.9357
490.8899
362.2209
358.1890
363.2246
309.1991
414.8899
426.2676
492.8923
253.1844
279.1633
311.9710
332.8896
376.2090
436.8679
454.8273
480.8221
m/z
GVH_20220309_03 1909 (35.387) Cm (1889:1963)
TOF MS ES+
4.75e3
362.2399
358.2078
363.2437
309.2153
308.2319
310.2147
376.2209
412.2778
426.2984
m/z

Compound 81

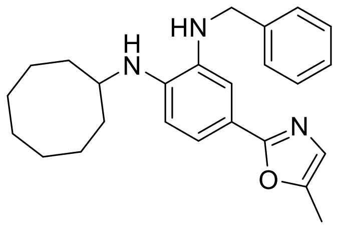

HN
H
N
N
O

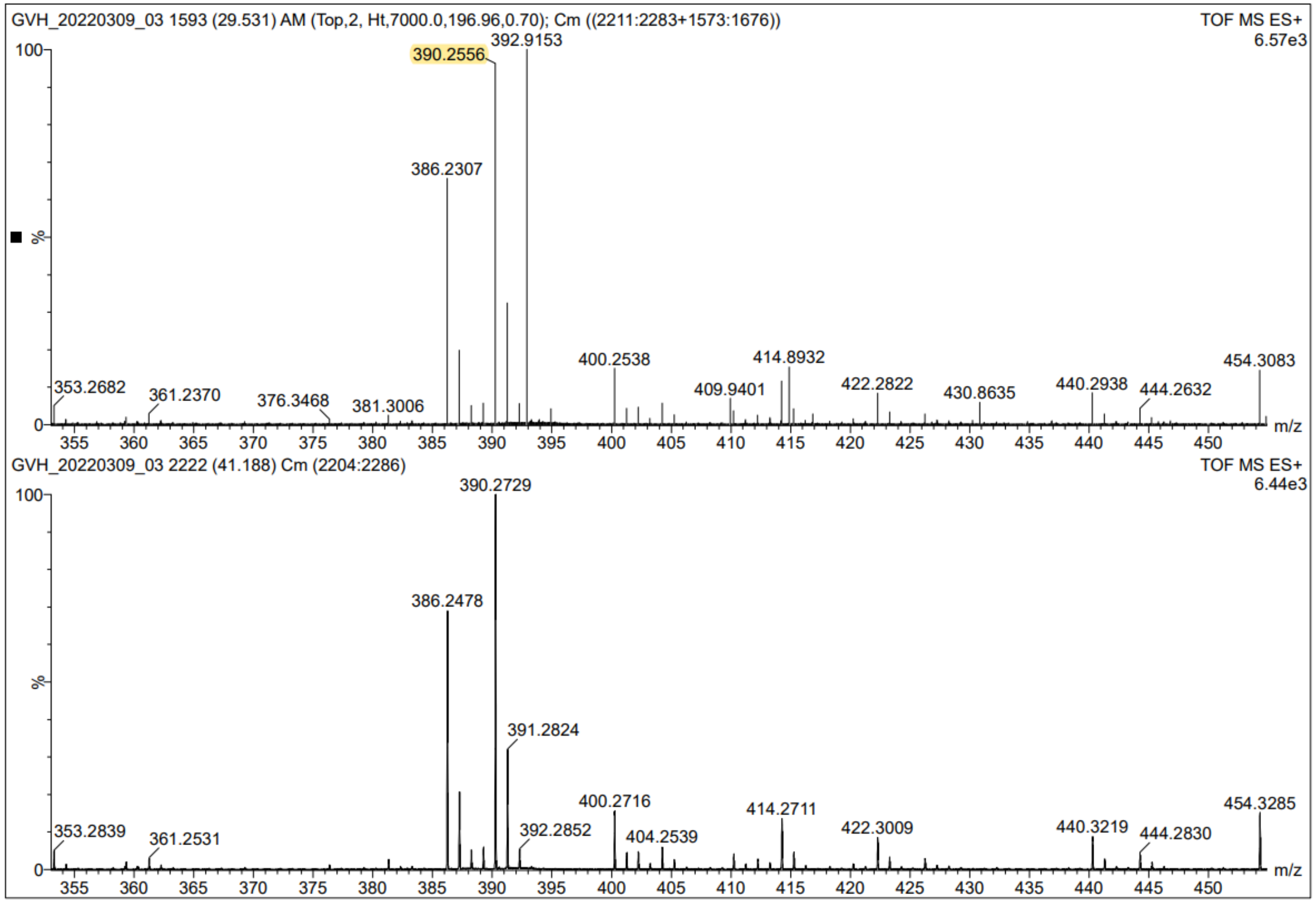

GVH_20220309_03 1593 (29.531) AM (Top,2, Ht,7000.0,196.96,0.70); Cm ((2211:2283+1573:1676))
TOF MS ES+
6.57e3
100
%
0
390.2556
392.9153
386.2307
353.2682
361.2370
376.3468
381.3006
400.2538
409.9401
414.8932
422.2822
430.8635
440.2938
444.2632
454.3083
m/z
355 360 365 370 375 380 385 390 395 400 405 410 415 420 425 430 435 440 445 450
GVH_20220309_03 2222 (41.188) Cm (2204:2286)
TOF MS ES+
6.44e3
100
%
0
390.2729
386.2478
391.2824
353.2839
361.2531
392.2852
400.2716
404.2539
414.2711
422.3009
440.3219
444.2830
454.3285
m/z
355 360 365 370 375 380 385 390 395 400 405 410 415 420 425 430 435 440 445 450

Compound 82

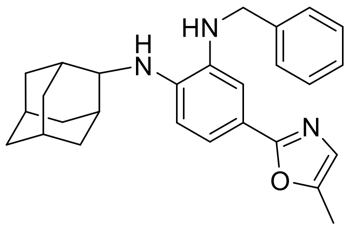

HN
H
N
N
O

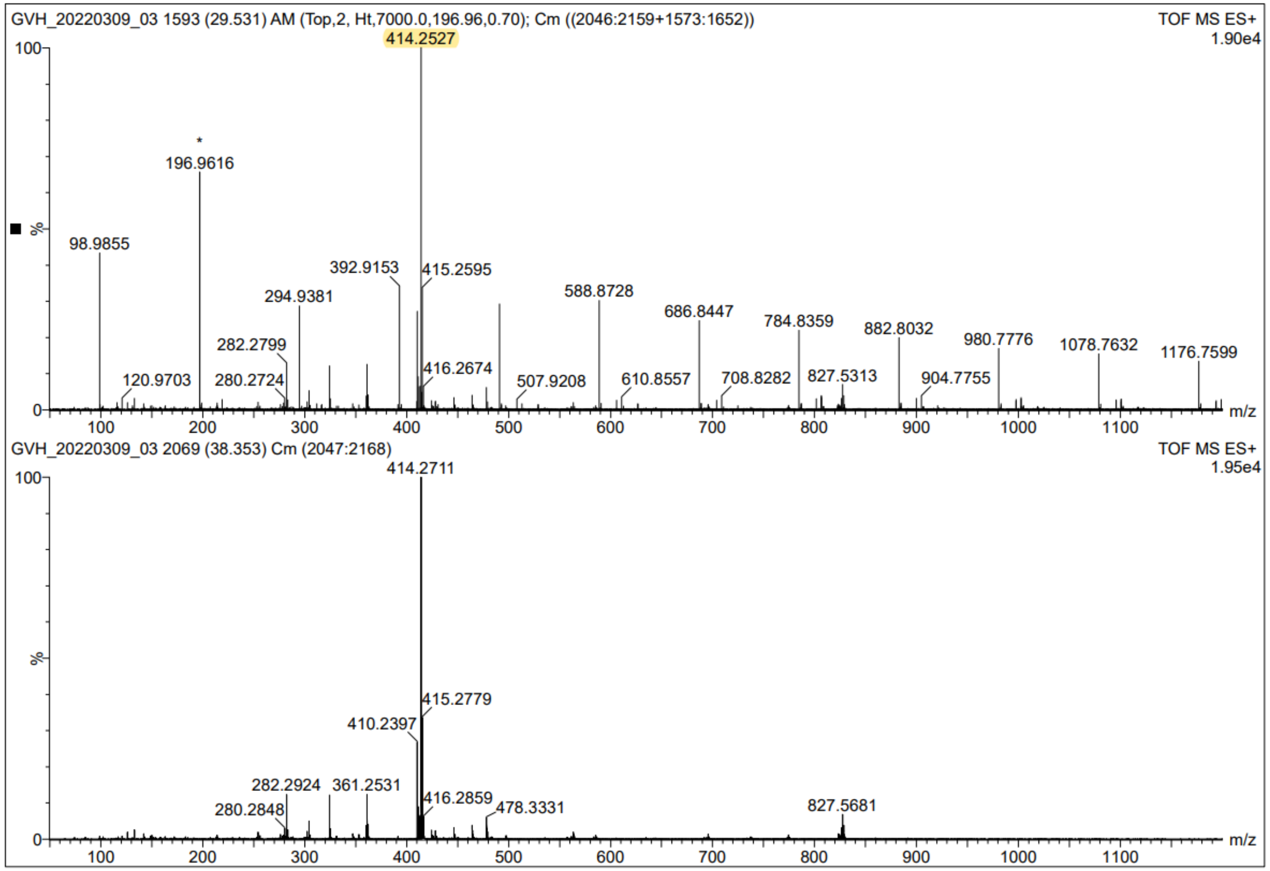

GVH_20220309_03 1593 (29.531) AM (Top,2, Ht,7000.0,196.96,0.70); Cm ((2046:2159+1573:1652))
TOF MS ES+
1.90e4
414.2527
196.9616
98.9855
392.9153
415.2595
294.9381
588.8728
686.8447
784.8359
882.8032
980.7776
1078.7632
1176.7599
282.2799
416.2674
120.9703
280.2724
507.9208
610.8557
708.8282
827.5313
904.7755
%
m/z
GVH_20220309_03 2069 (38.353) Cm (2047:2168)
TOF MS ES+
1.95e4
414.2711
415.2779
410.2397
282.2924
361.2531
280.2848
416.2859
478.3331
827.5681

Compound 83

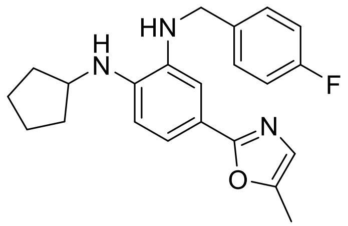

HN
H
N
N
O
F

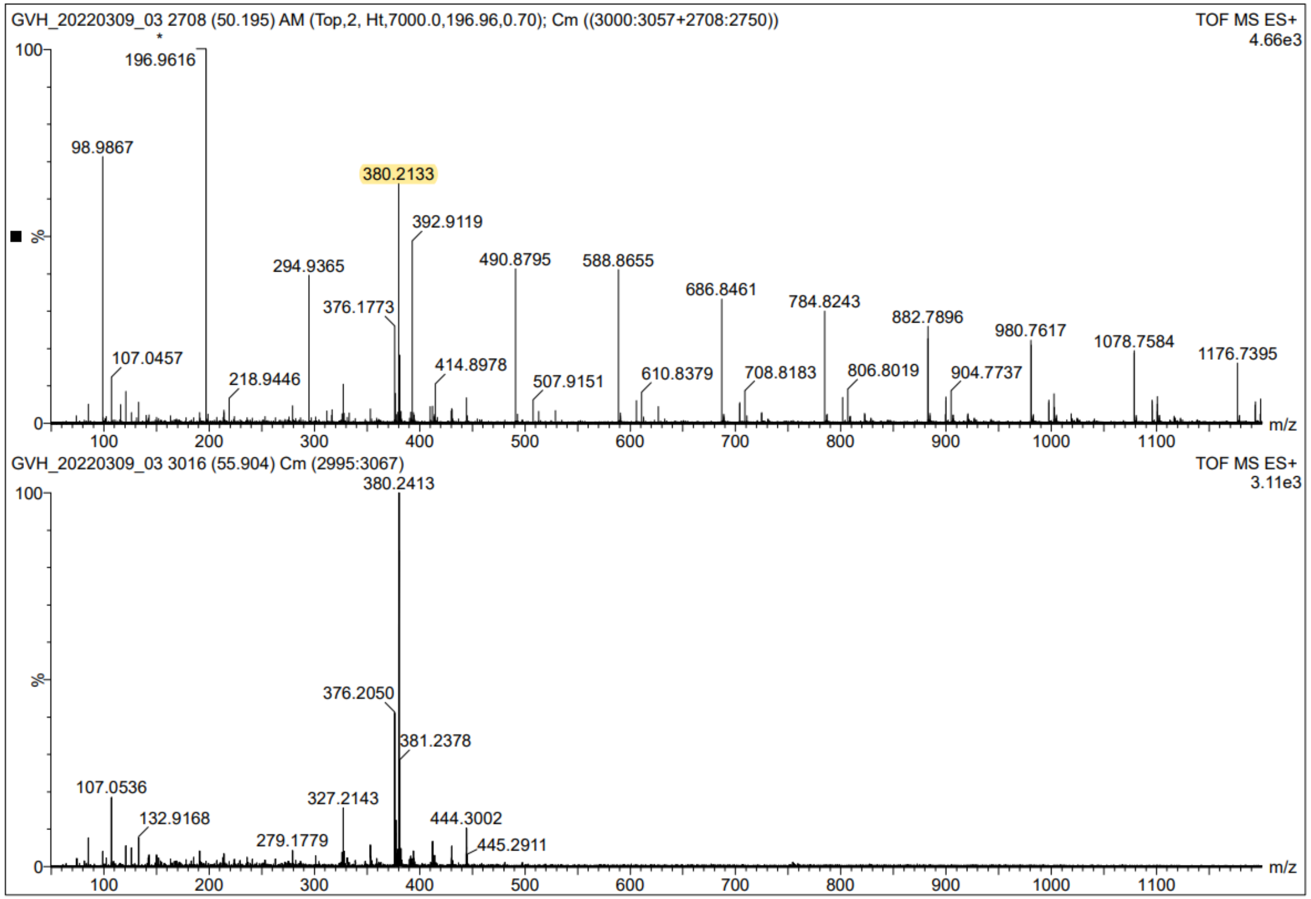

GVH_20220309_03 2708 (50.195) AM (Top,2, Ht,7000.0,196.96,0.70); Cm ((3000:3057+2708:2750))
TOF MS ES+
4.66e3
100
%
0
196.9616
98.9867
380.2133
392.9119
294.9365
490.8795
588.8655
686.8461
784.8243
376.1773
882.7896
980.7617
1078.7584
1176.7395
107.0457
414.8978
218.9446
507.9151
610.8379
708.8183
806.8019
904.7737
m/z
100 200 300 400 500 600 700 800 900 1000 1100
GVH_20220309_03 3016 (55.904) Cm (2995:3067)
TOF MS ES+
3.11e3
380.2413
376.2050
381.2378
107.0536
132.9168
327.2143
444.3002
279.1779
445.2911

Compound 84

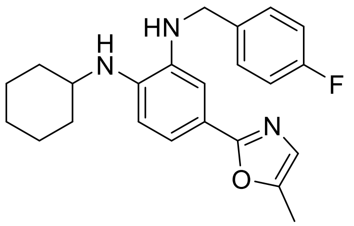

HN
H
N
F
N
O

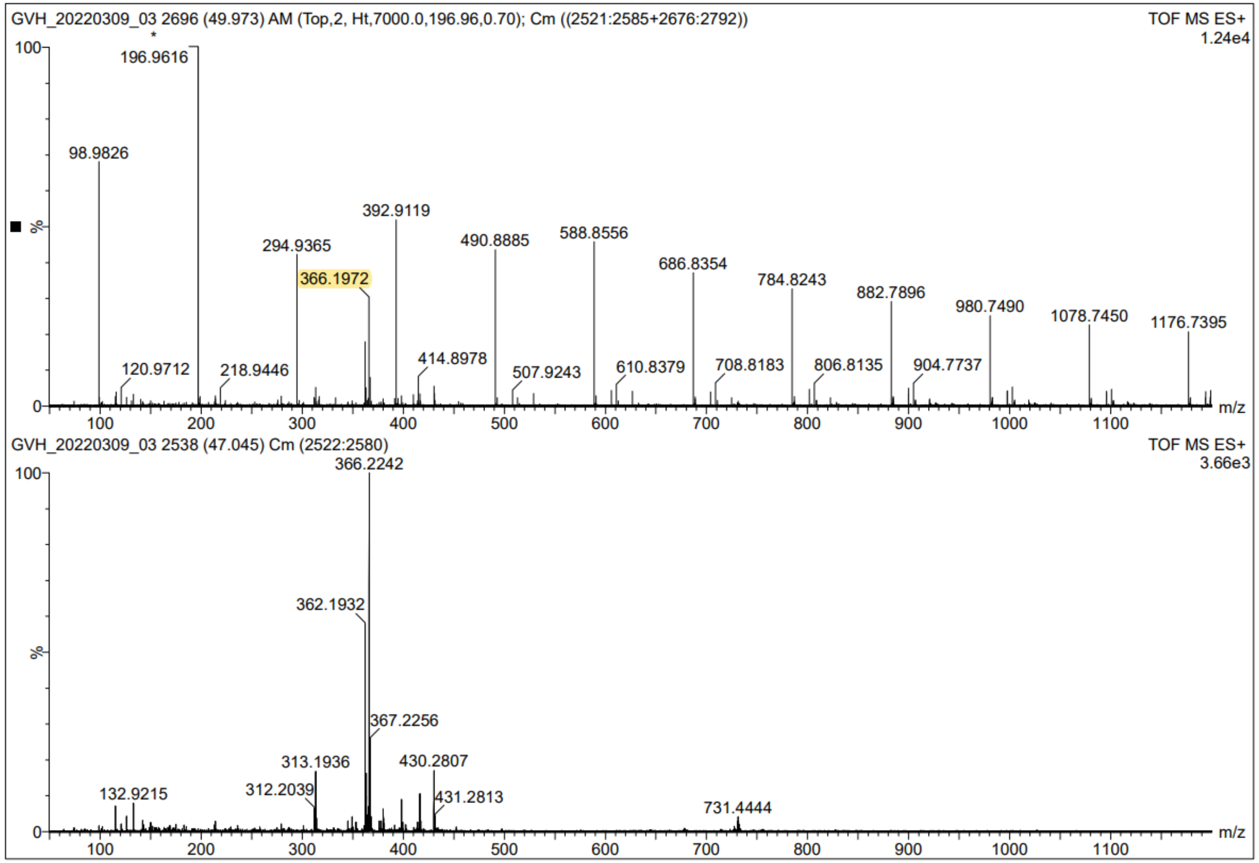

GVH_20220309_03 2696 (49.973) AM (Top,2, Ht,7000.0,196.96,0.70); Cm ((2521:2585+2676:2792))
TOF MS ES+
1.24e4
196.9616
98.9826
392.9119
294.9365
490.8885
588.8556
366.1972
686.8354
784.8243
882.7896
980.7490
1078.7450
1176.7395
120.9712
218.9446
414.8978
507.9243
610.8379
708.8183
806.8135
904.7737
%
m/z
GVH_20220309_03 2538 (47.045) Cm (2522:2580)
TOF MS ES+
3.66e3
366.2242
362.1932
367.2256
313.1936
430.2807
132.9215
312.2039
431.2813
731.4444

Compound 85

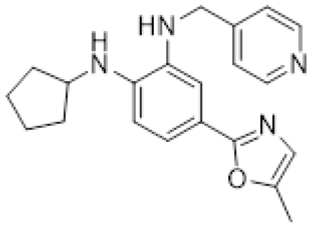
HN
H
N
N
N
O

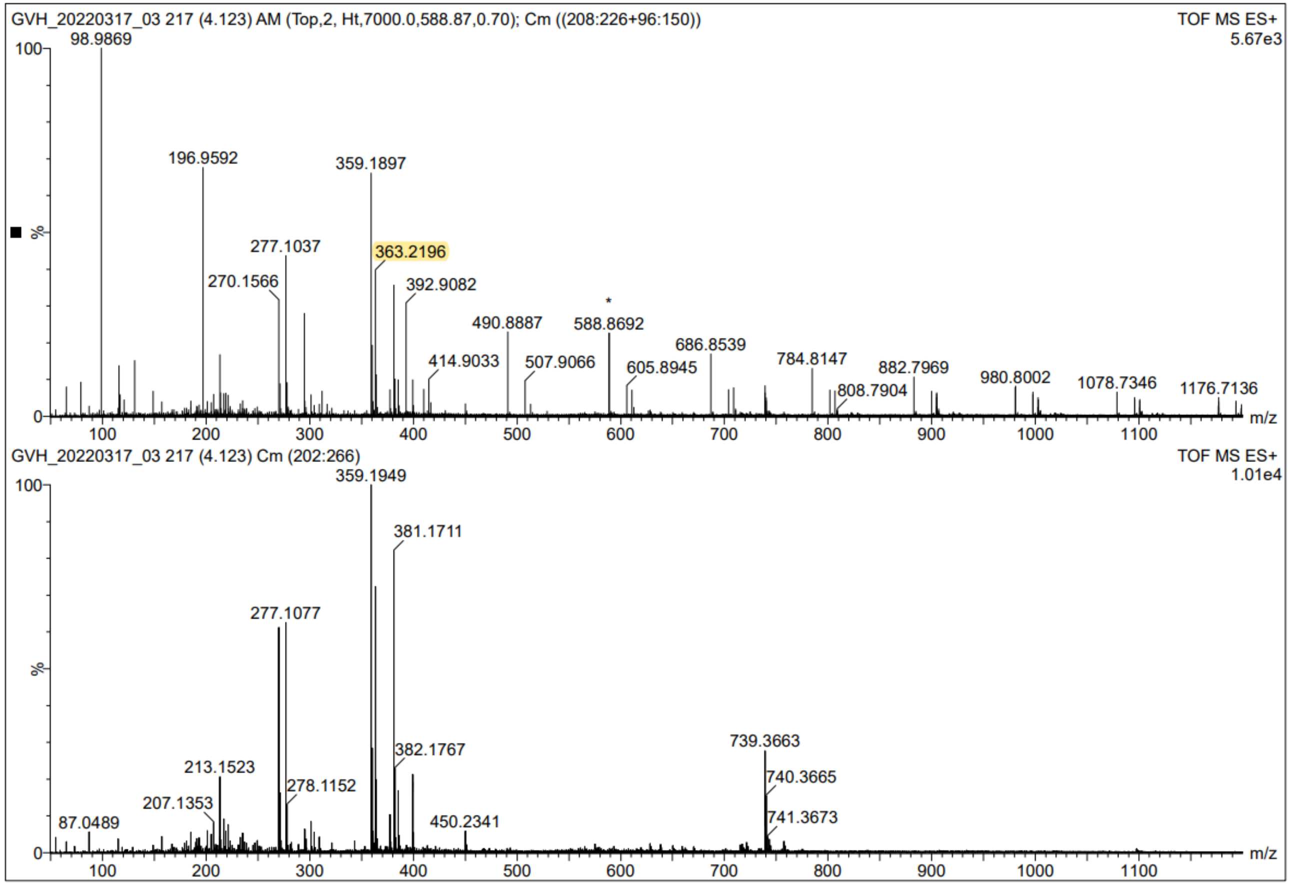
GVH_20220317_03 217 (4.123) AM (Top,2, Ht,7000.0,588.87,0.70); Cm ((208:226+96:150))
TOF MS ES+
5.67e3
98.9869
196.9592
359.1897
277.1037
363.2196
270.1566
392.9082
490.8887
588.8692
686.8539
784.8147
882.7969
980.8002
1078.7346
1176.7136
414.9033
507.9066
605.8945
808.7904
%
m/z
GVH_20220317_03 217 (4.123) Cm (202:266)
TOF MS ES+
1.01e4
359.1949
381.1711
277.1077
382.1767
739.3663
740.3665
741.3673
213.1523
278.1152
207.1353
87.0489
450.2341

Compound 86

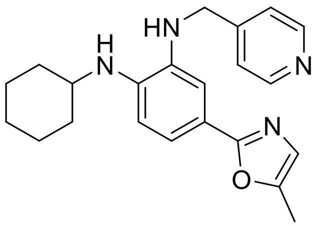

HN
H
N
N
N
O

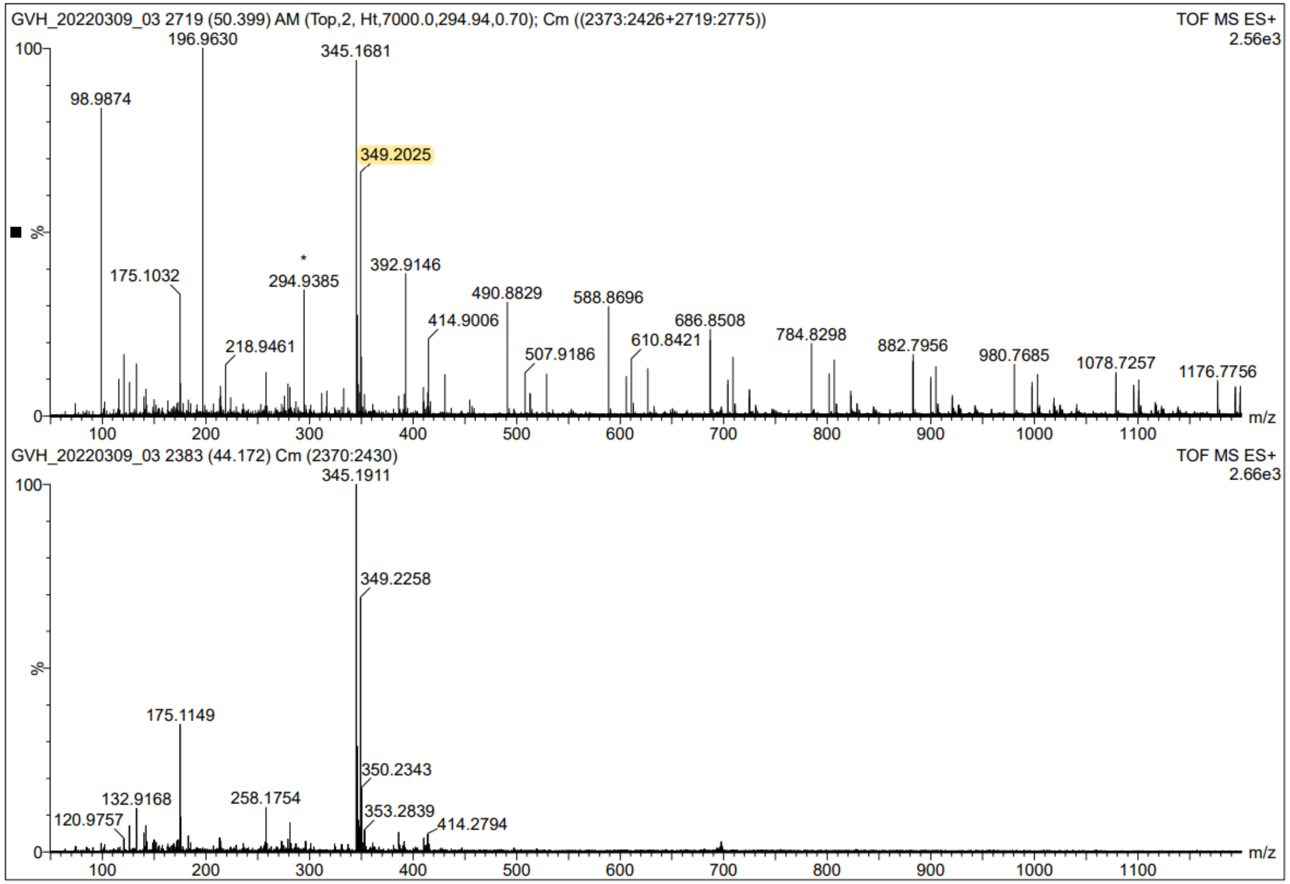

GVH_20220309_03 2719 (50.399) AM (Top,2, Ht,7000.0,294.94,0.70); Cm ((2373:2426+2719:2775))
TOF MS ES+
2.56e3
196.9630
345.1681
98.9874
349.2025
175.1032
294.9385
392.9146
490.8829
588.8696
414.9006
686.8508
218.9461
610.8421
784.8298
507.9186
882.7956
980.7685
1078.7257
1176.7756
m/z
GVH_20220309_03 2383 (44.172) Cm (2370:2430)
TOF MS ES+
2.66e3
345.1911
349.2258
175.1149
350.2343
132.9168
258.1754
120.9757
353.2839
414.2794
m/z

Compound 94 (UAMC-4749)

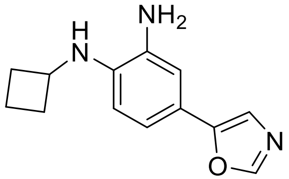

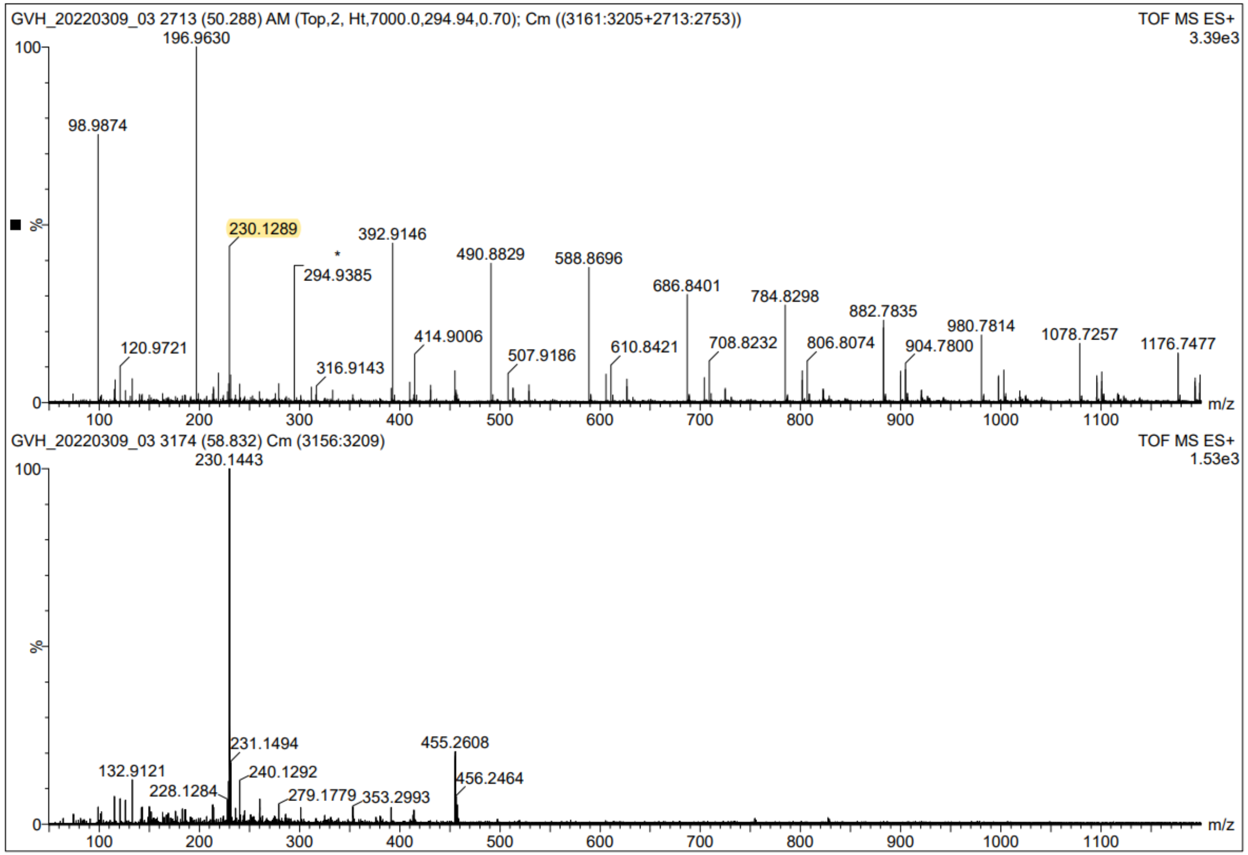

Compound 95

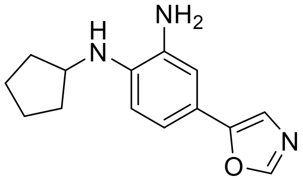

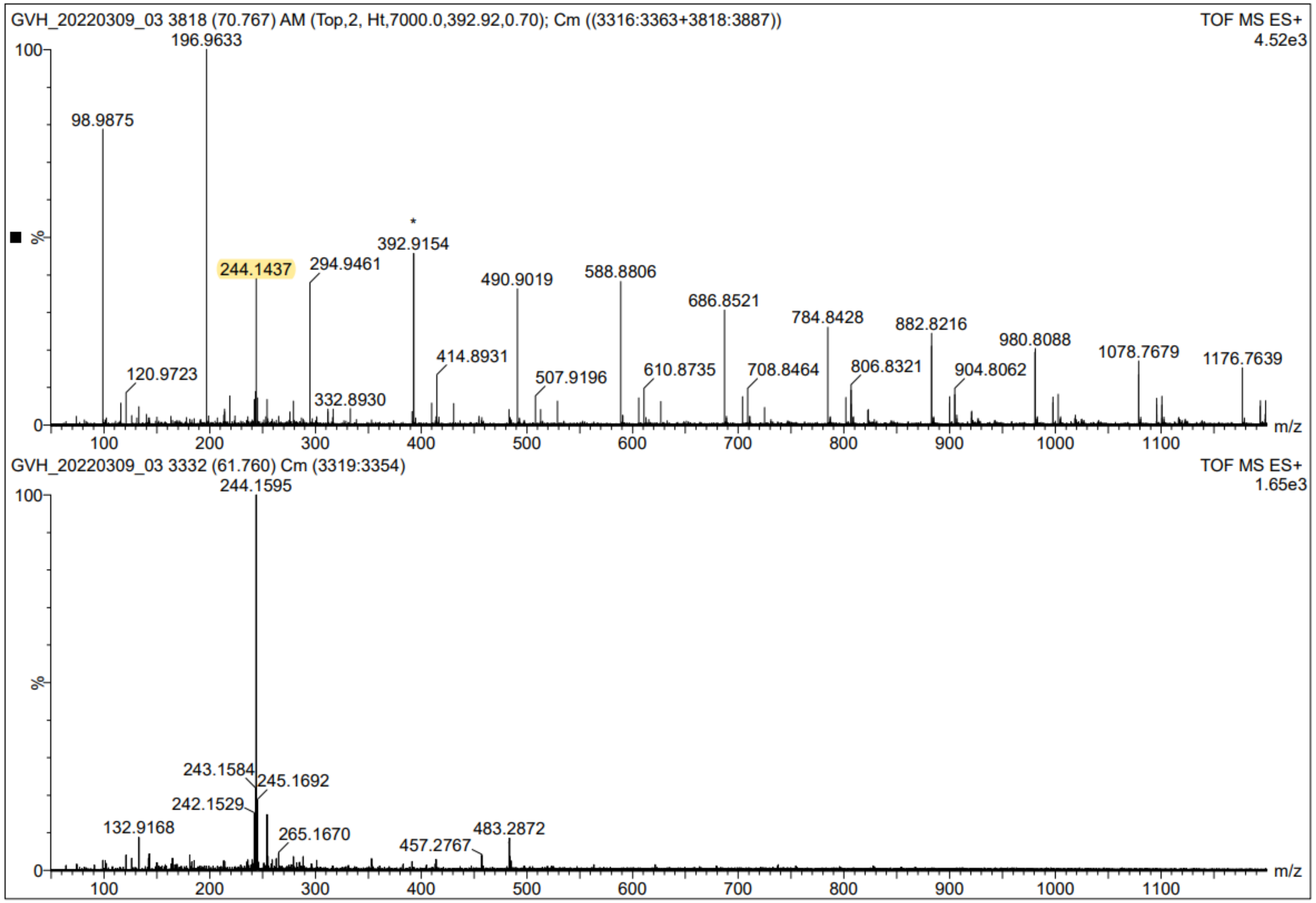
GVH_20220309_03 3818 (70.767) AM (Top,2, Ht,7000.0,392.92,0.70); Cm ((3316:3363+3818:3887))
TOF MS ES+
4.52e3
196.9633
98.9875
392.9154
244.1437
294.9461
490.9019
588.8806
686.8521
784.8428
882.8216
980.8088
1078.7679
1176.7639
414.8931
507.9196
610.8735
708.8464
806.8321
904.8062
120.9723
332.8930
%
m/z
GVH_20220309_03 3332 (61.760) Cm (3319:3354)
TOF MS ES+
1.65e3
244.1595
243.1584
245.1692
242.1529
132.9168
265.1670
457.2767
483.2872

Compound 96 (UAMC-4821)

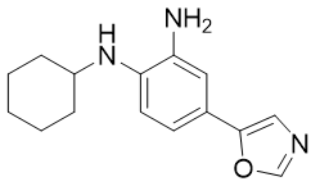

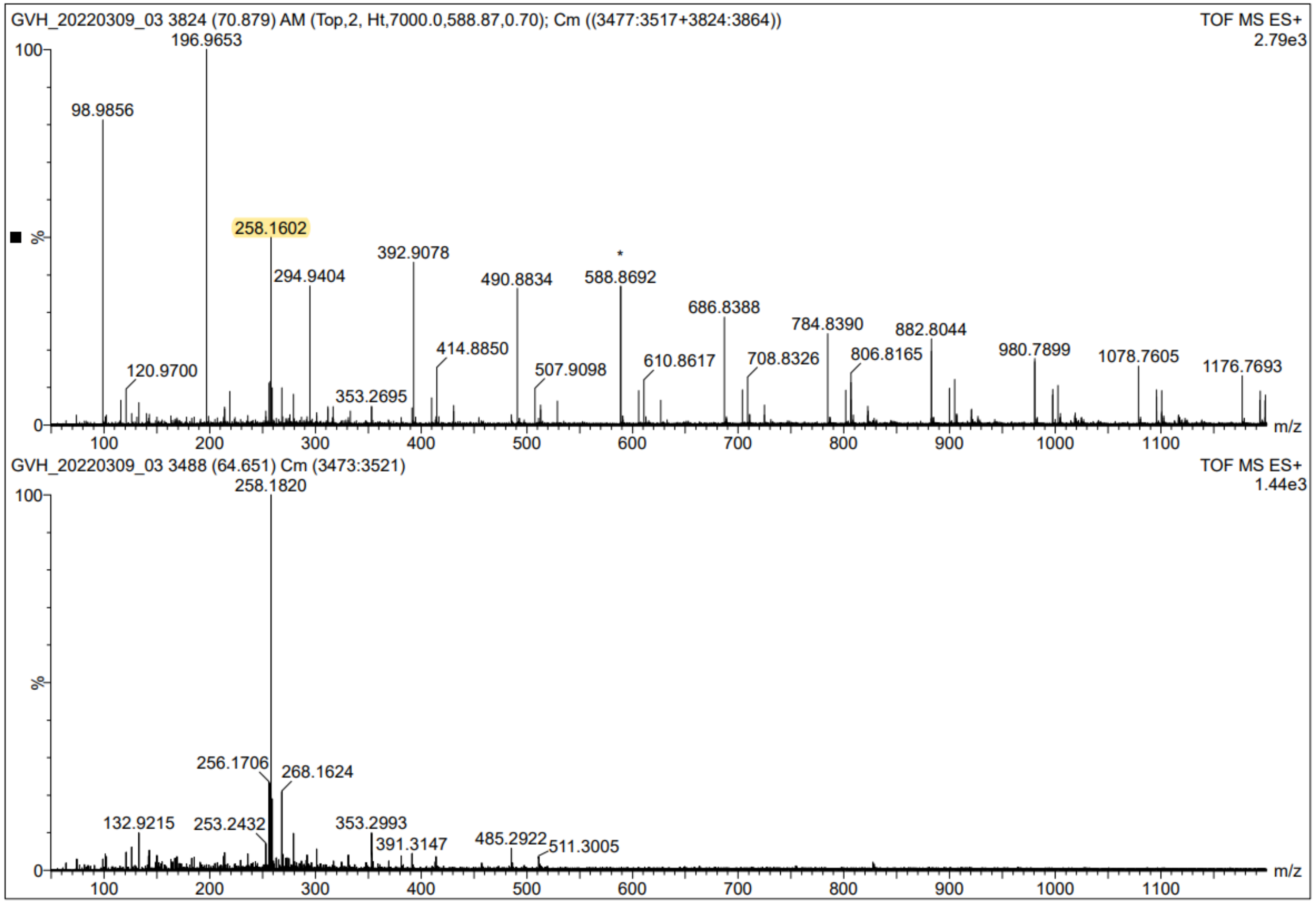

Compound 97

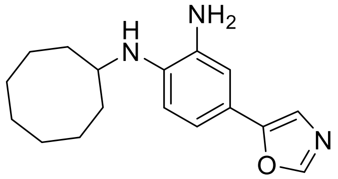
NH2
H
N
N
O

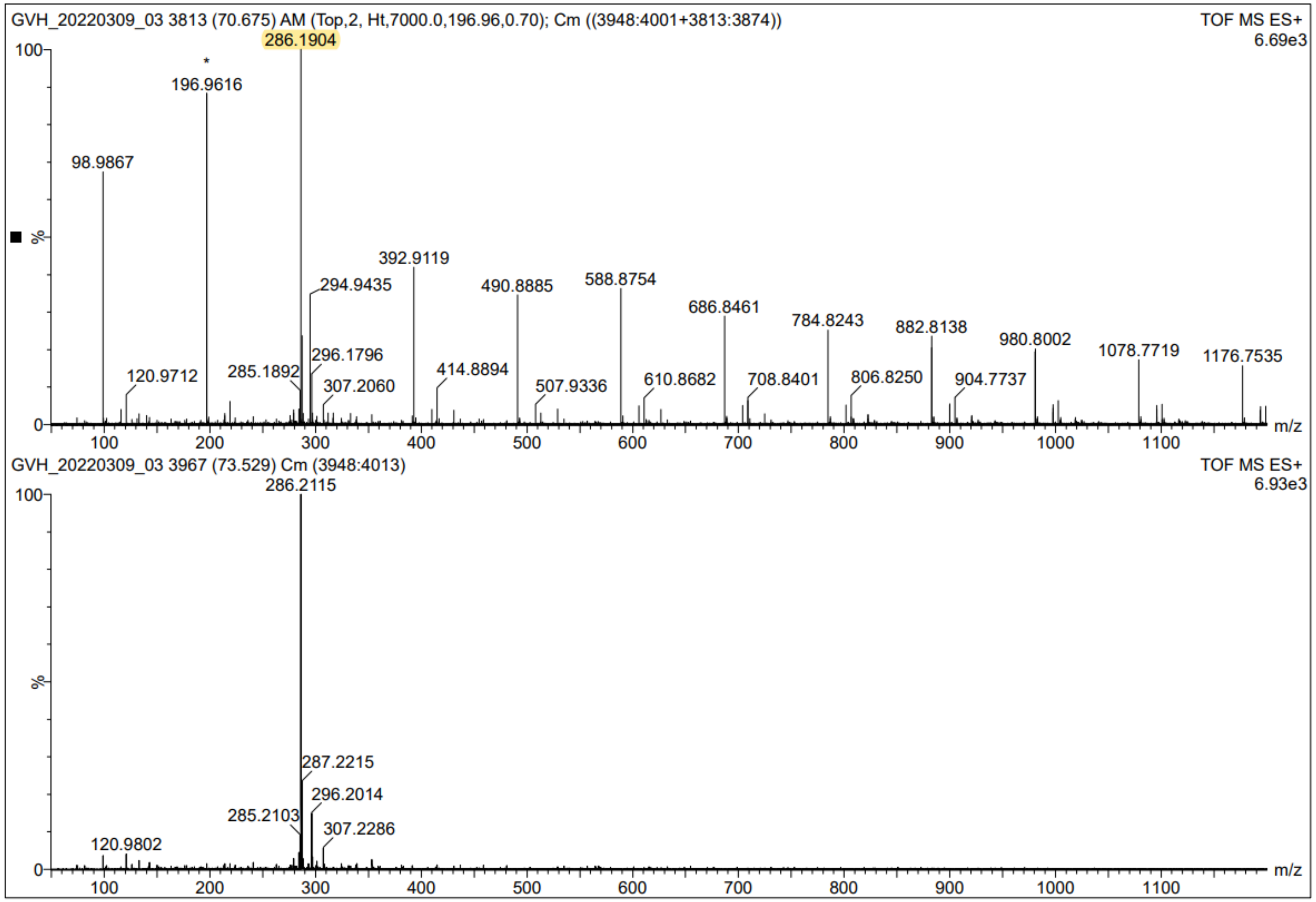
GVH_20220309_03 3813 (70.675) AM (Top,2, Ht,7000.0,196.96,0.70); Cm ((3948:4001+3813:3874))
TOF MS ES+
6.69e3
286.1904
196.9616
98.9867
392.9119
294.9435
490.8885
588.8754
686.8461
784.8243
882.8138
980.8002
1078.7719
1176.7535
296.1796
120.9712
285.1892
414.8894
307.2060
507.9336
610.8682
708.8401
806.8250
904.7737
%
m/z
GVH_20220309_03 3967 (73.529) Cm (3948:4013)
TOF MS ES+
6.93e3
286.2115
287.2215
296.2014
285.2103
307.2286
120.9802

Compound 98

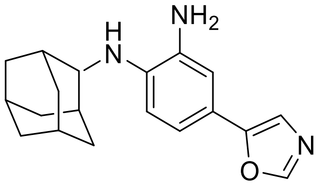
NH2
H
N
N
O

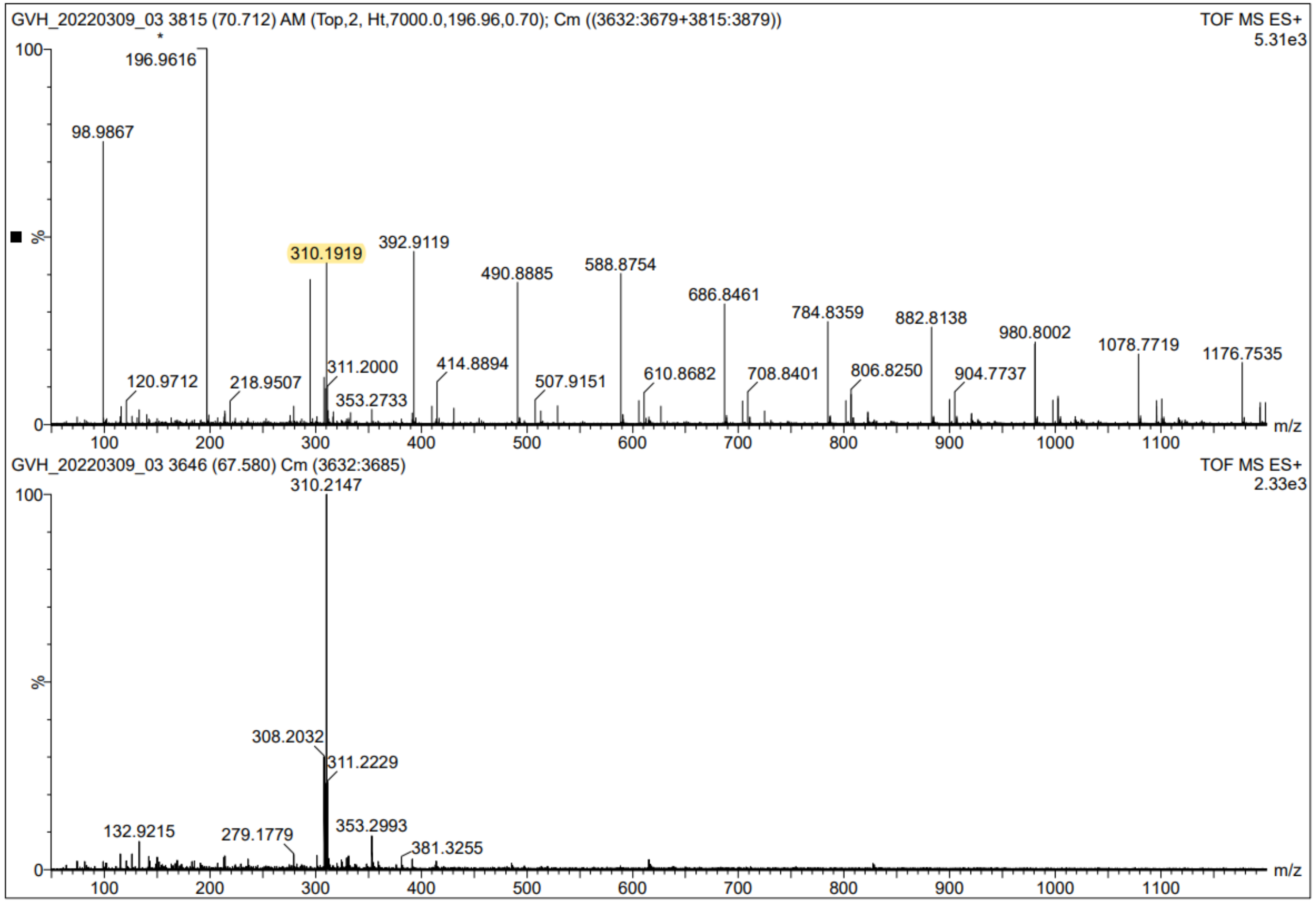
GVH_20220309_03 3815 (70.712) AM (Top,2, Ht,7000.0,196.96,0.70); Cm ((3632:3679+3815:3879))
TOF MS ES+
5.31e3
196.9616
98.9867
310.1919
392.9119
490.8885
588.8754
686.8461
784.8359
882.8138
980.8002
1078.7719
1176.7535
120.9712
218.9507
311.2000
353.2733
414.8894
507.9151
610.8682
708.8401
806.8250
904.7737
m/z
GVH_20220309_03 3646 (67.580) Cm (3632:3685)
TOF MS ES+
2.33e3
310.2147
308.2032
311.2229
132.9215
279.1779
353.2993
381.3255
m/z

Compound 99

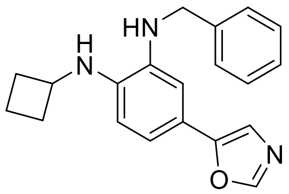
HN
H
N
N
O

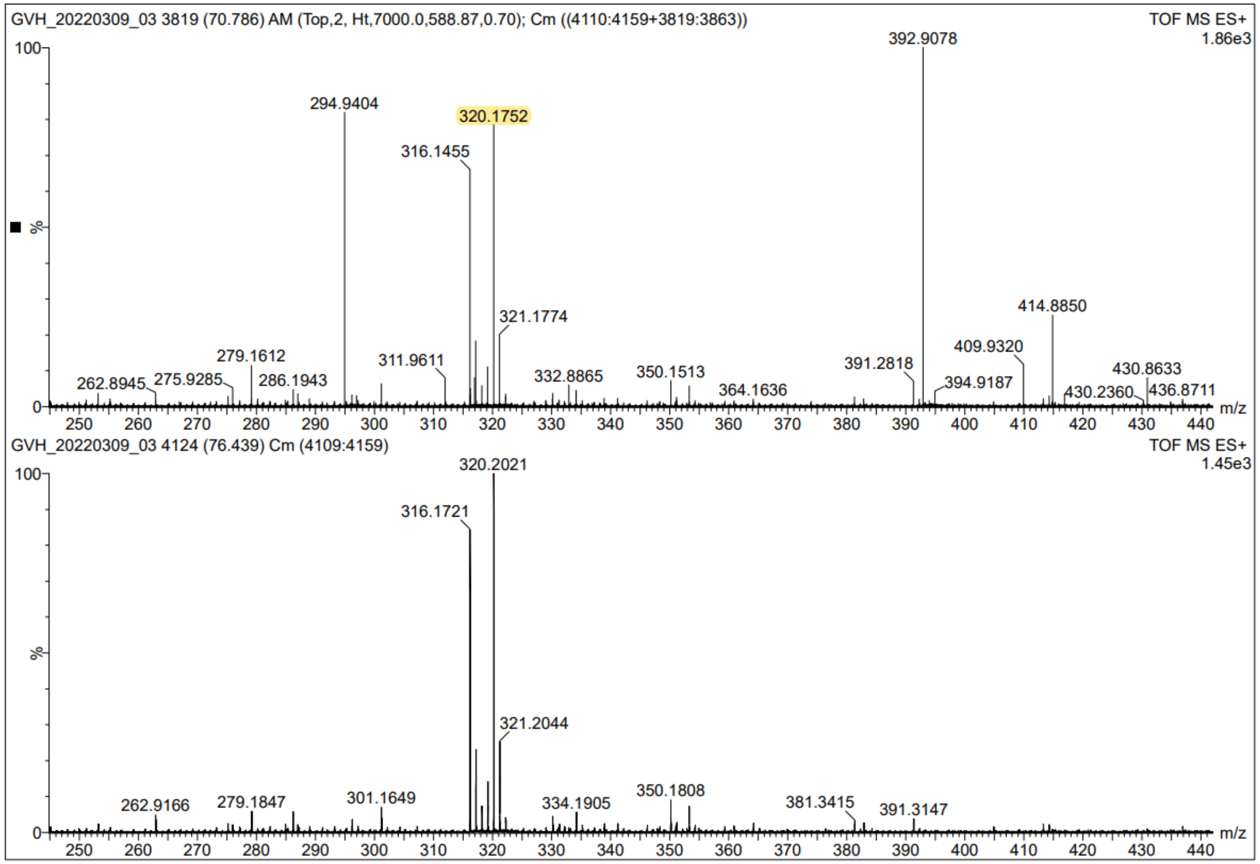
GVH_20220309_03 3819 (70.786) AM (Top,2, Ht,7000.0,588.87,0.70); Cm ((4110:4159+3819:3863))
TOF MS ES+
1.86e3
392.9078
294.9404
320.1752
316.1455
321.1774
414.8850
409.9320
279.1612
311.9611
391.2818
430.8633
262.8945
275.9285
286.1943
332.8865
350.1513
364.1636
394.9187
430.2360
436.8711
%
m/z
GVH_20220309_03 4124 (76.439) Cm (4109:4159)
TOF MS ES+
1.45e3
320.2021
316.1721
321.2044
262.9166
279.1847
301.1649
334.1905
350.1808
381.3415
391.3147

Compound 100

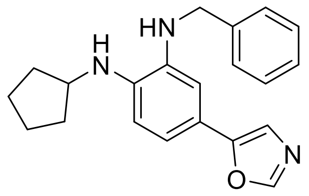

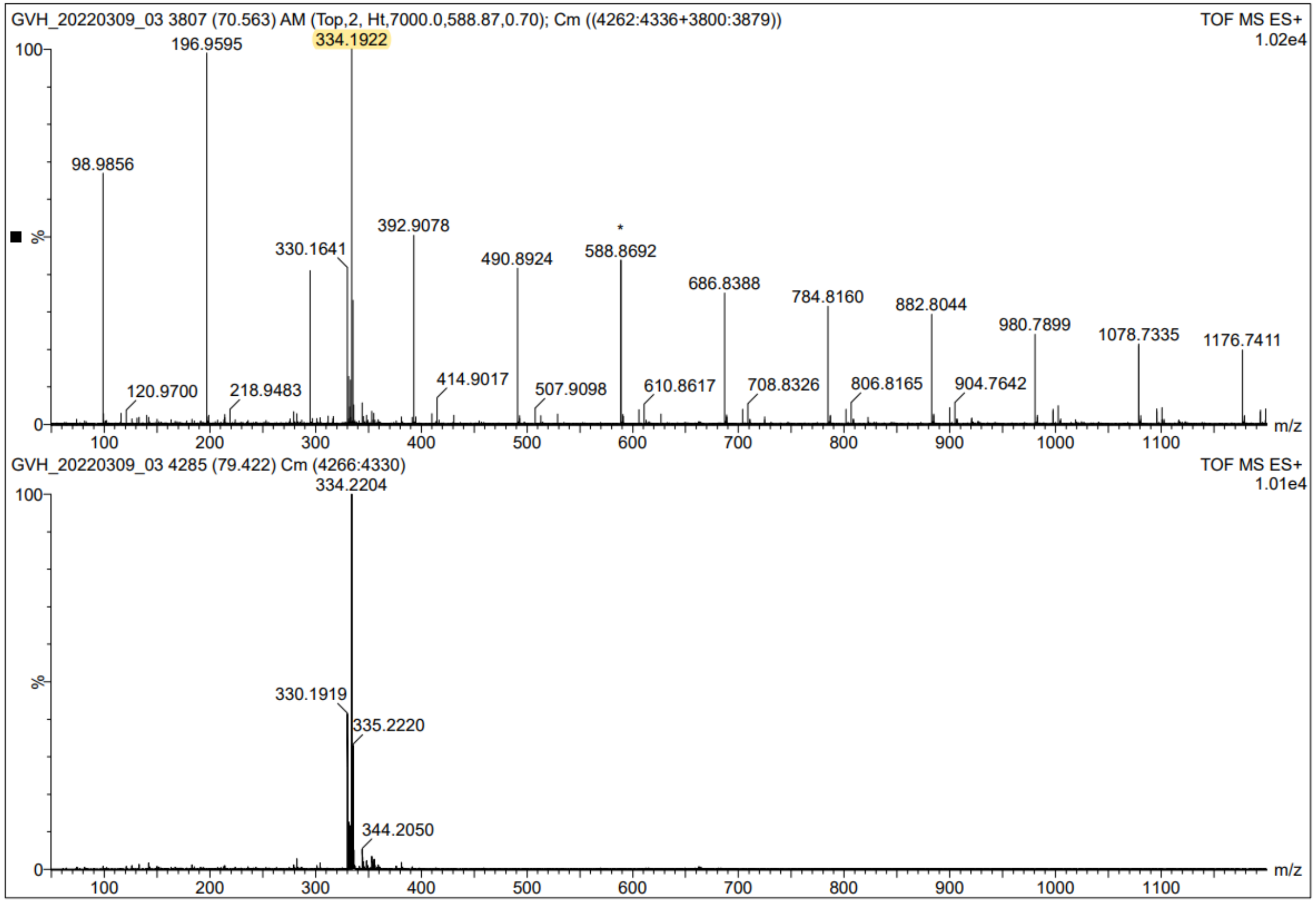

Compound 101

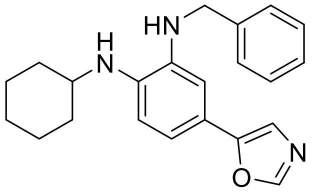

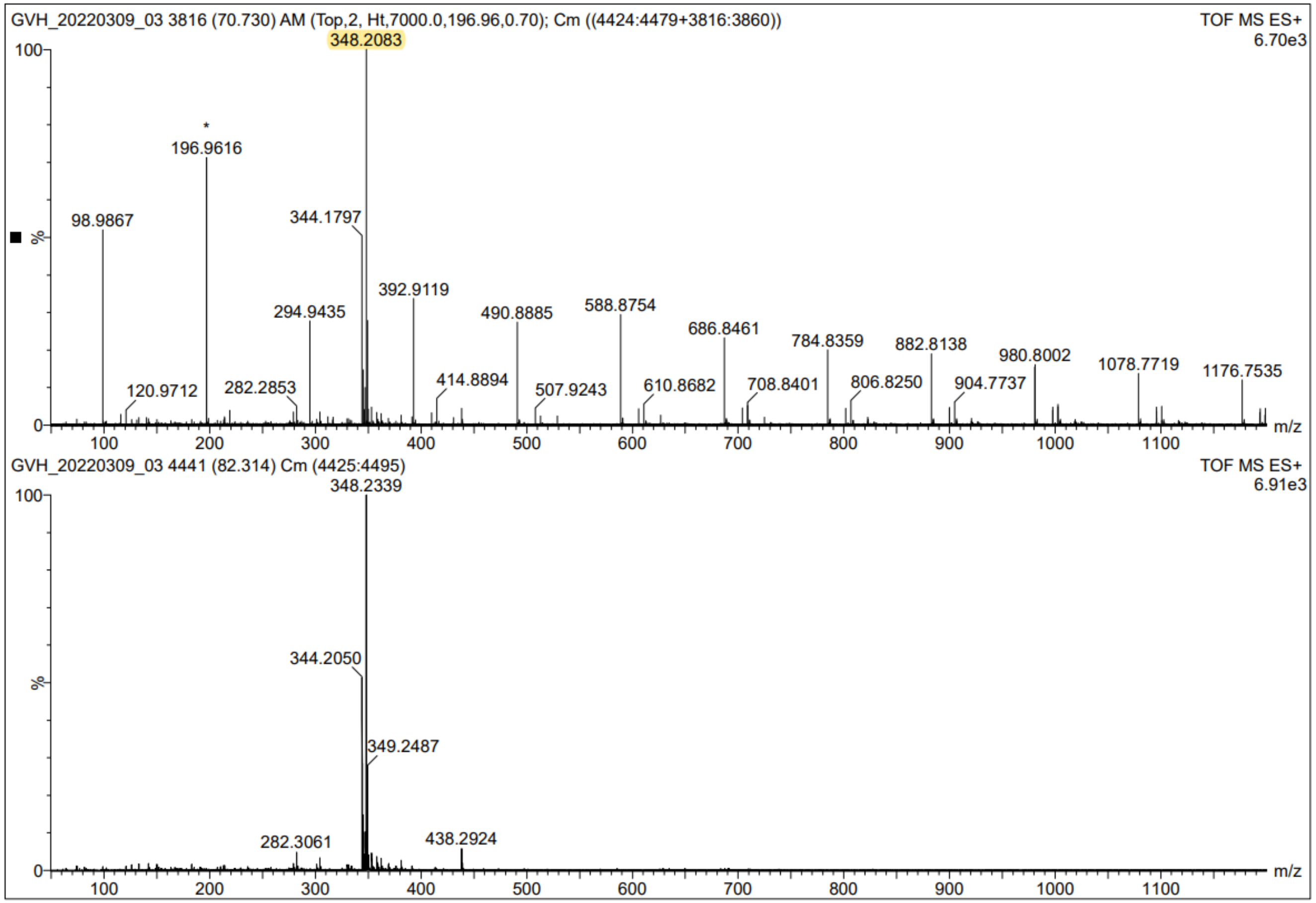

Compound 102

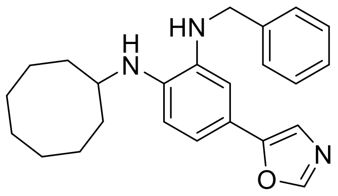

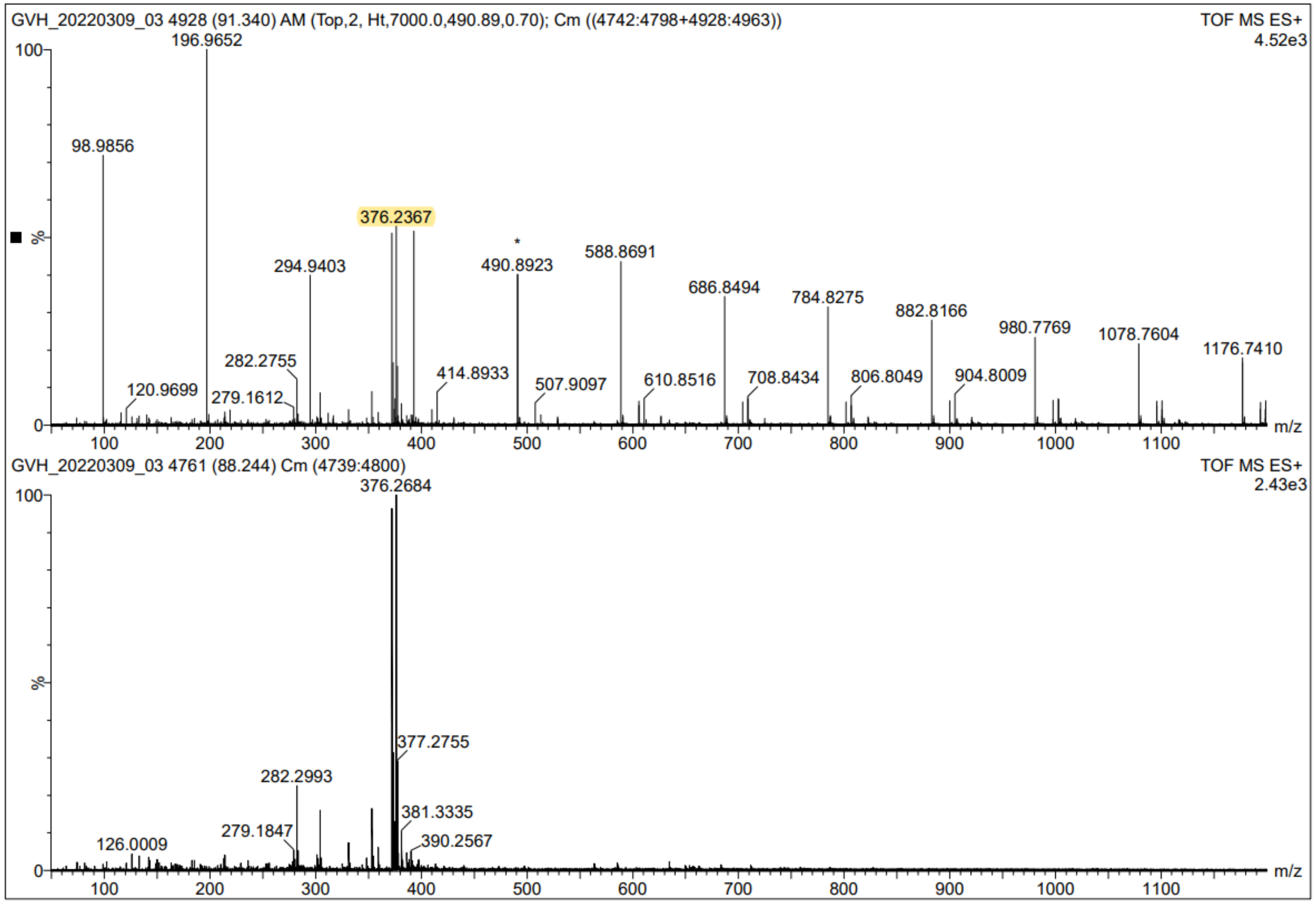

Compound 103

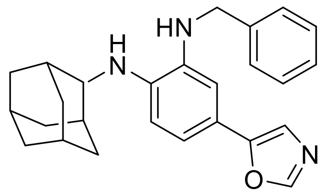

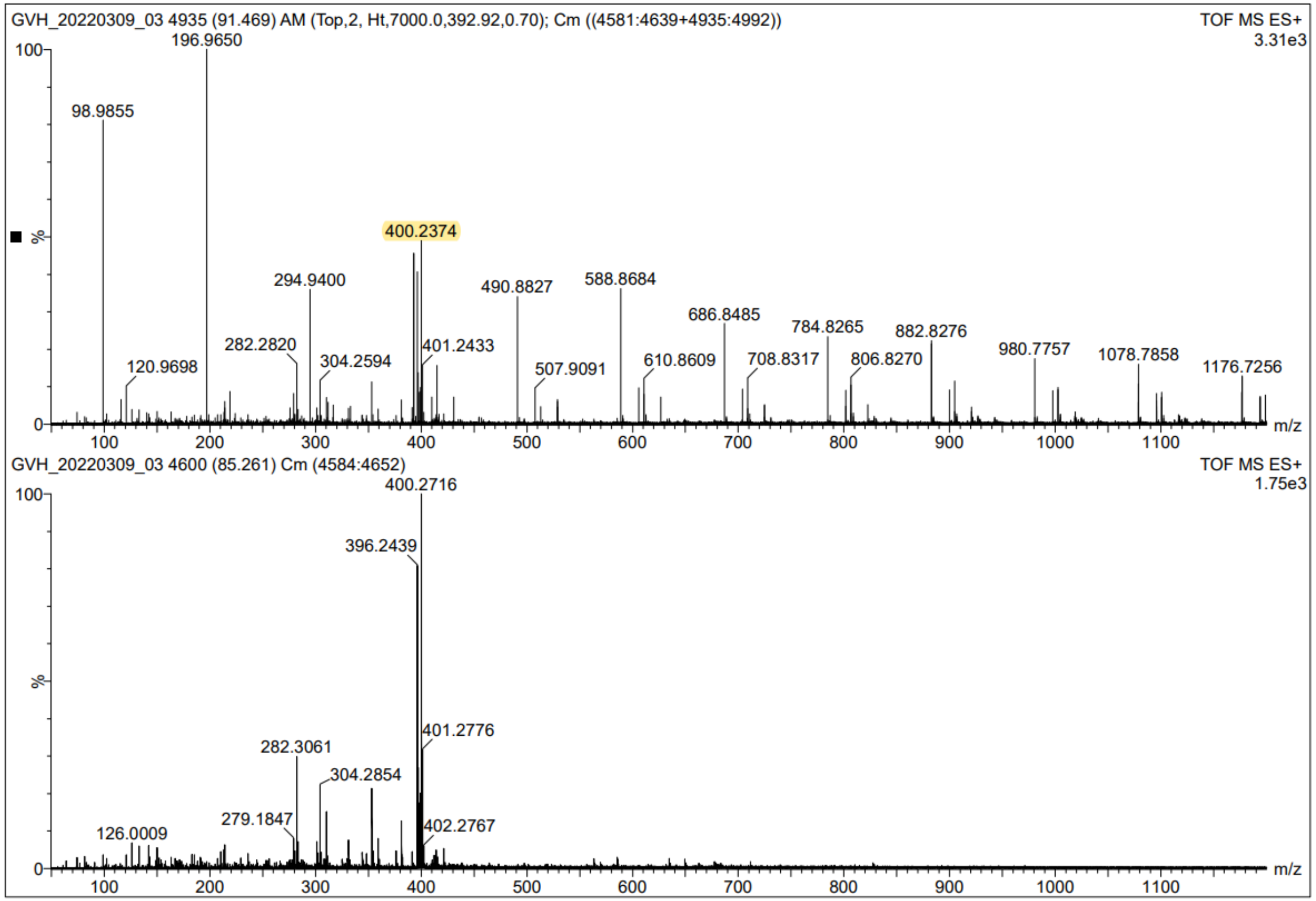

Compound 104

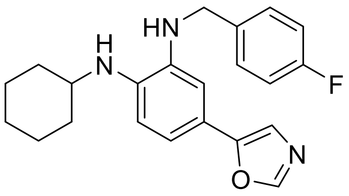

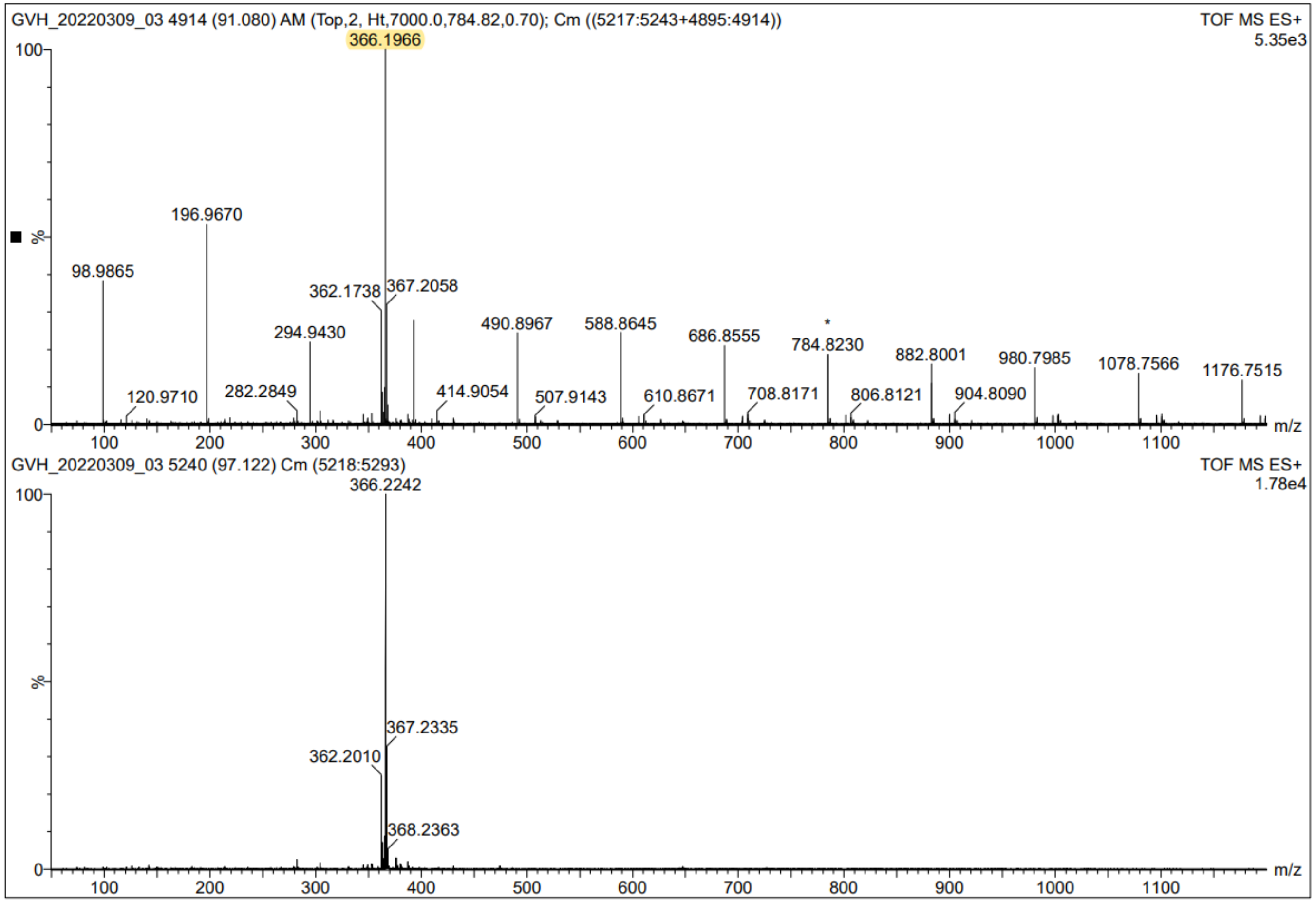

Compound 105

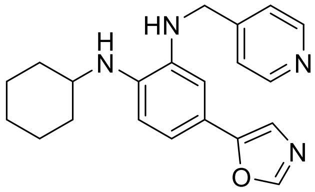

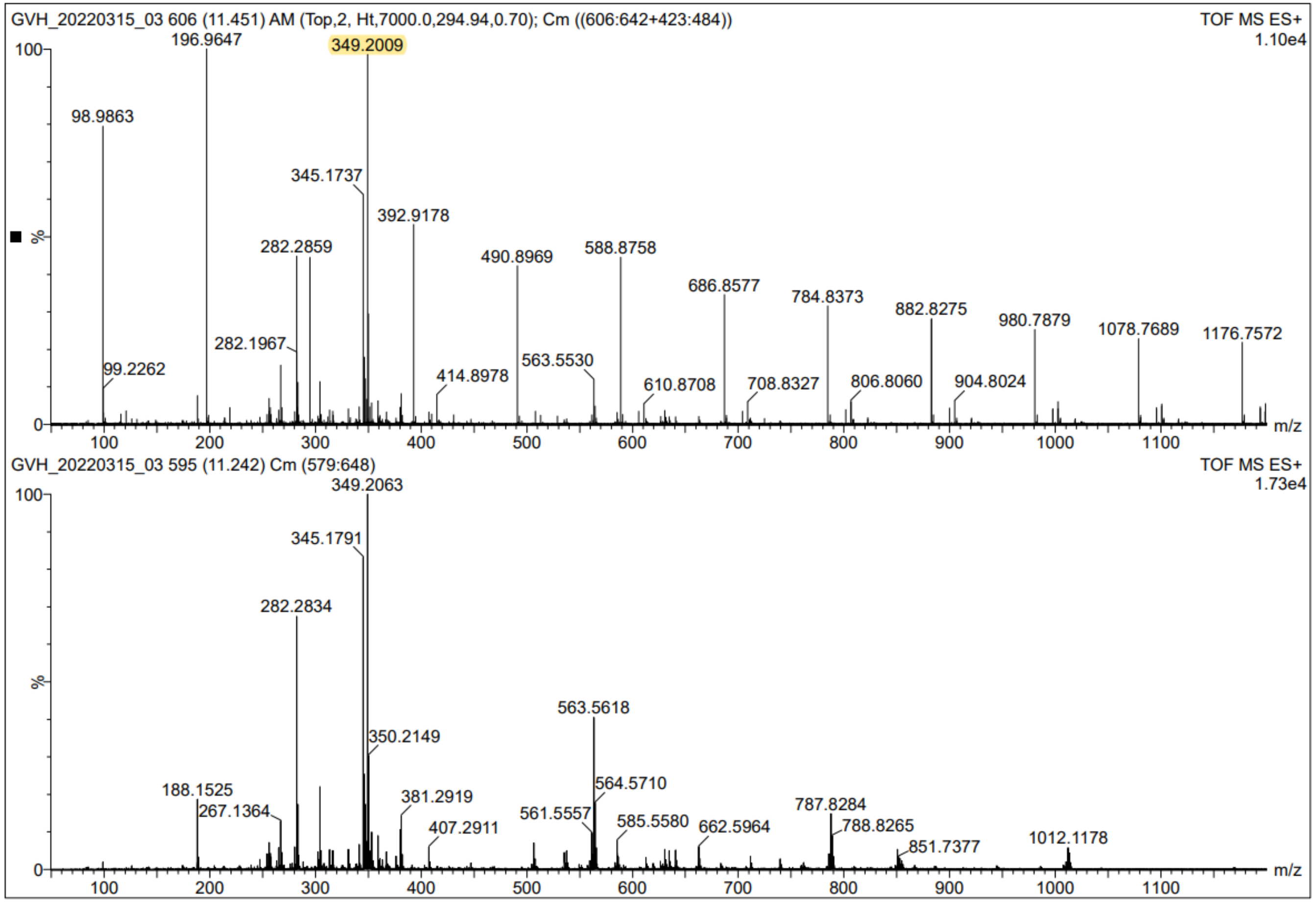

UPLC analyses of selected compounds

Compound 46

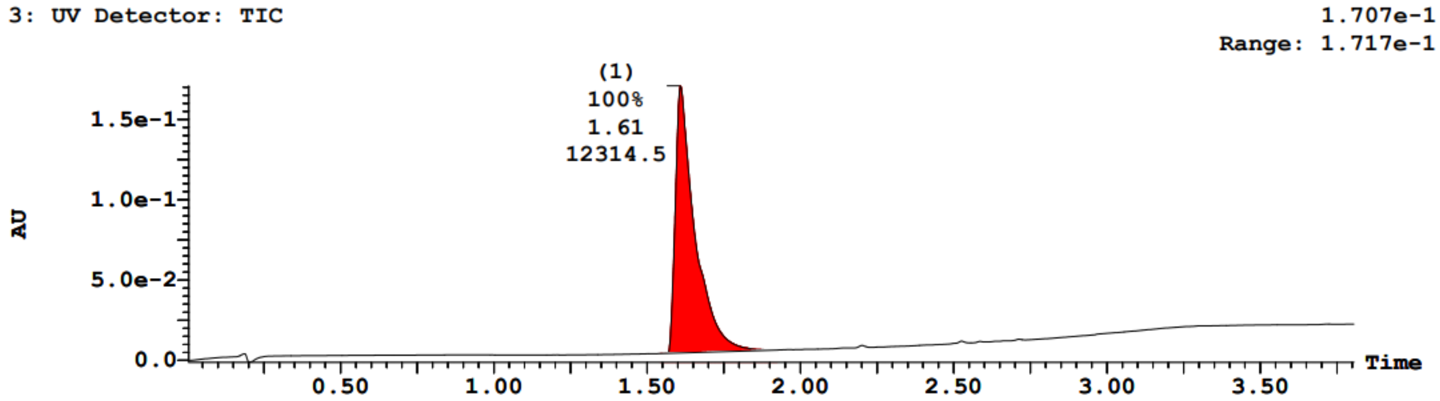

Peak Number	Compound	Time	AreaAbs	Area %Total	Width	Height	Mass Found
1		1.61	1e+004	100.00	0	2e+005	

Compound 47

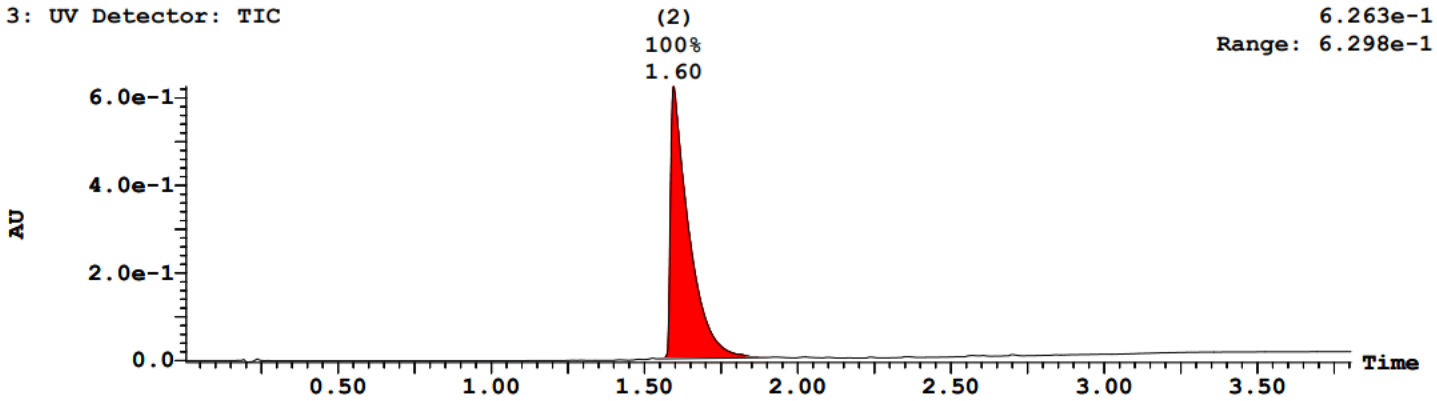

Peak Number	Compound	Time	AreaAbs	Area %Total	Width	Height	Mass Found
2		1.60	4e+004	100.00	0	6e+005	

Compound 50

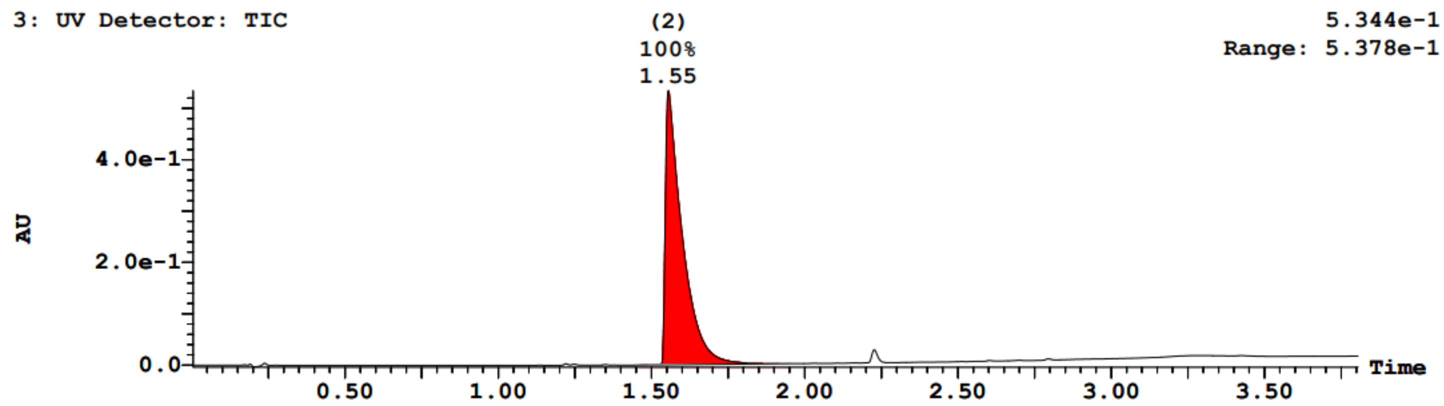

Peak Number	Compound	Time	AreaAbs	Area %Total	Width	Height	Mass Found
2		1.55	3e+004	100.00	0	5e+005	

Compound 94 (UAMC-4749)

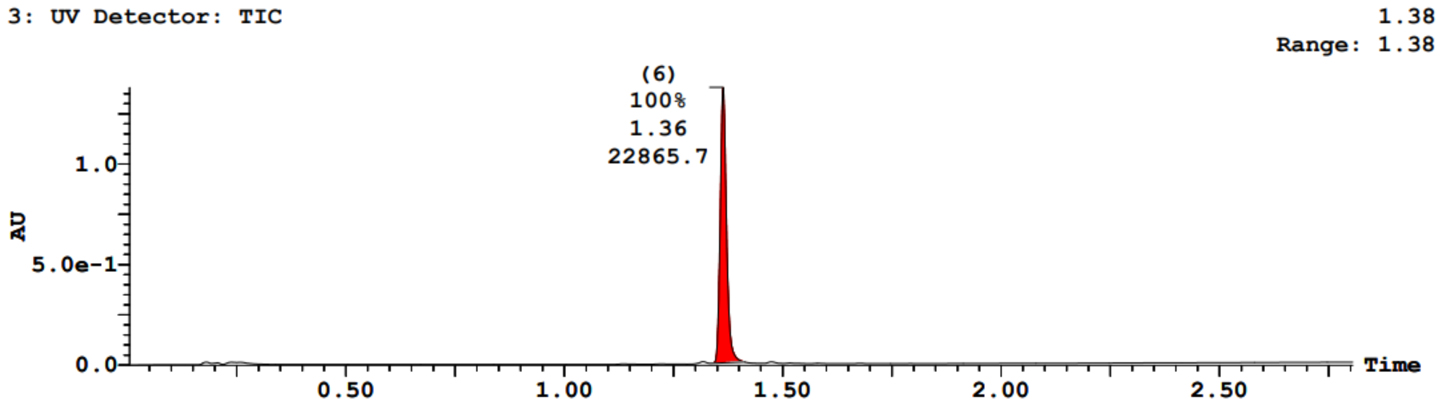

Peak Number	Compound	Time	AreaAbs	Area %Total	Width	Height	Mass Found

Compound 95

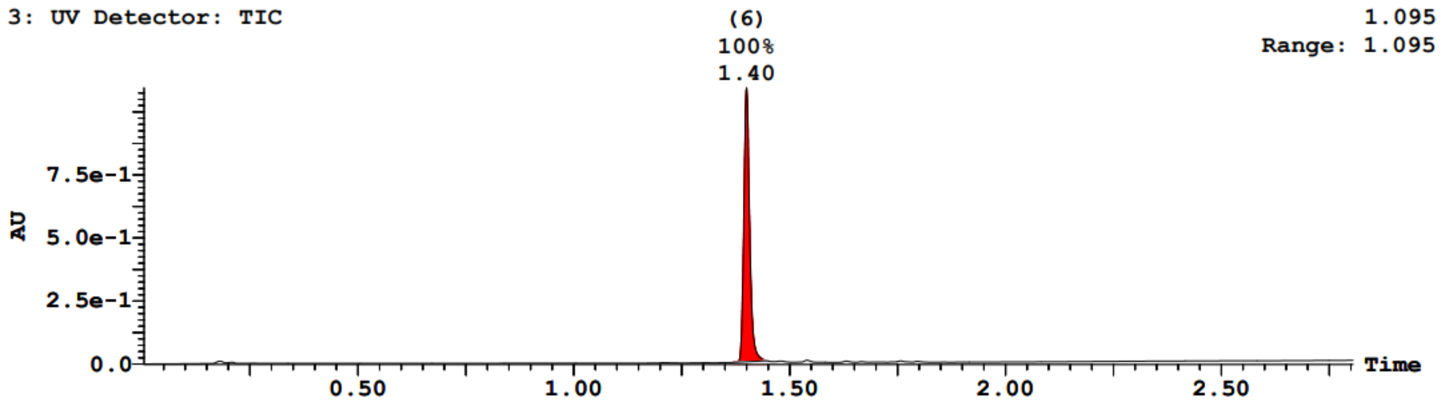

Peak Number	Compound	Time	AreaAbs	Area %Total	Width	Height	Mass Found
6		1.40	2e+004	100.00	0	1e+006	

Compound 96 (UAMC-4821)

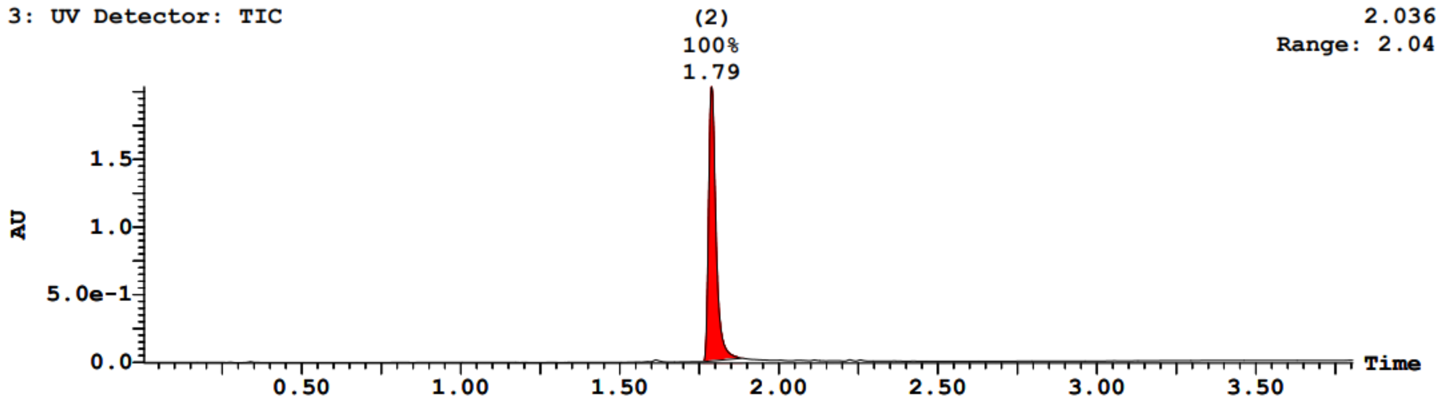

Peak Number	Compound	Time	AreaAbs	Area %Total	Width	Height	Mass Found
2		1.79	5e+004	100.00	0	2e+006	

Inhibition of ML162-Induced Ferroptosis in HT1080 human fibrosarcoma cells – dose-response curves of selected compounds

Cell death percentage was calculated as:

$$\frac{100 - (X - 100\%\ inhib)}{0\%\ inhib - 100\%\ inhib}\ x\ 100$$

Curves were plotted in Spotfire software, and IC_{50} values were calculated using logistic regression curves at 16 h. The curves of selected compounds **46, 47, 50** from the amides series and isoxazoles **94-96** are reported in figure S1.

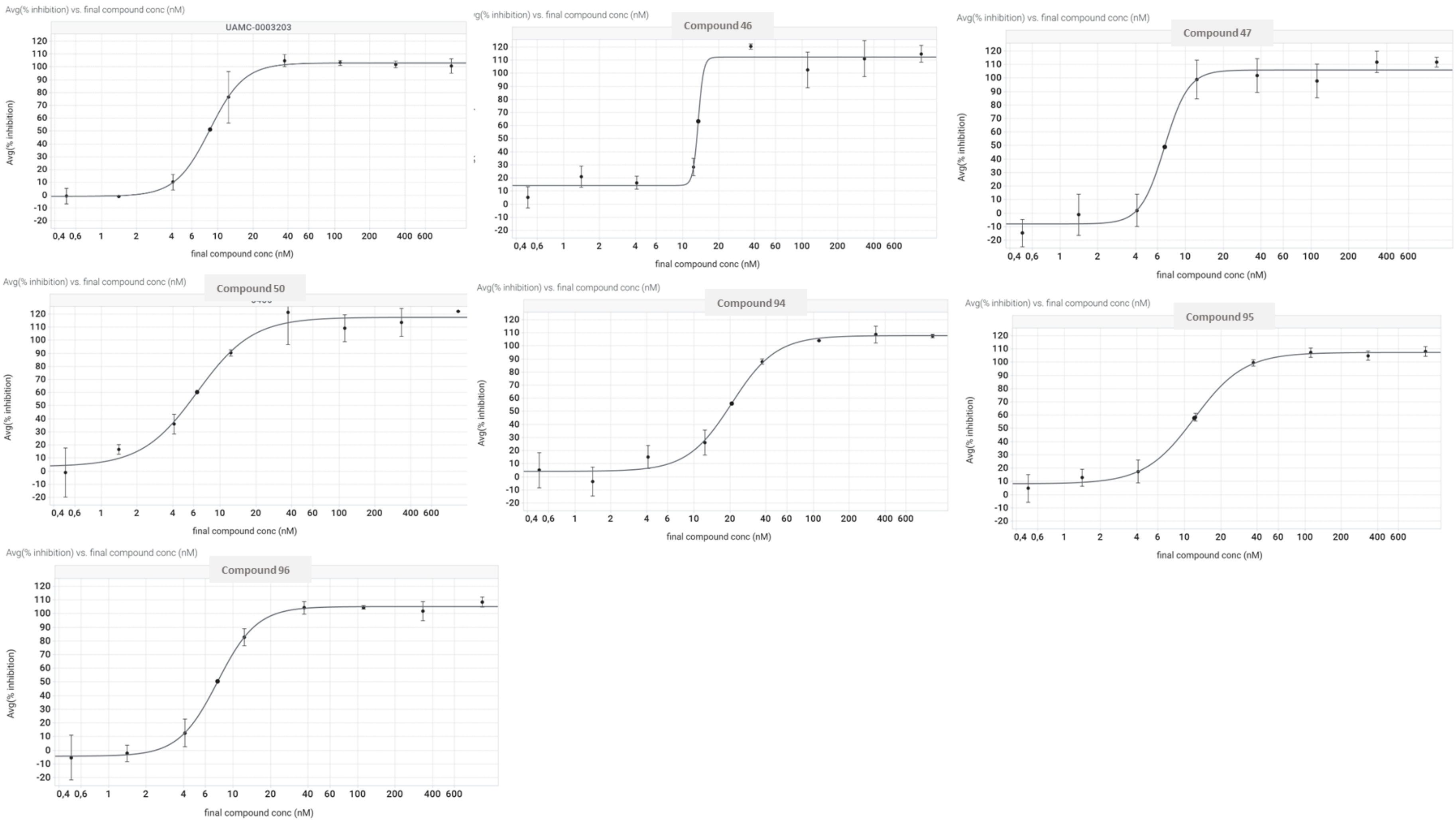

Figure S1. *Dose-response curves used to determine IC₅₀ values for selected compounds. Data presented in the graphs are from a single experiment (n=1).*

Fluorescence-Enabled Inhibited Autoxidation (FENIX assay) – stoichiometry, k_{inh} and Logk_{inh}

Table S1. Comparison of the stoichiometry (n) inhibition rate constants (k_{inh}), and the logarithmic values of the inhibition rate constants ($\log(k_{inh})$) calculated as a result of the FENIX assay with RTAs at 4 µM.

Name	n	k_{inh}	Logk_{inh}
**PMC**	2.0	5.1	4.71
**Fer-1**	3.0	3.8	4.57
**UAMC-3203**	2.9	1.5	4.16
**BENZAMIDES**			
**13**	0	0.1	3.00
**14**	0	0.1	3.00
**15**	5.0	0.6	3.80
**16**	0	0.1	3.00
**35**	2.1	8.9	4.84
**36**	2.2	9.1	4.96
**37**	2.8	5.1	4.71
**38**	2.9	4.6	4.67
**39**	2.0	6.4	4.81
**40**	2.2	6.8	4.83
**41**	3.5	3.5	4.54
**42**	3.7	3.4	4.54
**43**	2.9	9.1	4.55
**44**	2.6	4.8	4.68
**45**	2.6	5.5	4.74
**46**	3.0	1.9	4.28
**47**	3.8	1.7	4.22
**48**	3.9	1.5	4.19
**49**	2.7	2.2	4.38

**50**	3.7	1.4	4.53
**51**	3.6	1.4	4.15
**5-METHYL-OXAZOL-2-YL SCAFFOLD**			
**73**	3.7	2.1	4.31
**74**	3.8	4.1	4.61
**75**	3.8	6.4	4.80
**76**	3.2	3.7	4.57
**77**	2.5	3.9	4.60
**78**	2.4	4.5	4.66
**79**	2.1	13.7	5.13
**80**	2.0	9.9	4.99
**81**	2.0	8.2	4.91
**82**	1.4	3.6	4.56
**83**	1.8	24.6	5.39
**84**	1.1	12.8	5.10
**85**	1.6	57.8	5.76
**86**	0.9	20.5	5.31
**OXAZOL-5-YL SCAFFOLD**			
**94**	3.0	1.9	4.28
**95**	3.1	2.8	4.44
**96**	2.8	3.4	4.53
**97**	2.2	4.6	4.67
**98**	2.2	6.5	4.81
**99**	2.2	5.1	4.70
**100**	2.7	5.6	4.75
**101**	2.3	6.1	4.79
**102**	1.9	11.7	5.07
**103**	1.8	4.6	4.66
**104**	2.3	7.4	4.89
**105**	1.6	7.8	4.87

N and k_{inh} were determined using PMC as a standard, which has an established stoichiometry of 2. Kinetic data are given as averages of two independent measurements (n=2, standard deviation not showed), run in triplicate for each compound.[1]

$$\text{Uninhibited autoxidation: } \frac{\Delta[LOOH]}{\Delta t} = Kp\ [LH]\left(\frac{Ri}{2Kt}\right)^{\frac{1}{2}}$$

The equation expresses the kinetics of hydrocarbon autoxidation. The rate of hydroperoxide formation is proportional to the concentration of the substrate (L−H), the rate constant for H-atom abstraction (kp), from the substrate by a chain-carrying peroxyl radical (kp), the rate of initiation (Ri), and the reaction of two chain-carrying peroxyl radicals (kt). Kp = chain-carrying peroxyl radical; Ri = rate of initiation; kt = rate of termination.

$$\text{Inhibited autoxidation: } \frac{\Delta[LOOH]}{\Delta t} = \frac{Kp\ [LH]Ri}{n\ Kinh\ [RTA]}$$

The equation expressed the inhibition of the autoxidation process by RTAs in homogeneous solution and lipid bilayer. Compounds with high k_{inh} or n for reaction with peroxyl radicals inhibit the autocatalytic LOOH formation. Kp = chain-carrying peroxyl radical; Ri = rate of initiation; n = stochiometry; k_{inh} = rate inhibition constant.

In Figures S2-S4 are reported the curves of all compounds divided by series.

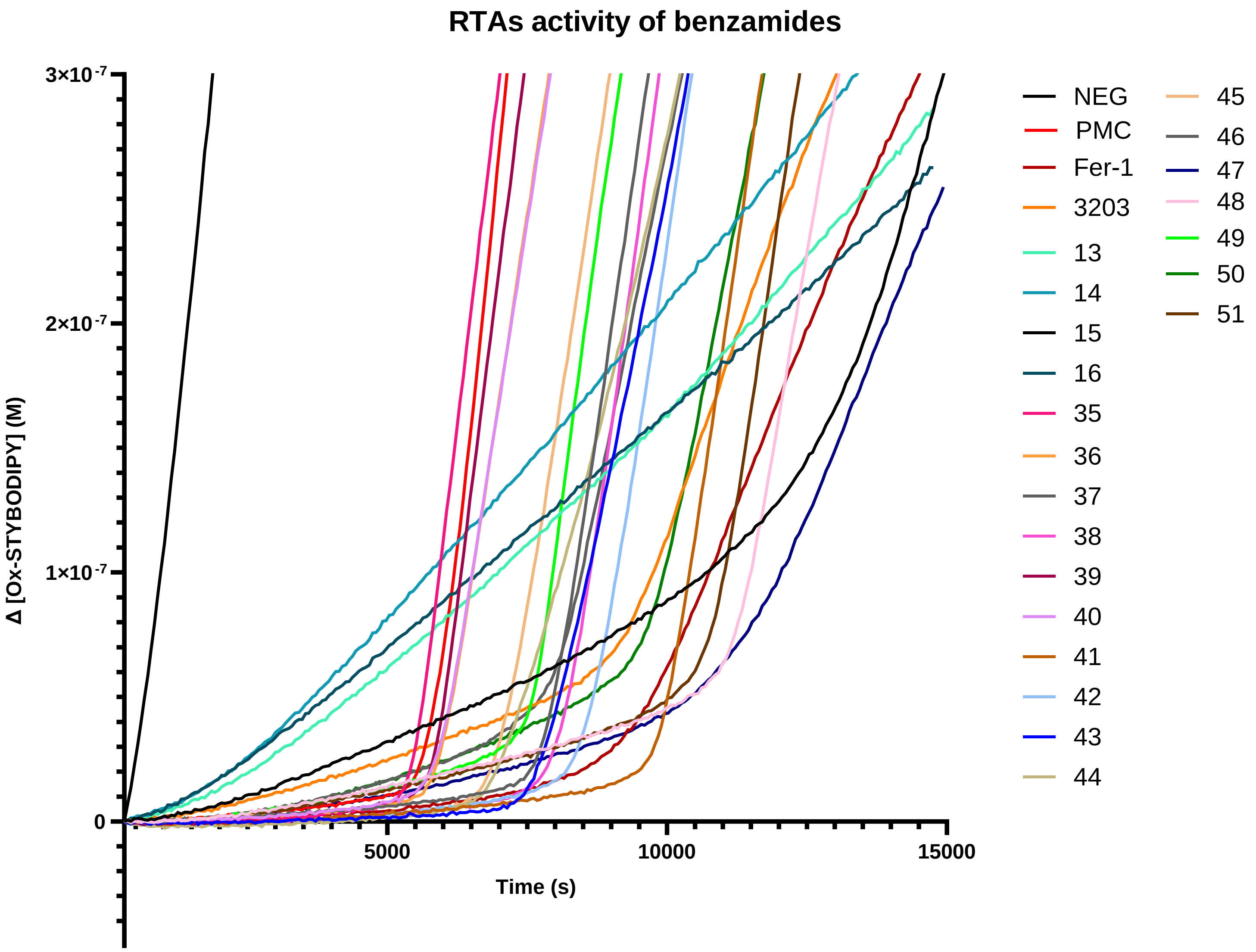

Figure S2. *RTA activity of benzamides* ***13-16*** *and* ***35-51*** *assessed through the FENIX assay in comparison with 2,2,5,7,8-pentamethyl-6-hydroxychromane (PMC, in red), Fer-1 (in brown), UAMC-3203 (in orange) and negative (in black).*

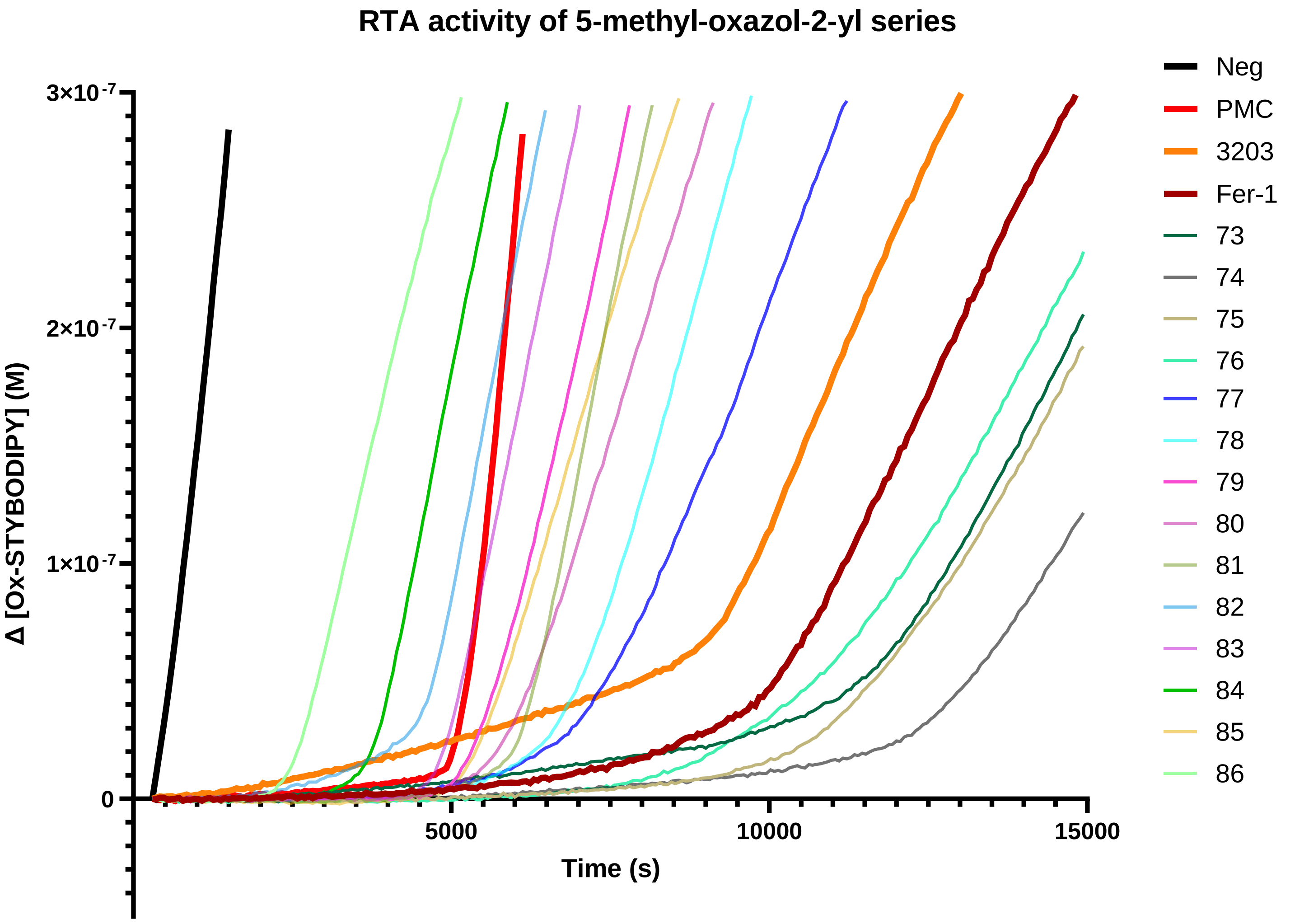

Figure S3. *RTA activity of 5-methyl-oxazol-2-yl* ***73-86*** *assessed through the FENIX assay in comparison with PMC (in red), Fer-1 (in brown), UAMC-3203 (in orange) and negative (in black).*

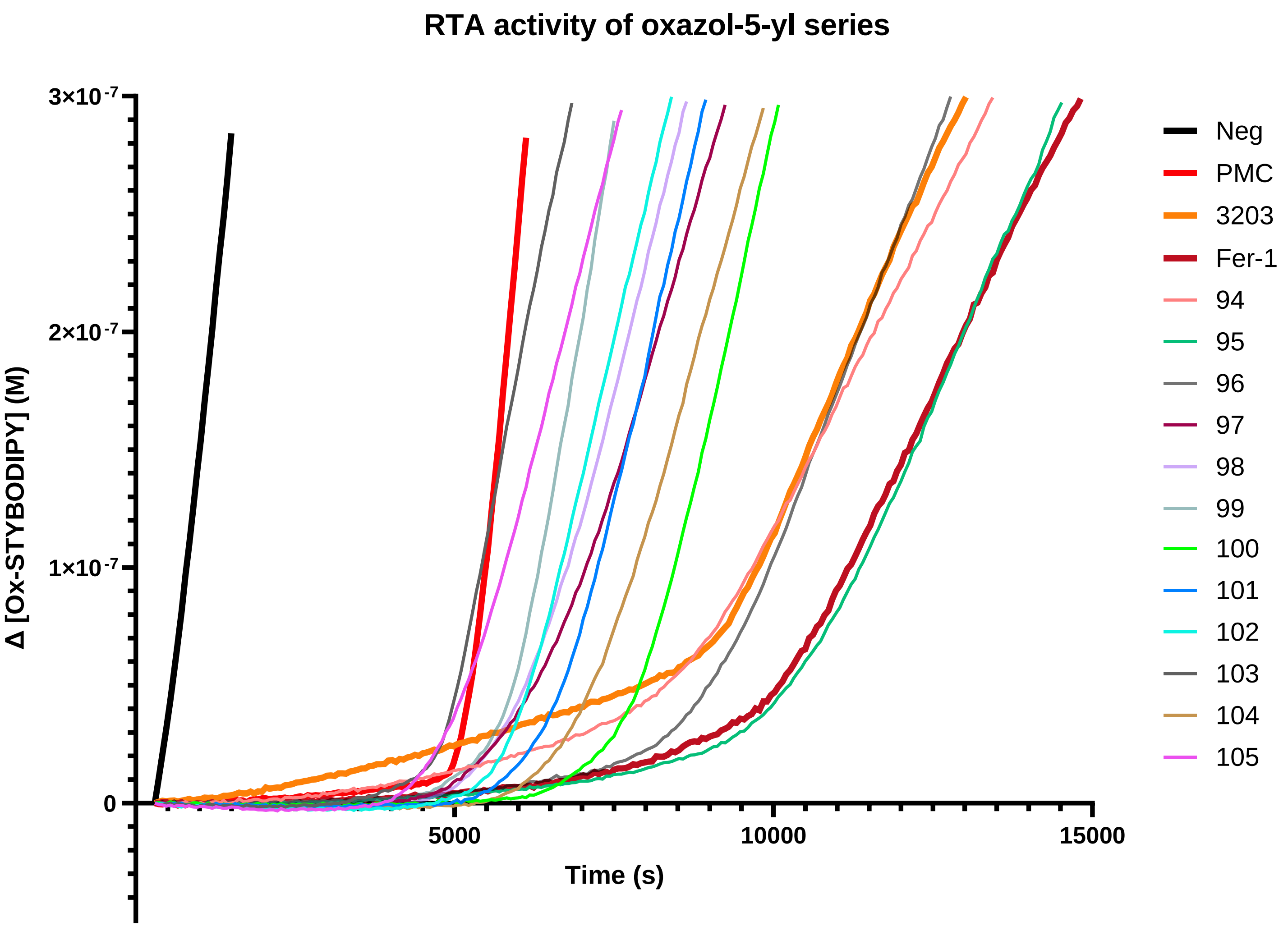

Figure S4. *RTA activity of oxazol-5-yl **94-105** assessed through the FENIX assay in comparison with PMC(in red), Fer-1 (in brown), UAMC-3203 (in orange) and negative (in black).*

Experimental data from kinetic solubility for selected compounds

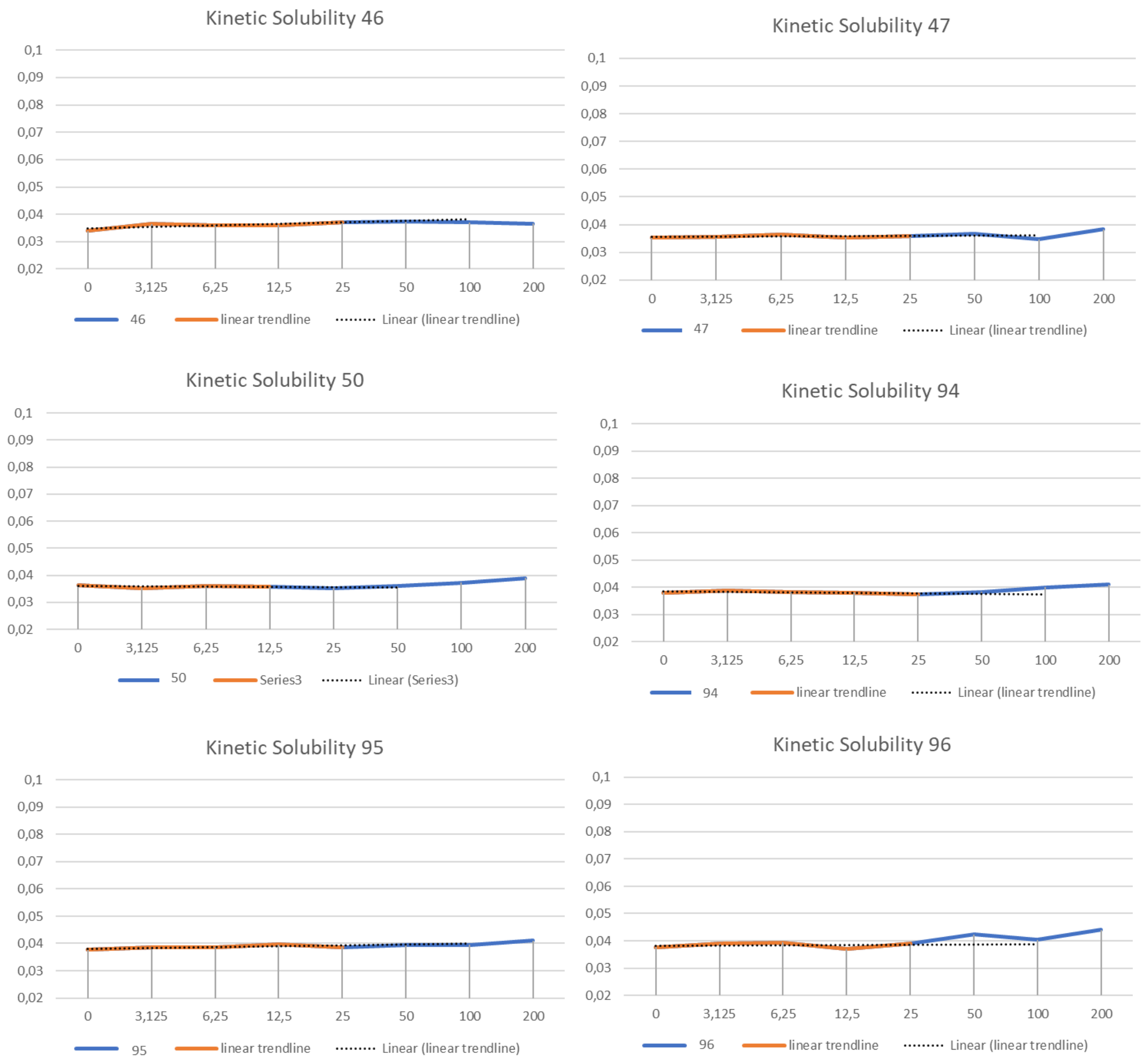

Figure S5. *Graphical representation of the kinetic solubility of compounds* ***46, 47, 50, 94, 95*** *and* ***96***

Microsomal stability - data processing description and detailed results for selected compounds

Data processing

All obtained readouts were processed using Microsoft Excel. From a plot of ln peak area ratio (compound peak area / internal standard peak area) against time, the gradient of a line is determined by linear regression. Subsequently several parameters are calculated using the equations below:

- k: elimination rate constant = (- gradient)
- $t_{1/2}$: half life time (min) = $\frac{\ln 2}{k}$. For the half life, the limit of detection was fixed at 250 min
- liver microsome concentration = 0.5 mg/ml
- Cl_{int}: intrinsic clearance liver microsomes (µL/(min/mg)) = $\frac{ln2}{t_{1/2}}\, x \,\frac{ml\ incubation}{mg\ protein}\, x\ 1000$
- MPPGL mg microsomal protein per gram liver (mg/g), which corresponds to 40 mg/g in human microsmes and 45 mg/g in mouse microsomes
- GLPKBW: gram of liver per body weight (g/kg), which corresponds 24 g/kg human microsomes and 60 g/kg mouse microsomes
- Cl_{int}: intrinsic clearance whole liver (mL/min/kg) = $\frac{Cl_{int}(liver\ microsomes)}{1000}\ x\ MPPGL\ x\ GLPKBW$
- QH: liver blood flow (mL/min/kg), which corresponds to 20 mL/min/kg human microsomes and 90 mL/min/kg mouse microsomes
- fub: fraction unbound in plasma = 1 (representing the limit case where a compound is highly bound to plasma protein)
- Cl_H: hepatic clearance assuming the well-stirred model (mL/min/kg) = $\frac{QH\ x\ fub\ x\ Cl_{int}}{QH+(fub\ x\ Cl_{int})}$
- ER : extraction rate = $\frac{CL_H}{QH}$

Reference for intrinsic clearance:

Cl_{int} (µL/min/mg)	Human	Mouse
Low	< 8.6	<13.1
High	> 47.0	> 71.1

Reference for Extraction rate:

ER	Values
Low	< 3.0
High	> 7.0

These parameters are based on https://www.evotec.com/en/drug-metabolism/microsomal-stability.

Table S2. Calculated parameters for selected compounds to determine human microsomal stability

Human microsomal stability

Compound	k	t1/2 (min)	Cl_{int} liver microsomes (microL/min*mg)	Cl_{int} whole liver (ml/min/kg)	Cl_H (ml/min/kg)	ER
**3203**	-0.0041352	167.6	8.3	7.9	5.7	0.28
**Verapamil**	-0.0566397	12.3	112.9	108.4	16.9	0.84
**Dextrometorphan**	-0.0143564	45.2	30.7	29.4	11.9	0.60
**46**	-0.0028203	245.8	5.6	5.4	4.6	0.21
**47**	-0,0001134	>250	5.5	5.3	4.2	0.21
**50**	-0,0005785	>250	5.5	5.3	4.2	0.21
**94**	-0.0056204	123.3	11.2	12.8	7.0	0.35
**95**	-0.0289990	239.0	5.8	5.6	4.4	0.22
**96**	-0,0057138	121.3	11.4	11.0	7.1	0.35

Data are expressed as mean of 2 experiments (n=2). The mean value is calculated based on the concentration of compound left at specific time points expressed as % of the concentration at t_0 (see experimental part for details). The percentage of compound was used for the calculation of the intrinsic clearance and all the other parameters reported in Table S2 and Table S3. The variability of results at 45 min < 10%.

Table S3. Calculated parameters for selected compounds to determine mouse microsomal stability

Mouse microsomal stability

Compound	k	t1/2 (min)	Cl_{int} liver microsomes (microL/min*mg)	Cl_{int} whole liver (ml/min/kg)	Cl_H (ml/min/kg)	ER
**3203**	-0.0041352	167.6	8.3	7.9	5.7	0.28
**Diazepam**	-0.3534877	1.9	707.0	1908.8	85.9	0.95
**Diphenhydramine**	-0.0225678	30.7	45.1	121.9	51.8	0.58
**46**	-0.0071035	97.6	14.2	38.4	26.9	0.30
**47**	-0.0084283	82.2	16.9	45.5	30.2	0.34
**50**	-0.0026669	>250	5.5	15.0	12.8	0.14
**94**	-0.0156689	44.2	31.3	84.6	43.6	0.48
**95**	-0.0630133	11.0	41.9	113	50.1	0.56
**96**	-0.0224417	30.9	44.9	121.2	51.6	0.56

Data were expressed as mean of 2 experiments (n=2). The mean value is calculated based on the concentration of compound left at specific time points expressed as % of the concentration at t_0(see experimental part for details). The percentage of compound was used for the calculation of the intrinsic clearance and all the other parameters reported in Table S2 and Table S3. The variability of results at 45 min < 10%.

In vivo experimental mouse PK – experimental details and concentration in different tissues

Plasma concentrations were expressed in ng/mL and tissue concentrations in mg/kg. Individual and mean concentrations (n=3 individual values) with standard deviation (SD) were reported.

- Pharmacokinetic analysis was performed using WinNonlin Phoenix® software (Certara, version 8.3) from animal plasma concentrations, non-compartmental analysis, and the target dose. The following PK parameters were calculated, if the data permits:
- C_o (ng/mL): Initial concentration
- C_{max} (ng/mL): maximum observed concentration, occurring at Tmax
- C_{max} /Dose (ng/mL): dose normalised C_{max}
- $AUC_{0\text{-}last}$ (ng*h/mL); area under the plasma concentration versus time curve up to last quantifiable concentration, and/or up to infinity $AUC_{0\text{-}inf}$ (ng*h/mL) were calculated according to the linear up log down method.
- t½ (h); Apparent terminal elimination half-life only reported if three or more time points in the elimination phase – excluding the Tmax - were used for linear regression, and if rad 2>0.9, and if % of AUC extrapolation to infinity is lower than 20%,
- CL (mL/min/kg): total clearance determined after intravenous administration
- V_{ss} (L/kg): Apparent volume of distribution at equilibrium determined after intravenous administration (Vss/F after oral dosing)
- Bioavailability (%)
- The following considerations were made when calculating and reporting PK parameters:
- At time points prior to Cmax and between measurable concentrations any concentrations below the limit of quantification was set to zero.
- If sample concentration is below the lower limit of quantification (BLQ) after the last quantifiable data point, the data was treated as BLQ as considered as 0.
- Where blood samples are not present (e.g., a blood sample could not be taken) it was excluded and treated as missing

- If insufficient data are available for the definition of a terminal phase of the plasma vs. concentration time profile, then parameters which rely on this definition will not be reported, e.g t½, AUC0-∞
- Parameters estimates are rounded as appropriate to the accuracy of the measurement, and should be calculated to 3 significant figures where bioanalytical data permits

Table S4. Plasma distribution of compounds **94** and **96** (UAMC-4821) after intravenous administration (IV) at 8 time points (ng/ml)

**Matrix**	**Time (h)**	**C (ng/mL)**	
		**94**	**96 (UMC-4821)**
Plasma	0.05	11190 ± 5487	13067 ± 1751
	0.25	3130 ± 1285	6540 ± 601
	0.5	675 ± 229	5497 ± 1314
	1	773 ± 87.8	3457 ± 1164
	2	103 ± 18.4	650 ± 119
	4	22.3 ± 5.27	191 ± 63.6
	8	117 ± 105	52.3 ± 9.66
	24	BLQ	2.49 ± 0.116

BLQ = below the detection limits considered as 0. Each mouse received an *iv* dose at 10 mg/kg. The samples were homogenized and further processed by protein precipitation (n = 3, except n = 6 at 0.05 h) and centrifugation. Analysis of the samples was conducted by LC-MS.

Table S5. Plasma distribution of compounds **94** and **96** (UAMC-4821) after oral administration (PO) at 7 time points (ng/ml)

**Matrix**	**Time (h)**	**C (ng/mL)**	
		**94**	**96 (UAMC-4821)**
Plasma	0.25	285 ± 78.1	2370 ± 483
	0.5	289 ± 114	2310 ± 416
	1	186 ± 73.2	2207 ± 788
	2	89 ± 66.6	1135 ± 523
	4	15.0 ± 1.88	408 ± 149
	8	5.41[a]	203 ± 105
	24	BLQ	2.34 ± 0.46 [b]

BLQ = below the detection limits considered as 0. Each mouse received a *po* dose at 10 mg/kg. The samples were homogenized and further processed by protein precipitation (n = 3) and centrifugation. Analysis of the samples was conducted by LC-MS. [a] For this measurement only one sample was processed (n = 1). [b] For this measurement only two samples were processed (n = 2).

Table S6. Distribution of compounds **94** and **96** (UAMC-4821) in liver, lung, heart and kidney at 0.5 h, 4 h and 24 h in ng/g

Matrix	Time (h)	ng/g	
		**94**	**96 (UAMC-4821)**
**Liver**	0.5	1117 ± 587	10333 ± 3183
	4	109 ± 18.0	714 ± 12.9
	24	BLQ	48.5 ± 5.79
**Lung**	0.5	1077 ± 103	2467 ± 638
	4	118 ± 18.8	226 ± 131
	24	83.2 ± 1.72	41.3 [a]
**Heart**	0.5	461 ± 107	859 ± 278
	4	BLQ	68.5 ± 8.05
	24	82.5 [a]	BLQ
**Kidney**	0.5	3403 ± 1767	9230 ± 3193
	4	367 ± 127	1015 ± 447
	24	BLQ	BLQ
**Brain**	0.5	375 ± 27.8	1223 ± 361
	4	132 ±17.6	103 ± 4.61
	24	175 ± 19.7	123 ± 28.8

BLQ = below the detection limits considered as 0. Each mouse received an *iv* dose at 10 mg/kg. The samples were homogenized and further processed by protein precipitation (n = 3) and centrifugation. Analysis of the samples was conducted by LC-MS. [a] For this measurement only one sample was processed (n = 1).

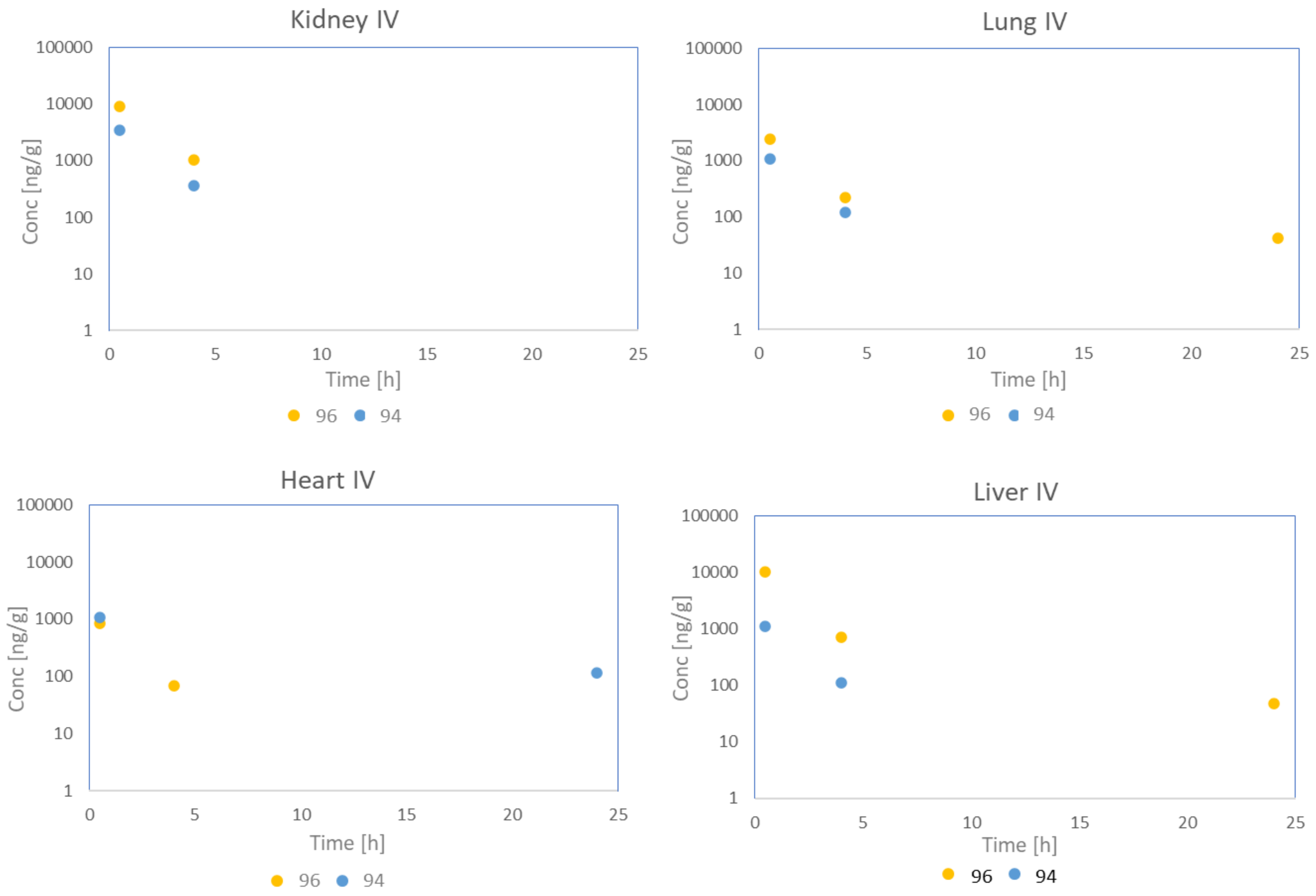

Figure S6. *Graphical representation of the concentrations of compound **94** and **96** (UAMC-4821) in different organs at 0.5 h, 4 h and 24 h.*

References

(1) Shah, R.; Farmer, L. A.; Zilka, O.; Kessel, A. T. M. Van; Pratt, D. A. Beyond DPPH : How Best to Assess and Screen for Inhibitors of Lipid Peroxidation and Associated Ferroptotic Cell Death. *ACS Cent. Sci.* **2019**, *26* (11), 1594–1607. https://doi.org/https://doi.org/10.1016/j.chembiol.2019.09.007.
